# The Japanese Clinical Practice Guidelines for Management of Sepsis and Septic Shock 2020 (J-SSCG 2020)

**DOI:** 10.1186/s40560-021-00555-7

**Published:** 2021-08-25

**Authors:** Moritoki Egi, Hiroshi Ogura, Tomoaki Yatabe, Kazuaki Atagi, Shigeaki Inoue, Toshiaki Iba, Yasuyuki Kakihana, Tatsuya Kawasaki, Shigeki Kushimoto, Yasuhiro Kuroda, Joji Kotani, Nobuaki Shime, Takumi Taniguchi, Ryosuke Tsuruta, Kent Doi, Matsuyuki Doi, Taka-aki Nakada, Masaki Nakane, Seitaro Fujishima, Naoto Hosokawa, Yoshiki Masuda, Asako Matsushima, Naoyuki Matsuda, Kazuma Yamakawa, Yoshitaka Hara, Masaaki Sakuraya, Shinichiro Ohshimo, Yoshitaka Aoki, Mai Inada, Yutaka Umemura, Yusuke Kawai, Yutaka Kondo, Hiroki Saito, Shunsuke Taito, Chikashi Takeda, Takero Terayama, Hideo Tohira, Hideki Hashimoto, Kei Hayashida, Toru Hifumi, Tomoya Hirose, Tatsuma Fukuda, Tomoko Fujii, Shinya Miura, Hideto Yasuda, Toshikazu Abe, Kohkichi Andoh, Yuki Iida, Tadashi Ishihara, Kentaro Ide, Kenta Ito, Yusuke Ito, Yu Inata, Akemi Utsunomiya, Takeshi Unoki, Koji Endo, Akira Ouchi, Masayuki Ozaki, Satoshi Ono, Morihiro Katsura, Atsushi Kawaguchi, Yusuke Kawamura, Daisuke Kudo, Kenji Kubo, Kiyoyasu Kurahashi, Hideaki Sakuramoto, Akira Shimoyama, Takeshi Suzuki, Shusuke Sekine, Motohiro Sekino, Nozomi Takahashi, Sei Takahashi, Hiroshi Takahashi, Takashi Tagami, Goro Tajima, Hiroomi Tatsumi, Masanori Tani, Asuka Tsuchiya, Yusuke Tsutsumi, Takaki Naito, Masaharu Nagae, Ichiro Nagasawa, Kensuke Nakamura, Tetsuro Nishimura, Shin Nunomiya, Yasuhiro Norisue, Satoru Hashimoto, Daisuke Hasegawa, Junji Hatakeyama, Naoki Hara, Naoki Higashibeppu, Nana Furushima, Hirotaka Furusono, Yujiro Matsuishi, Tasuku Matsuyama, Yusuke Minematsu, Ryoichi Miyashita, Yuji Miyatake, Megumi Moriyasu, Toru Yamada, Hiroyuki Yamada, Ryo Yamamoto, Takeshi Yoshida, Yuhei Yoshida, Jumpei Yoshimura, Ryuichi Yotsumoto, Hiroshi Yonekura, Takeshi Wada, Eizo Watanabe, Makoto Aoki, Hideki Asai, Takakuni Abe, Yutaka Igarashi, Naoya Iguchi, Masami Ishikawa, Go Ishimaru, Shutaro Isokawa, Ryuta Itakura, Hisashi Imahase, Haruki Imura, Takashi Irinoda, Kenji Uehara, Noritaka Ushio, Takeshi Umegaki, Yuko Egawa, Yuki Enomoto, Kohei Ota, Yoshifumi Ohchi, Takanori Ohno, Hiroyuki Ohbe, Kazuyuki Oka, Nobunaga Okada, Yohei Okada, Hiromu Okano, Jun Okamoto, Hiroshi Okuda, Takayuki Ogura, Yu Onodera, Yuhta Oyama, Motoshi Kainuma, Eisuke Kako, Masahiro Kashiura, Hiromi Kato, Akihiro Kanaya, Tadashi Kaneko, Keita Kanehata, Ken-ichi Kano, Hiroyuki Kawano, Kazuya Kikutani, Hitoshi Kikuchi, Takahiro Kido, Sho Kimura, Hiroyuki Koami, Daisuke Kobashi, Iwao Saiki, Masahito Sakai, Ayaka Sakamoto, Tetsuya Sato, Yasuhiro Shiga, Manabu Shimoto, Shinya Shimoyama, Tomohisa Shoko, Yoh Sugawara, Atsunori Sugita, Satoshi Suzuki, Yuji Suzuki, Tomohiro Suhara, Kenji Sonota, Shuhei Takauji, Kohei Takashima, Sho Takahashi, Yoko Takahashi, Jun Takeshita, Yuuki Tanaka, Akihito Tampo, Taichiro Tsunoyama, Kenichi Tetsuhara, Kentaro Tokunaga, Yoshihiro Tomioka, Kentaro Tomita, Naoki Tominaga, Mitsunobu Toyosaki, Yukitoshi Toyoda, Hiromichi Naito, Isao Nagata, Tadashi Nagato, Yoshimi Nakamura, Yuki Nakamori, Isao Nahara, Hiromu Naraba, Chihiro Narita, Norihiro Nishioka, Tomoya Nishimura, Kei Nishiyama, Tomohisa Nomura, Taiki Haga, Yoshihiro Hagiwara, Katsuhiko Hashimoto, Takeshi Hatachi, Toshiaki Hamasaki, Takuya Hayashi, Minoru Hayashi, Atsuki Hayamizu, Go Haraguchi, Yohei Hirano, Ryo Fujii, Motoki Fujita, Naoyuki Fujimura, Hiraku Funakoshi, Masahito Horiguchi, Jun Maki, Naohisa Masunaga, Yosuke Matsumura, Takuya Mayumi, Keisuke Minami, Yuya Miyazaki, Kazuyuki Miyamoto, Teppei Murata, Machi Yanai, Takao Yano, Kohei Yamada, Naoki Yamada, Tomonori Yamamoto, Shodai Yoshihiro, Hiroshi Tanaka, Osamu Nishida

**Affiliations:** 1 Department of Surgery Related, Division of Anesthesiology, Kobe University Graduate School of Medicine, Kusunoki-cho 7-5-2, Chuo-ku, Kobe, Hyogo Japan; 2 Department of Traumatology and Acute Critical Medicine, Osaka University Medical School, Yamadaoka 2-15, Suita, Osaka, Japan; 3 Department of Anesthesiology and Critical Care Medicine, Fujita Health University School of Medicine, Toyoake, Japan; 4 Department of Intensive Care Unit, Nara Prefectural General Medical Center, Nara, Japan; 5 Department of Disaster and Emergency Medicine, Kobe University Graduate School of Medicine, Kobe, Japan; 6 Department of Emergency and Disaster Medicine, Juntendo University, Tokyo, Japan; 7 Department of Emergency and Intensive Care Medicine, Kagoshima University Graduate School of Medical and Dental Sciences, Kagoshima, Japan; 8 Department of Pediatric Critical Care, Shizuoka Children’s Hospital, Shizuoka, Japan; 9 Division of Emergency and Critical Care Medicine, Tohoku University Graduate School of Medicine, Sendai, Japan; 10 Department of Emergency, Disaster, and Critical Care Medicine, Faculty of Medicine, Kagawa University, Kagawa, Japan; 11 Department of Surgery Related, Division of Disaster and Emergency Medicine, Kobe University Graduate School of Medicine, Kobe, Japan; 12 Department of Emergency and Critical Care Medicine, Graduate School of Biomedical and Health Sciences, Hiroshima University, Hiroshima, Japan; 13 Department of Anesthesiology and Intensive Care Medicine, Kanazawa University, Kanazawa, Japan; 14 Acute and General Medicine, Yamaguchi University Graduate School of Medicine, Ube, Japan; 15 Department of Acute Medicine, The University of Tokyo, Tokyo, Japan; 16 Department of Anesthesiology and Intensive Care Medicine, Hamamatsu University School of Medicine, Hamamatsu, Japan; 17 Department of Emergency and Critical Care Medicine, Chiba University Graduate School of Medicine, Chiba, Japan; 18 Department of Emergency and Critical Care Medicine, Yamagata University Hospital, Yamagata, Japan; 19 Center for General Medicine Education, Keio University School of Medicine, Tokyo, Japan; 20 Department of Infectious Diseases, Kameda Medical Center, Kamogawa, Japan; 21 Department of Intensive Care Medicine, Sapporo Medical University School of Medicine, Sapporo, Japan; 22 Department of Advancing Acute Medicine, Graduate School of Medical Sciences, Nagoya City University, Nagoya, Japan; 23 Department of Emergency and Critical Care Medicine, Nagoya University Graduate School of Medicine, Nagoya, Japan; 24 Department of Emergency Medicine, Osaka Medical College, Osaka, Japan; 25 Department of Emergency and Intensive Care Medicine, JA Hiroshima General Hospital, Hatsukaichi, Japan; 26 Member of Japanese Association for Acute Medicine, Tokyo, Japan; 27 Division of Trauma and Surgical Critical Care, Osaka General Medical Center, Osaka, Japan; 28 Department of Nursing, Fujita Health University Hospital, Toyoake, Japan; 29 Department of Emergency and Critical Care Medicine, Juntendo University Urayasu Hospital, Urayasu, Japan; 30 Department of Emergency and Critical Care Medicine, St. Marianna University School of Medicine, Yokohama City Seibu Hospital, Yokohama, Japan; 31 Division of Rehabilitation, Department of Clinical Support and Practice, Hiroshima University Hospital, Hiroshima, Japan; 32 Department of Anesthesia, Kyoto University Hospital, Kyoto, Japan; 33 Department of Psychiatry, School of Medicine, National Defense Medical College, Tokorozawa, Japan; 34 Curtin University, Perth, Australia; 35 Department of Emergency and Critical Care Medicine/Infectious Disease, Hitachi General Hospital, Hitachi, Japan; 36 The Feinstein Institute for Medical Research, Manhasset, NY USA; 37 Department of Emergency and Critical Care Medicine, St. Luke’s International Hospital, Tokyo, Japan; 38 Emergency and Critical Care Medical Center, Osaka Police Hospital, Osaka, Japan; 39 Department of Emergency and Critical Care Medicine, Graduate School of Medicine, University of the Ryukyus, Okinawa, Japan; 40 Intensive Care Unit, Jikei University Hospital, Tokyo, Japan; 41 The Royal Children’s Hospital Melbourne, Melbourne, Australia; 42 Department of Emergency and Critical Care Medicine, Jichi Medical University Saitama Medical Center, Saitama, Japan; 43 Department of Emergency and Critical Care Medicine, Tsukuba Memorial Hospital, Tsukuba, Japan; 44 Division of Anesthesiology, Division of Intensive Care, Division of Emergency and Critical Care, Sendai City Hospital, Sendai, Japan; 45 Department of Physical Therapy, School of Health Sciences, Toyohashi Sozo University, Toyohashi, Japan; 46 Critical Care Medicine, National Center for Child Health and Development, Tokyo, Japan; 47 Department of General Pediatrics, Aichi Children’s Health and Medical Center, Obu, Japan; 48 Department of Infectious Disease, Hyogo Prefectural Amagasaki General Medical Center, Amagasaki, Japan; 49 Department of Intensive Care Medicine, Osaka Women’s and Children’s Hospital, Izumi, Japan; 50 Human Health Science, Graduate School of Medicine, Kyoto University, Kyoto, Japan; 51 Department of Acute and Critical Care Nursing, School of Nursing, Sapporo City University, Sapporo, Japan; 52 Department of Pharmacoepidemiology, Kyoto University Graduate School of Medicine and Public Health, Kyoto, Japan; 53 College of Nursing, Ibaraki Christian University, Hitachi, Japan; 54 Department of Emergency and Critical Care Medicine, Komaki City Hospital, Komaki, Japan; 55 Gastroenterological Center, Shinkuki General Hospital, Kuki, Japan; 56 Department of Surgery, Okinawa Chubu Hospital, Uruma, Japan; 57 CHU Sainte Justines, University of Montreal, Montreal, Canada; 58 Department of Rehabilitation, Showa General Hospital, Tokyo, Japan; 59 Department of Emergency Medicine and Department of Infectious Diseases, Japanese Red Cross Wakayama Medical Center, Wakayama, Japan; 60 Department of Anesthesiology and Intensive Care Medicine, International University of Health and Welfare School of Medicine, Narita, Japan; 61 Department of Anesthesiology, Tokai University School of Medicine, Isehara, Japan; 62 Department of Anesthesiology, Tokyo Medical University, Tokyo, Japan; 63 Division of Intensive Care, Nagasaki University Hospital, Nagasaki, Japan; 64 Center for Innovative Research for Communities and Clinical Excellence (CiRC2LE), Fukushima Medical University, Fukushima, Japan; 65 Department of Cardiology, Steel Memorial Muroran Hospital, Muroran, Japan; 66 Department of Emergency and Critical Care Medicine, Nippon Medical School Musashi Kosugi Hospital, Kawasaki, Japan; 67 Nagasaki University Hospital Acute and Critical Care Center, Nagasaki, Japan; 68 Division of Critical Care Medicine, Saitama Children’s Medical Center, Saitama, Japan; 69 Department of Emergency and Critical Care Medicine, National Hospital Organization Mito Medical Center, Ibaraki, Japan; 70 Department of Emergency and Critical Care Medicine, St. Marianna University School of Medicine, Kawasaki, Japan; 71 Department of Intensive Care Medicine, Kobe University Hospital, Kobe, Japan; 72 School of Medicine, Tokai University, Isehara, Japan; 73 Department of Emergency and Critical Care Medicine, Hitachi General Hospital, Hitachi, Japan; 74 Department of Traumatology and Critical Care Medicine, Osaka City University Graduate School of Medicine, Osaka, Japan; 75 Department of Anesthesiology and Intensive Care Medicine, Division of Intensive Care, Jichi Medical University School of Medicine, Shimotsuke, Japan; 76 Department of Emergency and Critical Care Medicine, Tokyo Bay Urayasu Ichikawa Medical Center, Urayasu, Japan; 77 Department of Anesthesiology and Intensive Care Medicine, Kyoto Prefectural University of Medicine, Kyoto, Japan; 78 Department of Emergency and Critical Care Medicine, National Hospital Organization Tokyo Medical Center, Tokyo, Japan; 79 Department of Pharmacy, Yokohama Rosai Hospital, Yokohama, Japan; 80 Department of Anesthesiology and Nutrition Support Team, Kobe City Medical Center General Hospital, Kobe City Hospital Organization, Kobe, Japan; 81 Department of Anesthesiology, Kobe University Hospital, Kobe, Japan; 82 Department of Rehabilitation, University of Tsukuba Hospital/Exult Co., Ltd., Tsukuba, Japan; 83 Doctoral program in Clinical Sciences. Graduate School of Comprehensive Human Sciences, University of Tsukuba, Tsukuba, Japan; 84 Department of Emergency Medicine, Kyoto Prefectural University of Medicine, Kyoto, Japan; 85 Department of Clinical Engineering, Osaka University Hospital, Suita, Japan; 86 Department of Intensive Care Medicine, Showa University School of Medicine, Tokyo, Japan; 87 Department of Clinical Engineering, Kakogawa Central City Hospital, Kakogawa, Japan; 88 Division of Respiratory Care and Rapid Response System, Intensive Care Center, Kitasato University Hospital, Sagamihara, Japan; 89 Department of Nursing, Toho University Omori Medical Center, Tokyo, Japan; 90 Department of Primary Care and Emergency Medicine, Kyoto University Hospital, Kyoto, Japan; 91 Department of Emergency and Critical Care Medicine, Keio University School of Medicine, Tokyo, Japan; 92 Department of Anesthesiology and Intensive Care Medicine, Osaka University Graduate School of Medicine, Suita, Japan; 93 Nursing Department, Osaka General Medical Center, Osaka, Japan; 94 Toho University Omori Medical Center, Tokyo, Japan; 95 Department of Clinical Anesthesiology, Mie University Hospital, Tsu, Japan; 96 Department of Anesthesiology and Critical Care Medicine, Division of Acute and Critical Care Medicine, Hokkaido University Faculty of Medicine, Sapporo, Japan; 97 Department of Emergency and Critical Care Medicine, Eastern Chiba Medical Center, Togane, Japan; 98 Department of Emergency Medicine, Gunma University Graduate School of Medicine, Maebashi, Japan; 99 Department of Emergency and Critical Care Medicine, Nara Medical University, Kashihara, Japan; 100 Department of Anesthesiology and Intensive Care, Oita University Hospital, Yufu, Japan; 101 Department of Emergency and Critical Care Medicine, Nippon Medical School Hospital, Tokyo, Japan; 102 Department of Anesthesiology and Intensive Care Medicine, Graduate School of Medicine, Osaka University, Suita, Japan; 103 Department of Anesthesiology, Emergency and Critical Care Medicine, Kure Kyosai Hospital, Kure, Japan; 104 Department of General Internal Medicine, Soka Municipal Hospital, Soka, Japan; 105 Department of Emergency and Critical Care Medicine, Tokyo Metropolitan Children’s Medical Center, Tokyo, Japan; 106 Department of Biomedical Ethics, Graduate School of Medicine, The University of Tokyo, Tokyo, Japan; 107 Department of Infectious Diseases, Rakuwakai Otowa Hospital, Kyoto, Japan; 108 Department of Health Informatics, School of Public Health, Kyoto University, Kyoto, Japan; 109 Tohoku University Hospital Emergency Center, Sendai, Japan; 110 Department of Anesthesiology, National Hospital Organization Iwakuni Clinical Center, Iwakuni, Japan; 111 Advanced Medical Emergency Department and Critical Care Center, Japan Red Cross Maebashi Hospital, Maebashi, Japan; 112 Department of Anesthesiology, Kansai Medical University, Hirakata, Japan; 113 Advanced Emergency and Critical Care Center, Saitama Red Cross Hospital, Saitama, Japan; 114 Department of Emergency and Critical Care Medicine, University of Tsukuba, Tsukuba, Japan; 115 Department of Emergency and Critical Medicine, Showa University Fujigaoka Hospital, Yokohama, Japan; 116 Department of Clinical Epidemiology and Health Economics, School of Public Health, The University of Tokyo, Tokyo, Japan; 117 Shimane Advanced Trauma Center, Izumo, Japan; 118 Department of Primary care and Emergency medicine, Kyoto University Graduate School of Medicine, Kyoto, Japan; 119 Department of Anesthesiology, Kyorin University School of Medicine, Tokyo, Japan; 120 Department of ER, Hashimoto Municipal Hospital, Hashimoto, Japan; 121 Department of Community Medical Supports, Tohoku Medical Megabank Organization, Tohoku University, Sendai, Japan; 122 Tochigi prefectural Emergency and Critical Care Center, Imperial Gift Foundation Saiseikai, Utsunomiya Hospital, Utsunomiya, Japan; 123 Department of Anesthesiology, Faculty of Medicine, Yamagata University, Yamagata, Japan; 124 Department of Internal Medicine, Dialysis Center, Kichijoji Asahi Hospital, Tokyo, Japan; 125 Anesthesiology, Emergency Medicine, and Intensive Care Division, Inazawa Municipal Hospital, Inazawa, Japan; 126 Department of Anesthesiology and Intensive Care Medicine, Nagoya-City University Graduate School of Medical Sciences, Nagoya, Japan; 127 Department of Anesthesiology, Sendai Medical Center, Sendai, Japan; 128 Emergency and Critical Care Center, Mie University Hospital, Tsu, Japan; 129 Department of Emergency Medicine, Fukui Prefectural Hospital, Fukui, Japan; 130 Department of Gastroenterological Surgery, Onga Hospital, Fukuoka, Japan; 131 Department of Emergency and Critical Care Medicine, Seirei Mikatahara General Hospital, Hamamatsu, Japan; 132 Department of Pediatrics, University of Tsukuba Hospital, Tsukuba, Japan; 133 Center for Translational Injury Research, University of Texas Health Science Center at Houston, Houston, USA; 134 Department of Anesthesiology, Tokyo Medical University, Tokyo, Japan; 135 Department of General Medicine Shintakeo Hospital, Takeo, Japan; 136 Department of Emergency and Critical Care Medicine, University of Tsukuba Hospital, Tsukuba, Japan; 137 Department of Orthopaedic Surgery, Center for Advanced Joint Function and Reconstructive Spine Surgery, Graduate school of Medicine, Chiba University, Chiba, Japan; 138 Department of Pediatric Cardiology and Intensive Care, Gunma Children’s Medical Center, Shibukawa, Japan; 139 Department of Emergency and Critical Care Medicine, Tokyo Women’s Medical University Medical Center East, Tokyo, Japan; 140 Department of Anesthesiology, Yokohama City University, Yokohama, Japan; 141 Department of Acute Medicine, Division of Emergency and Critical Care Medicine, Nihon University School of Medicine, Tokyo, Japan; 142 Department of Intensive Care, Okayama University Hospital, Okayama, Japan; 143 Department of Anesthesiology, Keio University School of Medicine, Tokyo, Japan; 144 Department of Intensive Care Medicine, Miyagi Children’s Hospital, Sendai, Japan; 145 Department of Emergency Medicine, Asahikawa Medical University, Asahikawa, Japan; 146 Department of Cardiology, Fukuyama City Hospital, Fukuyama, Japan; 147 Department of General Internal Medicine, Koga General Hospital, Koga, Japan; 148 Department of Anesthesiology, Osaka Women’s and Children’s Hospital, Izumi, Japan; 149 Fukuoka Prefectural Psychiatric Center, Dazaifu Hospital, Dazaifu, Japan; 150 Department of Emergency Medicine, Teikyo University School of Medicine, Tokyo, Japan; 151 Emergency and Critical Care Center, Kyushu University Hospital, Fukuoka, Japan; 152 Department of Intensive Care Medicine, Kumamoto University Hospital, Kumamoto, Japan; 153 Department of Anesthesiology and Intensive Care Unit, Todachuo General Hospital, Toda, Japan; 154 Department of Pediatrics, Keio University School of Medicine, Tokyo, Japan; 155 Department of Emergency and Critical Care Medicine, Saiseikai Yokohamashi Tobu Hospital, Yokohama, Japan; 156 Department of Emergency, Critical Care, and Disaster Medicine, Okayama University Graduate School of Medicine, Dentistry and Pharmaceutical Sciences, Okayama, Japan; 157 Intensive Care Unit, Yokohama City Minato Red Cross Hospital, Yokohama, Japan; 158 Department of Respiratory Medicine, Tokyo Yamate Medical Center, Tokyo, Japan; 159 Department of Emergency and Critical Care Medicine, Japanese Red Cross Kyoto Daini Hospital, Kyoto, Japan; 160 Department of Anesthesiology and Critical Care Medicine, Nagoya Daini Red Cross Hospital, Nagoya, Japan; 161 Department of Emergency Medicine and Intensive Care Medicine, Shizuoka General Hospital, Shizuoka, Japan; 162 Department of Preventive Services, Kyoto University Graduate School of Medicine, Kyoto, Japan; 163 Division of Emergency and Critical Care Medicine Niigata University Graduate School of Medical and Dental Science, Niigata, Japan; 164 Department of Emergency and Critical Care Medicine, Juntendo University Nerima Hospital, Tokyo, Japan; 165 Department of Pediatric Critical Care Medicine, Osaka City General Hospital, Osaka, Japan; 166 Department of Emergency and Critical Care Medicine, Saiseikai Utsunomiya Hospital, Utsunomiya, Japan; 167 Research Associate of Minimally Invasive Surgical and Medical Oncology, Fukushima Medical University, Fukushima, Japan; 168 Department of Emergency Medicine, Japanese Red Cross Society Wakayama Medical Center, Wakayama, Japan; 169 Department of Emergency Medicine, Saitama Saiseikai Kurihashi Hospital, Kuki, Japan; 170 Division of Intensive Care Unit, Sakakibara Heart Institute, Tokyo, Japan; 171 Department of Emergency Medicine and Critical Care Medicine, Tochigi Prefectural Emergency and Critical Care Center, Imperial Foundation Saiseikai Utsunomiya Hospital, Utsunomiya, Japan; 172 Department of Anesthesiology, St. Mary’s Hospital, Our Lady of the Snow Social Medical Corporation, Kurume, Japan; 173 Department of Emergency and Critical Care Medicine, Japanese Red Cross Kyoto Daiichi Hospital, Kyoto, Japan; 174 Department of Critical Care Medicine, Kyushu University Hospital, Fukuoka, Japan; 175 Department of Healthcare Epidemiology, School of Public Health in the Graduate School of Medicine, Kyoto University, Kyoto, Japan; 176 Department of Intensive Care, Chiba Emergency Medical Center, Chiba, Japan; 177 Department of Internal Medicine, Kanazawa Municipal Hospital, Kanazawa, Japan; 178 Ishikawa Prefectual Central Hospital Emergency and Critical Care Center, Kanazawa, Japan; 179 Department of Emergency and General Internal Medicine, Saiseikai Kawaguchi General Hospital, Kawaguchi, Japan; 180 Department of Emergency and Disaster Medicine, Showa University, Tokyo, Japan; 181 Department of Cardiology, Tokyo Metropolitan Geriatric Hospital and Institute of Gerontology, Tokyo, Japan; 182 Department of Emergency Medicine, Kobe City Medical Center General Hospital, Kobe, Japan; 183 Department of Critical Care and Emergency Medicine, Miyazaki Prefectural Nobeoka Hospital, Nobeoka, Japan; 184 Department of Traumatology and Critical Care Medicine, National Defense Medical College, Tokorozawa, Japan; 185 Department of Emergency Medicine, University of Fukui Hospital, Fukui, Japan; 186 Pharmaceutical Department, JA Hiroshima General Hospital, Hatsukaichi, Japan

**Keywords:** Evidence-based medicine, GRADE, Guidelines, Sepsis, Septic shock, Systematic review

## Abstract

The Japanese Clinical Practice Guidelines for Management of Sepsis and Septic Shock 2020 (J-SSCG 2020), a Japanese-specific set of clinical practice guidelines for sepsis and septic shock created as revised from J-SSCG 2016 jointly by the Japanese Society of Intensive Care Medicine and the Japanese Association for Acute Medicine, was first released in September 2020 and published in February 2021. An English-language version of these guidelines was created based on the contents of the original Japanese-language version. The purpose of this guideline is to assist medical staff in making appropriate decisions to improve the prognosis of patients undergoing treatment for sepsis and septic shock. We aimed to provide high-quality guidelines that are easy to use and understand for specialists, general clinicians, and multidisciplinary medical professionals. J-SSCG 2016 took up new subjects that were not present in SSCG 2016 (e.g., ICU-acquired weakness [ICU-AW], post-intensive care syndrome [PICS], and body temperature management). The J-SSCG 2020 covered a total of 22 areas with four additional new areas (patient- and family-centered care, sepsis treatment system, neuro-intensive treatment, and stress ulcers). A total of 118 important clinical issues (clinical questions, CQs) were extracted regardless of the presence or absence of evidence. These CQs also include those that have been given particular focus within Japan. This is a large-scale guideline covering multiple fields; thus, in addition to the 25 committee members, we had the participation and support of a total of 226 members who are professionals (physicians, nurses, physiotherapists, clinical engineers, and pharmacists) and medical workers with a history of sepsis or critical illness. The GRADE method was adopted for making recommendations, and the modified Delphi method was used to determine recommendations by voting from all committee members.

As a result, 79 GRADE-based recommendations, 5 Good Practice Statements (GPS), 18 expert consensuses, 27 answers to background questions (BQs), and summaries of definitions and diagnosis of sepsis were created as responses to 118 CQs. We also incorporated visual information for each CQ according to the time course of treatment, and we will also distribute this as an app. The J-SSCG 2020 is expected to be widely used as a useful bedside guideline in the field of sepsis treatment both in Japan and overseas involving multiple disciplines.

## Introduction

Approximately 50 million people worldwide die from sepsis each year. Sepsis is a serious illness that affects all age groups, and the social significance of the creation of a high-quality guideline with the objective of providing medical support for this illness is high. The Surviving Sepsis Campaign Guideline (SSCG) [1, 2] has been revised as an international sepsis clinical practice guideline every 4 years since 2004. In 2012, the Japanese version of the Surviving Sepsis Campaign Guideline (J-SSCG), which considered the actual circumstances of Japanese clinical settings, was first published by the Japanese Society of Intensive Care Medicine (JSICM) [3, 4]. At the time of the 2016 revision (J-SSCG 2016), JSICM and the Japanese Association for Acute Medicine (JAAM) worked together to create a high-quality guideline that is easy to understand even for general clinicians, aiming for widespread dissemination. J-SSCG 2016 actively took up new domains not covered in SSCG 2016, such as imaging diagnosis, body temperature regulation, ICU-acquired weakness (ICU-AW), and post-intensive care syndrome (PICS), providing medical guidelines.

In this current revision (J-SSCG 2020), the two societies have once again cooperated with one another with the aim of providing support not only to specialists and general clinicians but also multidisciplinary medical professionals to make appropriate decisions to improve the prognosis of patients with sepsis. In addition to the 26 committee members and directors in charge selected from both societies, we received the participation and support of a total of 226 individuals, comprising 85 working group members that included multiple professions (nine nurses, four physiotherapists, two clinical engineers, and two pharmacists) and those with a history of sepsis or critical illness (two, one of which was a nurse) and 115 systematic review members. The participation of multiple professions and experienced patients as working group members in particular expanded the perspective of our work and enabled a more flexible evaluation, which was a great step forward from the J-SSCG 2016. Furthermore, systematic reviews were conducted by the working group members and systematic review members, and there was a certain degree of independence from the committee members who formulated the recommendations.

Four new topics were incorporated in the J-SSCG 2020 in addition to the domains in the previously mentioned J-SSCG 2016: neuro-intensive care, patient- and family-centered care, sepsis treatment system, and stress ulcers. The J-SSCG 2020 also included a section on children after considering the fact that there are few pediatric intensive care units in Japan, and the situation is such that medical professionals who primarily treat adult sepsis patients must treat pediatric sepsis patients. With these additions, this guideline comprised a total of 22 topics and 118 CQs. The GRADE system was incorporated to prepare the recommendations, and the modified Delphi method was used to decide recommendations by voting from all committee members. Responses to the CQs were as follows: 79 GRADE-based recommendations, 5 Good Practice Statements (GPS), 18 expert consensuses, 27 answers to background questions (BQs), and definition and diagnosis of sepsis. We will also incorporate visual information for each CQ according to time axes such as medical care flow charts as a new attempt. Each CQ will be clinically positioned, and we will also distribute this as an app.

The J-SSCG 2020 original Japanese version was first released in the official society websites of the JSICM and JAAM in September 2020, followed by the publication in their official journals *the Journal of JSICM* [2021; Volume 28 (Supplement)] 10.3918/jsicm.27S0001 and *Journal of Japanese Association for Acute Medicine* [2021; Volume 32, S1] 10.1002/jja2.S0024 in February 2021. It was then translated into English and released on the societies’ websites in April, in advance of the simultaneous publication in their English-language official journals *Journal of Intensive Care* and *Acute Medicine and Surgery*.

## Overview and basic principles of these guidelines

### Name

The English name of this guideline is the Japanese Clinical Practice Guidelines for Management of Sepsis and Septic Shock 2020, and the abbreviation used was J-SSCG 2020 in consideration of the comparison made with the international version (SSCG).

### Overall objective of this guideline

The objective of this guideline is to provide support for medical professionals to make appropriate decisions in order to improve the prognosis of patients in the clinical treatment of sepsis and septic shock.

### Target patient populations

This guideline targets patients with or who are suspected of sepsis or septic shock, ranging from children to adults. This includes patients who receive diagnoses and treatment not only in the intensive care unit but also in the general ward and emergency outpatient departments. However, sepsis patients require advanced systemic management, so we emphasize that it is desirable for those with or who are strongly suspected of sepsis to be promptly transferred to intensive care units as circumstances allow and undergo management there.

### Target users (users of this guideline)

All medical professionals such as specialists, general clinicians, nurses, pharmacists, physiotherapists, clinical engineers, and registered dietitians who are engaged in or involved in sepsis treatment.

### Participation of representatives of associated expert groups and support for guideline creation experts

In addition to the 26 committee members and directors in charge selected from the Japanese Society of Intensive Care Medicine and the Japanese Association for Acute Medicine, the J-SSCG 2020 received the participation and support of a total of 226 individuals, comprising 85 working group members that included multiple professionals (nine nurses, four physiotherapists, two clinical engineers, and two pharmacists) and those who had an experience of sepsis or critical illness (two; one of which was a nurse) and 115 systematic review members.

As guideline creation experts, these individuals reviewed and confirmed the work process at each stage of the guideline creation process under the guidance of the EBM Medical Information Department of the Japan Council for Quality Health Care and in accordance with the principles of the GRADE system. Specialists from the EBM Medical Information Department participated in committee meetings and responded to questions from the guideline creation managers in order to directly solve problems.

### Methods to reflect the values of the target populations (e.g., patients, general public)

Two medical professionals and researchers who had sepsis were added as committee members or working group members in order to reflect the values and hopes of patients and patient families. This point was considered useful in reflecting values and hopes from the position of patients and families after understanding the complexity, severity, and pathology of sepsis, which requires wide-ranging and advanced medical knowledge.

### Peer review and public comments

Transparency during the creation of the J-SSCG 2020 was considered to be crucial. Official mailing lists (ML) were created for discussions among members of each team. Core members joined the MLs established by each team as read-only members. Through these measures, we aimed to increase the transparency of team discussions, and by implementing the appropriate interventions, we were able to coordinate the directions taken by each team and achieve consistency throughout the entirety of the guidelines. Mutual peer review was conducted for various work processes by external team members across the region. Work products from each group were repeatedly edited and revised, and each revised draft was discussed by the Guideline Creation Committee.

The initial draft of the CQs received public comments over the Internet. Answer for each CQ also had public comments. Public commenters were requested to disclose any conflicts of interest.

### Disclosure of conflicts of interest (COIs) and members’ roles

Financial and academic COIs as well as the role(s) of each committee member are disclosed in the Additional file 1 (https://www.jsicm.org/pdf/guidelineEN/Additionalfile1.pdf). Financial COIs were disclosed in accordance with the standards used by the Japanese Association of Medical Sciences from 2017 through 2019.

### Funding

These guidelines were prepared with financial support from the Japan Society of Intensive Care Medicine and the Japanese Association for Acute Medicine. No member of the Guideline Creation Committee received any form of financial compensation during the preparation of these guidelines. The views and interests of these societies were not reflected in the preparation of the guidelines’ recommendations.

### Guideline dissemination strategy

The Japanese version of these guidelines is open access. To promote ease of use, the digest version of the guidelines booklet is available. In addition, the app version of the guideline is available for use to support the clinical setting. We will strive to make these guidelines available at various academic meetings and seminars and also monitor activities related to sepsis practice as well as the spread of these guidelines throughout the target medical community.

### Planned revisions

These guidelines are scheduled to undergo revision every 4 years. The next revision will occur in 2024. Should important new information warranting revision be obtained beforehand, partial revision will be considered.

## Methods used for creating this guideline

The J-SSCG 2020 was created through the three following processes: 1) planning a clinical question (CQ); 2) searching, collecting, and integrating evidence through a systematic review and evaluating its certainty; and 3) formulating a recommendation. Relevant information for a recommendation based on GRADE and expert consensus were available at https://www.jsicm.org/pdf/J-SSCG2020_supplementary_appendix01.pdf.

### Planning a CQ

Clinical practice guidelines should cover the basic knowledge of clinical practice and contribute to the construction of a standard clinical practice system. For this reason, important CQs were extracted from each domain regardless of presence or absence of evidences, and important CQs taken up in previous guidelines were adopted in this guideline. Based on the rules of planning a CQ, committee members and working group members collaborated to create a draft CQ in their area of responsibility, an opinion extracted from mutual peer review by committee members was reflected, and a CQ list was created by the Guideline Creation Committee. Public comments were solicited online for these CQs. The CQs were then revised using these public comments received, and a total of 118 CQs were ultimately decided by the committee.

### CQ classifications

CQs include background questions (BQs) and foreground questions. BQs indicate CQs that inquire about what is well known as general knowledge, such as diseases, diagnoses, and treatment. Meanwhile, foreground questions are CQs that inquire about information specialized to various situations in clinical settings and can influence decision-making in clinical practice (Table 1).

**Table 1:** CQ classifications

CQ classifications	
Background questions (BQ)	CQs which inquire about what is general knowledge, such as diseases, diagnoses, and treatment
	Standard knowledge is presented.
	Systematic review is not needed.
	No recommendations are given.
Foreground questions (FQ)	CQs which inquire about information specialized to various situations in clinical settings. For example, whether a particular treatment is effective for a patient with a specific illness. This can influence decisions in clinical settings.
	Treatment options are presented. Systematic review is required for FQs other than GPS.
	Recommendations on treatment selection are given.
Recommendation classifications for FQs	
Good practice statement (GPS)	Recommendations on topics that are so common that they cannot become a research theme and of which all medical personnel should be made aware
GRADE-based recommendation (GRADE)	Recommendations presented in accordance with the principles of the GRADE system. A systematic review is conducted, four factors (certainty of evidence, balance of benefits and harms, values and preferences, costs and resource utilization) based on the obtained evidence are taken into consideration, and recommendations are made in consultation with the committee.
Expert consensus-based recommendation (unGRADE)	Consensus made by experts for CQs for which a systematic review was conducted but had no target articles. Three factors (balance of expected benefits and harms, values and preferences, costs and resource utilization) are taken into consideration and recommendations are made in consultation with the committee.

### Formulating answers to BQs

BQs aim to present information that summarizes general knowledge such as illnesses, diagnoses, and treatment. Each area group prepared draft recommendations for the CQs, which were amended and revised repeatedly until the approval rate in the committee exceeded 95% for consensus.

### Formulating answers to foreground questions

Foreground questions include (1) GPS, which are CQs that are extremely common and of which all medical personnel should be aware, and (2) CQs that are subject to systematic review and for which recommendations are formulated. The latter CQ was given a recommendation based on GRADE or on expert consensus depending on whether target articles were present or absent, respectively.

#### Formulating GPS

GPS was displayed for CQs, which handled themes that were extremely common and for which randomized controlled trials were theoretically impossible. These were amended and revised repeatedly until the approval rate in the committee exceeded 95% for consensus.

#### Searching, collecting, and integrating evidence through systematic review

A comprehensive literature review was conducted for each CQ in the foreground questions except for GPS, from which randomized controlled trials (RCTs) were extracted. As a general rule, the methodology was based on the Grading of Recommendations Assessment, Development and Evaluation (GRADE).

- Step 1: Literature review

Literature reviews were conducted using the search engines of CENTRAL, PubMed, and Ichushi-Web.

The search equations were created by two or more independent reviewers using Medical Subject Headings (MeSH) terms and free search terms. Searches on PubMed used the sensitive-maximizing version of search strategies created by Cochrane as a general ruler for research design filters that specified RCTs. The publication date of the subject articles was not restricted. The languages of the manuscript were limited to Japanese and English. After confirming that the key RCTs specified in advance were included, the literature review equations underwent a final decision, and the literature review date and number of articles found in each search engine were recorded.

- Step 2: Primary screening

All the titles and abstracts specified in Step 1 were downloaded. The automatic duplicate deletion function of the literature management software EndNote (Clarivate Analytics, USA) or Mendeley (Mendeley Ltd., UK) were used to remove duplicates, with duplicate articles further deleted manually. Article screening was conducted online using Rayyan (https://rayyan.qcri.org/welcome). Two independent reviewers reviewed the titles and abstracts of the literature and excluded research methods and PICO criteria, which were clearly not within the target. If there was any possibility that it was a target article, it was not excluded.

- Step 3: Secondary screening

The full text of the remaining articles from Step 2 were ordered, and two reviewers selected articles whose research design and PICO criteria conformed to the CQ, and they confirmed them as target articles. Articles for which the opinions of the two reviewers did not match were sent to a third reviewer and discussed among the three reviewers. Articles excluded at this stage were provided a reason for exclusion. The process from literature review to target article selection is summarized in the PRISMA flow diagram.

- Step 4: Evaluation of the certainty of evidence for CQs where evidence existed

Risk evaluations were conducted for the certainty of evidence (A-D) of the CQ undergoing systematic review for which each group was responsible. The definitions for the certainty of evidence as set by the GRADE system adopted in this guideline are as follows.

Definition of the certainty of evidence

- High: Highly confident in the estimated value of effects
- Medium: Moderate confidence in the estimated value of effects
- Low: Limited confidence in the estimated value of effects
- Very low: Almost no confidence in the estimated value of effects

- Step 5: Data extraction, bias risk evaluation

Data extraction was performed by two independent reviewers, and a standardized data extraction form was used. In cases where insufficient information was recorded in the reference, this was stated as such, and the authors were not contacted.

- Step 6: Meta-analysis and evaluation of the certainty of evidence

Qualitative and quantitative evaluations of the references to be adopted were performed. The qualitative evaluations used RevMan 5 whenever possible to conduct meta-analyses. This was summarized so that each area group could create evaluations of the certainty of evidence.

### Handling of CQs with network meta-analysis

Indirect and network estimate values were calculated using a frequency-based analysis method for CQs with network meta-analyses (Confidence in Network Meta-Analysis [CINeMA] from R package *netmeta* used). The surface under the cumulative ranking curve (SUCRA) was used for rankings (calculated as Stata mvmeta command). The quality of evidence was evaluated based on the GRADE working group methods (ref). Network meta-analyses were conducted on CQ9–2 and CQ9–6 of this guideline.

### Handling of CQs with qualitative research as evidence

The GRADE-Confidence in the Evidence from Reviews of Qualitative research (CERQual) approach was adopted as an evidence extraction method for CQs, where qualitative research was thought to be an appropriate research method. This was used in CQ20–3, “Should physical binding (restraints) be avoid during intensive care?”, in this guideline.

#### Formulation of proposed recommendations

The committee members and working group collaborated to create an evidence to decision (EtD) table in advance of deciding the recommendations. They then considered four factors (certainty of evidence, balance of effects, values, and cost/resource utilization) and formulated recommendations in consultation with the committee. The strengths of the recommendations shown in the GRADE system are classified as recommended, suggested, not suggested, and not recommended.

=Description methods for the strength of recommendations=

Strength of recommendation “1”: recommended.

Strength of recommendation “2”: suggested.

Committee members and the working group collaborated to create an EtD table for foreground question type CQs, for which insufficient evidence was obtained through comprehensive literature reviews conforming to the PICO criteria and formed an expert consensus based on this EtD. Recommendations in this EtD took into consideration the expert-proposed factors of the balance between the desired and undesired effects of each intervention, values, and costs/resource utilization, conducted in consultation with the committee. Recommendations with these expert consensuses were “suggestions”, and “(expert consensus: insufficient evidence)” was added at the end of the text so that this could be distinguished from the above-mentioned recommendations based on GRADE.

#### Consensus building in CQs in accordance with GRADE and CQs showing expert consensus

The modified Delphi method was used for consensus building among committee members.

- Step 1: Voting

Each committee member anonymously voted online in an independent manner using a point system ranging from 1 to 9 (1: disagree, 9: agree). The median, interpercentile range (IPR), interpercentile range adjusted for symmetry (IPRAS), and disagreement index (DI) of the obtained scores were calculated.

- Step 2: Panel meeting

Panel meetings were conducted based on the aggregated results as shown below to reach a consensus.

1. When median < 7.5 and DI ≥0.2

Discussions were held within the committee, after which amendments were made to the EtD and recommended text, and a second vote was held.

2. When median ≥ 7.5 or DI < 0.2
  A When a serious opinion was present during voting for a comment or recommendation presented by committee member
    Discussions were held within the committee, and a consensus was reached. CQs for which a consensus was not reached within the committee resulted in amendments to the EtD and recommended text, after which a second vote was held.
  B When no serious opinions were present during voting for a comment or recommendation presented by a committee member.

The voting results were confirmed among the committee members, and a consensus was reached.

**Quick reference list of CQ&As**

**CQ1: Definition and diagnosis of sepsis**

**CQ1-1: Definition of sepsis**

***Summary:*** According to the Third International Consensus Definitions for Sepsis and Septic Shock (Sepsis-3), sepsis is defined as “life-threatening organ dysfunction caused by a dysregulated host response to infection.” Septic shock is defined as a subset of sepsis in which the underlying circulatory and cellular/metabolic abnormalities profoundly increase the risk of mortality.

**CQ1-2: Diagnosis of sepsis and septic shock**

***Summary:*** A diagnosis of sepsis is confirmed when the Sequential Organ Failure Assessment (SOFA) score of 2 points or more acutely increase in the presence of a clear infection or suspected infection. Patients with septic shock can be identified with a clinical construct of sepsis with persisting hypotension requiring vasopressors to maintain mBP ≥ 65 mmHg and having a serum lactate level > 2 mmol/L (18 mg/dL) despite adequate volume resuscitation. In out-of-hospital, emergency department, or general hospital ward settings, adult patients with suspected infection can be rapidly identified as more likely to have poor outcomes typical of sepsis if they have at least two of the following clinical criteria that together constitute the quick SOFA (qSOFA) score: a respiratory rate of 22 breaths/min or higher, altered consciousness, and a systolic blood pressure of ≤100 mmHg. The qSOFA criteria can be used to prompt clinicians to further investigate organ dysfunction, initiate or escalate therapy as appropriate, and to consider referral for critical care. Ultimately, an acutely increased SOFA score of 2 or more points confirms the diagnosis of sepsis. Daily routine screening for sepsis is recommended to support the early diagnosis and treatment of sepsis.

**CQ2: Diagnosis of infection**

**CQ2-1: When should a blood culture be taken?**

***Answer:*** Take two or more sets before administering the antibacterial drug (Good Practice Statement).

**CQ2-2: When should culture specimens other than blood be collected?**

***Answer:*** Each cultured specimen other than blood should be collected as needed prior to the administration of antibacterial drugs (Good Practice Statement).

**CQ2-3: Is Gram staining useful in the selection of antimicrobial agents before obtaining culture results?**

***Answer:*** We suggest referencing Gram staining findings of the culture specimen when selecting an antibacterial drug to use for empirical treatment (expert consensus: insufficient evidence).

**CQ2–4-1: What are the positions of C-reactive protein (CRP), procalcitonin (PCT), presepsin (P-SEP), and interleukin 6 (IL-6) as biomarker tests for sepsis diagnosis in general ward and emergency rooms (ER)?**

***Answer:*** Sensitivity and specificity in biomarker tests when sepsis was suspected in general ward and ER visits were as follows: CRP, 59, 79%; PCT, 74, 81%; P-SEP, 75, 74%; IL-6, 78, 78%. As such, sepsis diagnosis with biomarkers alone is generally thought to be difficult, and its use should be seen as supplemental to any observations of general conditions (Provision of information for background question).

**CQ2–4-2: What are the positions of C-reactive protein (CRP), procalcitonin (PCT), presepsin (P-SEP), and interleukin-6 (IL-6) as biomarker tests for sepsis diagnosis in the intensive care unit?**

***Answer:*** Sensitivity and specificity in biomarker tests when sepsis was suspected in the intensive care unit were as follows: CRP, 74, 70%; P-SEP, 82, 73%; IL-6, 72, 76%. As such, sepsis diagnosis with biomarkers alone is generally thought to be difficult, and its use should be supplemental to any observations of general conditions (Provision of information for background question).

**CQ3: Source control**

**CQ3-1: Should imaging tests be conducted in patients suspected of sepsis in order to search for the source of infection?**

***Answer:*** Imaging tests should be conducted when the source of infection is unclear in order to search for the source of infection (Good Practice Statement).

**CQ3-2: Should whole-body contrast-enhanced CT tests be conducted at an early stage for sepsis patients with unknown source of infection?**

***Answer:*** We suggest conducting whole-body contrast-enhanced CT tests as soon as possible for sepsis patients with unknown source of infection (expert consensus: insufficient evidence).

**CQ3-3: Should the source of infection be controlled by surgery/invasive drainage in patients with sepsis due to intraperitoneal infection?**

***Answer:*** We suggest controlling the source of infection as soon as possible with surgery/invasive drainage (including abscess drainage, biliary tract/gallbladder drainage) for patients with sepsis due to intraperitoneal infection (expert consensus: insufficient evidence).

**CQ3-4-1: Should the source of infection be controlled with invasive interventional therapy during the early period of infectious pancreatic necrosis?**

***Answer:*** We suggest against controlling the source of infection with invasive interventional therapy during the early period of infectious pancreatic necrosis (GRADE 2C: certainty of evidence = “low”).

**CQ3-4-2: Should the source of infection be controlled with low-invasive interventional therapy for infectious pancreatic necrosis?**

***Answer:*** We recommend controlling the source of infection with less invasive interventional therapy for patients with sepsis caused by infectious pancreatic necrosis (GRADE 2B: certainty of evidence = “moderate”).

**CQ3-5: Should the source of infection be controlled with invasive drainage for patients with sepsis due to acute pyelonephritis caused by ureteral obstruction?**

***Answer:*** We suggest controlling the source of infection as soon as possible with transurethral ureteral stent implantation or percutaneous nephrostomy in patients with sepsis due to acute pyelonephritis caused by ureteral obstruction (expert consensus: insufficient evidence).

**CQ3-6: Should source control be achieved by means of surgical debridement for sepsis patients due to necrotic soft tissue infection?**

***Answer:*** We suggest controlling the source of infection as soon as possible by means of surgical debridement for sepsis patients due to necrotic soft tissue infection (expert consensus: insufficient evidence).

**CQ3-7: Should the source of infection be controlled with catheter removal in patients with sepsis where catheter-related bloodstream infections are suspected?**

***Answer:*** We suggest controlling the source of infection as soon as possible with catheter removal in patients with sepsis where catheter-related bloodstream infections are suspected (expert consensus: insufficient evidence).

**CQ3-8: Should the source of infection be controlled through invasive drainage in patients with sepsis due to empyema?**

***Answer:*** We suggest controlling the source of infection as soon as possible with percutaneous thoracic drainage or surgical intervention in patients with sepsis due to empyema (expert consensus: insufficient evidence).

**CQ4: Antimicrobial therapy**

**CQ4-1: How should empirical antimicrobial therapy be selected?**

***Answer:*** Antimicrobials can be selected by estimating the causative microorganism based on suspected infectious foci, patient background, epidemiology and rapid microbial diagnostic tests, and by considering the tissue penetration properties of drugs and the probabilities of resistant bacteria (see Table 2 for reference). (Provision of information for background question).

**Table 2:** Empiric therapeutic agents for each infectious disease

Source of infection	Patient background / pathology		Expected causative bacteria	Drug examples (see note k) for VCM dose)	Remarks
**Pneumonia**^a)^	Community-acquired	Other than the reasons listed below	Pneumococcus, *Haemophilus influenzae*, *Klebsiella* spp., *Mycoplasma pneumoniae*, *Legionella pneumophila*	CTRX 2 g, every 24 h [5] ±AZM 500 mg, every 24 h [5]	See CQ4–3 for *Legionella* risk.
		After influenza, necrotizing pneumonia	Above + *Staphylococcus aureus* (including community-acquired MRSA)	CTRX 2 g, every 24 h [5, 6] ±VCM [5, 6], ^k^	See CQ4–3 for MRSA risk.
	Healthcare-associated/ ventilator-related		*Streptococcus pneumoniae*, *E. coli*, *Pseudomonas aeruginosa*, *Staphylococcus aureus*	“CFPM 2 g, every 8 h, or TAZ/PIPC 4.5 g, every 8 h” ±VCM [5],^k^	Option of community-acquired pneumonia is applicable at an early stage or when there is no risk of resistant bacteria. See CQ4–3 for MRSA risk.
	Decreased cell-mediated immunity + no prevention of *Pneumocystis jirovecii* + bilateral shadows		*Pneumocystis jirovecii*	ST trimethoprim 240–320 mg, every 8 h or pentamidine 4 mg/kg, every 24 h [5]	ST: trimethoprim 15 mg/kg/day ≒Japanese ST mixture (1 tablet or 1 g of trimethoprim is 80 mg) 3–4 tablets or 3–4 g, every 8 h.
**Urinary tract infection**^b)^	Community-acquired (low risk of ESBL-producing bacteria)		*E. coli*	CTRX 1–2 g, every 24 h [5]	See CQ4–2 for ESBL-producing bacteria risk.
	Community-acquired (high risk of ESBL-producing bacteria)			CMZ 1–2 g, every 8 h [7, 8] or TAZ/PIPC 4.5 g, every 8 h [9] or MEPM 1 g, every 8 h [5]	
	Healthcare-associated		*E. coli, Klebsiell*a spp., *Enterobacter* spp., *Pseudomonas aeruginosa*, *Enterococcus* spp.	“TAZ/PIPC 4.5 g, every 8 h or MEPM 1 g, every 8 h” ±VCM [5],^k^	VCM is added when Gram staining shows Streptococcus-like Gram-positive cocci.
**Biliary tract / intra-abdominal infection**^c)^	Community-acquired (low risk of ESBL-producing bacteria)		*E. coli*, anaerobic bacteria such as *Bacteroides* spp.	SBT/ABPC 3 g, every 6 h [10] or “CTRX 2 g, every 24 h + MNZ 500 mg, every 8 h” [10]	See CQ4–2 for ESBL-producing bacteria risk. Check antibiogram of facility / region to see if SBT / ABPC can be selected.
	Community-acquired (high risk of ESBL-producing bacteria)			CMZ 1–2 g, every 8 h [10] or TAZ/PIPC 4.5 g, every 8 h	
	Healthcare-associated		*E. coli*, anaerobic bacteria such as *Bacteroides* spp., *Enterobacter* spp., *Pseudomonas aeruginosa*, *Enterococcus* spp. ± *Candida* spp.	“TAZ/PIPC 4.5 g, every 8 h or (CFPM 2 g, every 8 h + MNZ 500 mg, every 8 h) or MEPM 1 g, every 8 h” [5, 10] ±MCFG 100 mg, every 24 h [5]	See CQ4–3 for *Candida* risk.
**Necrotic soft tissue infection**^d)^	Monomicrobial infection suspected (Gram-positive cocci or Gram-positive rods)		β-hemolytic Streptococci, *Clostridium* spp., rarely *Staphylococcus aureus* (including community-acquired MRSA)	“CTRX 2 g, every 24 h or SBT/ABPC 3 g, every 6 h” ±VCM [5],^k^ ±CLDM 600 mg, every 8 h [5]	See CQ4–3 for MRSA risk. CLDM is intended for suppressing toxin production in toxic shock syndrome.
	Polymicrobial infection suspected (diabetic, Fournier’s gangrene)		*Staphylococcus aureus*, *E. coli*, anerobic bacteria	TAZ/PIPC 4.5 g, every 8 h [5] ±VCM [5],^k^	
	Exposure to seawater / freshwater		*Aeromonas* spp., *Vibrio vulnificus*	CTRX 2 g, every 24 h +MINO 100 mg, every 12 h [5]	
**Vertebral osteomyelitis (spondylitis)**^e^	Community-acquired		MSSA, *Streptococcus* spp., rarely *Streptococcus pneumoniae*, Gram-negative bacilli	CEZ 2 g, every 8 h [5] or CTRX 2 g, every 24 h [5]	See CQ4–3 for MRSA risk.
	Healthcare-associated		*Staphylococcus aureus*, Gram-negative bacillus	CFPM 2 g, every 12 h +VCM [5],^k^	
**Endocarditis**^f^	Native valve: without MRSA risk		MSSA, *Streptococcus* spp., *Enterococcus* spp.	SBT/ABPC 3 g, every 6 h [5] or “CTRX 2 g, every 24 h +ABPC 2 g, every 4 h” [5, 11]	Select “CTRX+ABPC” when there is a high possibility of enterococcus. Select CTRX 2 g every 12 h if there is an intracranial disseminated lesion.
	Native-valve: with MRSA risk		Above+MRSA	CTRX 2 g, every 24 h +VCM [5, 11],^k^	Select CTRX 2 g every 12 h if there is an intracranial disseminated lesion. See CQ4–3 for MRSA risk.
	Prosthetic valve or pacemaker		Above+*Staphylococcus epidermidis*, Gram-negative bacilli	“CTRX 2 g, every 24 h or CFPM 2 g, every 12 h” +VCM [5, 11],^k^	
**Mycotic aneurysm**^g^	Community-acquired/native arteries		*Staphylococcus aureus*, *Salmonella* spp., Gram-negative bacilli	“CFPM 2 g, every 12 h or TAZ/PIPC 4.5 g, every 8 h” ±VCM^k^	See CQ4–3 for MRSA risk.
	Prosthetic vascular graft infections		*Staphylococcus aureus*, *Staphylococcus epidermidis*, *Pseudomonas aeruginosa*	“CFPM 1 g, every 8 h or TAZ/PIPC 4.5 g, every 8 h or MEPM 1 g, every 8 h” +VCM^k^	
**Catheter-related bloodstream infections**^h^	Intravascular catheter		*Staphylococcus epidermidis*, *Staphylococcus aureus* (including MRSA), *E. coli*, Pseudomonas aeruginosa, ±Candida	VCM^k^ +CFPM 2 g, every 8–12 h ±MCFG 100 mg, every 24 h [5]	See CQ4–3 for *Candida* risk
**Meningitis**^i^	Community-acquired (in a patient younger than 50 years)		*Streptococcus pneumoniae*, *Neisseria meningitidis*	CTRX 2 g, every 12 h +VCM [5, 12] ^,k^	
	Community-acquired (patient older than 50 years, cell-mediated immunodeficiency)		*Streptococcus pneumoniae*, *Neisseria meningitidis*, *Listeria monocytogenes*	ABPC 2 g, every 4 h +CTRX 2 g, every 12 h +VCM [5, 12] ^,k^	
	Post-neurosurgery or shunt-related meningitis		MRSA, *Pseudomonas aeruginosa*	“CAZ or CFPM or MEPM (2 g, every 8 h)” +VCM [5, 12], ^k^	
**Unknown or systemic source**^j^	Community-acquired (not any of the items listed below)		*Streptococcus pneumoniae*, *Neisseria meningitidis*,β-hemolytic streptococcus, *E. coli*	CTRX 2 g, every 24 h [5]	See section on meningitis if there is a possibility of meningitis
	Healthcare-associated (not any of the items listed below)		*Pseudomonas aeruginosa*, MRSA	“CFPM 2 g, every 8 h or TAZ/PIPC 4.5 g, every 8 h or MEPM 2 g, every 8 h” +VCM^k^	
	Toxic shock syndrome		*Staphylococcus aureus*, β-hemolytic streptococcus, *Clostridium* spp.	“CTRX 2 g, every 24 h or SBT/ABPC 3 g, every 6 h” +CLDM 600 mg, every 8 h ±VCM^k^	See CQ4–3 for MRSA risk
	Rickettsia endemic areas		Japanese spotted fever, scrub typhus	MINO 100 mg, every 12 h [13]	
	Febrile neutropenia		*Pseudomonas aeruginosa*, MRSA	CFPM 2 g, every 12 h +VCM [5],^k^	See CQ4–2 for anti-Pseudomonal drugs
	After splenectomy		Pneumococcus, *Neisseria meningitidis*, *Haemophilus influenzae*, *Capnocytophaga* spp.	When there is no possibility of meningitis: CTRX 2 g, every 24 h [5]	See section on meningitis if there is a possibility of meningitis
	Shock +rash		Purpura fulminans (meningococcus, pneumococcus), *Rickettsia* spp.	CTRX 2 g, every 12 h +VCM [5] +MINO 100 mg, every 12 h [13, 14]	See section on endocarditis if there is a possibility of endocarditis

[Precautions] This table is a list of infectious diseases related to sepsis based on guidelines for various infectious diseases and those published by the Japanese Association for Infectious Diseases and the Japanese Society of Chemotherapy, with the following information added. Typical options are shown to make the table practical for use

Given their very nature, empiric therapeutic agents are difficult to present as the only absolute option, and they are often presented in various guidelines as evidence and expert opinion suggestions. However, this also depends on the age and region of the antibiograms produced, and the types of antimicrobial agents available at each facility. This table can be used as a reference for experts in the septic/antimicrobial stewardship teams of each facility when developing antimicrobial guidelines for each facility

Abbreviations: *ABPC* ampicillin, *AZM* azithromycin, *CAZ* ceftazidime, *CFPM* cefepime, *CLDM* clindamycin, *CMZ* cefmetazole, *CTRX* ceftriaxone, *GM* gentamycin, *MCFG* micafungin, *MEPM* meropenem, *MINO* minocycline, *MNZ* metronidazole, *SBT/ABPC* sulbactam/ampicillin, *ST* sulfamethoxazole/trimethoprim, *TAZ/PIPC* tazobactam/piperacillin, *VCM* vancomycin (abbreviations of antimicrobial agents are based on JAID/JSC infectious disease treatment guidelines)

^a^Pneumonia: Staphylococcus aureus (including MRSA) can be a causative bacterium in addition to the usual causes of community-acquired pneumonia following influenza virus infection or necrotizing pneumonia; thus, a separate section has been created

^b^Urinary tract infection: Presented based on reports of epidemiology and treatment of ESBL-producing bacteria in Japan

^c^Biliary tract/intra-abdominal infection: Presented based on reports of epidemiology and treatment of ESBL-producing bacteria in Japan

^d^Necrotic soft tissue infection: Three types are presented as options when the causative bacteria can be estimated from the patient background (exposure history, underlying disease) and clinical course (rapid inspection results of the test incision sample are also taken into consideration)

^e^Vertebral osteomyelitis (spondylitis): Refraining from empiric therapeutic drugs is recommended for hemodynamically and neurologically stable spondylitis; however, empiric treatment is indicated when complications of sepsis are present [15]. The regimen of empiric treatment is not established, but options were selected based on the JAID/JSC infectious disease treatment guidelines [5]

^f^Endocarditis: Concomitant use of GM in native valve endocarditis was previously recommended for Staphylococcus aureus [5], but this is no longer recommended in recent years [16]. A combination regimen of CTRX and ABPC was indicated in place of GM for enterococci. In addition, a regimen without the concomitant use of GM was shown as an empiric treatment [16]. There was also no description in the JAID/JSC infectious disease treatment guideline in cases of endocarditis with a high rate of intracranial dissemination; however, this table presents this considering cerebrospinal fluid penetration. We presented an option for endocarditis of the prosthetic valve that does not include GM as an empiric treatment when the causative organism is uncertain, considering the nephrotoxicity of GM

^g^Mycotic aneurysm: There is no description in the JAID/JSC infectious disease treatment guidelines and no established recommendation exists [5, 17], but this was presented as an option

^h^Catheter-related bloodstream infections: options were presented based on the JAID/JSC infectious disease treatment guidelines [5]

^i^Meningitis: options were presented based on the JAID/JSC infectious disease treatment guidelines [5, 12]

^j^Unknown or systemic sources: There is no description in the JAID/JSC infectious disease treatment guidelines, but the source of infection is occasionally unknown in sepsis, so options for each possible pathology were presented

^k^ Please refer to the description of the TDM guideline 2016 (initial loading dose: 25–30 mg/kg intravenous injection, subsequent maintenance doses (normal renal function):15–20 mg/kg intravenous injection, every 12 h) for the VCM dose [18]

**CQ4-2: Under what circumstances should carbapenems be used in empirical antimicrobial therapy?**

***Answer:*** Carbapenems can be included in the empirical antimicrobial regimen when the use of carbapenem is considered to be particularly effective; ESBL-producing Enterobacteriaceae or *Pseudomonas aeruginosa* or Acinetobacter species with limited susceptibility for carbapenems (Provision of information for background question).

**CQ4–3: Under what circumstances should empirical antimicrobial therapy be selected for MRSA and non-bacterial pathogens (e.g., Candida, Viruses, Legionella, Rickettsia, or** ***Clostridioides difficile*)?**

***Answer:*** Each microorganism can be covered by empirical antimicrobial regimen if highly suspected by suspected infectious foci, patient background and culture results (Provision of information for background question).

**CQ4-4: Should empirical antimicrobial therapy be suspended if culture results were negative?**

***Answer:*** We suggest stopping any empiric antimicrobials where sepsis is excluded by negative culture results and after careful consideration of clinical progress (expert consensus: insufficient evidence).

**CQ4-5: Under what circumstances should an infectious disease specialist or antimicrobial stewardship team be consulted?**

***Answer:*** An infectious disease specialist and/or antimicrobial stewardship team can be consulted when 1) the cause of sepsis is unknown, 2) involvement of extensively drug-resistant bacteria is suspected, 3) emerging, re-emerging, or imported infectious diseases are suspected, or 4) in cases of *Staphylococcus aureus* bacteremia or candidemia (Provision of information for background question).

**CQ4-6: Should empirical antibacterial drugs for sepsis begin within 1 h upon identification of sepsis?**

***Answer:*** We suggest that antibacterial drugs be administered as soon as possible upon identification of sepsis or septic shock, but we suggest against using the target time of less than 1 h (GRADE 2C: certainty of evidence = “low”).

**CQ4-7: Should continuous or extended infusion of β-lactam antibiotics be used for sepsis?**

***Answer:*** We suggest using continuous or extended infusion of β-lactam antimicrobials (GRADE 2B: certainty of evidence = “moderate”).

**CQ4-8: Should de-escalation antimicrobial therapy be used for sepsis?**

***Answer:*** We suggest applying de-escalation antimicrobial therapy for sepsis (GRADE 2D, certainty of evidence = “very low”).

**CQ4-9: Should procalcitonin be used as an indicator for stopping antimicrobial therapy for sepsis?**

***Answer:*** We suggest using procalcitonin as an indicator for stopping antimicrobial therapy for sepsis (GRADE 2B, certainty of evidence = “moderate”).

**CQ4-10: Should relatively short-term (i.e. within 7 days) antimicrobial therapy be applied for sepsis?**

***Answer:*** We suggest applying relatively short-term (i.e. within 7 days) antimicrobial therapy for sepsis (GRADE 2D: certainty of evidence = “very low”).

**CQ4-11: What should be used as a reference for adjusting the dose for renal-excretion antimicrobial drugs?**

***Answer:*** Changes in bodily fluid volume and the presence of renal replacement therapy and other extracorporeal circulation therapies in addition to renal function test values (e.g., serum Cr level, eGFR level) measured at multiple time points are informative (Provision of information for background question).

CQ5: Intravenous immunoglobulin therapy

**CQ5-1: Should intravenous immunoglobulin (IVIG) be administered to adult patients with sepsis?**

***Answer:*** We suggest against administering IVIG to patients with sepsis (GRADE 2B: certainty of evidence = “moderate”).

**CQ5-2-1: Should IVIG be administered to patients with streptococcal toxic shock syndrome (STSS)?**

***Answer:*** We suggest administering IVIG to patients with STSS (GRADE 2D: certainty of evidence = “very low”).

**CQ5-2-2: Should IVIG be administered to patients with staphylococcal toxic shock syndrome (staphylococcal TSS)?**

***Answer:*** We suggest against administering IVG to patients with staphylococcal TSS (expert consensus: insufficient evidence).

CQ6: Initial resuscitation/inotropes

**CQ6-1: Should echocardiography be conducted in patients with sepsis?**

***Answer:*** We suggest, following initial fluid resuscitation, conducting cardiac function and hemodynamics assessments with echocardiography in patients with sepsis/septic shock (GRADE 2D: certainty of evidence = “very low”).

**CQ6-2: Is EGDT recommended for initial resuscitation in patients with sepsis?**

***Answer:*** We suggest against conducting EGDT as initial resuscitation in patients with sepsis/septic shock (GRADE 2C: certainty of evidence = “low”).

**CQ6-3: Should vasopressors be used simultaneously or in the early stage (within 3 h) of initial fluid resuscitation in adult patients with sepsis?**

***Answer:*** We suggest administering vasopressors simultaneously or in the early stages (within 3 h) of initial fluid resuscitation in patients with sepsis/septic shock who have difficult maintaining hemodynamics (GRADE 2C: certainty of evidence = “low”).

**CQ6-4: Should lactate levels be used as an indicator for initial resuscitation in adult patients with sepsis?**

***Answer:*** We suggest using lactate levels as an indicator of tissue hypoperfusion during initial resuscitation in patients with sepsis/septic shock (GRADE 2C: certainty of evidence = “low”).

**CQ6-5: What is the initial fluid infusion rate and volume in adult patients with sepsis?**

***Answer:*** There is an opinion that the initial fluid resuscitation in patients with reduced intravascular volume due to sepsis should be administered over 30 mL/kg of crystalloid solution within 3 h, aiming to optimize the circulating blood volume. It is important during initial fluid resuscitation to carefully observe vital signs and to avoid excessive fluid loads by using lactate clearance and echocardiography while conducting tissue oxygen metabolism and hemodynamics assessments (Provision of information for background question).

**CQ6-6: How should fluid responsiveness be assessed in adult patients with sepsis?**

***Answer:*** Fluid responsiveness is significant increase in stroke volume (SV) after fluid infusion, and multiple parameters, including static and dynamic parameters, should be used to predict fluid responsiveness. Static parameters, including central venous pressure (CVP) and pulmonary capillary wedge pressure (PCWP), are measured at a point. Dynamic parameters include changes in cardiac output by passive leg raising (PLR) and fluid challenge, pulse pressure variation (PPV) and stroke volume variation (SVV) during mechanical ventilation (Provision of information for background question).

**CQ6-7: Should albumin solution be used for initial resuscitation in adult patients with sepsis?**

***Answer:*** We suggest against administering albumin solution as a standard treatment at the beginning of initial fluid resuscitation in patients with sepsis (GRADE 2C: certainty of evidence = “low”). Albumin solution can be used in patients with sepsis when patients do not respond to standard treatment and require substantial amounts of crystalloids (expert consensus: insufficient evidence).

**CQ6-8: Should artificial colloids be used for initial resuscitation in adult patients with sepsis?**

***Answer:*** We suggest against administering artificial colloids in patients with sepsis/septic shock (GRADE 2D: certainty of evidence = “very low”).

**CQ6-9-1: Should noradrenaline, dopamine, or phenylephrine be used as a first-line vasopressor in adult patients with sepsis? noradrenaline vs. dopamine**

***Answer:*** Between noradrenaline and dopamine, we suggest administering noradrenaline as a first-line vasopressor in adult patients with sepsis (GRADE 2D: certainty of evidence = “very low”).

**CQ6-9-2: Should noradrenaline, dopamine, or phenylephrine be used as a first-line vasopressor in adult patients with sepsis? noradrenaline vs. phenylephrine**

***Answer:*** Between noradrenaline and phenylephrine, we suggest administering noradrenaline as a first-line vasopressor in adult patients with sepsis (GRADE 2D: certainty of evidence = “very low”).

**CQ6-10-1: Should adrenaline be used as a second-line vasopressor in adult patients with sepsis?**

***Answer:*** We suggest against using adrenaline as a second-line vasopressor in patients with sepsis/septic shock (GRADE 2D: certainty of evidence = “very low”).

**CQ6-10-2: Should vasopressin be used as a second-line vasopressor in adult patients with sepsis?**

***Answer:*** We suggest using vasopressin as a second-line vasopressor in patients with sepsis/septic shock (GRADE 2D: certainty of evidence = “very low”).

**CQ6-11: Should inotropes be used in adult patients with sepsis accompanied by cardiogenic shock?**

***Answer:*** We suggest administering inotropes (adrenaline, dobutamine) in adult patients with septic shock accompanied by cardiac dysfunction (expert consensus: insufficient evidence).

**CQ6-12: Should β-blockers be used in adult patients with sepsis?**

***Answer:*** We suggest administering short-acting β1-adrenoceptor antagonists in patients with sepsis/septic shock while being monitored with the objectives of managing tachycardia which cannot be controlled with standard therapy like initial fluid resuscitation (GRADE 2D: certainty of evidence = “very low”). Administering short-acting β1-adrenoceptor antagonists can induce hemodynamic fluctuations, so they should be administered under the supervision of a physician with expertise in cardiovascular management in the intensive care unit (expert consensus: insufficient evidence).

**CQ6-13: What are the indications of assisted circulation in adult patients with septic shock?**

***Answer:*** There is insufficient evidence for the effects of assisted circulation such as veno-arterial extracorporeal membrane oxygenation (V-A ECMO) and intra-aortic balloon pump (IABP) for cardiac dysfunction in septic shock, and its applications are still under investigation (Provision of information for background question).

**CQ7: Corticosteroid therapy**

**CQ7-1: Should low-dose corticosteroids (hydrocortisone) be administered to adult patients with septic shock who do not respond to initial fluid resuscitation and vasopressors?**

***Answer:*** We suggest administering low-dose corticosteroids (hydrocortisone) to adult patients with septic shock who do not respond to initial fluid resuscitation and vasopressors for the purpose of withdrawing from shock (GRADE 2D: certainty of evidence = “very low”).

**CQ7-2: Should hydrocortisone and fludrocortisone be administered to patients with septic shock who do not respond to initial fluid resuscitation and vasopressors?**

***Answer:*** We suggest concomitant administration of hydrocortisone and fludrocortisone to adult patients with septic shock who do not respond to initial fluid resuscitation and vasopressors (GRADE 2C: certainty of evidence = “low”).

**CQ7-3: Should corticosteroids (hydrocortisone) be administered to patients with sepsis without shock?**

***Answer:*** We suggest against administering hydrocortisone to patients with sepsis without shock (GRADE 2D: certainty of evidence = “very low”).

CQ8: Blood transfusion therapy

**CQ8-1: How should blood transfusion be conducted during the initial resuscitation of septic shock?**

***Answer:*** We suggest starting blood transfusion at a hemoglobin level of less than 7 g/dL during initial resuscitation for patients with septic shock (GRADE 2C: certainty of evidence = “low”).

**CQ8-2: How should blood transfusion be conducted during hemodynamically stable sepsis?**

***Answer:*** We suggest starting blood transfusion at a hemoglobin level of less than 7 g/dL in patients with hemodynamically stable sepsis (expert consensus: insufficient evidence).

**CQ8-3: How should fresh frozen plasma be administered in patients with sepsis?**

***Answer:*** We suggest administering fresh frozen plasma in patients with sepsis when hemorrhaging tendencies are observed. If surgical/invasive interventions are required, we suggest administering when PT/APTT is extended (PT is over INR 2.0 or activity level of less than 30%; APTT is over two times the upper limit of standards at each medical institution or activity level less than 25%) or when fibrinogen levels are less than 150 mg/dL (expert consensus: insufficient evidence).

**CQ8-4: How should platelet transfusion be conducted for patients with sepsis?**

***Answer:*** We suggest conducting platelet transfusion in patients with sepsis and platelet counts of less than 10,000/μL, or less than 50,000/μL when accompanied by hemorrhaging symptoms (expert consensus: insufficient evidence). We suggest conducting platelet transfusion so as to maintain a platelet count of over 50,000/μL when active hemorrhaging is observed or when surgical/invasive procedures are needed (expert consensus: insufficient evidence).

**CQ9: Respiratory management**

**CQ9-1: What is the S**_**P**_**O**_**2**_ **range for respiratory management in adult patients with sepsis?**

***Answer:*** We suggest against setting a high target S_P_O_2_ (98–100%) during respiratory management in adult patients with sepsis (GRADE 2B: certainty of evidence = “moderate”).

***Remarks:*** This does not apply in cases where there is the possibility of a disruption in the oxygen supply/demand balance due to severe anemia or increased metabolism due to infection in cases where hemodynamics are unstable.

**CQ9-2: Should non-invasive ventilation (NIV) or nasal high-flow therapy (NHFT) be conducted for early respiratory failure in adult patients with sepsis?**

***Answer:*** We suggest conducting non-invasive ventilation (NIV) or nasal high-flow therapy (NHFT) for early respiratory failure in adult patients with sepsis (GRADE 2A: certainty of evidence = “high”).

**CQ9-3: Should protective ventilation strategies be implemented for ventilation management in adult patients with sepsis?**

***Answer:*** We suggest implementing protective ventilation strategies for ventilation management in adult patients with sepsis (GRADE 2B: certainty of evidence = “moderate”).

**CQ9-4: Should high PEEP settings be utilized for ventilation management in adult patients with sepsis?**

***Answer:*** We suggest against utilizing high PEEP settings (PEEP over 12 cm H_2_O) for the initial stage of ventilation management in adult patients with sepsis (GRADE 2B: certainty of evidence = “very low”).

**CQ9-5: Should spontaneous breathing trials (SBT) be conducted prior to extubation in adult patients with sepsis placed under ventilation management?**

***Answer:*** We suggest utilizing weaning protocols from ventilators, including spontaneous breathing trials (SBTs) prior to extubation in adult patients with sepsis placed under ventilation management (GRADE 2D: certainty of evidence = “very low”).

**CQ9–6: Should preventative non-invasive ventilation (NIV) or nasal high-flow therapy (NHFT) be conducted after extubation for adult patients with sepsis placed under ventilation management?**

***Answer:*** We suggest conducting preventative non-invasive ventilation (NIV) or nasal high-flow therapy (NHFT) over standard oxygen therapy following extubation for adult patients with sepsis placed under ventilation management (GRADE 2B: certainty of evidence = “moderate”).

CQ10: Management of pain, agitation, and delirium

**CQ10-1: Should management based on analgesia-first sedation protocol be used for adult patients with sepsis on mechanical ventilation?**

***Answer:*** We suggest using management based on analgesia-first sedation protocol in adult patients with sepsis on mechanical ventilation (GRADE 2C: certainty of evidence = “low”).

**CQ10-2: Should propofol or dexmedetomidine be prioritized over benzodiazepines as sedatives for adult patients with sepsis on mechanical ventilation?**

***Answer:*** We suggest using propofol or dexmedetomidine over benzodiazepines as sedatives for patients with sepsis on mechanical ventilation (GRADE 2D: certainty of evidence = “very low”).

**CQ10–3: Should light sedation through the interruption of sedatives once a day or sedative adjustments based on protocol be used for adult patients with sepsis on mechanical ventilation?**

***Answer:*** We suggest using light sedation through the interruption of sedatives once a day or sedative adjustments based on protocol for patients with sepsis on mechanical ventilation (GRADE 2C: certainty of evidence = “low”).

**CQ10-4: Should drug therapy be used to prevent delirium in adult patients with sepsis?**

***Answer:*** We suggest administering dexmedetomidine for delirium prevention in adult patients with sepsis (GRADE 2C: certainty of evidence = “low”). We suggest against the administration of haloperidol (GRADE 2B: certainty of evidence = “moderate”). We suggest against the administration of atypical antipsychotics (GRADE 2C: certainty of evidence = “low”). We suggest against the administration of statins (GRADE 2D: certainty of evidence = “very low”).

***Remarks:*** We recommend against the routine administration of dexmedetomidine to patients who do not require sedation. Furthermore, dexmedetomidine administration can cause hemodynamic fluctuations, so this should ideally be administered under the supervision of a physician who is experienced with systematic management in an intensive care unit (expert consensus).

**CQ10-5: Should drug therapy be used to treat delirium in adult patients with sepsis?**

***Answer:*** We suggest against administering dexmedetomidine for delirium treatment in adult patients with sepsis (GRADE 2D: certainty of evidence = “very low”). We suggest against administering haloperidol (GRADE 2C: certainty of evidence = “low”). We suggest against administering atypical antipsychotics (GRADE 2B: certainty of evidence = “moderate”).

***Remarks:*** The use of dexmedetomidine, haloperidol, or atypical antipsychotics should not be prevented when the patient’s life or body is at risk due to hyperactive delirium.

**CQ10-6: Should non-drug therapy be used to prevent delirium in adult patients with sepsis?**

***Answer:*** We suggest using non-drug therapy to prevent delirium in adult patients with sepsis (GRADE 2C: certainty of evidence = “low”).

CQ11: Acute kidney injury/blood purification

**CQ11-1: Should furosemide be used to prevent or treat septic AKI?**

***Answer:*** We suggest against using furosemide for preventing or treating septic AKI (GRADE 2C, certainty of evidence = “low”).

**CQ11-2: Should atrial natriuretic peptide (ANP) be used to prevent or treat septic AKI?**

***Answer:*** We suggest against using ANP for preventing or treating septic AKI (GRADE 2D, certainty of evidence = “very low”).

**CQ11-3: Should dopamine be used to prevent or treat septic AKI?**

***Answer:*** We suggest against using dopamine for preventing or treating septic AKI (GRADE 2C, certainty of evidence = “low”).

**CQ11-4: Should continuous renal replacement therapy (RRT) rather than intermittent RRT be used for the management of septic AKI?**

***Answer:*** Either continuous or intermittent RRT can be selected for septic AKI (GRADE 2C, certainty of evidence = “low”). Continuous RRT should be used for hemodynamically unstable patients (Good Practice Statement).

**CQ11-5-1: Should RRT be initiated early for septic AKI (Stage 2 vs. Stage 3 or absolute indications)?**

***Answer:*** We make no recommendation on whether RRT should be initiated early at Stage 2 for patients with septic AKI.

**CQ11-5-2: Should RRT be initiated early for septic AKI (Stage 3 vs. absolute indications)?**

***Answer:*** We suggest against initiating RRT at Stage 3 for patients with septic AKI rather than absolute indication (GRADE 2D, certainty of evidence = “very low”).

**CQ11-6: Should a large RRT dose be delivered for septic AKI?**

***Answer:*** We suggest against increasing a RRT dose beyond the standard dose for patients with septic AKI (GRADE 2C, certainty of evidence = “low”).

**CQ11-7: Should PMX-DHP be used for patients with septic shock?**

***Answer:*** We suggest against using PMX-DHP for patients with septic shock (GRADE 2B, certainty of evidence = “moderate”).

CQ12: Nutrition support therapy

**CQ12-1: Should either enteral nutrition or parenteral nutrition be given for nutrition administration in septic patients?**

***Answer:*** We suggest enteral nutrition be administered for septic patients. (GRADE 2D: certainty of evidence = “very low”).

**CQ12-2: Should hemodynamically unstable septic shock patients receive enteral nutrition?**

***Answer:*** We suggest against administering enteral nutrition in hemodynamically unstable septic shock patients (GRADE 2D: certainty of evidence = “very low”).

**CQ12-3: When should enteral nutrition be initiated in septic patients?**

***Answer:*** We suggest initiating enteral nutrition at an early period of acute phase (within 24–48 h following the start of treatment to critical illness) for septic patients (GRADE 2D: the certainty of evidence = “very low”).

**CQ12-4: Should the septic patients receive enteral nutrition less than their energy expenditure in the acute phase?**

***Answer:*** We suggest the septic patients receive enteral nutrition less than their energy expenditure in the acute phase. (GRADE 2B: certainty of evidence = “moderate”).

**CQ12-5: Should parenteral nutrition be combined with enteral nutrition in septic patients?**

***Answer:*** We suggest supplemental parenteral nutrition be combined in septic patients receiving insufficient amount of enteral nutrition (GRADE 2D: certainty of evidence = “very low”).

**CQ12-6: What is the optimal protein dose in the acute phase for septic patients?**

***Answer:*** We suggest providing less than 1 g/kg/day of protein (peptides, amino acids) to septic patients in the acute phase (GRADE 2D: certainty of evidence = “very low”).

**CQ12-7-1: Should vitamin C be actively provided to septic patients in the acute phase?**

***Answer:*** We suggest providing vitamin C to septic patients (GRADE 2D: certainty of evidence = “very low”).

**CQ12-7-2: Should vitamin D be actively provided to septic patients in the acute phase?**

***Answer:*** We suggest against providing vitamin D in septic patients (GRADE 2D: certainty of evidence = “very low”).

**CQ12-8: What are the methods for determining enteral nutrition initiation and monitoring intolerance in septic patients?**

***Answer:*** Findings such as bowel sounds, which indicate contractility of the gastrointestinal tract, at the start of enteral nutrition should not be required. Meanwhile, various findings show intolerance following the initiation of enteral nutrition, including the lack of intestinal sounds, abnormal intestinal sounds, vomiting, intestinal dilation, diarrhea, gastrointestinal bleeding, and excessive gastric residue. Excessive gastric residue suggests intolerance, but the gastric residue volume criteria for determining the presence of intolerance are unknown (Provision of information for background question).

**CQ12-9: What nutrition support therapy should be provided to septic patients after the acute phase?**

***Answer:*** Provision of energy that meets the goals (around 25–30 kcal/kg/day, including protein) is thought to be needed when the patients overcome the clinical conditions of acute phase, or where about 1 week has passed following the onset of critical illness. Some experts are of the opinion that protein dose of over 1 g/kg/day is ideal in this phase. However, there are other expert opinions that the energy dose should be increased at an earlier phase for patients with malnutrition prior to exacerbation of the disease (Provision of information for background question).

CQ13: Blood glucose management

**CQ13-1: Should blood glucose be measured using a glucometer with capillary blood in septic patients?**

***Answer:*** We suggest against the use of a glucometer with capillary blood in patients with sepsis (GRADE 2A: certainty of evidence = “high”).

**CQ13-2: What is the optimal blood glucose target level in septic patients?**

***Answer:*** We suggest an optimal target blood glucose range of 144–180 mg/dL in septic patients (GRADE 2D: certainty of evidence = “very low”).

CQ14: Body temperature control

**CQ14-1: Should antipyretic therapy be applied to sepsis patients presenting with fever?**

***Answer:*** We suggest against conducting antipyretic therapy to sepsis patients presenting with fever (GRADE 2A: certainty of evidence = “high”).

**CQ14-2: Should rewarming therapy be applied to hypothermic sepsis patients?**

***Answer:*** We suggest attempting to correct the body temperature of hypothermic (core body temperature < 35 °C) sepsis patients while considering hemodynamic stabilization when hemodynamic disorders and coagulation abnormalities related to hypothermia are observed (expert consensus: insufficient evidence).

CQ15: Diagnosis and treatment of disseminated intravascular coagulation in patients with sepsis

**CQ15-1: What is the diagnosis method for septic disseminated intravascular coagulation (DIC)?**

***Answer:*** There are multiple diagnostic criteria for conducting DIC diagnosis. The acute DIC diagnostic criteria are widely used in Japan, while the ISTH overt-DIC is used as the international standard. It is difficult to determine the superiority between diagnostic criteria, and these should be used according to the purpose (Provision of information for background question).

**CQ15-2: What are differential diseases for patients where septic DIC is suspected?**

***Answer:*** Thrombotic thrombocytopenic purpura (TTP), hemolytic uremic syndrome (HUS) and heparin-induced thrombocytopenia (HIT) are common DIC-like pathological conditions. These types of diseases require managements that are different from that of DIC (Provision of information for background question).

**CQ15-3: Should antithrombin replacement therapy be administered in sepsis-associated DIC?**

***Answer:*** We suggest antithrombin replacement therapy for patients with sepsis-associated DIC (GRADE 2C, certainty of evidence = “low”).

**CQ15-4: Should heparin or heparin analogs be administered in sepsis-associated DIC?**

***Answer:*** We suggest against administering heparin or heparin analogs as a standard treatment for patients with sepsis-associated DIC (GRADE 2D, certainty of evidence = “very low”).

**CQ15-5: Should recombinant thrombomodulin be administered to patients with sepsis-associated DIC?**

***Answer:*** We suggest administering recombinant thrombomodulin for patients with sepsis-associated DIC (GRADE 2C, certainty of evidence = “low”).

**CQ15-6: Should protease inhibitors be administered to patients with sepsis-associated DIC?**

***Answer:*** We suggest against administering protease inhibitors as standard treatment for patients with sepsis-associated DIC (GRADE 2D, certainty of evidence = “very low”).

CQ16: Venous thromboembolism countermeasures

**CQ16-1: Should mechanical prophylaxis (elastic stockings, intermittent pneumatic compression) be used to prevent deep vein thrombosis during sepsis?**

***Answer:*** We suggest using mechanical prophylaxis (elastic stockings, intermittent pneumatic compression) to prevent deep vein thrombosis in patients with sepsis (expert consensus: insufficient evidence).

**CQ16-2: Should anticoagulation therapy (unfractionated heparin, low-molecular-weight heparin, warfarin, NOAC/DOAC) be conducted to prevent deep vein thrombosis during sepsis?**

***Answer:*** We suggest conducting anticoagulation therapy to prevent deep vein thrombosis in patients with sepsis (expert consensus: insufficient evidence).

**CQ16-3: For how long should VTE prophylaxis be conducted in patients with sepsis?**

***Answer:*** We suggest conducting venous thromboembolism (VTE) prophylaxis in patients with sepsis until they are able to walk or discharged from the hospital (expert consensus: insufficient evidence).

CQ17: ICU-acquired weakness and early rehabilitation

**CQ17-1: Should early rehabilitation be implemented to prevent PICS?**

***Answer:*** We suggest conducting early rehabilitation to prevent PICS in patients with sepsis (GRADE 2D, certainty of evidence = “very low”).

**CQ17-2: Should passive joint exercise therapy be conducted to prevent ICU-AW in patients with sepsis?**

***Answer:*** We suggest conducting passive mobilization as standard treatment for patients with sepsis (GRADE 2D: certainty of evidence = “very low”).

**CQ17-3: Should neuromuscular electrical stimulation be used to prevent ICU-AW?**

***Answer:*** We suggest against using neuromuscular electrical stimulation as a standard treatment to prevent ICU-AW in patients with sepsis (GRADE 2D: certainty of evidence = “very low”).

CQ18: Pediatric considerations

**CQ18-1: Should the initial resuscitation algorithm be used for pediatric sepsis?**

***Answer:*** We suggest using the initial resuscitation algorithm for pediatric sepsis (GRADE 2D: certainty of evidence = “very low”).

**CQ18-2: How should empirical antibacterial drugs be selected for pediatric sepsis where the source of infection is difficult to estimate?**

***Answer:*** Antibacterial drugs which cover the possible microorganisms should be selected with consideration of the site of occurrence (e.g., community, hospital, intensive care unit) and patient background (e.g., immune status, treatment history) (see Table 3 for reference) (Provision of information for background question).

**Table 3:** Thresholds and limits of dynamic indicators

Method	Threshold	Main limits
PPV (pulse pressure variation) SVV (stroke volume variation)	12%	Difficult to use in the following cases: patients with spontaneous breathing, patients with arrhythmia, patients with low tidal ventilation, and patients with low lung compliance
IVC diameter fluctuations	12%	Difficult to use in the following cases: patients with spontaneous breathing, patients with arrhythmia, and patients with low lung compliance
SVC diameter fluctuations	12–40%	Requires transesophageal echocardiography. Difficult to use in the following cases: patients with spontaneous breathing, patients with low tidal ventilation, and patients with low lung compliance
PLR (passive leg raising)	10%	Cardiac output is to be directly measured Difficult to use in the following cases: patients with lower limb defects, pregnant women, patients receiving vasoactive drugs, and patients with increased intra-abdominal pressure
EEO (end-expiratory occlusion test)	5%	Difficult to use in the following cases: non-intubated patients and patients who cannot hold their breath for more than 15 s
Low-dose fluid challenge (100 mL)	6–10%	Cardiac output needs to be measured directly and accurately
Fluid challenge (500 mL)	15%	Risk of fluid overload if repeated. Cardiac output needs to be measured directly

**CQ18-3: Under what scenarios should anti-herpetic agents be included in empirical treatment for pediatric sepsis?**

***Answer:*** There are cases where a central nervous system infection is suspected or a bacterial source of infection cannot be specified in neonates, because the prevalence of the herpes simplex virus is higher and they can easily become severe once infected (Provision of information for background question).

**CQ18-4: What is the optimal blood pressure for hemodynamic management in pediatric sepsis?**

***Answer:*** Suitable values for the optimal blood pressure are unknown, and this should be set with consideration to age and organ perfusion. The median value for the mean blood pressure “55 + age x 1.5 mmHg” and the 5th percentile value “40 + age x 1.5 mmHg” in healthy children are used as a reference (Provision of information for background question).

**CQ18-5: What is the method for assessing fluid responsiveness during the management of pediatric sepsis?**

***Answer:*** Assessments for fluid responsiveness include clinical findings (changes in pulse rate, blood pressure, temperature difference between peripheral and central skins, strength of pulsation, and capillary refill time (CRT)) and test values (e.g., lactate clearance, echocardiography findings) (Provision of information for background question).

**CQ18-6: What is the initial fluid infusion rate and volume for pediatric sepsis?**

***Answer:*** In children with sepsis not complicated by heart failure, there is a method for repeating a bolus administration 10–20 mL/kg at a time while assessing response to an initial fluid resuscitation. Meanwhile, the occurrence of clinical findings which suggest fluid overload or a blunted fluid response should serve as a reference for suspending fluid resuscitation. There is no high-quality evidence regarding the upper limits of fluid infusion rate or volume (Provision of information for background question).

**CQ18-7: Should dopamine be used as a first-line vasoactive agent in children with septic shock?**

***Answer:*** We suggest against using dopamine ad a first-line vasoactive agent in children with septic shock, and instead suggest selecting either adrenaline or noradrenaline according to hemodynamics (for adrenaline - GRADE 2D: certainty of evidence = “very low”; for noradrenaline - expert consensus: insufficient evidence).

**CQ18-8: Should vasopressin be used as a vasoactive agent in children with septic shock?**

***Answer:*** We suggest against using vasopressin as a vasoactive agent in children with septic shock (GRADE 2D: certainty of evidence = “very low”).

**CQ18-9: Should corticosteroids be administered to children with septic shock when they do not respond to initial fluid resuscitation and inotropic agents?**

***Answer:*** We suggest against the routine administration of corticosteroids in children with septic shock when they do not respond to initial fluid resuscitation and inotropic agents (GRADE 2D: certainty of evidence = “very low”).

**CQ18-10: When should blood infusions be started in hemodynamically stable children with sepsis?**

***Answer:*** We suggest starting blood transfusions with a hemoglobin level of 7.0 g/dL as a threshold for critical, hemodynamically stable children with sepsis (GRADE 2C: certainty of evidence = “low”).

**CQ18-11: Should blood purification therapy (including plasma exchange) be used to treat children with sepsis without acute kidney injury?**

***Answer:*** We suggest against using blood purification therapy to treat children with sepsis without acute kidney injury (GRADE 2D: certainty of evidence = “very low”).

**CQ18-12: Should intravenous immunoglobulin (IVIG) therapy be administered in children with sepsis?**

***Answer:*** We suggest against administering IVIG for children with sepsis (expert consensus: insufficient evidence).

**CQ18-13: Should blood glucose level be controlled tightly in children with sepsis?**

***Answer:*** We suggest against controlling blood glucose level tightly in children with sepsis (GRADE 2C: certainty of evidence = “low”).

**CQ19: Neuro intensive care**

**CQ19–1: What are the differential diseases and its testing methods in sepsis patients where brain damage is suspected due to symptoms such as disturbances in consciousness, convulsions, and paralysis?**

***Answer:*** Intracranial lesions (e.g., stroke) and potential causes (e.g., metabolic disorders) are first differentiated with the assumption that there may be compound causes for brain damage. Tests include neuroimaging, continuous electroencephalography (EEG) monitoring, biochemical tests, confirmation of the causative agent, and cerebrospinal fluid examination if necessary. Neuroimaging are performed urgently if focal neurologic signs were observed (Provision of information for background question).

CQ20: Patient- and Family-Centered Care

**CQ20-1: What are methods for providing information regarding PICS and PICS-F to patients and their families?**

***Answer:*** Providing accurate yet continuous information regarding PICS and PICS-F to patients and their families is thought to be important. There are increasing tendencies among medical staff working with the patient to provide handouts at the time of ICU admission/discharge and providing appropriate information. There are initiatives which continuously provide information, such as rounds after discharge from the ICU and the establishment of follow-up outpatients (Provision of information for background question).

**CQ20-2: Should ICU diaries be kept by patients with sepsis or those undergoing intensive care?**

***Answer:*** We suggest keeping an ICU diary for adult patients with sepsis or those undergoing intensive care (GRADE 2D: certainty of evidence = “very low”).

**CQ20-3: Should physical restraints be avoided during intensive care?**

***Answer:*** We suggest avoiding physical restraints during intensive care for adult patients with sepsis or those undergoing intensive care (GRADE 2C: certainty of evidence = “low”).

**CQ20-4-1: Should ventilation support be provided for sleep care?**

***Answer:*** We suggest adding ventilation support as part of sleep care for adult patients with sepsis or those undergoing intensive care (GRADE 2D: certainty of evidence = “very low”).

**CQ20-4-2: Should non-pharmacological sleep management (earplugs, eye-masks, music therapy) be used for sleep care?**

***Answer:*** We suggest non-pharmacological sleep management for adult patients with sepsis or those undergoing intensive care (GRADE 2D: certainty of evidence = “very low”).

**CQ20-5: Should family visiting restrictions be relaxed for the ICU?**

***Answer:*** We suggest relaxing family visiting restrictions for adult patients with sepsis or those undergoing intensive care (GRADE 2D: certainty of evidence = “very low”).

**CQ20-6: What are methods for supporting decision-making which respects the value systems and ways of thinking in the patient?**

***Answer:*** There are methods which support decision making which respects the value systems and ways of thinking of the patient through repeated multi-disciplinary conferences including patients and their families. Methods which carefully identify surrogate intention-estimating individuals (e.g., families) who estimate the intentions of the patient themselves have been proposed when the intentions of the patient are unclear. It is important to respect the intentions of the patients as well as to provide medically accurate information to patients and their families (Provision of information for background question).

CQ21: Sepsis Treatment System

**CQ21-1: What methods are there for detecting sepsis at an early stage in the general ward and ER?**

***Answer:*** Screening tools such as qSOFA and the early warning score are available as methods which can detect sepsis at an early stage in general wards and in the ER (Provision of information for background question).

**CQ21-2: What is the role of a rapid response system (RRS) which acts against changes in the condition of patients in the general ward where sepsis is suspected?**

***Answer:*** The rapid response system (RRS) is a system which detects and responds to changes in the condition of patients in the hospital, and there is an opinion where its introduction is expected to improve prognosis of patients even for sepsis (Provision of information for background question).

**CQ21-3: Where should sepsis which does not respond to initial fluid resuscitation be managed?**

***Answer:*** Sepsis which does not respond to initial fluid resuscitation should be managed in a facility where intensive care can be conducted (Good Practice Statement).

**CQ21-4: What quality indicators are there for initial treatment of sepsis?**

***Answer:*** Quality indicators for initial treatment of sepsis include implementation rates for each indicator, such as blood culture collection, lactate level measurement, early administration of antimicrobial drug, initial fluid resuscitation, and repeated intravascular volume/cardiac function assessment (Provision of information for background question).

**CQ21-5: What kinds of activities raise awareness for sepsis?**

***Answer:*** There have been events like “World Sepsis Day” for the general public and seminars for healthcare professionals held, taking the lead by the Global Sepsis Alliance and World Health Organization (WHO) (Provision of information for background question).

CQ22: Stress Ulcer Prophylaxis

**CQ22-1: Should antiulcer drugs be administered to septic patients to prevent gastrointestinal bleeding?**

***Answer:*** We suggest administering antiulcer drugs to septic patients to prevent gastrointestinal bleeding (GRADE 2B: certainty of evidence = “moderate”).

**CQ22-2: How should the suspension of antiulcer drugs be determined for septic patients?**

***Answer:*** The specific decision criteria for suspending antiulcer drugs are unclear. Clinical decision criteria include when bleeding risk factors have decreased, side effects such as pancytopenia or liver dysfunction have occurred, and when sufficient enteral nutrition was able to be administered (Provision of information for background question).

**CQ1: Definition and diagnosis of sepsis**

**CQ1-1: Definition of sepsis**

***Summary:*** According to the Third International Consensus Definitions for Sepsis and Septic Shock (Sepsis-3), sepsis is defined as “life-threatening organ dysfunction caused by a dysregulated host response to infection.” Septic shock is defined as a subset of sepsis in which the underlying circulatory and cellular/metabolic abnormalities profoundly increase the risk of mortality.

***Commentary:*** Sepsis is defined according to Sepsis-3 [19] in the J-SSCG 2020, similar to the J-SSCG-2016 [3, 4].

In 1992, the definition of sepsis (Sepsis-1) with the concept of systemic inflammatory response syndrome (SIRS) [20] was provided by the American College of Chest Physicians/Society of Critical Care Medicine Consensus Conference. The SIRS criteria is widely accepted worldwide, including Japan. According to Sepsis-1, sepsis is defined as SIRS due to infection. However, the Sepsis-1 definition had a low ability to predict the progression of organ damage and low diagnostic specificity for prognosis. Thus, the Sepsis-3 [19] definition adopted in the J-SSCG 2020 guideline focuses on the progression of organ injury in infectious diseases.

In the J-SSCG 2020, sepsis is defined as a condition in which organ dysfunction newly develops after infection. Septic shock is defined as a condition in which sepsis is accompanied by cardiovascular dysfunction, cellular damage, and severe metabolic abnormality. The definition focuses on organ dysfunction associated with infection and assesses the progression of organ dysfunction in infectious diseases that do not meet the criteria for SIRS [20].

**CQ1-2: Diagnosis of sepsis and septic shock**

***Summary:*** A diagnosis of sepsis is confirmed when the Sequential Organ Failure Assessment (SOFA) score of 2 points or more acutely increase in the presence of a clear infection or suspected infection. Patients with septic shock can be identified with a clinical construct of sepsis with persisting hypotension requiring vasopressors to maintain mBP ≥ 65 mmHg and having a serum lactate level > 2 mmol/L (18 mg/dL) despite adequate volume resuscitation. In out-of-hospital, emergency department, or general hospital ward settings, adult patients with suspected infection can be rapidly identified as more likely to have poor outcomes typical of sepsis if they have at least two of the following clinical criteria that together constitute the quick SOFA (qSOFA) score: a respiratory rate of 22 breaths/min or higher, altered consciousness, and a systolic blood pressure of ≤100 mmHg. The qSOFA criteria can be used to prompt clinicians to further investigate organ dysfunction, initiate or escalate therapy as appropriate, and to consider referral for critical care. Ultimately, an acutely increased SOFA score of 2 or more points confirms the diagnosis of sepsis. Daily routine screening for sepsis is recommended to support the early diagnosis and treatment of sepsis.

***Commentary:*** In the Japanese clinical practice guidelines for the J-SSCG 2020, the severity of sepsis is classified into two categories: sepsis and septic shock according to the Sepsis-3 definition [19]. The diagnosis and treatment of sepsis involves the progression of organ dysfunction in cases of suspected infection. The diagnosis of sepsis is based on agreement with various guidelines, such as the Sepsis-3 definition [19], the J-SSCG 2016 [3, 4], and the SSCG2016 [1, 2]. The qSOFA tool is advantageous as it enables the early evaluation of sepsis. The SOFA score [21] is used for the final diagnosis of sepsis, similar to the J-SSCG 2016 [3, 4]. On the other hand, the low sensitivity of the qSOFA tool for the diagnosis of sepsis and mortality outcome, and evaluation of its utility as an early alert system for sepsis are issues to be resolved in the future [22, 23]. Updates of the SOFA score remain an important issue considering current practices in the treatment of sepsis [24, 25].

**CQ2: Diagnosis of infection**

**Introduction**

It is important to diagnose the cause of infection in the treatment of sepsis/septic shock. Identifying pathogenic microorganisms by collecting samples is of utmost importance when diagnosing infections, and this also leads to appropriate treatment. The source of infection should be narrowed down as soon as possible using information from the medical history, physical examination findings, the results of imaging tests, etc., and culture samples should be collected appropriately along with blood cultures from the estimated infection site. Blood culture is the most important test among cultures. Many reports have described the importance of blood culture, which has a high clinical significance in identifying pathogenic microorganisms that cause bacteremia, regardless of the presence of good evidence. However, the method and timing of blood sample collection are not yet well known; thus, we decided to cover this topic in the present guideline [3, 4].

The positivity rate of blood culture tests among patients with septic shock is reported to be 69%. However, there are limits to blood cultures since the positivity rate did not increase despite performing blood culture tests for fever. There is no evidence that an improved prognosis resulted from collecting samples from sites where the possible source of infection could not be ruled out on the basis of clinical images prior to the initiation of antibacterial drugs; however, this is recommended by expert consensus in many guidelines. Describing various culture tests other than blood culture was extremely important in the present guideline as well.

Antibacterial drugs are selected without waiting for blood culture results in clinical practice; however, the practice of referring to Gram stain findings when selecting antibacterial drugs is widespread, and is valid to some extent from the perspective of pathophysiology [3, 4]. Describing the benefit of Gram staining was extremely important in the present guideline as well.

**Fig. 1:**
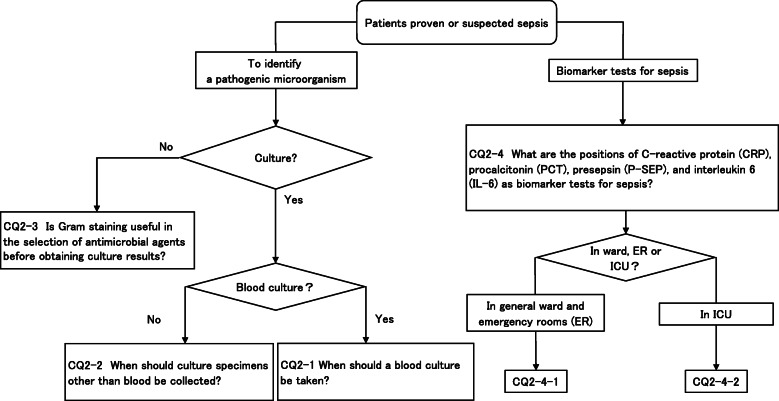
CQ2: Diagnosis of infection (clinical flow)

Furthermore, it is important to confirm the effectiveness of these biomarkers for the diagnosis of infection. Four biomarkers (C-reactive protein, procalcitonin, presepsin, and interleukin 6) are currently used to assist in the diagnosis of sepsis. The evaluation of non-severely ill patients, such as emergency outpatients and those in general wards, differs from that of severely ill patients, such as those admitted to the ICU. Thus, these have been discussed separately. Clinical flow of these CQs is shown in Fig. 1.

**CQ2-1: When should a blood culture be taken?**

***Answer:*** Take two or more sets before administering the antibacterial drug (Good Practice Statement).

***Rationale***

Bacteremia is generally caused by infections such as endocarditis, central venous catheter infection, pneumonia, abscesses, osteomyelitis, intraperitoneal infection, and urinary tract infections, resulting in a high mortality rate [26]. Various rapid diagnostic methods have been developed [27]; however, at present, blood cultures are the standard test method in the diagnosis of bacteremia. There is no high-quality evidence regarding the timing of blood culture collection, and we have not made a clear recommendation in this CQ.

It has been recommended that sepsis should be suspected in the presence of symptoms indicative of bacteremia (e.g., fever, shivering, hypotension, and tachypnea), hypothermia with an unknown cause, hypotension, altered state of consciousness, increased/decreased white blood cell count, and metabolic acidosis, as well as respiratory failure, acute kidney injury (AKI), and acute liver dysfunction in immunodeficient patients. In these cases, it is recommended that two or more sets of blood cultures be collected as rapidly as possible when the patient has a temperature greater than 38.5 °C or is shivering [28]. However, some reports have indicated that blood cultures do not need to be obtained exclusively for the reasons of fever or an increased white blood cell count, which indicate a low possibility of sepsis [29].

As a general rule, it is important to collect sets before administering antibacterial drugs, while keeping in mind not to delay the initiation of antibacterial drug treatment. This is because the sensitivity of detection often decreases after drug administration, and the bacteria may not be detected [30]. During antibacterial therapy, samples should be collected near the trough of the antibacterial drug concentration, or in other words, immediately before the administration of the next round of antibacterial drugs. Furthermore, samples should be collected again when the patient responds poorly to treatment, and the anti-bacterial drug is changed.

With regard to the amount of sample to collect, it is known that larger collection volumes increase the likelihood of bacterial identification [31]. However, increasing the collection volume can increase the risk of iatrogenic anemia; thus, it is generally recommended that a collection volume of 20–30 mL be used per set. In Japan, the commonly used blood culture bottle often has a capacity of 10 mL, so 20 mL is typical for a single set. Cheruvanky et al. reported that from a clinical economy perspective, 20 mL was better than 30 mL [32].

Reports regarding the number of sets to collect indicated that just one set was characterized by negative results due to a lower sensitivity and an inability to exclude contamination, indicating that two sets (three if possible) were ideal [31, 33]. In reality, it has been said that the blood culture positivity rate is only 5–13%, and that 20–56% of samples are contaminated [34]. A report has indicated that increasing the number of sets would increase the sensitivity (approximately 80, 89, and 98% for one, two, and three sets, respectively) [30]. No increases in sensitivity was seen when four or more sets were collected, and this should be avoided, as it increases the burden on the patient.

Appropriate skin disinfection and the collection of multiple sets are necessary to reduce the likelihood of contamination. It is unclear which among 1% chlorhexidine gluconate, povidone iodine, and 70% alcohol is the optimal antiseptic suitable for skin disinfection; however, there is no doubt regarding the importance of using these agents to ensure an accurate aseptic procedure [35].

**CQ2-2: When should culture specimens other than blood be collected?**

***Answer:*** Each cultured specimen other than blood should be collected as needed prior to the administration of antibacterial drugs (Good Practice Statement).

***Rationale***

Blood cultures are a standard diagnostic tool for diagnosing bloodstream infections and bacteremia. Patients with septic shock have been reported to have a blood culture positivity rate of 69%; however, there are limits to blood cultures since the presence of a fever alone does not result in a high positivity rate even with blood culture tests [29]. Identifying infected organs and causative microorganisms is extremely difficult, particularly in cases of sepsis caused by urinary tract infections, pneumonia, and meningitis. Despite showing no evidence of improved prognosis, many guidelines recommend that specimens be collected from areas where the source of infection cannot be ruled out based on clinical findings prior to the administration of anti-bacterial drugs as much as possible [36–38].

The diagnosis and treatment of pneumonia can vary depending on the underlying pathology, although diagnoses via sputum culture can be useful. However, as sputum samples have an increased risk of contamination in evaluating the upper respiratory tract, care should be taken in interpreting its test results when they are inconsistent with those of pleural effusion and blood culture. Critically ill patients who have undergone tracheal intubation for mechanical ventilation should have their endotracheal sputum collected and quantitatively cultured; if the bacterial count is found to be over 10 [4] CFU/mL (sputum prior to antibacterial drug administration, sensitivity of 90%, specificity of 77%), then a high possibility of infection with causative bacteria is suspected [39]. Furthermore, a report on the diagnosis of ventilator-associated pneumonia indicated that the probability of non-isolation of causative bacteria was 94% when bacteria were not isolated from endotracheal sputum [40]. Furthermore, searching for microorganisms in bronchoalveolar lavage fluid is also important for deciding the treatment policy for acute respiratory distress syndrome (ARDS) with pneumonia as either a cause or complication, and this is effective for excluding pneumocystis pneumonia or pulmonary mycosis when the immune system of the patient is weakened [41].

Most urinary tract infections are of the ascending type, caused by indigenous bacteria in the colon, and a urine culture test should be performed prior to administering antibacterial drugs in order to isolate the causative bacteria and investigate drug sensitivity. Antibacterial drugs should be administered in recurrent or refractory diseases, and urine culture tests should be performed between drug withdrawals lasting 2–3 days [37, 42].

No RCTs have confirmed the efficacy of blood/cerebrospinal fluid cultures for the diagnosis of bacterial meningitis. However, it is ideal to collect cerebrospinal fluid in all patients with suspected meningitis due to the presence of headaches and altered consciousness so long as cerebral hernias are not suspected based on cranial computed tomography (CT) scans or clinical findings, and lumbar punctures are not contraindicated [38]. However, antibacterial drug administration should be prioritized in cases where cerebrospinal fluid collection takes time. The cerebrospinal fluid culture positivity rate is 70–80% without treatment and less than 50% following antimicrobial therapy [43]. Regarding the cerebrospinal fluid positivity rate for bacterial meningitis, an increased collection volume and centrifugation speed (1500–2500×g, 15 min) increases the detection rate [44].

**CQ2-3: Is Gram staining useful in the selection of antimicrobial agents before obtaining culture results?**

***Answer:*** We suggest referencing Gram staining findings of the culture specimen when selecting an antibacterial drug to use for empirical treatment (expert consensus: insufficient evidence).

***Rationale***

The desired effect of Gram staining may be helpful in selecting antibacterial drugs for use in empiric therapy. The 2019 Infectious Diseases Society of America (IDSA) guidelines for community-acquired pneumonia [45] stated that pre-treatment sputum Gram staining and culture should be performed. This should be done when there is severe pneumonia, empiric therapy was commenced for methicillin-resistant *Staphylococcus aureus* or *Pseudomonas aeruginosa*, or when oral antibacterial drugs were administered during hospitalization or within 90 days.

The 2015 Japanese Association for Infectious Disease/Japanese Society of Chemotherapy infection treatment guideline [37] for urinary tract infections and male genital infections have shown that urinary Gram staining was deemed useful in estimating the causative organism in cases of catheter-related urinary tract infections. The selection of antibacterial drugs based on Gram stain findings leads to suitable empiric therapy and often leads to definitive therapy. Furthermore, Gram staining has been reported to evaluate bacterial meningitis in that the results can be obtained in a simple and prompt manner, with a sensitivity of 50–90%, specificity of 60–90%, and minimum detection sensitivity of 105 cfu/mL [12].

Selecting antibacterial drugs based only on the results of this test alone has an inherent risk of selecting inappropriate narrow-range antimicrobial drugs regardless of the severity of the patient’s condition. Sensitivity and specificity are also influenced by the tester, and there is a risk of selecting inappropriate antibacterial drugs. The balance between benefits and harms are thought to vary according to the patient’s condition. Gram staining can be performed in a simple yet prompt manner and is also inexpensive; thus, it is thought that the benefits of performing it while understanding its utility and limits outweigh its harms.

Meanwhile, its undesirable effects are as follows. Selecting the antibacterial drug based solely on these test results has the risk of selecting inappropriate narrow-range antimicrobial drugs regardless of the severity of the patient’s condition. Sensitivity and specificity are also influenced by the tester, and there is a risk of selecting inappropriate antibacterial drugs (there is the possibility of the tester using inappropriate testing methods, or the possibility of arriving at false positive/false negative results due to insufficient testing experience). The 2019 IDSA guidelines for community-acquired pneumonia [45] also recommended against Gram staining for sputum obtained after treatment due to the fact that the bacterial strain results could change due to the administration of antibacterial drugs.

Based on the above, it is thought that the balance between benefits and harms vary according to the patient’s condition. However, Gram staining can be performed in a simple and prompt manner and is also inexpensive; thus, it is thought that the benefits of performing Gram staining while understanding its utility and limits outweigh its harms.

**CQ2–4-1: What are the positions of C-reactive protein (CRP), procalcitonin (PCT), presepsin (P-SEP), and interleukin 6 (IL-6) as biomarker tests for sepsis diagnosis in general ward and emergency rooms (ER)?**

***Answer:*** Sensitivity and specificity in biomarker tests when sepsis was suspected in general ward and ER visits were as follows: CRP, 59, 79%; PCT, 74, 81%; P-SEP, 75, 74%; IL-6, 78, 78%. As such, sepsis diagnosis with biomarkers alone is generally thought to be difficult, and its use should be seen as supplemental to any observations of general conditions (Provision of information for background question).

***Rationale***

*How CQ2–4-1 and CQ2–4-2 became BQs*

CQ2–4-1 and CQ2–4-2 were initially grade-based CQs, as follows: “Which among C-reactive protein (CRP), procalcitonin (PCT), presepsin (P-SEP), and interleukin 6 (IL-6) should be used as a biomarker for infectious disease diagnosis?” However, the target infectious diseases varied extremely; thus, in light of the characteristics of this guideline, we focused on sepsis, which is a critical condition that negatively affects general physiological conditions. A comprehensive literature search was conducted as part of a systematic review, with a focus on the diagnostic accuracy of dividing the extracted articles into “general ward or emergency rooms (ERs)” (CQ2–4-1) or “ICUs” (CQ2–4-2). A total of 11 articles were included in the category “general ward or ER”, and the number of papers assessed via a meta-analysis on each biomarker were as follows: CRP, eight articles [46–53]; PCT, 11 articles [1–4, 19–25]; P-SEP, four articles [51, 52, 54, 55]; IL-6, four articles [46, 48, 49, 56]. Furthermore, a total of nine articles were included in the category “ICUs”, and the number of papers assessed via a meta-analysis on each biomarker were as follows: CRP, seven articles [57–63]; PCT, nine articles [57–65]; P-SEP, four articles [57, 61, 62, 64]; and IL-6, six articles [58–61, 63, 65].

An evidence profile and EtD were summarized based on these results, and the following responses were presented: “The diagnostic accuracies of PCT, P-SEP, and IL-6 are thought to be relatively high; however, we do not recommend the use of each biomarker, including CRP, in the diagnosis of sepsis, because this antagonizes the balance of effects against important outcomes among patients and their families” for “general wards and ER” (CQ2–4-1), and “We suggest that the levels of CRP, PCT, and P-SEP be measured as biomarkers for the diagnosis of sepsis in the ICU. We do not recommend the measurement of IL-6 levels” for “ICUs” (CQ2–4-2). A committee vote was then held.

The results of two rounds of voting did not yield any consensus for either CQ, and for CQ2–4-1, committee members indicated that “the role of biomarkers alone is ultimately supplemental for the diagnosis of sepsis but not infectious diseases”, and “this may be interpreted as indicating that the levels of CRP, PCT, and P-SEP, which have until now been widely measured on a regular basis, are no longer necessary, with a concern that biomarker measurements may no longer be conducted”. Furthermore, for CQ2–4-2, there were opinions that “suggesting the usefulness of CRP at the same level as PCT and P-SEP, and suggesting against only IL-6, were inappropriate”. The results of repeated discussions within the committee ultimately resulted in CQ2–4-1 and CQ2–4-2 being handled as BQs.

Explanation: The following explanation was provided using the EP (Tables 4, 5, 6 and 7) created as a result of systematic review and the grade recommendation process.

**Table 4:**
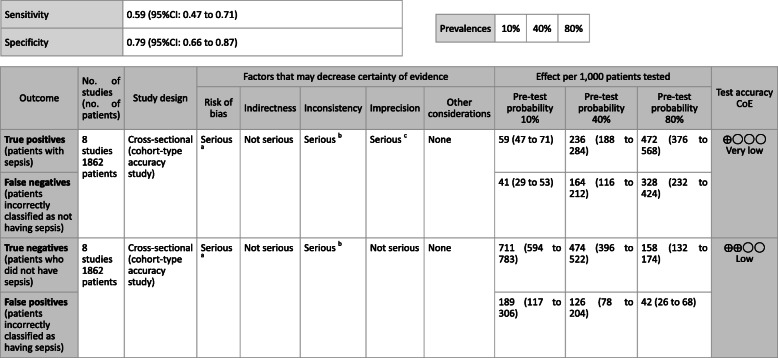
Evidence profile (CRP in general wards or the ER)

^a^ Observational studies only, and the biases of 8 studies were high against markers (indices)

^b^ *Q* level, *p*-value < 0.05: heterogeneity present 95%CI overlap: insufficient *I*^2^ > 90

^c^ Wide confidence interval and large number of false negatives, particularly when the prevalence was high

**Table 5:**
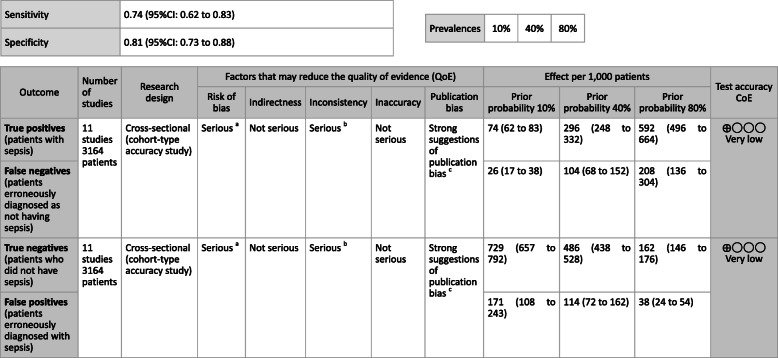
Evidence profile (PCT in general wards or the ER)

^a^Observational studies only, and the biases of 11 studies were high against markers (indices)

^b^*Q* level, *p*-value < 0.05: heterogeneity present 95%CI overlap: insufficient *I*^2^ > 75

^c^Asymmetric with Deeks’ funnel plot asymmetry test (*p* = 0.01)

**Table 6:**
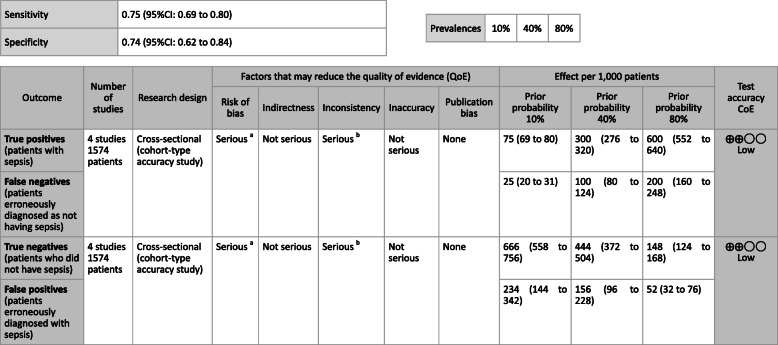
Evidence profile (P-SEP in general wards or the ER)

^a^ Observational studies only, and the biases of 11 studies were high against markers (indices)

^b^ *Q* level, high *I*^2^, *p*-value < 0.05: heterogeneity present

**Table 7:**
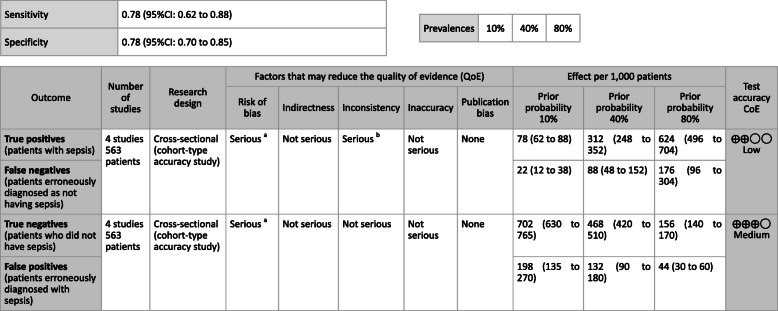
Evidence profile (IL-6 in general wards or the ER)

^a^ Observational studies only, and the biases of 4 studies were high against markers (indices)

^b^ *Q* level, *p*-value < 0.05: heterogeneity present 95%CI overlap: insufficient: *I*^2^ = 91

The results of the systematic review for this CQ in terms of the respective sensitivities and specificities of biomarker tests when sepsis was suspected in the general ward or ER were as follows: CRP, 59, 79%; PCT, 74, 81%; P-SEP, 75, 74%; and IL-6, 78, 78%. In actual clinical settings, there are facilities that can only measure CRP levels as well as other facilities that can measure multiple biomarkers. For these reasons, it is worth noting that CRP has an inferior sensitivity to those of PCT, P-SEP, and IL-6 when used as a supplement for the suspicion of sepsis among patients. Based on the above results of systematic review, in facilities in which the levels of the biomarkers PCT, P-SEP, and IL-6 can be measured in addition to CRP, they can be used as a reference to aid the suspicion of sepsis. In these ways, these biomarkers have the potential to bring about significant results in some patients; however, care must be taken as the interpretation of these measurements differ under various conditions depending on patients’ conditions, time of blood sample collection, and location. For these reasons, we decided to specifically display the sensitivities and specificities obtained in the meta-analysis and to leave this to the discretion of the readers in their various respective circumstances.

**CQ2–4-2: What are the positions of C-reactive protein (CRP), procalcitonin (PCT), presepsin (P-SEP), and interleukin-6 (IL-6) as biomarker tests for sepsis diagnosis in the intensive care unit?**

***Answer:*** Sensitivity and specificity in biomarker tests when sepsis was suspected in the ICU were as follows: CRP, 74, 70%; P-SEP, 82, 73%; IL-6, 72, 76%. As such, sepsis diagnosis with biomarkers alone is generally thought to be difficult, and its use should be supplemental to any observations of general conditions (Provision of information for background question).

***Rationale***

The background and recommendation making process was described in the rationale for CQ2–4-1. The following rationale was created in reference to the evidence profile (Tables 8, 9, 10 and 11) created as a result of an systematic review and the grade recommendation process.

**Table 8:**
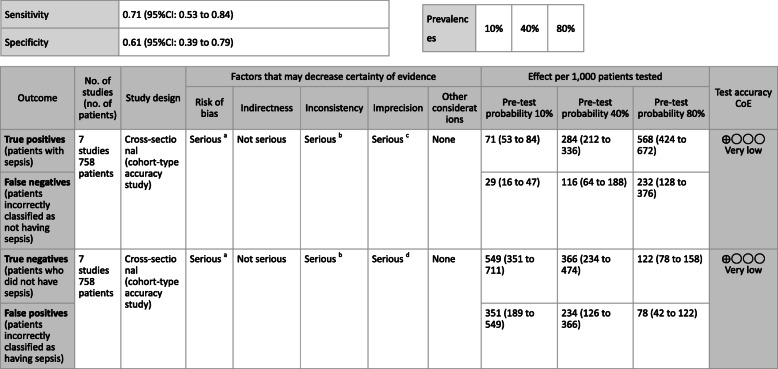
Evidence profile (CRP in the ICU)

^a^ Observational studies only, and the biases of 7 studies were high against markers (indices)

^b^ *Q* level, *p*-value < 0.05: heterogeneity present 95%CI overlap: Insufficient *I*^2^ > 85

^c^ Wide confidence interval and large number of false negatives, particularly when the prevalence was high

^d^ Wide confidence interval, and a large number of false positives

**Table 9:**
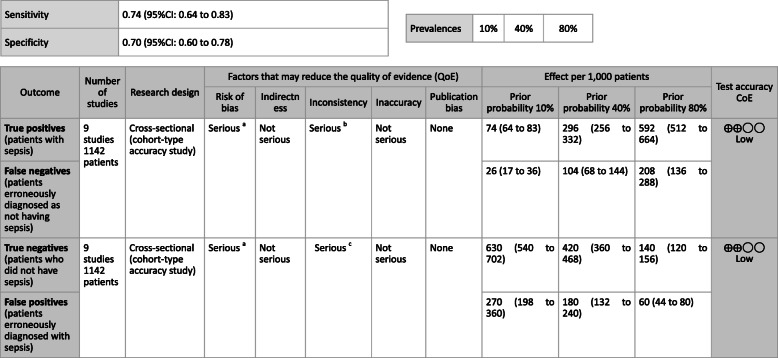
Evidence profile (PCT in the ICU)

^a^ Observational studies only, and the biases of 9 studies were high against markers (indices)

^b^ *Q* level, *p*-value < 0.05: heterogeneity present 95%CI overlap: insufficient *I*^2^ = 86

^c^ *Q* level, *p*-value < 0.05: heterogeneity present 95%CI overlap: insufficient *I*^2^ = 76

**Table 10:**
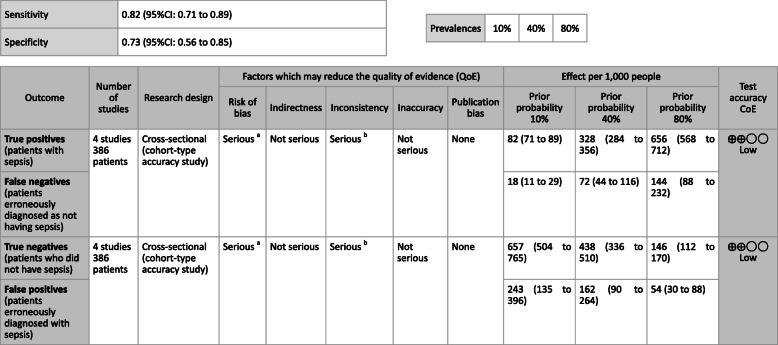
Evidence profile (P-SEP in the ICU)

^a^ Observational studies only, and the biases of 4 studies were high against markers (indices)

^b^ *Q* level, *p*-value < 0.05: heterogeneity present 95%CI overlap: sufficient

**Table 11:**
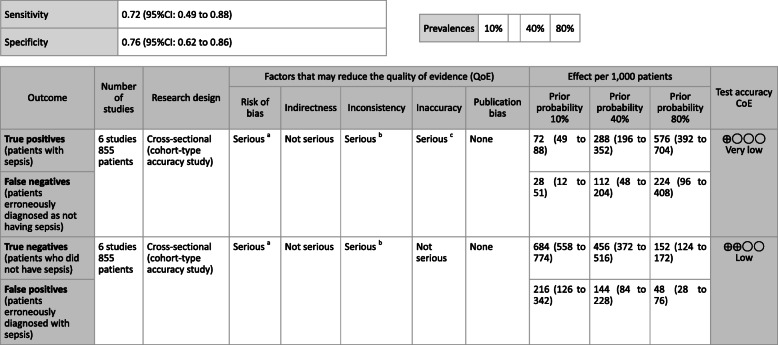
Evidence profile (IL-6 in the ICU)

^a^ Observational studies only, and the biases of 6 studies were high against markers (indices)

^b^ *Q* level, *p*-value < 0.05: heterogeneity present, 95%CI overlap: insufficient *I*^2^ > 90

^c^ Wide confidence interval and large number of false negatives, particularly when the prevalence was high

The results of the systematic review for this CQ showed that the respective sensitivities and specificities of the biomarker tests when sepsis was suspected in the ICU were as follows: CRP, 71, 61%; PCT, 74, 70%; P-SEP, 82, 73%; and IL-6, 72, 76%. Based on these results, it cannot be determined whether the sensitivities and specificities were sufficiently high or low.

The biomarker tests suggested significant results for the diagnosis of sepsis in individual articles assessed in the systematic review [57–65]. Meanwhile, care must be taken because the results of biomarker tests can change or can be influenced by the bacterial type or location of the infection depending on various factors such as patient status or time of blood sample collection. For these reasons, we have specifically displayed the sensitivities and specificities obtained in the meta-analyses and have left this to the discretion of the individual readers in their respective circumstances.

**CQ3: Source control**

**Introduction**

The importance of initiating treatment for sepsis at an early stage is widely accepted. Among early treatment modalities, controlling the source of infection is one that exhibits its effectiveness by cutting off and “controlling” the “infection source” that is at the root of sepsis, and forms the basis of initial treatment. Diagnostic imaging is essential to promptly control the source of infection. Therefore, two CQs on diagnostic imaging were first incorporated, after which seven CQs on controlling the source of infection were incorporated.

The first CQ on diagnostic imaging that was incorporated was “CQ3-1: Should imaging tests be performed in patients with suspected sepsis to identify the source of infection?” Diagnostic imaging modalities for identifying the source of infection include simple radiography, ultrasonography, CT scans, and magnetic resonance imaging (MRI) scans, and highly useful test methods vary by site. The explanations in this CQ include a table on diagnostic imaging methods thought to be specific for each organ/illness in order to be of use in actual clinical practice.

The second CQ on diagnostic imaging is that regarding full-body contrast CT scans: “CQ3-2: Should full-body contrast-enhanced CT tests be performed at an early stage in patients with sepsis and an unknown source of infection?” Identifying the source of infection early when the source is unknown is essential for formulating a treatment policy. Performing CT scans, which are diagnostic imaging modalities that have seen widespread use in Japan, are important for local diagnosis as well as for determining the severity of the source of infection. Thus, this was taken up as a CQ.

Subsequent discussions on the selection of CQs regarding the control of the source of infection resulted in the following six sources of infection that were thought to be of particular importance and set as CQs: 1) intraperitoneal infection, 2) infectious pancreatic necrosis, 3) acute pyelonephritis secondary to ureteral obstruction, 4) necrotic soft tissue infection, 5) catheter-related bloodstream infections, and 6) empyema.

**Fig. 2:**
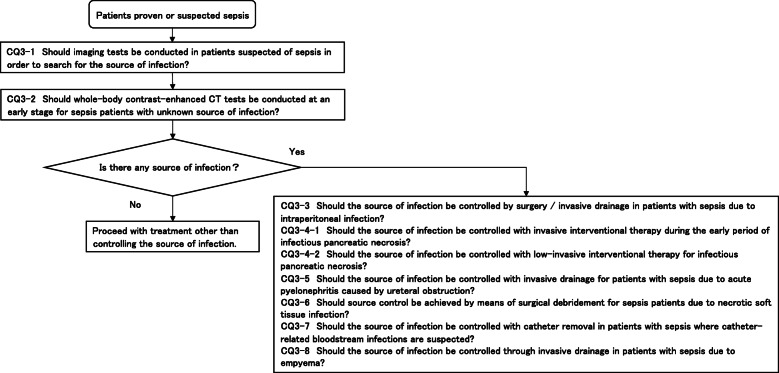
CQ3: Source control (clinical flow)

It is universal knowledge that the basic concept underlying the control of the source of infection is to do so “promptly” and “appropriately.” The best methods are those that are minimally invasive, have a low incidence of complications, and have sufficient expected effects. Furthermore, the source of infection should generally be controlled promptly; however, we also suggest that elective interventions may be considered for patients with infectious pancreatic necrosis. Clinical flow of these CQs is shown in Fig. 2.

**CQ3-1: Should imaging tests be conducted in patients suspected of sepsis in order to search for the source of infection?**

***Answer:*** Imaging tests should be conducted when the source of infection is unclear in order to search for the source of infection (Good Practice Statement).

***Rationale***

Controlling the source of infection at an early stage is an important treatment strategy that is linked to an improved outcome among patients with sepsis. For this reason, it is important to assess early whether there is a source of infection that needs to be controlled among patients with suspected sepsis, and imaging tests need to be considered for this procedure. Imaging tests useful for identifying the source of infection include plain radiography, ultrasonography, CT scans, and MRI scans. The most effective testing modality varies with the site of suspected infection. Diagnostic imaging modalities considered characteristic of each organ/disease are shown in Table 12.

**Table 12:** Diseases that require control of the source of infection and imaging tests

		Main tests expected			
Region		Simple X-ray	Ultrasonography	CT scan	MRI scan
Head and neck	Brain abscess / meningoencephalitis			○(contrast-enhanced imaging)	○(contrast-enhanced imaging), contrast enhanced fluid-attenuated inversion recovery (FLAIR) (for encephalitis)
Chest	Cervical abscess (descending mediastinitis)		○	○(contrast-enhanced imaging)	
	Empyema	○	○	○(contrast-enhanced imaging)	
	Infective endocarditis		○^a^	○(contrast-enhanced imaging)	
Abdomen	Intestinal perforation / peritonitis	○	○	○(contrast-enhanced imaging)	
	Cholecystitis / cholangitis		○	○(contrast-enhanced imaging)	○(MRI/MRCP)
	Obstructive urinary tract infection	○	○	○	
Other	Necrotic soft tissue infections			○(contrast-enhanced imaging)	

^a^Transesophageal echocardiography other than the transthoracic wall variant is more accurate in diagnosing infective endocarditis

1. Head and neck

Cerebral abscess: CT scans are easier to conduct in an emergency relative to MRI scans; thus, the former is often prioritized in its implementation. Contrast-enhanced MRI scans are the most recommended imaging modality because of their ability to detect the spread of inflammation to the capsule or tissue surrounding the abscess [66].

Cervical abscess (descending mediastinitis): Cervical abscesses near the surface of the body can be detected via ultrasonography; however, there are limits to the detection of deep cervical abscesses, and CT scans are considered effective. Contrast-enhanced CT scans are recommended because they can clearly differentiate between fluid retention due to infection and structures such as blood vessels [67].

(2) Chest

Empyema: Plain X-ray imaging and ultrasonography are first-line evaluation modalities. Contrast-enhanced CT scans are effective for controlling the source of infection or as an indicator for assessing the course of treatment when an empyema is suspected.

Infectious endocarditis: One of the two major categories in the diagnostic criteria for infectious endocarditis (the Duke diagnostic criteria) [68] is based on the findings of echocardiography, and transthoracic echocardiography should be implemented as a first-line evaluation modality for all patients when infectious endocarditis is suspected. The accuracy of transesophageal echocardiography for the diagnosis of infectious endocarditis is superior relative to the transthoracic variation; therefore, we recommend that additional transesophageal echocardiography should be performed when necessary [69].

(3) Abdomen

Intestinal perforation/peritonitis: Plain X-ray imaging and ultrasonography should be performed first. CT scans should be subsequently performed when further assessments are needed. We recommend that contrast-enhanced CT scans be performed when detailed assessments of phenomena such as the presence of ischemia in organs or the intestinal tract needs to be determined [70].

Cholecystitis/cholangitis: Ultrasonography and CT scans are the most recommended evaluation modalities. Contrast-enhanced CT scans can be used to identify important findings. We also recommend MRI/magnetic resonance cholangiopancreatography as alternative imaging modalities [71].

Obstructive urinary tract infection: Ultrasonography should be performed as a first-line assessment modality. We recommend that CT scans should be performed to carefully evaluate the causes of obstruction if the clinical findings are suggestive of obstructive urinary tract infection [72].

(4) Others

Necrotizing soft tissue infection: A contrast-enhanced CT scan should be performed because of its ability to detect the swelling and fluid retention in soft tissue. However, a definitive diagnosis of necrotizing fasciitis cannot be made with a contrast-enhanced CT scan alone; such a diagnosis requires surgical examination of the subcutaneous tissue/fascia and direct observation of the fascia/muscle [73].

Imaging modalities are beneficial for the selection of the optimal treatment method. Meanwhile, the risk of exposure to X-rays or utilization of contrast agents, particularly the risk of sudden changes while transferring critically ill patients to the examination room, must be recognized.

**CQ3-2: Should whole-body contrast-enhanced CT tests be conducted at an early stage for sepsis patients with unknown source of infection?**

***Answer:*** We suggest conducting whole-body contrast-enhanced CT tests as soon as possible for sepsis patients with unknown source of infection (expert consensus: insufficient evidence).

***Rationale***

Appropriate therapeutic interventions at an early stage against the source of infection are recommended for sepsis [74]. Searching for the source of infection at an early stage when it is unknown is also essential to formulating a treatment plan. The use of CT scans, which are widespread diagnostic imaging modalities in Japan, is essential for local diagnosis and determining the severity of the source of infection.

The results of a systematic review showed that there were no RCTs conforming to the PICO criteria that were conducted on patients who satisfied the sepsis diagnostic criteria or who were undergoing intensive care.

It is possible that improvements in general conditions are not achieved even with standard therapy in cases of sepsis in which the sources of infection are unclear. Therefore, efforts must be made to perform whole-body contrast-enhanced CT scans at an early stage and clarify the source of infection to improve vital prognosis, and it is thought that a desirable therapeutic intervention for the patient could be possible. It is feared that patients with complications of shock will have experience destabilization of hemodynamics accompanied by moving them. Furthermore, it is feared that the use of contrast agents will result in the onset of allergies to iodine or contrast agent-induced nephropathy.

At the very least, it is possible that the source of infection could be clarified by performing whole-body contrast-enhanced CT scans. It is thought that the benefits outweigh the harms, such as destabilized hemodynamics accompanied by moving, contrast agent-induced nephropathy, and allergies to iodine.

Japan has the highest number of CT scanning devices per capita worldwide, and there are many facilities in which sepsis can be treated and this is thought to be possible.

Contrast-enhanced CT scans are not necessarily useful for all organs when searching for the source of infection. In some cases, specific inspection methods should be prioritized for each organ, and further investigations are necessary on the usefulness of contrast-enhanced CT scans according to organs involved in sepsis with an unknown source of infection.

**CQ3-3: Should the source of infection be controlled by surgery/invasive drainage in patients with sepsis due to intraperitoneal infection?**

***Answer:*** We suggest controlling the source of infection as soon as possible with surgery/invasive drainage (including abscess drainage, biliary tract/gallbladder drainage) for patients with sepsis due to intraperitoneal infection (expert consensus: insufficient evidence).

***Rationale***

The potential benefits of rapidly controlling the source of infection among patients is considered large in cases of sepsis due to intraperitoneal infection such as generalized peritonitis due to the perforation of the lower gastrointestinal tract, where the possibility of improvements with only typical antibacterial drug treatment without controlling the source of infection is extremely low. Possible harms that can occur in actual clinical practice include bleeding, organ damage, deteriorating general conditions due to biological invasion, and infection. There were no RCTs conforming to the PICO criteria, and the balance of effects is unclear. It is thought that the benefits outweigh the harms, even when comparing the advantages obtained via surgical intervention by way of drainage (including abscess and biliary drainage) for sepsis due to intraperitoneal infection, and the harms of bleeding, organ damage, deteriorating general conditions due to biological invasion, and infection due to surgery or drainage.

**CQ3-4-1: Should the source of infection be controlled with invasive interventional therapy during the early period of infectious pancreatic necrosis?**

***Answer:*** We suggest against controlling the source of infection with invasive interventional therapy during the early period of infectious pancreatic necrosis (GRADE 2C: certainty of evidence = “low”).

Answer: We suggest against controlling the source of infection with invasive interventional therapy during the early period of infectious pancreatic necrosis (GRADE 2C: certainty of evidence = “low”).

***Rationale***

Necrotic tissue is a cause of infection, and early intervention is a general principle underlying treatment. However, pancreatic necrosis does not fall under this general principle of early intervention. Furthermore, RCTs that incorporated minimally invasive and effective methods to control the sources of infection have been conducted; thus, the timing of intervention for this disease is an important CQ.

The results of a systematic review confirmed a single RCT conforming to the PICO criteria with a small sample size (early intervention, 25 patients; late intervention, 11 patients). The mortality rates were 56 and 27% for the early and late intervention groups, respectively. The estimated value of effects yielded a risk difference (RD) of 286 more per 1000 (95% confidence interval [CI]: 71 fewer to 1000 more), and no desired effects related to vital outcomes were observed in the early intervention group compared to the late intervention group [75]. No investigations have been conducted on adverse effects or medical costs, and the desired effects in the early intervention group are unknown. The mortality rate of the late intervention group was lower than that of the early intervention group; thus, it is likely that the benefits of late intervention outweigh its harms.

**CQ3-4-2: Should the source of infection be controlled with low-invasive interventional therapy for infectious pancreatic necrosis?**

***Answer:*** We recommend controlling the source of infection with less invasive interventional therapy for patients with sepsis caused by infectious pancreatic necrosis (GRADE 2B: certainty of evidence = “moderate”).

***Rationale***

Infectious pancreatic necrosis is a condition that requires the removal of the source of infection with some types of interventional treatment. A number of treatment strategies have been reported in recent years, such as (1) surgical drainage, (2) endoscopic drainage, (3) percutaneous drainage (mainly via the retroperitoneal route), and (4) the step-up approach, which becomes incrementally more invasive according to the treatment effect. The relationship between treatment invasiveness and treatment effect is therefore an important CQ.

The results of systematic reviews confirmed the existence of two RCTs (less invasive methods, 94 patients; invasive methods, 92 patients) [76, 77]. The data used in these two RCTs showed that the onset of complications when the source of infection was controlled with less invasive methods (drainage methods) was lower than that when invasive methods were used RD of 187 fewer per 1000 (95%CI: 305 fewer to 55 more). Based on the above results, the desired effects of less invasive interventional treatment are considered moderate.

In terms of mortality outcomes, researchers investigated the three timings of short-term (6 months), medium-term (3 years), and long-term (10 years) outcomes. It was possible to pool data from the 2 RCTs (less invasive methods, 94 patients; invasive methods, 92 patients) using only mortality within six months and the number of effects of mortality outcomes yielded a RD of 40 more per 1000 (95%CI: 48 fewer to 211 more). Furthermore, the number of effects for the length of stay in the ICU and in-hospital stay each yielded a mean difference (MD) of 19.74 days longer (95%CI: 20.84 shorter to 60.31 longer) and 7.76 days shorter (95%CI: 27.86 shorter to 12.34 longer), respectively. The number of effects varied widely and the undesired effects of controlling the source of infection with less invasive interventional methods when compared to invasive interventional methods were unclear.

The invasiveness of procedures for controlling the source of infection, their timing, the range over which debridement is to be performed, and the necessity of repeated debridement needs to be investigated alongside the general conditions of patients, and this is not recommended for standard treatment among all cases.

**CQ3-5: Should the source of infection be controlled with invasive drainage for patients with sepsis due to acute pyelonephritis caused by ureteral obstruction?**

***Answer:*** We suggest controlling the source of infection as soon as possible with transurethral ureteral stent implantation or percutaneous nephrostomy in patients with sepsis due to acute pyelonephritis caused by ureteral obstruction (expert consensus: insufficient evidence).

***Rationale***

The results of a systematic review showed that there were no RCTs that conformed to the PICO criteria. Patients with acute pyelonephritis secondary to ureteral obstruction are unlikely to recover from sepsis unless transurethral stenting or percutaneous nephrostomy is performed to eliminate the cause. Therefore, it is thought that the potential benefits of rapidly controlling the source of infection are high among these patients. There was no significant difference between patients who underwent percutaneous renal fistula construction and transurethral ureteral stenting, which are methods of providing emergency relief for ureteral obstruction. Complications associated with invasive procedures include bleeding, organ damage, and the spread of infection to the retroperitoneal space (cavity). However, it is thought that the benefits outweigh the harms, even when considering complications or the burden of transferring a patient to a facility in which rapid specialized treatment modalities (transurethral ureteral stenting or percutaneous renal fistula) can be performed when such treatments cannot be offered.

**CQ3-6: Should source control be achieved by means of surgical debridement for sepsis patients due to necrotic soft tissue infection?**

***Answer:*** We suggest controlling the source of infection as soon as possible by means of surgical debridement for sepsis patients due to necrotic soft tissue infection (expert consensus: insufficient evidence).

***Rationale***

Necrotic soft tissue infection is a condition that requires early surgical control of the source of infection, and the need for debridement is difficult to determine with imaging tests. Performing surgical debridement of the necrotic tissue (soft tissue) that causes sepsis can reliably control the source of infection, and desirable effects such as an increased survival rate and a shortened therapeutic duration can be obtained. Meanwhile, most patients require surgery under general anesthesia, and there is concern about further anesthesia-induced destabilization due to unstable hemodynamics, as well as effects on hemodynamics due to hemorrhaging, and in some patients, multiple sessions of surgical debridement are necessary. There were no RCTs that conformed to the PICO criteria, and the balance of effects was unclear. The benefits of surgically removing the source of infection are thought to outweigh the harms, even when the harm caused by surgical treatment is compared.

**CQ3-7: Should the source of infection be controlled with catheter removal in patients with sepsis where catheter-related bloodstream infections are suspected?**

***Answer:*** We suggest controlling the source of infection as soon as possible with catheter removal in patients with sepsis where catheter-related bloodstream infections are suspected (expert consensus: insufficient evidence).

***Rationale***

Vascular catheter infections may not be improved with normal antibacterial drug treatment alone without controlling the source of infection. There have been cases in which the prognosis or mortality rate worsened if the cause was not resolved; therefore, it is thought that promptly controlling the source of infection has a high potential of yielding benefits among patients. This desirable effect is influenced by the accuracy of diagnosis of catheter infections. Patients who require vascular catheters do not only require the removal of the vascular catheter but also its re-insertion when controlling the source of infection. This may yield complications associated with vascular catheter insertion and affect the risks associated with re-insertion. Furthermore, frequent route exchanges increase costs and work burden. There are no RCTs that conform to the PICO criteria, and the balance of effects is unknown. It is thought in the case of vascular catheter infection that the benefits obtained by controlling the source of infection (catheter removal) outweigh the harms of complications relating to vascular catheter removal.

**CQ3-8: Should the source of infection be controlled through invasive drainage in patients with sepsis due to empyema?**

***Answer:*** We suggest controlling the source of infection as soon as possible with percutaneous thoracic drainage or surgical intervention in patients with sepsis due to empyema (expert consensus: insufficient evidence).

***Rationale***

The results of a systematic review showed that there were no RCTs that conform to the PICO criteria. Encapsulated empyema cannot be improved with conventional antibacterial drug treatment; thus, the possibility of recovery from sepsis is low without resolving the source. Therefore, the potential benefits of promptly controlling the source of infection are thought to be high for patients. It is thought that patients could be rapidly transferred to facilities capable of performing open chest drainage when parenchymal organs are present in the drainage route due to tissue adhesion and when percutaneous drainage is difficult. Possible harms associated with invasive damage include bleeding, lung injury, and pain in the wound or around the drain. Open chest drainage is highly invasive compared to percutaneous drainage and likely has a greater degree of undesired effects. However, the benefits of open chest and subcutaneous drainage are thought to outweigh its harms in cases of sepsis due to empyema, even when considering complications such as hemorrhage and lung injury or the rapid transfer to a facility capable of performing drainage procedures.

**CQ4: Antimicrobial therapy**

**Introduction**

Antimicrobial therapy for underlying infectious diseases is an essential aspect of sepsis treatment. The importance of antimicrobial therapy is that not only it is directly associated with an outcome, but it is also related to the global concern regarding antimicrobial resistance and the associated risk of reducing effective therapeutic options in the future. The judicious use of antimicrobial agents that fully incorporates the concepts of antimicrobial stewardship [78] is required.

This guideline targets the treatment of sepsis and will not delve into the details of drug selection. The basic principles underlying drug selection for patients with sepsis are the same as those for general infection treatment. In other words, antimicrobial agents to be administered are selected by assuming specific microorganisms and drug resistance as much as possible based on patients’ backgrounds, suspected infectious foci, epidemiological information on the region or facility, and recent antimicrobial use. However, it is important to promptly administer effective antimicrobials against causative pathogens in septic patients compared to non-critically ill patients.

With regard to antimicrobial therapy for patients with sepsis, empiric antimicrobials should initially be selected after assuming the underlying microorganism, which should then be optimized to targeted antimicrobial agent(s) after the causative pathogens and their susceptibility patterns have been determined.

The appropriateness of empiric antimicrobials is associated with mortality outcomes [79]. The underlying microorganism should be determined for each suspected source of infection based on patients’ background, epidemiology, and rapid diagnostic tests, and the drug should be selected in consideration of the properties of drug distribution/tissue penetration and antimicrobial resistance. Indications for carbapenems and pathogens that require antimicrobial drugs other than β-lactams have been described. The timing of initiation of empiric antimicrobial drug administration has also been described.

With regard to interventions after culture results are obtained, 1) the possibility of termination when culture results are negative, 2) the significance of de-escalation to target antimicrobial agents with narrower spectrum, 3) procalcitonin guidance as a reference for the discontinuation of antimicrobial drugs, and 4) the possibility of setting up a relatively short duration (within 7 days) of antimicrobial therapy are provided. These reflect fundamental concepts of antimicrobial stewardship.

For the selection and administration of drugs, 1) when to consult the antimicrobial stewardship team, 2) continuous or prolonged infusion of β-lactams based on the pharmacokinetic-pharmacodynamic theory, and 3) dose adjustment of renally excreted antimicrobials are discussed.

**Fig. 3:**
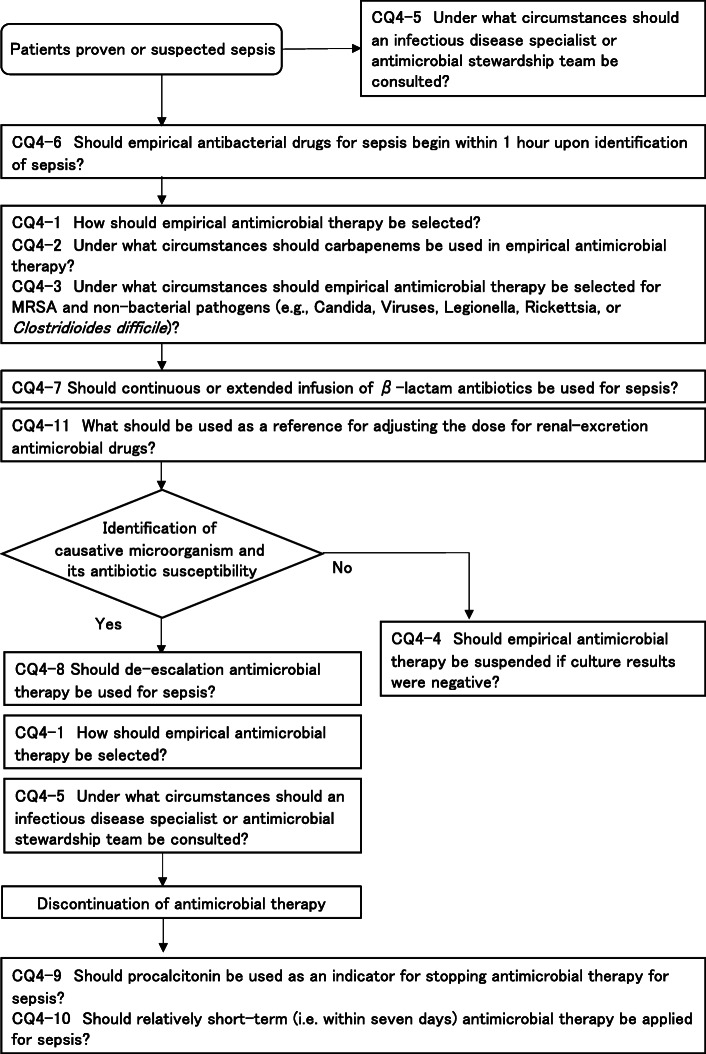
CQ4: Antimicrobial therapy (clinical flow)

Clinical flow of these CQs is shown in Fig. 3.

**CQ4-1: How should empirical antimicrobial therapy be selected?**

***Answer:*** Antimicrobials can be selected by estimating the causative microorganism based on suspected infectious foci, patient background, epidemiology and rapid microbial diagnostic tests, and by considering the tissue penetration properties of drugs and the probabilities of resistant bacteria (see Table 2 for reference). (Provision of information for background question).

***Rationale***

The selection of empiric antimicrobial therapy should include the determination of the causative microorganisms for each suspected source of infection based on the patient’s background and the epidemiology of the disease. This should be done according to the tissue penetration properties of drugs, antibacterial spectrum (including the possibility of resistant bacteria), clinical evidence, and the results of rapid diagnostic testing if available.

Table 2 (Empiric therapeutic agents for each infectious disease) shows a list of empiric antimicrobial therapy selections for each combination of common sources of infection and patient background based on expert opinions. It is assumed that this table will serve as a reference for decision-making by adding information such as an individual patient’s circumstances and the local/regional epidemiological factors and using them alongside antimicrobial therapy guidelines in each region or medical facility. Furthermore, antimicrobial therapy guidelines for each region or facility can be created using this table as a foundation if such guidelines do not exist.

The causative microorganisms can be determined based on the epidemiology of each source of infection. As such, the identification of the source of infection is important not only for surgical drainage, but also for specimen collection to select appropriate antimicrobial therapy. Two epidemiological studies conducted in Japan (2010–2011: 15 facilities; 2016–2017: 59 facilities) indicated that common sources of sepsis were respiratory infections, intra-abdominal infections, urinary tract infections, and soft tissue infections, all of which accounted for approximately 90% of cases [80, 81] Similar trends were observed in multiple international studies [82–86]. Meanwhile, reports have also indicated that a source of infection was not identified in approximately 1/6th of patients with sepsis [82–86]. Infectious diseases that should be considered when a specific source of infection could not be identified included diseases in which specific findings are difficult to determine (e.g., infectious endocarditis, catheter-related bloodstream infections) and systemic infections in which a source of infection did not form or was unclear (e.g., fulminant infection following splenectomy, purpura fulminans, rickettsial infection, febrile neutropenia with unknown source, etc.). Caution should be taken in evaluating implantable device-related infections (e.g., catheter-related bloodstream infections, prosthetic valve endocarditis, cerebrospinal fluid shunt-related meningitis/ventriculitis, and prosthetic joint infection) since specific findings are difficult to determine [87–90].

The causative microorganisms can also be determined based on patient background. There are two factors: 1) external factors such as history of exposure (including healthcare exposure or travel history), and 2) internal factors – the patient’s own conditions (including age, sex, and underlying diseases). The classification of patient background factors for selecting antimicrobial therapy varies depending on the source of infection. Community-acquired infections generally have causative microorganisms that differ from those of healthcare-associated infections, and *Pseudomonas aeruginosa* does not need to be routinely covered as a community-acquired pathogen. Exposures, which serve as risk factors for healthcare-associated infections, include invasive procedures or devices (surgery, transplantation, intravascular catheters, urinary catheters, endotracheal tubes, enteral feeding tubes, etc.) and antimicrobial therapy history. For patients with sepsis with a travel history, there is a need to consider systemic infections such as malaria, meningococcal infections, viral hemorrhagic fever, rickettsial diseases, and infections due to drug-resistant bacteria [91, 92]. Sepsis due to rickettsial infection (Japanese spotted fever and scrub typhus) or severe fever with thrombocytopenia syndrome (SFTS) should be included in the differential diagnosis if the patient has a history of travel to endemic areas of tick-borne infectious diseases in Japan [93]. Furthermore, age is an important patient factor because the causative bacteria in meningitis differ depending on whether the patient is older than 50 years [94]; more than 90% of cases of Legionnaires’ disease leading to pneumonia occur in patients older than 50 years [95]. Urinary tract infections and soft tissue infections are common among diabetic patients [96]. *Pseudomonas aeruginosa* and/or methicillin-resistant *Staphylococcus aureus* (MRSA) should be considered in neutropenic sepsis [97]. Pneumocystis pneumonia should be included in the differential diagnosis of pneumonia in patients with cellular immunodeficiency, such as human immunodeficiency virus infection [98].

Rapid diagnostic testing should be implemented if possible after the causative microorganisms have been determined from epidemiological information relating to the source of infection and the patient’s background. Gram staining can aid in the identification of significant microorganisms by determining whether local inflammation is present through the presence of leukocytes in the collected specimen. It is important to examine whether the coverage of empiric antimicrobial therapy is sufficient while considering the quality of the specimen when performing Gram staining [73].

Antimicrobial therapy covering inferred or confirmed microorganisms should be selected with due consideration to tissue penetration properties of drugs, antimicrobial resistance, and clinical evidence. Caution with regard to the tissue penetration and in situ activity of the antimicrobial therapy are shown as follows: ceftriaxone, cefepime, and meropenem can be used as β-lactams in the treatment of meningitis; however, cefazolin should be avoided due to inappropriate cerebrospinal fluid penetration. Daptomycin should be avoided in the treatment of pneumonia because it is deactivated by alveolar surfactants [99].

Drug resistance is an increasingly widespread problem globally and constitutes a threat to the treatment of sepsis [100–104]. The susceptibility rates of antimicrobial therapy vary according to time and place (country, region, facility, and hospital ward), and it is important to determine the local data by region or facility via methods such as antibiograms [105]. As antibiograms are the collected data of specimens that were submitted for various objectives, caution should be taken in their usage as reported resistance rates may be higher than the actual rates of limited specimens prior to antimicrobial therapy [106]. The previous culture testing results of an individual patient are also important. Previously colonizing or infecting bacteria do not always become the causative microorganisms of sepsis; however, the detection of resistant bacteria is a risk factor, and their coverage should be considered.

Empiric antimicrobial therapy should be selected to minimize the lapse in coverage of the inferred causative microorganisms and to anticipate a later transition to targeted or definitive antimicrobial agents. Changes in drug therapy need to be implemented rapidly if coverage is deemed insufficient. Targeted antimicrobial therapy should be selected to maximize the treatment effect and minimize adverse effects and collateral damage (i.e., negative influences on indigenous microbiota) [107]. It is beneficial to consider the targeted antimicrobial agents that can be used later when selecting empiric antimicrobial agents. For example, in Japan, cefazolin is the first-line treatment for methicillin-sensitive *Staphylococcus aureus* (MSSA) bacteremia with no intracranial dissemination, and its treatment performance against MSSA bacteremia was superior to that of vancomycin, which is frequently used when the presence of methicillin resistance is not known [108, 109]. With this in mind, the concomitant use of cefazolin should be considered when using vancomycin as an empiric antimicrobial agent with the objective of covering MRSA if the possibility of MSSA is deemed high. In this way, Table 13 (Target therapeutic agents by causative microorganism) shows a list of targeted antimicrobial therapies likely to be encountered in the treatment of sepsis by susceptibility result patterns. When focusing on spectrums in shifting from empirical to targeted antimicrobial therapy, changing from wide-spectrum to narrow-spectrum antimicrobial therapy is referred to as de-escalation, while the opposite is referred to as escalation [1, 3, 110] Refer to CQ4–8 for information on whether de-escalation is an effective strategy against sepsis.

**Table 13:** Target therapeutic agents by causative microorganism

Causative microorganism	Source of infection	Susceptibility result		Options	Alternatives	Remarks
**Gram-positive cocci in clusters [GPC in clusters]**						
***Staphylococcus aureus Staphylococcus aureus*(continued)**	Catheter-related bloodstream infections, vertebral osteomyelitis / septic arthritis / iliopsoas abscess, native valve endocarditis (without intracranial dissemination), pneumonia	MSSA(PCG: S & CEZ: S) * When determining “PCG: S”, non-producer of penicillinase must be confirmed.		PCG 4,000,000 units, every 4–6 h [111–113] or ABPC 2 g, every 4–6 h [5] (endocarditis: every 4 h; other: every 4–6 h)	CEZ	
		MSSA(PCG: R & CEZ: S)		CEZ 2 g, every 8 h [5, 11, 114]		Concomitant GM is not recommended [11].
		MRSA(CEZ: R & VCM: S)		VCM initial dose 25–30 mg/kg, subsequent doses 15–20 mg/kg, every 12 h [5, 11, 18, 87, 114, 115]	DAP (excluding pneumonia) or TEIC or LZD [5, 11, 18, 115]	VCM target trough value 15–20 μg/mL [11, 18, 115].
	Native valve endocarditis(with intracranial dissemination), post-operative meningitis (including cerebrospinal fluid shunt infection)	MSSA(PCG: S & CEZ: S) *When determining “PCG: S”, non-producer of penicillinase must be confirmed.		PCG 4,000,000 units, every 4–6 h [111–113] or ABPC 2 g, every 4–6 h [5] (endocarditis: every 4 h; other: every 4–6 h)	Avoid CEZ	
		MSSA(CEZ: S)		CTRX 2 g, every 12 h or CFPM 2 g, every 8 h or MEPM 2 g, every 8 h [11, 89]	Avoid CEZ	CTX was listed in ESC 2015 [114].
		MRSA(CEZ: R & VCM: S)		VCM initial dose 25–30 mg/kg,subsequent doses 15–20 mg/kg, every 12 h [5, 11, 18, 87, 114, 115]	DAP or TEIC or LZD [11, 18, 115]	VCM target trough levels: 15–20 μg/mL [11, 18, 115]. VCM + RFP, etc. for BSAC 2012 [116]
	Prosthetic valve endocarditis	GM: S & RFP: S		Each regimen of native valve endocarditis (mentioned above) + GM 2–3 mg/kg, every 24 h ± oral RFP 600 mg once a day (combination of three drugs) [11, 87, 114, 115]		Concomitant use of GM for 2 weeks. Target GM levels: 3–5 μg/mL at peak, less than 1 μg/mL at trough [11, 18]. See section on “Coagulase Negative Staphylococcus (CNS)” (next section) for RFP addition
		GM: R, AMK or LVFX: S		Substitute for GM in previous section: AMK or LVFX		
	Toxic shock syndrome	CLDM: S		Each above-mentioned regimen+CLDM 600 mg, every 8 h [117]		
		CLDM: R & LZD: S		Each above-mentioned regimen+CLDM 600 mg, every 8 h or each above-mentioned regimen+LZD 600 mg, every 12 h [117]		CLDM is for toxin production suppression purposes (suppression can also be done even when R) [118]
**Coagulase-negative*Staphylococcus*(CNS)**	Catheter-related bloodstream infections, prosthetic valve endocarditis, prosthetic joint infection	・Susceptibility-based selection is similar with that for *Staphylococcus aureus*. → see section on “*Staphylococcus aureus*” (above). ・RFP addition can be considered when conducting hardware retention strategy for prosthetic valve endocarditis or prosthetic joint infection. → Never use RFP alone due to rapid development of resistance. There is expert opinion on avoiding its use when there is a large quantity of bacteria. Susceptibility test results serve as a reference [5, 11, 87, 114].				
**Gram-positive cocci in chains <GPC in chains>**						
Streptococcus pneumoniae*Note that PCG susceptibility criteria differ for meningitis and non-meningitis	Other than meningitis (e.g., pneumonia)	PCG: S(MIC ≤2 μg/mL)		PCG 2,000,000 units, every 4 h or ABPC 2 g, every 6–8 h [5] (PCG 4,000,000 units, every 4 h or ABPC 2 g, every 4 h for endocarditis/invasive infection)	CTRX	
		PCG: I or R(MIC ≥4 μg/mL)		CTRX 2 g, every 24 h [5]	VCM or LVFX (if S)	
	Meningitis	PCG: S(MIC ≤0.06 μg/mL)		PCG 4,000,000 units, every 4 h [5, 12] or ABPC 2 g, every 4 h [5, 119]	CTRX	
		PCG: R(MIC ≥0.12 μg/mL)& CTRX: S(MIC ≤0.5 μg/mL)		CTRX 2 g, every 12 h [5, 12]	CFPM [89]	
		PCG: R(MIC ≥0.12 μg/mL)& CTRX: I or R(MIC ≥1.0 μg/mL)		VCM initial dose 25–30 mg/kg,subsequent doses 15–20 mg/kg, every 12 h + CTRX 2 g, every 12 h [5, 12, 18] (considering CTRX MIC>2 μg/mL& RFP: S, and RFP addition) [5, 119]		VCM target trough levels: 15–20 μg/mL [12, 18]
**Group A, B, C, F, G Streptococcus β-hemolytic cocci in chains**	Bacteremia, soft tissue infection	PCG: S		PCG 2–4,000,000 units, every 4 h [5] or ABPC 2 g, every 4–6 h	CEZ or CTRX	CLDM is for toxin production suppression purposes (suppression can also be done even when R)
	Toxic shock syndrome	PCG: S		Each above-mentioned regimen+CLDM 600 mg, every 8 h [5, 73]		
**Viridans Streptococcus,*S. gallolytics (S. bovis)***	Endocarditis	PCG MIC ≤0.12 μg/mL		PCG 4,000,000 units, every 4 h [5] or ABPC 2 g, every 4–6 h [11]	CTRX [5]	PCG can be continuously infused for 24 h [5], or divided between 6-h intervals [87, 114]. PCG 2–3,000,000 units, every 4 h is also an option (native valve [87, 114], prosthetic valve [114])
		PCG MIC =0.25 μg/mL		“PCG 4,000,000 units, every 4 h or ABPC 2 g, every 4 h” +GM 3 mg/kg, every 24 h (or 1 mg/kg, 2–3 times per day) [5, 11, 18, 87, 114]	CTRX (if MIC ≤0.5 μg/mL) + GM	PCG can be continuously infused for 24 h [5]. Target GM levels: 3–5 μg/mL at peak, less than 1 μg/mL at trough [11, 18]. Concomitant GM for 2 weeks in case native valve, 6 weeks in case of prosthetic valve
		PCG MIC ≥0.5		Consult an infectious disease specialist [11, 87, 114]		
	Other than endocarditis (e.g., pneumonia, bacteremia, febrile neutropenia)	PCG: S		PCG 2–3,000,000 units, every 4–6 h or ABPC 2 g, every 6–8 h [5, 120]	CTRX	For PCG, there is also a method of continuous infusion for 24 h [5]
		PCG: I/R & CTRX: S		CTRX 2 g, every 24 h [120]		
		PCG: I/R & CTRX: R & VCM: S		VCM initial dose 25–30 mg/kg,subsequent doses 15–20 mg/kg, every 12 h [120]		
***Enterococcus*sp.**	Endocarditis	PCG: S		(1) When MIC≤500 μg/mL in high-level GM resistance tests: “PCG 4,000,000 units, every 4 h or ABPC 2 g, every 4 h” + GM 3 mg/kg, every 24 h (or 1 mg/kg, 2–3 times per day) [5, 11, 87, 114] (2) GM MIC > 500 μg/mL, or when there is no combined use of GM: ABPC 2 g, every 4 h + CTRX 2 g, every 12 h [5, 11, 87, 114]		Implement high-level resistance test of GM for endocarditis. Target GM levels: 3–5 μg/mL at peak, less than 1 μg/mL at trough [11, 18]
		PCG: R (MIC ≥16 μg/mL)& VCM: S		When MIC≤500 μg/mL in high-level GM resistance tests: VCM initial dose 25–30 mg/kg,subsequent doses 15–20 mg/kg, every 12 h [18] + GM 3 mg/kg, every 24 h (or 1 mg/kg, 2–3 times per day) [5, 11]	SBT/ABPC: if S, SBT/ABPC+ GM [87, 114]	Target GM levels: 3–5 μg/mL at peak, less than 1 μg/mL at trough [11, 18] Target VCM trough levels: 15–20 μg/mL [11, 18]
		VCM: R(VRE)		DAP+ABPC(Curr Infect Dis Rep 16: 431, 2014) [87, 114]		Consultation with infectious disease specialist also necessary
	Other than endocarditis	PCG: S		PCG 3,000,000 units, every 4 h or ABPC 2 g, every 4–6 h [5]		
		PCG: R & VCM: S		VCM initial dose 25–30 mg/kg,subsequent doses 15–20 mg/kg, every 12 h [18]		
**Gram-positive rods [GPR]**						
***Bacillus*sp. (*other than Bacillus anthracis*)**	Catheter-related bloodstream infections, etc.	VCM: S		VCM initial dose 25–30 mg/kg,subsequent doses 15–20 mg/kg, every 12 h [5, 18]	CLDM [5]	
***Corynebacterium*sp.**	Catheter-related bloodstream infections, prosthetic infections, etc.	VCM: S		VCM initial dose 25–30 mg/kg,subsequent doses 15–20 mg/kg, every 12 h [5, 18]	PCG (if S) or TEIC or LZD (if S) [5]	
***Listeria monocytogenes***	Meningitis	ABPC: S		ABPC 2 g, every 4 h [5] ± GM 1.7 mg/kg, every 8 h	ST or “ABPC+ST”	Consultation with infectious disease specialist also necessary for concomitant use
***Nocardia*sp.**	Severe pneumonia / brain abscess / disseminated infection	(Routine susceptibility tests are difficult to implement, so antibacterial drug options are shown for severe cases with suspected Nocardia)		“ST trimethoprim 240–320 mg, every 8 h + IPM/CS 0.5 g, every 6 h” or “IPM/CS 0.5 g, every 6 h + AMK 15 mg/kg, every 24 h” [5, 121]	LZD, MEPM, CTRX, MINO	Consultation with infectious disease specialist also necessary. LZD is usually S. ST is rarely R, but room for debate regarding correlation between susceptibility tests and clinical effects. ST: trimethoprim 15 mg/kg/day ≒Japanese ST mixture (1 tablet or 1 g of trimethoprim is 80 mg) 3–4 tablets or 3–4 g, every 8 h
**Gram-negative cocci [GNC]**						
***Neisseria meningitidis***	Meningitis, bacteremia	PCG: S (MIC < 0.1 mg/mL)		PCG 4,000,000 units, every 4 h or ABPC 2 g, every 4 h [5, 119]	CTRX	
		PCG: R		CTRX 2 g, every 12 h [5, 119]		
**Gram-negative rods (Enterobacteriaceae) [GNR (1)]**						
***Escherichia coli*,*Proteus mirabilis*Note:See section on*Enterobacter*for*Proteus vulgaris Enterobacter***	Urinary tract infection, bacteremia, etc. (excluding meningitis)	ABPC: S		ABPC 1–2 g, every 6 h [122]	CPFX (if S) or ST (if S)	
		ABPC: R & CEZ: S		CEZ 2 g, every 8 h [5, 123, 124]		
		ABPC: R & CEZ: R & CTRX(CTX): S		CTRX 1–2 g, every 24 h [5, 123, 125]		
		ESBL-producing bacteria “CTRX(CTX): R or CAZ: R”& “MEPM:S & TAZ/PIPC: S & CMZ: S”		CMZ 1–2 g, every 8 h [7, 8, 126] TAZ/PIPC 4.5 g, every 6–8 h [9, 127] MEPM 1 g, every 8 h [5, 123, 125]		Reports indicating that CMZ and TAZ/PIPC were clinically stable and can be an option for pyelonephritis
		Either MEPM or IPM/CS are not S		Consult an infectious disease specialist		
	Meningitis	CTRX: S		CTRX 2 g, every 12 h [5, 89]		Avoid CEZ for meningitis
		CTRX: R & MEPM: S		MEPM 2 g, every 8 h [89]		
		Either MEPM or IPM/CS are not S		Consult an infectious disease specialist		
***Klebsiella*sp.**	Urinary tract infection, pneumonia, liver abscess, etc.	・ABPC: even S is naturally resistant, so ABPC is not selected. ・Similar with *Escherichia coli* in cases other than ABPC, so see section on “*Escherichia coli, Proteus*” mentioned above. ・Observational studies showing that CTRX has better performance than CEZ even with CEZ:S for invasive liver abscess syndrome [128].				
***Enterobacter*sp.,*Citrobacter*sp.,*Serratia marcescens*,*Proteus vulgaris*,*Morganella*sp.**	Bacteremia, pneumonia, etc. (excluding meningitis)	「CTRX(CTX): S & CAZ: S」& CFPM: S		CFPM (1 g, every 8 h or 2 g, every 8–12 h) [5, 123, 125, 129] TAZ/PIPC 4.5 g, every 6–8 h [125] or CTRX 1–2 g, every 24 h [5, 123, 125]	MEPM or CPFX (if S) or ST(if S)	ABPC is naturally resistant. CTRX, CAZ, and TAZ/PIPC have the potential to become resistant during treatment due to AmpC cephalosporinase production during treatment. Caution is required with cholangitis associated with biliary tract malignancies [130]
		「CTRX(CTX): R or CAZ: R」	CFPM: S & MEPM: S	CFPM (1 g, every 8 h or 2 g, every 8–12 h) [5, 123, 125]	CPFX (if S) or ST(if S)	
			CFPM: R & MEPM: S	MEPM 1 g, every 8 h [5, 123, 125]		
		Either MEPM or IPM/CS are not S		Consult an infectious disease specialist		*Serratia* is naturally resistant to colistin
	Meningitis	CFPM: S		CFPM 2 g, every 8 h [89]		Also consult an infectious disease specialist. CTRX is also an option for *C. koseri*.
		MEPM: S		MEPM 2 g, every 8 h [89, 131]		
		Either MEPM or IPM/CS are not S		Consult an infectious disease specialist.		*Serratia* is naturally resistant to colistin.
***Salmonella*sp. (other than abdominal typhus)**	Bacteremia, extra-intestinal infections (e.g., mycotic aneurysms)	ABPC: S		ABPC 2 g, every 6 h [131]	CPFX (if S)	
		ABPC: R & CTRX: S		CTRX 2 g, every 24 h [131]		2 g, every 12 h for meningitis
		ABPC: R & CTRX: R & MEPM: S		MEPM 1 g, every 8 h [131]		2 g, every 8 h for meningitis
**Gram-negative rods (non-glucose fermenting bacteria) [GNR (2)]**						
***Pseudomonas aeruginosa***	Pneumonia, urinary tract infection, bacteremia, febrile neutropenia, etc. (excluding meningitis)	CAZ: S		CAZ 2 g, every 8 h(or 1 g, every 6 h) [5, 123]	MEPM (if S) or CPFX (if S)	
		CFPM: S		CFPM 2 g, every 8–12 h(or 1 g, every 8 h) [ 67, 122]		
		PIPC: S		PIPC 4 g, every 6 h [5]		PIPC susceptibility criteria set when at least 3 g is used every 6 h [123]
		All of the above and R & MEPM: S		MEPM 1 g, every 8 h [5, 123]	CPFX (if S)	
		Either MEPM or IPM/CS are not S		Consult an infectious disease specialist		
	Meningitis	CAZ: S or CFPM: S		CAZ 2 g, every 8 h or CFPM 2 g, every 8 h [89]		
		MEPM: S		MEPM 2 g, every 8 h [89]		
***Acinetobacter baumannii***	Hospital-acquired pneumonia / ventilator-associated pneumonia, wound infection	CFPM: S		CFPM 2 g, every 8 h [5]	CPFX (if S) or MINO (if S)	
		SBT/ABPC: S		At least SBT/ABPC 3 g, every 6 h (consult an infectious disease specialist for severe cases) [5, 132]		SBT part exerts antibacterial effect
		MEPM: S		MEPM 1 g, every 8 h [123]		
		Either MEPM or IPM/CS are not S		Consult an infectious disease specialist		
***Stenotrophomonas maltophilia***	Bacteremia, pneumonia	ST: S		240–320 mg, every 8 h as ST trimethoprim [5]	MINO [5]or CPFX(if S)	Naturally resistant to carbapenem. ST: trimethoprim 15 mg/kg/day ≒Japanese ST mixture (1 tablet or 1 g of trimethoprim is 80 mg) 3–4 tablets or 3–4 g, every 8 h
**Gram-negative rods (others) [GNR (3)]**						
***Haemophilus influenzae***	Meningitis	ABPC: S		ABPC 2 g, every 4 h [12, 119]	CTRX [89]	
		ABPC: R & CTRX(CTX): S		CTRX 2 g, every 12 h [5, 119]	CFPM [89]	
	Pneumonia, epiglottitis	ABPC: S		ABPC 2 g, every 6 h [5]		
		ABPC: R & SBT/ABPC: S		SBT/ABPC 3 g, every 6 h [5]		
		ABPC: R & CTRX(CTX): S		CTRX 1–2 g, every 24 h [5]		
***Pasteurella multocida*,*Capnocytophaga canimorsus***	Animal bite	PCG: S		SBT/ABPC 3 g, every 6 h [73]	CTRX	PCG 4,000,000 units every 4 h for infections due to single bacteria
		PCG: R & SBT/ABPC: S		SBT/ABPC 3 g, every 6 h [73]	CTRX	
***Aeromonas*sp.**	Soft tissue infection, bacteremia	CTRX: S or MINO: S		CTRX 2 g, every 24 h + MINO 100 mg, every 12 h [73]	CPFX+MINO, LVFX	
***Vibrio vulnificus***	Soft tissue infection, bacteremia	CTRX: S & MINO: S		CTRX 2 g, every 24 h + MINO 100 mg, every 12 h [73]	CTX + CPFX, LVFX	Observational studies have indicated that single β-lactams had a higher mortality rate than combination therapy [133]
**Obligate anaerobic bacteria**						
**Obligate anaerobic bacteria (other than*C. difficile*)**	Polymicrobial infections	・The extent to which undetected obligate anaerobic bacteria that should be covered depends on whether drainage was sufficient. ・Antibacterial drug selections for polymicrobial infections caused by obligate anaerobic bacteria not only are determined by the susceptibility results of detected anaerobic bacteria but also involves the estimation of mixed infection by multiple anaerobic and aerobic bacteria. ・Obligate anaerobic bacteria have the three following characteristics depending on the susceptibility rate. (1) Most obligate anaerobic bacteria above the diaphragm (e.g., *Peptostreptococcus* sp., *Prevotella* sp.) are susceptibile to β-lactams represented by PCG and CLDM. However, some include β-lactamase-producing bacteria and CLDM-resistant bacteria (e.g., some *Prevotella*). (2) Obligate anaerobic bacteria below the diaphragm (e.g., *Bacteroides* sp.) include β-lactamase-producing bacteria. The resistance rates of non-fragilis Bacteroides(other than *B. fragilis*) in particular against CLDM and CMZ have been increasing. (3) Most obligate anaerobic bacteria which include (1) and (2) are susceptibile to SBT/ABPC, TAZ/PIPC, MEPM, and MNZ. ・Therefore, the two following points should be considered when selecting a target therapeutic drug for polymicrobial infections where obligate anaerobic bacteria contribute: (1) To what extent obligate anaerobic bacteria are covered based on information about whether it is above or below diaphragm or drainage is sufficient, and (2) Causative bacteria other than obligate anaerobic bacteria are covered.				
***Peptostreptococcus*sp.,*Prevotella*sp. (obligate anerobic bacteria above the diaphragm)**	Lung abscess, deep cervical infection, etc.	The right shows typical options. Susceptibility results of detected bacteria other than obligate anaerobic bacteria can also serve as a reference for selection.		SBT/ABPC 3 g, every 6 h or CLDM 600 mg, every 8 h or “MNZ 500 mg, every 8 h + (PCG 2–3,000,000 units, every 4 h or CTRX 2 g, every 24 h” [134]	TAZ/PIPC	
	Brain abscess			“(PCG 4,000,000 units, every 4 h or CTRX 2 g, every 12 h or CFPM 2 g, every 8 h) + MNZ 500 mg, every 8 h” [66]		
***Bacteroides*sp. (obligate anerobic bacteria below the diaphragm)**	Polymicrobial intra- abdominal infection (secondary peritonitis, intraperitoneal abscess, cholangitis)	Insufficient drainage	The right shows typical options. Susceptibility results of detected bacteria other than obligate anaerobic bacteria can also serve as a reference for selection.	SBT/ABPC 3 g, every 8 h or TAZ/PIPC 4.5 g, every 8 h or “MNZ 500 mg, every 8 h + (CEZ 2 g, every 8 h or CTRX 2 g, every 24 h or CFPM 2 g, every 12 h or CPFX 400 mg, every 12 h)“ [5]	MEPM	CMZ: R and CLDM: R are increasing [5]
		Sufficient drainage		CMZ 1 g, every 8 h or 「CLDM 600 mg, every 8 h + (CEZ 2 g, every 8 h or CTRX 2 g, every 24 h or CFPM 2 g, every 12 h or CPFX 400 mg, every 12 h)” or “insufficient drainage” option in previous section [5]		
***Clostridium*sp. (e.g.,*C. perfringens*)**	Gas gangrene	PCG: S		PCG 4,000,000 units, every 4 h +CLDM 600 mg, every 8 h [5, 73]		CLDM is for toxin production suppression purposes (suppression can also be done even when R) [5]
***Clostridioides (Clostridium) difficile***						
***Clostridioides (Clostridium) difficile***	*Clostridioides difficile* infection (CDI)	Initial onset		VCM 125 mg, four times a day (orally or through nasogastric tube) [5, 135]	Non-severe: MNZ orally	Intravenous VCM is ineffective
		Initial onset		VCM tapering regimen (starting at 125 mg, four times a day) or FDX 200 mg, two times a day [135]	When initial treatment is MNZ: VCM	
		Shock, hypotension, megacolon, ileus, VCM 125 mg regimen is ineffective		“VCM 500 mg, every 6 h (orally or through nasogastric tube) 500 mg / saline 100 mL as stationary enema through anus for ileus” ±MNZ 500 mg, intravenously every 8 h [135]		
**Other bacteria**						
***Legionella*sp.**	Pneumonia			LVFX 500–750 mg, every 24 h [5] or AZM 500 mg, every 24 h [5]	MINO [20]	
***Mycoplasma pneumoniae***	Pneumonia			MINO 100 mg, every 12 h [5]	AZM or LVFX	
***Rickettsia japonica***	Japanese spotted fever			MINO 100 mg, every 12 h [13]	CPFX	
***Orientia tsutsugamushi***	Scrub typhus			MINO 100 mg, every 12 h [13]	AZM	CPFX is ineffective
***Leptospira interrogans***	Leptospirosis			PCG 1500,000 units, every 6 h [136]	CTRX or MINO	
**Fungi**						
***Candida***	Candidemia, disseminated candidiasis (includes febrile neutropenia)	・Empirical treatment (normally MCFG) should be stepped down to oral FLCZ or VRCZ mentioned below if blood culture negativity and clinical stability are confirmed. ・Complications of endophthalmitis should involve switching to FLCZ or VRCS since MCFG has poor intraocular penetration (L-AMB ± 5-FC if there is resistance to FLCZ and VRCZ). ・Most of *C. albicans, parapsilosis,* and *tropicalis* are susceptible to FLCZ, *C. glabrata* is either susceptible or resistant, and *C. krusei* is naturally resistant. The difficult-to-identify *C. auris* (can be FLCZ resistant or multi-drug resistant) has been recently reported. ・Most cases of candiduria are not treated; however, candidemia and disseminated candidiasis can be diagnosed as a result of candiduria. An infectious disease specialist should also be consulted when candiduria requires treatment (MCFG and L-AMB have poor urinary tract penetration).				
***Candida albicans, C. parapsilosis, C. tropicalis***	After stabilization of candidemia	FLCZ: S		FLCZ initial dose 800 mg(subsequent doses 400 mg), every 24 h [137]		
***C. glabrata***		FLCZ: S		FLCZ initial dose 800 mg(subsequent doses 400 mg), every 24 h [137]		Completing treatment as only MCFG is also an option. Consult an infectious disease specialist
		FLCZ: R & VRCZ: S		VRCZ initial dose 6 mg/kg, every 12 h (subsequent doses 4 mg/kg, every 12 h) [137]		
***C. krusei***		FLCZ: R & VRCZ: S		VRCZ initial dose 6 mg/kg, every 12 h (subsequent doses 4 mg/kg, every 12 h) [137]		
***Aspergillus*sp.**	Invasive pulmonary aspergillosis			VRCZ initial dose 6 mg/kg, every 12 h (subsequent doses 4 mg/kg, every 12 h) [5, 137]	L-AMB [20]	
***Pneumocystis jirovecii***	Pneumocystis			240–320 mg as ST trimethoprim, every 8 h [5],	Intravenous infusion of pentamidine [5]	ST: trimethoprim 15 mg/kg/day ≒Japanese ST mixture (1 tablet or 1 g of trimethoprim is 80 mg) 3–4 tablets or 3–4 g, every 8 h
***Cryptococcus*sp.**	Meningitis (non-HIV)			L-AMB 3–4 mg/kg, every 24 h + 5-FC 25 mg/kg orally, every 6 h [137]	FLCZ (high dose)	
***Mucor*sp., etc.**	Mucormycosis			L-AMB 5–10 mg/kg, every 24 h [137]		
**Virus**						
**Influenza**	Pneumonia, etc.			Oseltamivir 75 mg orally, twice a day [138]	Peramivir	
**SFTS**	Severe fever with thrombocytopenia syndrome			Undergoing research [139]		
**CMV**	Pneumonia, etc.			Ganciclovir 5 mg/kg, every 12 h [5]	Foscarnet	
**HSV**	Pneumonia, etc.			Acyclovir 10 mg/kg, every 8 h [140]		

[Precautions] This table refers to guidelines relating to each infectious disease and the JAID/JSC infectious disease treatment guidelines and adds susceptibility test criteria [123] and information regarding proper use of antimicrobial agents [141] to provide an overview of items relating to sepsis. Representative options were displayed for practical use

Experts in the septic/antimicrobial appropriate use support stewardship teams of each facility can use this table as a reference when promoting de-escalation by adding the local information of each facility (e.g., available antimicrobial agents)

Abbreviations: *PCG* penicillin G, *ABPC* ampicillin, *AMK* amikacin, *AZM* azithromycin, *CAZ* ceftazidime, *CEZ* cefazolin, *CFPM* cefepime, *CLDM* clindamycin, *CMZ* cefmetazole, *CPFX* ciprofloxacin, *CTRX* ceftriaxone, *CTX* cefotaxime, *DAP* daptomycin, *5-FC* flucytosine, *FDX* fidaxomicin, *FLCZ* fluconazole, *GM* gentamycin, *IPM/CS* imipenem/cilastatin, *L-AMB* liposomal amphotericin B, *LVFX* levofloxacin, *LZD* linezolid, *MCFG* micafungin, *MEPM* meropenem, *MINO* minocycline, *MNZ* metronidazole, *PIPC* piperacillin, *RFP* rifampicin, *SBT/ABPC* sulbactam/ampicillin, *ST* sulfamethoxazole, *TAZ/PIPC* tazobactam/piperacillin, *TEIC* teicoplanin, *VCM* vancomycin, *VRCZ* voriconazole. (Abbreviations of antimicrobials are based on JAID/JSC infectious disease treatment guidelines)

Various investigations have been conducted to improve prognosis by optimizing the selection of antimicrobial therapy in sepsis. The J-SSCG 2016 recommended against routine empiric combination therapy due to the lack of evidence of improved prognosis and treatment harm, including renal injury(1B) [3]. Carbapenem is often selected for the treatment of sepsis; however, some initiatives have strategically implemented carbapenem-sparing regimens that avoid excessive carbapenem use as the threat of drug resistance has become a global issue. The use of appropriate antimicrobial therapy in supporting additional payments were established in 2018 in Japan, and the establishment of consultation systems of infectious disease specialists and support systems for proper antimicrobial therapy (i.e., antimicrobial stewardship teams) has been promoted. Refer to CQ4–2 (Under what circumstances should carbapenems be used in empiric antimicrobial therapy?), CQ4–3 (Under what circumstances should empiric antimicrobial therapy be selected for MRSA and non-bacterial pathogens (e.g., *Candida*, viruses, *Legionella*, *Rickettsia*, and *Clostridioides difficile*)), and CQ4–5 (Under what circumstances should an infectious disease specialist or antimicrobial stewardship team be consulted?).

Finally, the complexity of the body of specialized knowledge of infectious diseases continues to grow, including the diversification of the causes of sepsis, the global threat of antimicrobial resistance, the decline in antimicrobial agent development, the constraints of drug supply, and multiple revisions to the criteria of susceptibility tests. There are also problems in clinical practice that hinder the optimization of antimicrobial therapy, such as “culture-negative sepsis” (CQ4–4) which refers to the fact that even if the proper test is performed in cases of sepsis, 30–50% of culture tests yield negative results. It is important to faithfully practice the basic principles underlying infectious disease management –“estimating the causative microorganisms based on the source of infection, patient background, epidemiology, and rapid diagnostic testing results, as well as considering the tissue penetration properties of drugs, antimicrobial resistance, and clinical evidence”, in order to effectively use limited antimicrobial therapy resources.

**CQ4-2: Under what circumstances should carbapenems be used in empirical antimicrobial therapy?**

***Answer:*** Carbapenems can be included in the empirical antimicrobial regimen when the use of carbapenem is considered to be particularly effective; ESBL-producing Enterobacteriaceae or *Pseudomonas aeruginosa* or Acinetobacter species with limited susceptibility for carbapenems (Provision of information for background question).

***Rationale***

Carbapenems that are currently available in Japan for intravenous injection include meropenem, doripenem, imipenem/cilastatin, panipenem/betamipron, and biapenem. The antibacterial spectrum of all of these drugs is virtually the same and wide-ranging, from Gram-positive to Gram-negative bacteria. However, methicillin-resistant Staphylococcus and Enterococcus species, *Stenotrophomonas maltophilia*, and fungi are not sensitive to these agents.

Several RCTs have compared the effects of carbapenems and other wide-spectrum β-lactams, but were not designed to distinguish between empiric and target therapy for sepsis. The treatment effects of carbapenems were identical to those of β-lactams alone or concomitant use of β-lactams, aminoglycosides, or metronidazole [142]. RCTs of patients with severe infections showed that carbapenems had an efficacy comparable to that of tazobactam/piperacillin in the treatment of pneumonia [143–145]. Carbapenems also had an efficacy comparable to that of tazobactam/piperacillin [146] or quinolones [147] for intraperitoneal infection, and to third-generation cephalosporins [148, 149] for meningitis. Taken together, the routine use of carbapenems in patients with sepsis has not yet been determined to be superior.

There is an opinion that carbapenems should only be used selectively if a specific microorganism is suspected as a causative pathogen. Currently, the increase in the number of strains that produce extended-spectrum β-lactamase (ESBL) among the Enterobacteriaceae is a concern [150]. Apart from carbapenems, other treatment options for ESBL-producing strains include broad-spectrum penicillin with β-lactamase inhibitors combinations, cephamycin, and aminoglycosides. Observational studies have shown that these agents are not inferior to carbapenems [151], while some RCTs have shown that carbapenems are superior [152, 153]. As empiric therapy, carbapenems are likely the first-line treatment option, particularly in critically ill patients, such as those with sepsis/septic shock. Furthermore, the number of resistant *Pseudomonas aeruginosa* and Acinetobacter species with sensitivity only to carbapenems has been increasing. It is logical to select carbapenems when these microorganisms are suspected. However, these types of resistant strains are rarely encountered in Japanese clinical settings.

Meanwhile, the issue of carbapenem-resistant Gram-negative bacilli is becoming a global problem. The resistance rate of *Pseudomonas aeruginosa* due to antimicrobial exposure is the highest, particularly to carbapenems [154, 155]. The use of carbapenems has been found to be the commonest risk factor for multidrug-resistant *Pseudomonas aeruginosa* or *Acinetobacter* species [156]. A meta-analysis showed that the odds ratio (OR) of the occurrence of carbapenem-resistant *Pseudomonas aeruginosa* due to the use of carbapenems was 7.09 (95%CI 5.43 to 9.25) [157]. The proportions of carbapenem-resistant *Pseudomonas aeruginosa* detected in the Japanese Nosocomial Infection Surveillance (JANIS) study were high, at 11 and 17% for meropenem and imipenem, respectively [158]. Furthermore, carbapenem use is a risk factor for the identification of carbapenem-resistant Enterobacteriaceae, including carbapenemase-producing strains [159]. The increase in the number of resistant bacteria worldwide, particularly in developing countries, has become a concern. The proportion of Gram-negative bacilli in the group of carbapenem-resistant Enterobacteriaceae is still low (at less than 0.5% according to the JANIS data) [158]; however, this is expected to increase in the future with globalization. The presence of resistant bacteria increase the inappropriateness of empiric antimicrobial therapy and are associated with poor outcomes [160–162]. Taken together, carbapenems should be used when appropriate, being aware of the risk of development of drug-resistant strains.

The emphasis on the appropriate use of carbapenem leads to the use of carbapenem in limited cases in which the causative bacteria are one of the aforementioned microorganisms with carbapenem-limited sensitivity. This is the most conservative option when using carbapenems. This guideline supports this conservative option from the viewpoint of prioritizing antimicrobial stewardship and the current situation of frequent use of carbapenems in Japan. Specifically, carbapenems can be selected when ESBL-producing Gram-negative bacilli, multidrug-resistant *Pseudomonas aeruginosa*, or Acinetobacter species with limited sensitivity to carbapenem are suspected.

Multiple studies including systematic reviews have reported on the risk factors for infections caused by ESBL-producing strains [150, 163, 164], third-generation cephalosporin-resistant Enterobacteriaceae [165], and multidrug-resistant *Pseudomonas aeruginosa* [159]. Although there are differences according to the microorganism, the primary risk factors shared by many studies were a history of administration of antimicrobial agents and colonization by any resistant pathogen.

Rottier et al. [165] assessed the risk factors for infection due to the third-generation cephalosporin-resistant Enterobacteriaceae among Gram-negative bacilli bacteremia. The authors showed that using carbapenems selectively in cases of colonization with multidrug-resistant bacteria could avoid the excessive use of carbapenems or aminoglycosides without reducing their appropriateness.

Lambregts et al. [166] extracted the risk factors for the presence of second-generation cephalosporins and aminoglycoside-resistant bacteria as colonization and history of using those antimicrobials in Enterobacteriaceae bacteremia. The authors showed that carbapenem use could be decreased and the appropriateness of empiric therapy increased when carbapenem was administered selectively for cases with risk factors.

Based on these results, (i) colonization or a history of infection with a resistant pathogen, or (ii) a history of administration of antimicrobial agents can be listed as risk factors for infections with ESBL-producing bacteria, multidrug-resistant *Pseudomonas aeruginosa* or Acinetobacter species that have sensitivity only to carbapenems, against which carbapenems can be considered as empiric therapy.

However, antimicrobial agents that can be used as alternatives to carbapenems and their resistance patterns can vary according to the country, region, facility, and department. Thus, consideration should be given to each clinical setting.

**CQ4–3: Under what circumstances should empirical antimicrobial therapy be selected for MRSA and non-bacterial pathogens (e.g., Candida, Viruses, Legionella, Rickettsia, or Clostridioides difficile)?**

***Answer:*** Each microorganism can be covered by empirical antimicrobial regimen if highly suspected by suspected infectious foci, patient background and culture results (Provision of information for background question).

***Rationale***

The onset of infectious diseases and the risks of exacerbation should be considered when selecting empiric antimicrobials for the treatment of infection with MRSA and other specific bacteria, as described here.

- MRSA

Reports have indicated that 30 and 50% of adults are temporary or permanent carriers, respectively, of *Staphylococcus aureus* [167, 168]. Bacterial loads in permanent carriers and the risk of *S. aureus*-based infection were particularly high [169]. The risks of carriage among patients with diabetes, hemodialysis, peritoneal dialysis, atopic dermatitis, medical exposure, recurrent *S. aureus* skin infections, human immunodeficiency virus (HIV) infection, and drug addiction were found to be high [170]. Medical exposures, such as diabetes, chronic obstructive pulmonary disease, and heart failure, are also risk factors for carriage of MRSA, which is a multiple drug-resistant bacterium [171].

*Staphylococcus aureus*-based infectious diseases are of a wide range, and include skin/soft tissue infections, osteomyelitis, arthritis, surgical site infections, community-acquired pneumonia following influenza virus infection, nosocomial pneumonia/ventilator-associated pneumonia, bacteremia, catheter-related bloodstream infections, infectious endocarditis, and toxic shock syndrome [172]. Known risk factors caused by *S. aureus*-based infectious diseases include hemodialysis (risk ratio [RR] 257–291), peritoneal dialysis (RR 150 to 204), diabetes (RR 7), heart disease (RR 20.6), stroke (RR 6.4), cancer (RR 7.1 to 12.9), systemic lupus erythematosus (RR 2.4), rheumatoid arthritis (RR 2.2), HIV infection (RR 23.7), solid organ transplantation (RR 20.7), and alcohol addiction (RR, 8.2) [172]. *S. aureus* should be considered a possible causative bacterium in skin and soft tissue infections in which clustered Gram-positive cocci are observed in Gram’s staining from specimen via puncture of subcutaneal or lymph node abscesses [73].

- *Legionella pneumophila*

It is clinically difficult to distinguish between Legionnaires’ disease and bacterial pneumonia [173]. *L. pneumophila* is a Gram-negative bacilli that lives in aquatic environments and grows well in warm water with temperatures ranging between 25 °C and 40 °C. The most important source of Legionnaires’ disease is aerosolized contaminated water [174, 175]. High risk factors for onset include male sex, smoking, chronic heart disease, lung disease, diabetes, end-stage renal disease, solid organ transplantation, immunodeficiency, cancer presence, and an age greater than 50 years [175].

Infection with *L. pneumophila* should be considered when the above-mentioned risk factors are present in patients with pneumonia and aquatic exposure.

- *Rickettsia* spp.

Cases of rickettsia reported in Japan include Tsutsugamushi disease due to *Orientia tsutsugamushi* and Japanese spotted fever due to *Rickettsia japonica*. Both are tick-borne diseases, with infection in the former caused by the bite of Tsutsugamushi larvae and the latter by hard ticks (part of the *Haemaphysalis* and *Ixodes* genera).

The three main characteristics of Tsutsugamushi disease are fever (95%), rashes (86%), and black scabs/eschars (85%) [176]. Eschars more often form on the trunk rather than on the limbs, and are difficult to find when not suspected. Delayed treatment can result in signs such as elevated levels of hepatic enzymes and a decreased platelet count, with a mortality rate of 0.5%.

Japanese spotted fever presents with a higher rate of fever (99%) and rashes (94%); however, eschars are relatively less common compared to that of Tsutsugamushi disease (at 66%). Elevated levels of hepatic enzymes (73%), headaches (31%), and disseminated intravascular coagulation (DIC) (20%) are commonly observed, with a mortality rate of 0.9% [176].

Outdoor activities in tick habitats, a history of tick bites, and eschars are important findings; however, these are not always present, and organ failure can be fatal in cases in which treatment is delayed. Specimens should be collected in consultation with a public health center when suspected to be the cause of sepsis, and there are opinions that empiric treatment should be initiated without waiting for test results. Furthermore, consideration should be given to rickettsial diseases such as Q fever, anaplasmosis, ehrlichiosis, Rocky Mountain spotted fever, and typhus fever following overseas travel. Empiric treatment should be initiated without waiting for laboratory results following specimen collection in consultation with a public health center if suspected to be a cause of sepsis.

- *Clostridiodes difficile*

*Clostridiodes difficile* is a microorganism that is ubiquitous in the environment, including soil, water, and food. *C. difficile* infection (CDI), caused by a toxin-producing type, has been reported to range from mild cases that presents only with self-limiting diarrhea to severe cases. Severe cases that affect vital prognosis are characterized by high fever, abdominal pain, hyperleukocytosis (leukocyte count ≥25,000/μL), hypoalbuminemia, renal failure, shock, and toxic megacolon [177].

Exposure to antibacterial drugs is the most important risk factor for the onset of CDI, and the risk of onset is highest during and 1 month after antimicrobial therapy. The risks vary according to the type of antibacterial drug (see Table 2 for reference) [178], with the use of proton pump inhibitors and antacids such as histamine 2 receptor blockers known to be a risk factor for CDI [179]. Other risk factors for CDI include old age, a history of hospitalization, severe underlying disease, following abdominal surgery, nasal catheter placement, and long-term hospitalization [180].

CDI should be considered when there is a history of exposure to antibacterial drugs and when the above-mentioned risk factors are present in patients with abdominal symptoms or shock.

- *Candida* spp.

*Candida* is a yeast-like fungus that is ubiquitous in the human body. It normally does not induce infectious diseases; however, it can cause superficial infections such as thrush or esophageal candida in immunosuppressed patients, as well as invasive infections such as bacteremia, catheter-related bloodstream infections, infective endocarditis, solid organ abscesses, meningitis, and endophthalmitis [181]. The risk factors of invasive *Candida* infection include the use of broad-spectrum antibacterial drugs, intravascular catheter placement, artificial device placement, parenteral nutrition via high-calorie infusion, the use of cytotoxic anticancer agents, following solid organ transplantation, and *Candida* colonization. Reports have indicated that appropriate antifungal drug administration in the early stage can reduce the mortality rate by up to 50%, and there is the opinion that the concomitant use of antifungal drugs should be assessed when treating sepsis in patients with these risk factors [182]. However, there is also the opinion that administering antifungal drugs to persons with only *Candida* colonization is inappropriate, and it is thought that further investigation combined with other clinical information is needed [183].

- Viral infections

Influenza virus

Seasonal influenza can cause symptoms such as a sudden onset of high fever, chills, muscle pain, and nausea, and may naturally improve without complications. However, some patients may experience severe disease with complications such as pneumonia, myocarditis, and encephalitis/encephalopathy [184]. As an imported infection, avian influenza (such as H7N9) can cause acute respiratory distress syndrome (ARDS), which has an extremely high mortality rate of approximately 30% [185]. The risk factors for exacerbation of influenza infection include an age greater than 65 years; pregnancy during an epidemic; chronic respiratory diseases including asthma; heart, kidney, liver, and blood disorders; diabetes; immunodeficiency; decreased respiratory function; patients at a high risk of aspiration or professionals who handle respiratory secretions; obesity with a body mass index greater than 40 kg/m^2^; long-term care on a hospital ward; and a history of travel to areas with avian influenza or novel influenza spread [186]. The sensitivity of rapid influenza antigen diagnostic testing is still low (62%) [187]; therefore, there are opinions that anti-influenza drugs should be administered to patients with a history of travel to areas with seasonal influenza or avian influenza epidemics among whom respiratory failure/myocarditis or encephalitis/encephalopathy is suspected [186].

Herpes simplex virus

The herpes simplex virus (HSV) is a DNA virus, and reports have indicated that more than 90% of adults have already been infected with HSV I [188]. The virus typically causes recurrent cold sores; however, fatal infections such as encephalitis and disseminated infections can occur among immunocompromised patients.

Encephalitis has a bimodal age distribution among persons younger than 30 years and older than 50 years, and reports have indicated its onset even in immunocompetent persons [189]. It characteristically presents with temporal lobe neuropathy compared to other types of viral encephalitis; however, its differentiation is difficult [190].

Reactivation in immunosuppressed conditions such as post-solid organ transplantation, bone marrow transplantation, or during HIV infection can cause severe HSV infection, which can result in fatal disseminated infections such as widespread mucosal rashes and internal organ disorders such as liver failure. The risk of severe HSV infection in organ transplant patients is highest within 30 days of transplantation, when the risk of T-cell immunosuppression is the highest [191], and when an HSV1-positive recipient receives a bone marrow transplant from an HSV1-negative donor [192]. Furthermore, initial HSV2 infection in pregnant women increases the risk of a disseminated infection [188].

Regarding the diagnosis, serum antibody titer tests take time, and the interpretation of results in immunocompromised patients is difficult. Thus, polymerase chain reaction (PCR) assay tests are performed using samples such as serum, cerebrospinal fluid, and blistering fluid. However, as it is difficult to obtain the results of these tests, there is an opinion that treatment should be started when HSV infection is suspected in patients at a high risk of severe HSV infection.

Cytomegalovirus

The cytomegalovirus (CMV) is a DNA virus, and reports have indicated that more than 50% of adults in developing countries have been previously infected with this virus [193]. Typically, the virus does not cause fatal infections, but cytomegalovirus diseases such as encephalitis, retinochoroiditis, enteritis, and pneumonia can be fatal in immunosuppressed patients following solid organ transplantation, bone marrow transplantation, or during HIV infection. For this reason, the cytomegalovirus load in immunosuppressed patients should be periodically monitored using rapid virus identification (shell vial method), CMV antigenemia test (CMV antigenemia method), and quantitative PCR methods, and there is the opinion that treatment should be initiated promptly when symptoms appear [194].

Severe fever with thrombocytopenia syndrome virus

The severe fever with thrombocytopenia syndrome (SFTS) virus was discovered in China in 2010, and there have been reports of infection in China, Japan, and South Korea [195]. It is a tick-borne disease, and infection occurs through the bite of the intermediate host, the Asian longhorned tick (*Haemaphysalis longicornis*). Its symptoms are nonspecific and include fever, digestive symptoms, headache, and muscle aches; however, SFTS can also induce central nervous system signs such as altered consciousness, hemorrhage, and elevated hepatic enzyme levels. The infection resolves naturally after approximately 1–2 weeks, although 27% of patients die, and reports have indicated that many cases of mortality have malignant tumors [196]. Furthermore, half of those infected were engaged in agricultural work, and reports indicated that they engaged in outdoor activities prior to disease onset [196]. Effective drugs for the treatment of SFTS are still in development.

**CQ4-4: Should empirical antimicrobial therapy be suspended if culture results were negative?**

***Answer:*** We suggest stopping any empiric antimicrobials where sepsis is excluded by negative culture results and after careful consideration of clinical progress (expert consensus: insufficient evidence).

***Rationale***

The results of a systematic review conducted to evaluate whether antimicrobial administration could be concluded after empiric antimicrobial treatment was started based on the diagnosis of sepsis in the face of a negative culture result yielded one RCT [197]. The subjects of this open-label, single-center, small-scale (*n* = 46) pilot study were patients whose source of infection was unclear, but among whom it was determined that antimicrobial agents should be used, and were divided into an intervention group in which antimicrobial therapy was completed after 48 h and a control group in which drugs were administered for 7 days [197]. This study suggested that short-term administration for fewer than 48 h could contribute to a decreased administration of broad-spectrum antimicrobial agents without worsening vital prognosis. However, this study did not necessarily determine whether antimicrobial treatment should be suspended based on the result of culture, did not accurately conform to the PICO criteria, and was not an RCT that could directly answer this CQ. Therefore, this was not considered a study that was relevant to the systematic review of this CQ.

The sepsis diagnostic criteria (Sepsis-1 and − 2), which incorporated the systemic inflammatory syndrome prior to Sepsis-3, often included cases in whom the final diagnosis was not even sepsis or even an infectious disease when antimicrobial agents were started after an initial diagnosis of sepsis [198]. Meanwhile, observational studies that compared culture-negative sepsis and culture-positive sepsis reported that there was either no difference in vital prognosis between the two groups, or a slight worsening in the latter group [30, 199]. The results of culture cannot be predicted at the initial stage when sepsis is diagnosed clinically; thus, the practice of antimicrobial agent administration after obtaining various cultures such as those of the blood is widespread. It is thought that concluding antimicrobial agent administration as rapidly as possible when culture results are confirmed to be negative and it could be comprehensively clinically determined that the illness is not sepsis is an important measure against antimicrobial resistance.

**CQ4-5: Under what circumstances should an infectious disease specialist or antimicrobial stewardship team be consulted?**

***Answer:*** An infectious disease specialist and/or antimicrobial stewardship team can be consulted when 1) the cause of sepsis is unknown, 2) involvement of extensively drug-resistant bacteria is suspected, 3) emerging, re-emerging, or imported infectious diseases are suspected, or 4) in cases of *Staphylococcus aureus* bacteremia or candidemia (Provision of information for background question).

***Rationale***

Several studies have reported an association between appropriate antimicrobial agent selection and reduced patient mortality [79]; therefore, the selection of initial antimicrobial agents which target the assumed causative microorganism is important. However, there is no consensus on which initial antimicrobial agents should be selected for the treatment of sepsis. Antimicrobial agents need to be selected according to the individual patient, which imposes a large burden on the treating physician. Raineri et al. compared infection treatment among ICU patients before and after initiating consultations regarding antimicrobial agent selection with infectious disease specialists and showed that both the selection rate of appropriate antimicrobial agents and the guideline compliance rate increased through consultations, and the mortality rate decreased [200]. Antimicrobial agent selection becomes more difficult when a particular cause of sepsis cannot be specified, when advanced drug-resistant bacteria are thought to be the culprits, and when emerging/re-emerging or imported infections with few opportunities for treatment are suspected. Therefore, consultations with infectious disease specialists or antimicrobial stewardship teams (ASTs) are expected to decrease the burden on the physician and increase the frequency of selection of appropriate antimicrobial agents.

Patients with sepsis often have bacteremia; however, some cases of bacteremia require careful examination of the source based on the bacterial species. *Staphylococcus aureus* bacteremia requires assessment with echocardiography tests for the complications of infectious endocarditis [88], whereas candidemia requires assessment for the complications of endophthalmitis [182]. Furthermore, the duration of antimicrobial administration needs to be set up according to the results of blood culture tests or the presence of the previously mentioned sources. However, not all clinical departments that treat patients with sepsis have sufficient knowledge or experience in this area. Numerous observational studies that investigated *Staphylococcus aureus* bacteremia reported that consulting with infectious disease specialists or ASTs improved the rate of compliance with guideline-based treatment (blood culture re-examination and echocardiography tests) and patient prognosis [201, 202]. Furthermore, observational studies on candidemia reported similar improvements in the rate of compliance with guidelines and patient prognosis [203–205]. These study results show that consulting with infectious disease specialists or ASTs, performing appropriate source assessment, and antimicrobial administration duration are valid for sepsis patients diagnosed with *Staphylococcus aureus* bacteremia or candidemia.

**CQ4-6: Should empirical antibacterial drugs for sepsis begin within 1 h upon identification of sepsis?**

***Answer:*** We suggest that antibacterial drugs be administered as soon as possible upon identification of sepsis or septic shock, but we suggest against using the target time of less than 1 h (GRADE 2C: certainty of evidence = “low”).

***Rationale***

The SSCG 2016 and the J-SSCG 2016 have both recommended that antimicrobial agents should be administered to patients with sepsis within 1 h based on the results of multiple observational studies, and this target is globally accepted. Large-scale cohort studies conducted at the state level in the United States after the 2016 guidelines was issued reported that the risk of death increased linearly according to the time from onset to the initiation of empiric antimicrobial therapy. It is necessary to evaluate whether the time frame of 1 h of sepsis recognition is worth recommending.

The results of a systematic review showed that there were no RCTs that conformed to the PICO criteria. A meta-analysis was conducted using seven observational studies [206–212]. The estimated value of effects relating to all-cause mortality obtained from the seven observational studies yielded a RD of 10 fewer per 1000 (95%CI: 23 fewer to 7 more), indicating that desired effects were limited. Undesired effects due to the early administration of antibacterial drugs did not occur within the evaluable range. Early administration of antibacterial drugs has the inherent risk of being administered to patients who really do not need them without sufficient evaluation, while the undesired effects due to this cannot be evaluated. It was determined that neither intervention nor the comparisons were predominant since the estimated value of effects of the RD relating to mortality rate is quite small and severe or serious harms due to intervention or the expected undesired effects could not be evaluated.

It should be noted that this recommendation does not imply the denial of the direction of administering appropriate antibacterial therapy that covers the expected target microorganism as soon as possible.

**CQ4-7: Should continuous or extended infusion of β-lactam antibiotics be used for sepsis?**

***Answer:*** We suggest using continuous or extended infusion of β-lactam antimicrobials (GRADE 2B: certainty of evidence = “moderate”).

***Rationale***

Antimicrobial agents are often administered intermittently to date; however, the continuous administration of time-dependent β-lactams or the extension of its administration times may be effective in terms of pharmacokinetics/pharmacodynamics.

The results of a systematic review showed that there were 13 RCTs which compared intermittent administration of β-lactams to either its continuous administration or the extension of its administration times among patients with sepsis or septic shock, and a meta-analysis of these RCTs was performed [213–225]. The estimated value of the effects on mortality (10 RCTs, *n* = 844) yielded a RD of 69 fewer per 1000 (95%CI: 135 fewer to 32 more), and the estimated value of the effects on clinical cures (9 RCTs, *n* = 886) yielded an RD of 113 more per 1000 (95%CI: 9 more to 241 more). The estimated value of the effects on the incidence of adverse effects (3 RCTs, *n* = 691) yielded an RD of 0 per 1000 (95%CI: 41 fewer to 59 more), and no increases in the incidence of adverse effects were found. The estimated value of the effects on the detection of drug-resistant bacteria (1 RCT, *n* = 198) yielded an RD of 18 fewer per 1000 (35 fewer to 72 more).

No special procedure is required for the continuous administration of antimicrobial agents or the extension of their time of administration. Although a syringe pump is required, this can be relatively easily performed at the ICU and will be well tolerated by healthcare professionals. Interventions are thought to be possible in many medical facilities. Few facilities perform continuous administration of antimicrobial agents or extend their times of administration, and there may be a need to educate nurses, obtain the cooperation and monitoring of pharmacy departments, and in-hospital consensus prior to implementation. Furthermore, the time of usage of medical resources needed for continuous administration (e.g., infusion pumps and syringe pumps) will also likely increase.

**CQ4-8: Should de-escalation antimicrobial therapy be used for sepsis?**

***Answer:*** We suggest applying de-escalation antimicrobial therapy for sepsis (GRADE 2D, certainty of evidence = “very low”).

***Rationale***

The desired effects of de-escalation strategy, such as the decreased use of broad-spectrum antimicrobial agents, decreased antimicrobial resistance or cost reduction, are unclear. With regard to the undesired effects of de-escalation interventions, one RCT (*n* = 116) [110] showed that the 90-day mortality rate was 78 more per 1000 (95%CI: 64 fewer to 335 more). On the other hand, the mortality rate due to long-term follow-ups in 13 observational studies (*n* = 3635) [226–238] was 80 fewer per 1000 (95%CI: 114 fewer to 40 fewer). The quality of the evidence for all of these was “very low.” The incidence of superinfections was 166 more per 1000 (95%CI: 8 more to 539 more) in the RCTs; however, no observational studies have evaluated these outcomes. Taken together, these results suggest that the undesired effects were trivial.

Based on the above, the desired effects of de-escalation strategy have not been evaluated, the mortality rate of the desired effects is difficult to evaluate, and there is a possibility of increased superinfections. Therefore, we considered that there is a slight undesired tendency in terms of the balance of effects. The certainty of the evidence across all outcomes is “very low.”

One small-scale RCT that evaluated the superinfection rate [110] showed that the incidence of superinfection was 16/59 (27%) in the intervention group and 6/57 (11%) in the control group; however, this was likely due to the extended total duration of antimicrobial administration in the intervention group. In other words, it has not been accurately evaluated whether increases in superinfection rates were due to de-escalation or an extended duration of antimicrobial administration. It was also reported that superinfections did not affect significant outcomes such as death. Extending the duration of antimicrobial administration due to antimicrobial de-escalation was dissociated from standard clinical practice. Furthermore, de-escalation is recommended in terms of antimicrobial stewardship and is a widely used practice. Therefore, we concluded that it is difficult to recommend against de-escalation based on the aforementioned evidence.

De-escalation strategy is a widely accepted and rationalized treatment modality, and the only intervention is changing the antimicrobial agents, which can be performed without problems in many medical facilities. However, care must be taken not to extend the total duration of antimicrobial administration when de-escalation is performed.

**CQ4-9: Should procalcitonin be used as an indicator for stopping antimicrobial therapy for sepsis?**

***Answer:*** We suggest using procalcitonin as an indicator for stopping antimicrobial therapy for sepsis (GRADE 2B, certainty of evidence = “moderate”).

***Rationale***

A systematic review of studies which compared procalcitonin-guided termination of antimicrobial drugs (intervention group) to termination based on the physician’s decision or protocols which did not include procalcitonin (control group) among patients with sepsis or septic shock was performed. A meta-analysis of the extracted RCTs [239–250] showed that the estimated value of effects for 28-day mortality outcomes during intervention (5 RCTs, *n* = 2867) was 42 fewer per 1000 (95%CI: 69 fewer to 11 fewer), and that of in-hospital mortality outcomes (9 RCTs, *n* = 2422) was 50 fewer per 1000 (95%CI: 79 fewer to 18 fewer). Outcomes for the number of days of antimicrobial drug administration (3 RCTs: *n* = 231) yielded a MD of 1.16 days shorter (95%CI: 2.33 shorter to 0) compared to the intervention group. Meanwhile, the estimated value of effects for sepsis recurrence as an outcome (4 RCTs: *n* = 261) yielded a MD of 8 more per 1000 (95%CI: 27 fewer to 113 more). The undesired effects were trivial since the confidence interval was close to the threshold for clinical decisions. Therefore, desired effects were present in 28-day mortality rate and in-hospital mortality rate, whereas undesired effects were unclear, and the certainty of the evidence was “moderate”. However, there is insufficient research based on which to make a decision on the recurrence of sepsis, detection of drug-resistant bacteria, and the number of days of antimicrobial drug administration, as well as the limited number of facilities from which one can promptly obtain procalcitonin measurement results.

**CQ4-10: Should relatively short-term (i.e. within 7 days) antimicrobial therapy be applied for sepsis?**

***Answer:*** We suggest applying relatively short-term (i.e. within 7 days) antimicrobial therapy for sepsis (GRADE 2D: certainty of evidence = “very low”).

***Rationale***

We performed a systematic review of RCTs which compared antimicrobial agents administered within 7 to 8 days and more than 7 to 8 days on sepsis or infections requiring intensive treatment (excluding those requiring long-term treatment of more than four weeks such as endocarditis and purulent osteomyelitis). There were three studies on ventilator-associated pneumonia and one study on intra-abdominal infection among studies on sepsis or infections requiring critical care; however, there was no research involving multiple infections simultaneously [251–254]. Meta-analyses of these four studies showed that the RD of 28-day mortality (3 RCTs, *n* = 804) was 12 more per 1000 (95%CI: 34 fewer to 78 more); that of mortality during maximum follow-up (4 RCTs, *n* = 1029) was 11 more per 1000 (95%CI: 27 fewer to 62 more). The RD of clinical cures (2 RCTs, *n* = 392) was 50 fewer per 1000 (95%CI: 202 fewer to 144 more); that of new events (recurrence and reinfection) (3 RCTs, *n* = 862) was 77 more per 1000 (95%CI: 0 to 185 more). Detection of drug-resistant organisms was evaluated in two RCTs, with an RD of 132 fewer per 1000 (95%CI: 292 fewer to 166 more). Both the benefits and harms were low, and the certainty of the overall evidence was “very low”. There were limited evidence available on sepsis or infections requiring intensive treatment.

**CQ4-11: What should be used as a reference for adjusting the dose for renal-excretion antimicrobial drugs?**

***Answer:*** Changes in bodily fluid volume and the presence of renal replacement therapy and other extracorporeal circulation therapies in addition to renal function test values (e.g., serum Cr level, eGFR level) measured at multiple time points are informative (Provision of information for background question).

***Rationale***

Since the decrease in clearance of renally excreted antimicrobial agents induces an increase in blood concentrations in case of renal injury, it is necessary to adjust the dose of antimicrobials among patients with renal injury with sepsis [255–258]. Care must be taken in these cases as the antimicrobial drug concentration, particularly in the initial stage of sepsis, may be insufficient considering the recommended doses for each renal function set for general renal injury [259, 260].

Creatinine (Cr) levels calculated based on age and sex are generally used as indicators of renal function as well as the estimated glomerular filtration rate (eGFR). Meanwhile, Cr levels are known to change with a delay of 24–48 h following sudden fluctuations in GFR and have a high possibility of not accurately reflecting the true renal function in acute disease states. Therefore, the GFR should be predicted using multiple measurements of Cr levels as references. In other words, the true GFR should be assumed to be smaller than the eGFR if Cr levels have a tendency to increase, and larger than the eGFR if Cr levels have a tendency to decrease [261].

Furthermore, the dose of antimicrobial agents based on renal function assessments using Cr and eGFR may be insufficient due to changes as shown in items (1) and (2) below among patients with sepsis. Therefore, it is important to obtain variations in body fluid volume, particularly in the administration of water-soluble antimicrobial agents (β-lactams, aminoglycosides, glycopeptides, linezolid, colistin, triazoles, echinocandins, and polyene macrolides) [262–272].

1. Capillary leakage and edema, fluid therapy, pleural and ascitic fluid, drainage of fluid, hypoalbuminemia, increases in distribution volume associated with decreased protein binding rate, and dilution of antimicrobial agents in plasma and extracellular fluid
2. Increased cardiac output, increased renal blood flow, augmented renal clearance due to vasodilation, capillary leakage, and hypoalbuminemia

Antimicrobial drug concentrations are also influenced by extracorporeal circulation [270]. In extracorporeal membrane oxygenation (ECMO), the changes in distribution volume and antimicrobial drug clearance caused by the capture of antimicrobial agents in the circuit, and ECMO induced inflammation have been indicated [273–275].

Furthermore, antimicrobial drug concentrations also change when renal replacement therapy is initiated [276–282]. The changes vary with the setting of renal replacement therapy [283–286]; however, it has been pointed out that the doses recommended for renal replacement therapy may be insufficient [284, 287–293].

**CQ5: Intravenous immunoglobulin therapy**

**Introduction**

Polyclonal immunoglobulin possesses various biological characteristics, including neutralization of pathogenic microorganisms and their toxins, promotion of phagocyte/bacteriolysis via complement activation, opsonization, antibody-dependent cellular cytotoxicity, non-specific anti-inflammatory actions, and inhibition of inflammatory cytokine production [294]. Intravenous immunoglobulin (IVIG) use has been recommended for several immunological diseases, including idiopathic thrombocytopenic purpura, myasthenia gravis, chronic inflammatory demyelinating polyneuritis, Guillain-Barré syndrome, and Kawasaki disease, in several guidelines. For infectious diseases, in addition to the above-mentioned biological activities, since hypogammaglobulinemia was frequently observed in sepsis [295], IVIG has been administered to patients with severe conditions.

In Japan, IVIG for severe infectious diseases is covered by national insurance based on the positive results of a clinical trial by Masaoka et al. [296] A prospective observational study conducted by the JAAM from 2010 to 2011 showed that IVIG was administered to 34.6% of patients with severe sepsis and 44.0% of patients with septic shock [297]. Among sepsis guidelines, the SSCG 2016 [1, 2] recommended against IVIG use, and the J-SSCG 2016 [3, 4] could not present a recommendation, since the agreement rate of the committee for the “weak recommendation” proposed by the managing group was low. Meanwhile, some medical treatises recommend IVIG for specific infectious diseases in which bacterial toxins contribute to their pathophysiology, such as streptococcal toxic shock syndrome (STSS), Staphylococcal toxic shock syndrome (TSS), and necrotizing soft tissue infection [298, 299] and IVIG use for them needs to be considered.

In the current guidelines, systematic reviews on all extracted RCTs and RCTs with a low risk of bias (RoB) were performed for CQ5–1, and the latter was adopted based on pre-determined settings. As a result, the above-mentioned RCT conducted by Masaoka et al. was not included in the latter systematic review. Further, we added CQs for IVIG administration against specific pathogens such as STSS (CQ5–2-1) and TSS (CQ5–2-2).

**Fig. 4:**
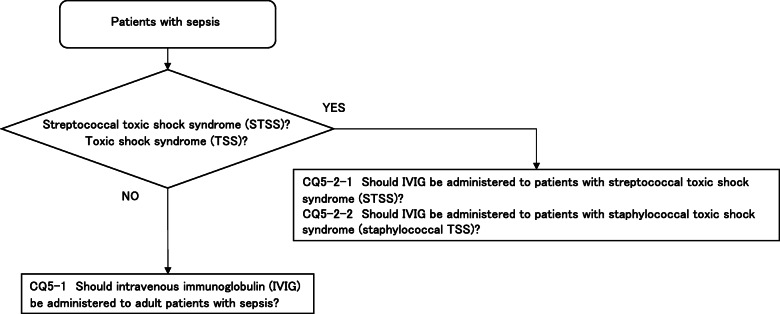
CQ5: Intravenous immunoglobulin therapy (clinical flow)

Clinical flow of these CQs is shown in Fig. 4.

**CQ5-1: Should intravenous immunoglobulin (IVIG) be administered to adult patients with sepsis?**

***Answer:*** We suggest against administering IVIG to patients with sepsis (GRADE 2B: certainty of evidence = “moderate”).

***Rationale***

The outcomes of this CQ were all-cause mortality, length of ICU stay, and all serious adverse effects. Two systematic reviews were performed on all extracted RCTs and RCTs with low RoB for all-cause mortality, and the latter was adopted based on predetermined settings. The results of an systematic review yielded 9 RCTs that conformed to the PICO criteria [296, 300–307], and a meta-analysis was performed using these trials.

The estimate of effect for all-cause mortality obtained from the 3 RCTs with a low RoB yielded a RD of 7 more per 1000 (95%CI: 58 fewer to 83 more), and that for length of ICU stay yielded a MD of 1.1 days shorter (95%CI: 5.44 shorter to 3.25 longer). Based on these results, the desirable effects were judged as trivial. The estimate of effect for all serious adverse effects yielded an RD of 1 fewer per 1000 (95%CI: 23 fewer to 46 more), and the undesirable effects were judged as trivial. In summary, the desirable and undesirable effects were both trivial. Therefore, the balance of effects did not support either the intervention or the comparison. Based on the above judgement, we proposed a weak recommendation not to use IVIG for sepsis to the committee. This proposal was adopted by voting based on the modified RAND method, with a median of 8 and a DI of 0.178 (7 points or more: 87.5%).

**CQ5-2-1: Should IVIG be administered to patients with streptococcal toxic shock syndrome (STSS)?**

***Answer:*** We suggest administering IVIG to patients with STSS (GRADE 2D: certainty of evidence = “very low”).

***Rationale***

During the systematic review process, only one RCT with a sample size of 18 patients targeting STSS was found, and considering the low incidence of STSS, it is unlikely that a large-scale RCT will be conducted in the future. Therefore, although exceptional, we additionally performed a systematic review of the observational studies for this CQ, The outcomes of this CQ were all-cause mortality, length of ICU stay, and all serious adverse effects. For all-cause mortality, systematic reviews of all extracted RCTs/observational studies, and systematic reviews of RCTs/observational studies limited to clindamycin-treated cases were performed, and it was set in advance to adopt the one with a lower RoB. The results of the systematic review yielded 1 RCT [303] and four observational studies [308–311] that conformed to the PICO criteria, and a meta-analysis was performed on each of these. For all-cause mortality rate, RoB of the systematic review limited to CLDM-treated cases was lower, and thus was adopted for making a recommendation. The estimate of effect for all-cause mortality obtained from 1 RCT yielded a RD of 174 fewer per 1000 (95%CI: 285 fewer to 684 more), indicating the desirable effects of IVIG administration were limited. Meanwhile, the estimate of effect for all-cause mortality obtained from observational studies yielded an RD of 143 fewer per 1000 (95%CI: 214 fewer to 18 fewer), indicating significant desirable effects of IVIG administration.

The length of ICU stay was unassessable due to the lack of studies used for outcomes. From the above results, we judged that the small desirable effects could be expected. All serious adverse effects were also unassessable due to the lack of studies. However, considering the systematic review results of sepsis (CQ5–1), we judged that the undesirable effects were trivial. In summary, the desirable effects were small, whereas the undesirable effects were trivial. Therefore, the balance of effects was judged as probably favoring the intervention. Based on the above judgement, we proposed a weak recommendation to use IVIG for STSS to the committee. This proposal was adopted by voting based on the modified RAND method, with a median of 7.5 and a DI of 0.164 (7 points or more: 75%).

**CQ5-2-2: Should IVIG be administered to patients with staphylococcal toxic shock syndrome (staphylococcal TSS)?**

***Answer:*** We suggest against administering IVG to patients with staphylococcal TSS (expert consensus: insufficient evidence).

***Rationale***

The outcomes of this CQ were all-cause mortality, length of ICU stay, and all serious adverse effects. As a result of systematic review, neither RCT nor observational study matching PICO criteria was found. The desirable effects could not be evaluated, and although some experts recommend the use of IVIG for staphylococcal TSS based on the hypothesis that bacterial toxins play major roles in inducing severe pathological conditions, we judged that the desirable effects were trivial. The undesirable effects also could not be evaluated but based on systematic review results of sepsis (CQ5–1), we judged that the undesirable effects were trivial. In summary, the desirable and undesirable effects were both trivial, and therefore the balance of effects did not support either intervention or the comparison. Based on the above judgement, we proposed a weak recommendation not to use IVIG for staphylococcal TSS to the committee, and this proposal was adopted by voting based on the modified RAND method, with a median of 7 and DI of 0.164 (7 points or more: 75%).

**CQ6: Initial resuscitation/inotropes**

**Introduction**

We presented “CQ6-1: Should echocardiography be conducted in patients with sepsis?” after considering that it is necessary to evaluate cardiac function and hemodynamics to promptly and appropriately enact treatment strategies for septic shock. We presented “CQ6-2: Is early goal directed therapy (EGDT) recommended for initial resuscitation in patients with sepsis?” to re-evaluate the usefulness of EGDT. We presented “CQ6-3: Should vasopressors be used simultaneously or in the early stage (within 3 h) of initial fluid resuscitation in adult patients with sepsis?” to determine the timing of administration of vasopressor drugs in cases in which organ perfusion pressure cannot be maintained with initial fluid resuscitation. We presented “CQ6-4: Should lactate levels be used as an indicator for initial resuscitation in adult patients with sepsis?” because the mixed venous oxygen saturation is an indicator that expresses the balance between tissue oxygen supply and demand, and serum lactate levels have generally been used as an indicator of anaerobic metabolism. We presented “CQ6-5: What is the initial fluid infusion rate and volume in adult patients with sepsis?” as a BQ since the initial fluid infusion rate and volume were thought to be important. We presented “CQ6-6: How should fluid responsiveness be assessed in adult patients with sepsis?” as a BQ because it is desirable to use multiple monitoring systems to predict responsiveness to fluid replacement. We presented “CQ6-7: Should albumin solution be used for initial resuscitation in adult patients with sepsis?” and “CQ6-8: Should artificial colloids be used for initial resuscitation in adult patients with sepsis?” regarding the use of albumin or artificial colloids as initial fluids for resuscitation. We presented “CQ6-9-1, CG6-9-2: Should noradrenaline, dopamine, or phenylephrine be used as a first-line vasopressor in adult patients with sepsis?” regarding the first-line vasopressor to be used in the initial fluid resuscitation of patients with sepsis. We presented “CQ6-10-1: Should adrenaline be used as a second-line vasopressor in adult patients with sepsis?” and “CQ6-10-2: Should vasopressin be used as a second-line vasopressor in adult patients with sepsis?” regarding second-line treatments when the pressor effects of noradrenaline are insufficient. We presented “CQ6-11: Should inotropes be used in adult patients with sepsis accompanied by cardiogenic shock?” regarding the use of inotropic drugs for cardiac dysfunction in septic shock. We presented “CQ6-12: Should β-blockers be used in adult patients with sepsis?” regarding the use of β-adrenergic receptor blockers to control the heart rate of patients with tachycardia associated with septic shock. We presented “CQ6-13: What are the indications of assisted circulation in adult patients with septic shock?” as a BQ for adult patients with sepsis presenting with severe cardiac dysfunction.

We hope that the CQs and answers on initial fluid resuscitation and circulatory agonists will be utilized together with the medical care flow chart presented in this guideline.

**Fig. 5:**
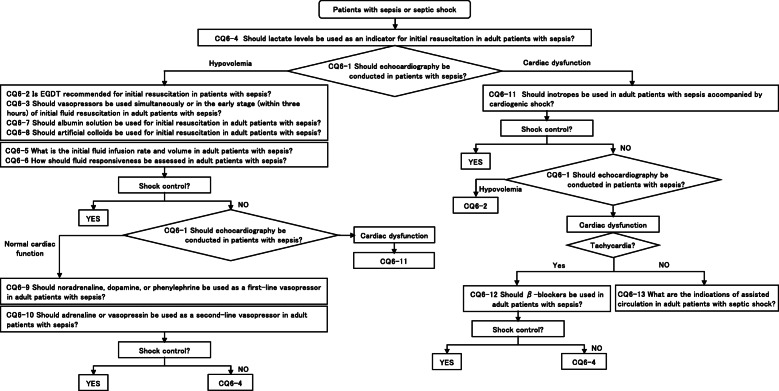
CQ6: Initial resuscitation/inotropes (clinical flow)

Clinical flow of these CQs is shown in Fig. 5.

**CQ6-1: Should echocardiography be conducted in patients with sepsis?**

***Answer:*** We suggest, following initial fluid resuscitation, conducting cardiac function and hemodynamics assessments with echocardiography in patients with sepsis/septic shock (GRADE 2D: certainty of evidence = “very low”).

***Rationale***

Sepsis and septic shock are conditions in which the main cause is distributive shock associated with peripheral vasodilation. In addition, hypovolemia and shock due to decreased cardiac function (hypovolemic shock and cardiogenic shock) can also be complications and result in a complicated pathological condition. Therefore, it is clinically important to evaluate the cardiac function and hemodynamics using echocardiography at the time of initial resuscitation; thus, this was brought up as an important clinical issue. The results of our systematic review yielded 1 RCT that was a feasibility study as an example of a study that conformed to the PICO criteria [312], and a meta-analysis using this study was performed.

The estimated effect of short-term mortality outcomes (1 RCT, *n* = 30) was 134 more per 1000 (95%CI: 104 fewer to 952 more), and that of the outcome of length of stay in the ICU (1 RCT, *n* = 30) was a MD of 0.3 days shorter (95%CI: 4.46 shorter to 3.86 longer). However, both the number of studies and the sample size were insufficient; thus, it was decided that the effects could not be determined. It was also decided that undesired effects could not be determined since in the RCT obtained in this search such investigations were not conducted. In this CQ, the control groups tended to predominate as regards to short-term mortality, and interventions tended to predominate as regards to the length of stay in the ICU. However, the study obtained in this search was a single RCT with a small sample size; thus, the balance of effects could not be determined.

However, echocardiography is a non-invasive and simple test that imposes minimal burdens on the patient; therefore, we suggest that cardiac function and hemodynamics should be assessed among patients with septic or septic shock during initial resuscitation using echocardiography.

**CQ6-2: Is EGDT recommended for initial resuscitation in patients with sepsis?**

***Answer:*** We suggest against conducting EGDT as initial resuscitation in patients with sepsis/septic shock (GRADE 2C: certainty of evidence = “low”).

***Rationale***

Initial resuscitation plays an important role in maintaining acute organ perfusion in patients with sepsis and septic shock. We aimed to verify the usefulness of EGDT, which sets a specific method for initial resuscitation that indicates the basis of sepsis treatment; thus, this was taken up as a CQ. A systematic review yielded four RCTs that conformed to the PICO criteria; thus [313–316], a meta-analysis was performed using these studies.

The estimated value of the effects of short-term mortality outcome (4 RCTs, *n* = 3993) yielded 8 fewer per 1000 (95%CI: 32 fewer to 17 more), that of long-term mortality outcome (3 RCTs, *n* = 3648) was 5 fewer per 1000 (95%CI: 31 fewer to 26 more), that of the outcome of length of stay in the ICU (3 RCTs, *n* = 3737) yielded a MD of 0.22 days longer (95%CI: 0.13 shorter to 0.58 longer), and it was adjudged that the desired effects of initial resuscitation with EGDT were limited. The estimated value of the effects of various serious adverse effects (3 RCTs, *n* = 3734) was one more per 1000 (95%CI: 19 fewer to 32 more), and it was adjudged that the undesired effects of initial resuscitation with EGDT were limited. The net balance between desired and undesired effects was predominant for interventions by 12 per 1000, and the balance of effects may slightly favor EGDT interventions over control when considering the relative value of short-term and long-term mortality outcomes. However, the harms were greater for 44 per 1000 when the uncertainty of mortality outcomes was considered, and the worst values in the confidence intervals were used. Therefore, it was adjudged that neither the intervention nor comparative controls were predominant.

The central venous pressure and central venous oxygen saturation need to be monitored, and red blood cells need to be transfused in order to administer the standard EGDT. Modified EGDT, which is less invasive and burdensome, is currently advocated. The standard EGDT is considered unacceptable due to the burdens imposed on medical staff and patients, and we suggest against administering EGDT as initial resuscitation in patients with sepsis or septic shock.

**CQ6-3: Should vasopressors be used simultaneously or in the early stage (within 3 h) of initial fluid resuscitation in adult patients with sepsis?**

***Answer:*** We suggest administering vasopressors simultaneously or in the early stages (within 3 h) of initial fluid resuscitation in patients with sepsis/septic shock who have difficult maintaining hemodynamics (GRADE 2C: certainty of evidence = “low”).

***Rationale***

Vasopressor administration is necessary in patients with sepsis or septic shock if organ perfusion cannot be maintained after initial fluid resuscitation. However, the optimal timing to initiate vasopressors is unclear. A multi-center RCT conducted by Macdonald et al. [317] examined a regimen of restricted fluids and early vasopressors and a single-center blinded RCT conducted by Permpikul et al. [318] compared continuous norepinephrine infusion at 0.05 μg/kg/min to placebo among patients with septic shock within 1 h of onset. A meta-analysis was performed using these 2 RCTs (*n* = 409). The early use of vasopressors decreased the incidence of pulmonary edema (RD 104 fewer per 1000, 95%CI: 145 fewer to 39 fewer); however, there was no difference in the mortality rate. The estimated value of the effects of myocardial ischemia was 15 more per 1000 (95%CI: 9 fewer to 95 more), although other forms of organ ischemia were not evaluated as adverse events. Based on the above, the balance of benefits and harm was judged as “intervention likely superior”.

**CQ6-4: Should lactate levels be used as an indicator for initial resuscitation in adult patients with sepsis?**

***Answer:*** We suggest using lactate levels as an indicator of tissue hypoperfusion during initial resuscitation in patients with sepsis/septic shock (GRADE 2C: certainty of evidence = “low”).

***Rationale***

Initial resuscitation plays an important role in maintaining acute organ perfusion among patients with sepsis or septic shock. However, there is no consensus on what a good indicator for confirming the maintenance of organ perfusion is. Searching for an optimal evaluation indicator is a clinically important issue, and so was taken up as a CQ.

The results of a systematic review yielded 5 RCTs [319–323]. Hernández et al. [319] evaluated whether using lactate or peripheral circulation as indicators for initial resuscitation improved the mortality rate among adult patients with early septic shock. Jansen et al. [320] evaluated whether using lactate or factors other than lactate (e.g., central venous oxygen saturation [ScvO_2_] and peripheral circulation) as indicators for initial resuscitation improved the mortality rate among patients with hyperlactatemia (more than 3.0 mmol/L) during admission on the ICU. Jones et al. [321] evaluated whether initial resuscitation with either lactate clearance or ScvO_2_ as indicators improved the in-hospital mortality rate. Puskarich et al. [322] evaluated whether lactate clearance or ScvO_2_ as indicators for initial resuscitation improved the mortality rate among patients with sepsis. Zhou et al. [323] evaluated whether lactate clearance or ScvO_2_ as indicators for initial resuscitation improved the mortality rate among patients with hyperlactatemia due to sepsis.

Initial resuscitation with lactate as an indicator resulted in a short-term mortality of 62 fewer per 1000, a long-term mortality rate of 21 fewer per 1000, and a MD of 0.03 days longer for ICU length of stay when compared to initial resuscitation using factors other than lactate as indicators. Meanwhile, the MD for serious adverse effects (SOFA score after 72 h) was 0.04 higher. Based on the above, the balance of effects was judged such that the “initial resuscitation with lactate as an indicator is likely superior”.

The amount of blood needed to measure lactate levels is minimal in practice; however, consideration should be given to the risk of anemia due to frequent blood sampling.

**CQ6-5: What is the initial fluid infusion rate and volume in adult patients with sepsis?**

***Answer:*** There is an opinion that the initial fluid resuscitation in patients with reduced intravascular volume due to sepsis should be administered over 30 mL/kg of crystalloid solution within 3 h, aiming to optimize the circulating blood volume. It is important during initial fluid resuscitation to carefully observe vital signs and to avoid excessive fluid loads by using lactate clearance and echocardiography while conducting tissue oxygen metabolism and hemodynamics assessments (Provision of information for background question).

***Rationale***

In the J-SSCG 2016 [3, 4], it was stated that “patients with tissue hypoperfusion and decreased intravascular volume due to sepsis should receive more than 30 mL/kg of the crystalloid solution”. In SSCG 2016 [1], it was stated that “administering at least 30 mL/kg of crystalloid solution within the first 3 h is recommended for the resuscitation of patients with hypoperfusion caused by sepsis”. In three recently conducted large-scale RCTs (the ProCESS [315], ARISE [316], and ProMISe [314] trials), the researchers administered initial fluid resuscitation prior to the start of the protocol (i.e., before randomization), comprising 2.1–2.3 L in the ProCESS trial, 2.5–2.6 L in the ARISE trial, and 1.9–2.0 L in the ProMISe trial (approximately 30 mL/kg). The concept of early high-dose fluid therapy (30 mL/kg) against septic shock has already become commonplace, and it was thought that early goal-directed therapy administered subsequent to initial fluid resuscitation in the previously mentioned large-scale RCTs were not found to be useful. Meanwhile, Boyd et al. [324] indicated the harmful effects of fluid overload, and Murphy et al. [325] reported that fluid restriction could lead to an improved prognosis. A systematic review of 15 studies (*n* = 31,443) on septic shock [326] showed that excess fluid balance increased the mortality risk by 70% (pooled RR 1.70, 95%CI: 1.20 to 2.41, *P* = 0.003). However, those who received large volumes of fluid infusions within 3 h after the onset of sepsis (2085 mL vs. 1600 mL, *P* = 0.007) showed an improved in-hospital mortality rate (OR 0.34, 95%CI: 0.15–0.75, *P* = 0.008). In an observational study of 1032 patients with septic shock, Kuttab et al. [327] reported that the in-hospital mortality rate significantly increased when it was not possible to administer 30 mL/kg of initial fluid resuscitation within 3 h of the onset of sepsis (OR 1.52, 95%CI: 1.03–2.24). Meanwhile, Wardi et al. [328] recommended that an initial fluid volume of less than 30 mL/kg should be administered to patients with septic shock with complications of heart failure with an ejection rate of less than 40%. There is no high-quality evidence for the initial fluid resuscitation rate or amount for sepsis/septic shock. There is also no evidence currently that rejects the concepts of compensating the relatively decreased circulating blood volume, improving tissue hypoperfusion, and balancing oxygen demand/supply promptly. An important principle is to continuously evaluate treatment effects, carefully observe vital signs during initial fluid resuscitation, and evaluate tissue oxygen metabolism and hemodynamics using lactate clearance and echocardiography, while avoiding fluid overload.

**CQ6-6: How should fluid responsiveness be assessed in adult patients with sepsis?**

***Answer:*** Fluid responsiveness is significant increase in stroke volume (SV) after fluid infusion, and multiple parameters, including static and dynamic parameters, should be used to predict fluid responsiveness. Static parameters, including central venous pressure (CVP) and pulmonary capillary wedge pressure (PCWP), are measured at a point. Dynamic parameters include changes in cardiac output by passive leg raising (PLR) and fluid challenge, pulse pressure variation (PPV) and stroke volume variation (SVV) during mechanical ventilation (Provision of information for background question).

***Rationale***

Fluid responsiveness reflects a significant increase in cardiac output or stroke volume when 250–500 mL of fluid is administered and is defined by an increase of at least 10–15% [329, 330]. Monitoring parameters used for predicting fluid responsiveness can be divided into static and dynamic parameters. Static parameters are biometric information at a given point and include central venous pressure (CVP), pulmonary capillary wedge pressure (PCWP), global-end diastolic volume (GEDV), and intrathoracic blood volume (ITBV) based on transpulmonary thermal dilution methods. Dynamic parameters are methods that evaluate variation using some type of intervention and include changes in cardiac output based on passive leg raising (PLR) or fluid challenges, changes in stroke volume based on the end-expiratory occlusion test (EEO), pulse pressure variation (PPV) using pre-load respiratory variation induced by mechanical ventilation, stroke volume variation (SVV), and variation in the inferior vena cava (IVC) or superior vena cava (SVC) (see Table 3 for reference).

The static parameters CVP and PCWP were evaluated as reflecting fluid responsiveness when CVP was below 8 mmHg or PCWP was less than 12 mmHg, but their reliability was low. GEDV and ITBV could be measured by transpulmonary thermal dilution techniques with rapid infusion of cold water and can be used as a pre-load parameter [331]. Moreover, it provides pulmonary extravascular water content and reflects the pulmonary vascular permeability index. However, the reliability of evaluations of fluid responsiveness is reported to be low [332].

Dynamic parameters are better at predicting fluid responsiveness than static parameters [333]. However, there are few cases in which these can be applied in clinical settings. PPV and SVV are evaluated as reflecting fluid responsiveness if a variation of more than 12% due to positive pressure ventilation is seen when the tidal volume is more than 8 mL/kg without spontaneous breathing. The variation is likely to become larger if there is spontaneous breathing, arrhythmia, increased intra-abdominal pressure, or right heart failure. These variations also decrease in patients with tachycardia or undergoing lung protective ventilation [334]. The PPV has also been reported to be smaller when lung compliance is low [335]. Evaluation of fluid responsiveness using echocardiography includes variations in IVC and SVC diameter-based breathing, which are better predictors of fluid responsiveness than CVP [336]. It has been reported that the SVC diameter is a better parameter than the IVC diameter [337], but evaluating the respiratory variation in the diameter of the SVC requires transesophageal echocardiography and is more invasive. Evaluations based on echocardiography are likely to be discordant among operators compared to other monitoring methods. Respiratory variations in IVC diameter are less reliable when compared to PPV or SVV [338] and should not be prioritized when PPV or SVV can be used. PLR involves an evaluation of the increased cardiac output based on lower limb elevation, and the lower limb elevation-based pre-load corresponds to approximately 250–350 mL of fluid [339]. PLR is evaluated as reflecting fluid responsiveness if an increase in cardiac output of more than 10% is observed. PLR is also useful in patients with spontaneous breathing or arrhythmia [340]. Pre-load increases due to lower limb raising are dependent on the vascular resistance of the venous system and is thus affected by vasoactive drugs and increased intra-abdominal pressure [341]. The EEO is a test that temporarily occludes the airways at the exhalation terminal of mechanical ventilation, during which venous return increases because the intrathoracic pressure does not increase without ventilation. Occlusion is performed for 15 s, and this is evaluated as reflecting fluid responsiveness if an increase in cardiac output of more than 5% is observed [342]. The EEO requires tracheal intubation and ventilator management and cannot be performed among patients who cannot undergo the EEO for over 15 s due to spontaneous breathing [343]. It has been reported that the EEO is more reliable than the PPV among patients with decreased lung compliance [344]. However, validation in the prone position has not been confirmed [345]. Cardiac output should be evaluated before and after fluid loading when none of the above can be used. Low fluid volumes indicate the potential influence of measurement errors, whereas high fluid volumes increase the risk of fluid overload. It has also been reported that improved hemodynamics were temporary in approximately half of the patients who showed fluid responsiveness by fluid loading [346]. Thus, there is a need to continuously evaluate whether further fluid administration is necessary while confirming findings consistent with hypoperfusion.

**CQ6-7: Should albumin solution be used for initial resuscitation in adult patients with sepsis?**

***Answer:*** We suggest against administering albumin solution as a standard treatment at the beginning of initial fluid resuscitation in patients with sepsis (GRADE 2C: certainty of evidence = “low”). Albumin solution can be used in patients with sepsis when patients do not respond to standard treatment and require substantial amounts of crystalloids (expert consensus: insufficient evidence).

***Rationale***

Initial fluid resuscitation is an important intervention in patients with sepsis or septic shock. However, there is no consensus as to whether albumin should be used as a standard infusion preparation. Clarifying whether to use albumin as a standard infusion preparation for initial fluid resuscitation is a clinically important issue; thus, this was taken up as a CQ.

The results of a systematic review yielded three RCTs [347–349]. Rackow et al. [347] compared the effectiveness of 5% albumin, 6% hetastarch, and saline solutions in patients with hypovolemic shock and septic shock. Finfer et al. [348] compared the effectiveness of 4% albumin and saline solutions in the initial resuscitation of patients with severe sepsis. Van der Heijden et al. [349] compared the effectiveness of 5% albumin, 6% hydroxyethyl starch, 4% gelatin, and saline solutions in the management of severely septic/non-septic patients with hypovolemia.

Initial resuscitation using albumin preparations resulted in 45 fewer per 1000 as regards short-term mortality and a MD of 0.7 days longer for the length of stay in the ICU. Meanwhile, serious adverse effects (pulmonary injury score) yielded an MD of 0.75 higher. The pulmonary injury score was determined on a scale of 0–4, with severe pulmonary injury adjudged to be present with a score of 2.5 or higher. Based on the above, the balance of effects was adjudged as “the effects of initial resuscitation using albumin preparations are neither superior nor inferior to initial resuscitation using other infusion preparations”.

The costs and infection risks of albumin preparations are often a concern in practice. There have been no investigations that set these as outcomes in this CQ; thus, it should be noted that among some groups of patients, albumin preparations might be beneficial or harmful.

**CQ6-8: Should artificial colloids be used for initial resuscitation in adult patients with sepsis?**

***Answer:*** We suggest against administering artificial colloids in patients with sepsis/septic shock (GRADE 2D: certainty of evidence = “very low”).

***Rationale***

Determining what fluid to use for initial resuscitation among patients with septic shock is an extremely important problem. However, as there is no consensus on whether to use artificial colloids as standard infusion during initial resuscitation, this was taken up as a clinically important issue. The results of a systematic review yielded four RCTs that conformed to the PICO criteria [350–353], and a meta-analysis was performed using these studies. The estimated value of the effects of short-term mortality outcomes (4 RCTs, *n* = 2586) was 9 more per 1000 (95%CI: 25 fewer to 46 more), and that of long-term mortality outcomes (3 RCTs, *n* = 2545) was 19 more per 1000. That of the outcome of length of stay in the ICU (2 RCTs, *n* = 214) yielded a MD of 1.13 days shorter (95%CI: 8.28 shorter to 6.03 longer). Based on the above, it was adjudged that the desired effects due to artificial colloid administration were trivial. The estimated value of the effects of outcomes of dialysis use associated with AKI yielded a RD of 16 more per 1000 (95%CI: 24 fewer to 71 more) (4 RCTs, *n* = 3891) and that of severe hemorrhage yielded an RD of 42 more per 1000 (95%CI: 3 more to 97 more) (2 RCTs, *n* = 994). Based on the above, it was adjudged that the undesired effects of artificial colloid administration were moderate. The net balance of benefits and harms was higher for the latter by 86 per 1000. Even when considering the uncertainty for short-term mortality, using the minimum values of the CI (25 fewer per 1000), and setting the relative value of outcomes relating to death at three times that of other outcomes, the harms exceeded the benefits by two per 1000. Therefore, the balance of effects was adjudged such that the “comparative control is likely superior” based on which we suggest against the administration of artificial colloids in patients with sepsis or septic shock.

**CQ6-9-1: Should noradrenaline, dopamine, or phenylephrine be used as a first-line vasopressor in adult patients with sepsis? noradrenaline vs. dopamine.**

***Answer:*** Between noradrenaline and dopamine, we suggest administering noradrenaline as a first-line vasopressor in adult patients with sepsis (GRADE 2D: certainty of evidence = “very low”).

***Rationale***

The J-SSCG 2016 and the SSCG 2016 recommended noradrenaline as a first-line vasopressor for the initial resuscitation of patients with sepsis. However, the SSCG 2016 also suggested the use of dopamine in patients without tachycardia. Vasopressor selection is important in the initial resuscitation of patients with sepsis; thus, the decision to administer either noradrenaline or dopamine as a first-line vasopressor was taken up as a CQ.

Five RCTs [354–358] were included in the meta-analysis as a result of a systematic review. Only the RCT conducted by De Backer et al. [358] included shock patients with or without sepsis, while the other RCTs compared noradrenaline and dopamine in the treatment of patients with septic shock. Noradrenaline administration resulted in a short-term mortality of 54 fewer per 1000 compared to that of dopamine administration. The incidence of arrhythmia events decreased by 110 per 1000. Meanwhile, the incidence of limb ischemia events increased by 3 per 1000 that of myocardial ischemia events increases by 8 per 1000, and that of mesenteric ischemia events decreased by 6 per 1000. The net benefit of noradrenaline was 187 per 1000 and was higher for the desired effects. Even when considering the uncertainty of mortality outcomes and using the worse values in the confidence intervals, the net benefit was 133 per 1000 in favor of desired effects. Based on the above, the balance of benefits and harms was adjudged such that “noradrenaline administration is likely superior”.

Caution is required in actual clinical cases in which the incidences of organic ischemic complications are expected to increase due to noradrenaline administration, due to underlying diseases among patients.

**CQ6-9-2: Should noradrenaline, dopamine, or phenylephrine be used as a first-line vasopressor in adult patients with sepsis? noradrenaline vs. phenylephrine.**

***Answer:*** Between noradrenaline and phenylephrine, we suggest administering noradrenaline as a first-line vasopressor in adult patients with sepsis (GRADE 2D: certainty of evidence = “very low”).

***Rationale***

The J-SSCG 2016 and the SSCG 2016 recommended noradrenaline as a first-line vasopressor in the initial resuscitation of patients with sepsis. However, phenylephrine was also described as a first-line vasopressor in the SSCG 2016. Vasopressor selection is important in the initial resuscitation of patients with sepsis; thus, the decision to administer either noradrenaline or phenylephrine as a first-line vasopressor was taken up as a CQ.

A literature search yielded 3 RCTs [359–361]. Of these, the RCT conducted by Keriwala et al. [361] was publicly available on ClinicalTrials.gov but had not yet been published (NCT02203630). All the RCTs compared noradrenaline and phenylephrine among patients with septic shock. As a result of meta-analyses, noradrenaline administration resulted in a short-term mortality of 27 fewer per 1000 compared to phenylephrine administration. The incidence of arrhythmia events increased by 98 more per 1000. Based on the above, the desired effects of noradrenaline were limited, and the balance of effects was adjudged such that neither noradrenaline nor phenylephrine was superior to the other.

Both drugs are commonly adopted and used in Japan; however, it is thought that some medical staff may have minimal experience using phenylephrine for initial resuscitation. Therefore, in facilities with minimal experience with phenylephrine use, health providers may be hesitant to use phenylephrine as a first-line vasopressor.

**CQ6-10-1: Should adrenaline be used as a second-line vasopressor in adult patients with sepsis?**

***Answer:*** We suggest against using adrenaline as a second-line vasopressor in patients with sepsis/septic shock (GRADE 2D: certainty of evidence = “very low”).

***Rationale***

A literature search yielded 2 RCTs that investigated the use of adrenaline among patients with septic shock whose hemodynamics did not improve regardless of initial resuscitation or vasopressor administration [362, 363]. Patients with septic shock who received vasopressors were included in both RCTs, with a control group that received dopamine. A meta-analysis was performed using these studies. The estimated value of the effects of 28-day mortality yielded an RD of 48 more per 1000 (95%CI: 40 fewer to 165 more) (2 RCTs, *n* = 390), and that of 90-day mortality yielded an RD of 20 more per 1000 (95%CI: 80 fewer to 141 more) (1 RCT, *n* = 330). That of arrhythmia yielded an RD of 22 more per 1000 (95%CI: 44 fewer to 125 more) (2 RCTs, *n* = 390), and that of limb ischemia yielded an RD of 12 fewer per 1000 (95%CI: 33 fewer to 77 more) (2 RCTs, *n* = 390). The net harm was 78 per 1000, and the harm outweighed the benefit. Thus, it was adjudged that the comparative control was likely superior.

It should be noted that this investigation verified the effects of adrenaline as a vasopressor, and did not investigate its effects as an inotropic agent (see CQ6–11 for investigations of its utility as an inotropic agent among patients with cardiac dysfunction).

**CQ6-10-2: Should vasopressin be used as a second-line vasopressor in adult patients with sepsis?**

***Answer:*** We suggest using vasopressin as a second-line vasopressor in patients with sepsis/septic shock (GRADE 2D: certainty of evidence = “very low”).

***Rationale***

A literature search yielded 4 RCTs which investigated adrenaline among patients with septic shock whose hemodynamics did not improve regardless of initial resuscitation or vasopressor administration [364–367]. A meta-analysis was performed using these studies. All RCTs compared noradrenaline and vasopressin among patients with sepsis who required vasopressors, and open-label vasopressors were used when the target blood pressure could not be maintained. The VANISH trial conducted by Gordon et al. [367] compared vasopressin and noradrenaline in addition to low-dose corticosteroids and a placebo.

The estimated value of the effects of 28-day mortality yielded a RD of 10 fewer per 1000 (95%CI: 56 fewer to 45 more) (4 RCTs, *n* = 1260) and that of 90-day mortality yielded an RD of 54 fewer per 1000 (95%CI: 114 fewer to 20 more) (1 RCT, *n* = 792). The estimated value of the effects of arrhythmia was 5 fewer per 1000 (95%CI: 16 fewer to 19 more) (3 RCTs, *n* = 1217), that of myocardial ischemia was 10 more per 1000 (95%CI: 7 fewer to 61 more) (2 RCTs, *n* = 1187), and that of limb ischemia had 22 more per 1000 (95%CI: 4 more to 69 more) (3 RCTs, *n* = 1217). The net effect was 37 per 1000, with the intervention being superior. Based on the above, the balance of benefits and harms was adjudged such that the “intervention was likely superior”.

**CQ6-11: Should inotropes be used in adult patients with sepsis accompanied by cardiogenic shock?**

***Answer:*** We suggest administering inotropes (adrenaline, dobutamine) in adult patients with septic shock accompanied by cardiac dysfunction (expert consensus: insufficient evidence).

***Rationale***

Cardiac dysfunction, referred to as sepsis-induced myocardial dysfunction (SIMD), is a complication seen in approximately 40% of patients with septic shock, and it has been suggested that it is associated with exacerbation [368, 369]. The inotropic drugs dobutamine and adrenaline have been administered in addition to the vasopressor noradrenaline for the management of septic shock with complications of SIMD; however, its effects are still under investigation. Whether inotropic drugs can be used for the management of cardiac dysfunction in septic shock is an important question in initial resuscitation, and this was taken up as a CQ.

The results of a systematic review yielded no RCTs that conformed to the PICO criteria. RCTs on septic shock in which cardiac function is normal or decreased included a report that comparatively investigated patients who received adrenaline as a control group and dobutamine + noradrenaline as an intervention group, and a report that comparatively investigated patients who received adrenaline + noradrenaline as a control group and dobutamine + noradrenaline as an intervention group [362, 363]. Both reports showed no differences in mortality rates or complications.

Considering that the mortality rate of patients with septic shock with decreased cardiac function is extremely high, it is thought that the administration of inotropic drugs such as dobutamine or adrenaline is beneficial when compared to cases in which they are not administered. However, some patients with septic shock accompanied by decreased cardiac function may experience the onset of serious arrhythmias due to the administration of inotropic drugs; therefore, it is necessary to carefully administer these drugs or promptly discontinue them in these cases.

**CQ6-12: Should β-blockers be used in adult patients with sepsis?**

***Answer:*** We suggest administering short-acting β1-adrenoceptor antagonists in patients with sepsis/septic shock while being monitored with the objectives of managing tachycardia which cannot be controlled with standard therapy like initial fluid resuscitation (GRADE 2D: certainty of evidence = “very low”). Administering short-acting β1-adrenoceptor antagonists can induce hemodynamic fluctuations, so they should be administered under the supervision of a physician with expertise in cardiovascular management in the ICU (expert consensus: insufficient evidence).

***Rationale***

Conventional treatment strategies for septic shock include initial fluid infusion and administration of vasopressor and cardiotonic drugs. Several recent studies have reported the effects of administering β_1_-adrenergic receptor blockers on tachycardia among patients with septic shock with the intent of controlling the heart rate. These studies reported improvements in hemodynamics, reduced fluid requirements, and a decreased short-term mortality rate associated with initial resuscitation with β_1_-adrenergic receptor blockers. This was an opportunity to review conventional treatment strategies and can be considered a standard treatment in the future, and so was taken up as a CQ.

The results of a systematic review yielded 2 RCTs that conformed to the PICO criteria [370, 371]. The research conducted by Morelli et al. [370] was a non-blinded single-center RCT that assessed esmolol among patients with a heart rate of more than 95/min Meanwhile, the study conducted by Wang et al. [371] was a blinded single-center RCT that compared a control group, an additional group that received milrinone, and another group that concomitantly received milrinone + esmolol among patients with a heart rate > 95/min despite sufficient fluid replacement. The estimated value of the effects of short-term mortality outcome (2 RCTs, *n* = 244) was 304 fewer per 1000 (95%CI: 395 fewer to 195 fewer), the length of stay in the ICU among survivors (1 RCT, *n* = 42) yielded a MD of 4 days shorter (95%CI: 18.06 shorter to 10.06 longer), and the number of ICU free days (1 RCT, *N* = 50) yielded an MD of 4.1 days longer (95%CI: 1.8 longer to 6.4 longer). Meanwhile, bradycardia was observed among 2 out of 30 patients in the intervention group in 1 RCT (*n* = 60). The estimated value of the effects of renal replacement therapy (1 RCT, *n* = 154) was 12 fewer per 1000 (95%CI: 141 fewer to 175 more). Based on the above, it was adjudged that the desired effects were larger and the undesired effects were trivial, with the intervention being superior.

β_1_-adrenergic receptor blocker administration may cause fluctuations in hemodynamics; thus, we decided to add the following comment: “it is desirable that this be administered in an ICU under the care of a physician who is experienced in circulatory management” after sufficiently administering standard treatment while being monitored.

**CQ6-13: What are the indications of assisted circulation in adult patients with septic shock?**

***Answer:*** There is insufficient evidence for the effects of assisted circulation such as veno-arterial extracorporeal membrane oxygenation (V-A ECMO) and intra-aortic balloon pump (IABP) for cardiac dysfunction in septic shock, and its applications are still under investigation (Provision of information for background question).

***Rationale***

Septic shock presents with not only shock due to relative decreases in intravascular volume associated with vasodilation but also cardiogenic shock due to cardiac dysfunction referred to as either SIMD or septic cardiomyopathy [372, 373]. An intra-aortic balloon pumping (IABP) randomized trial (IABP-SHOCK II trial) on cardiogenic shock cases [374, 375] showed no improved prognosis in cardiogenic shock from the use of IABP. A meta-analysis that compared veno-arterial (V-A) ECMO and IABP for cardiogenic shock [376] showed that V-A ECMO was safe to use and improved hemodynamics but yielded no significant differences in 30-day survival rate and had higher bleeding-related complications. Meanwhile, the Japanese guideline on the diagnosis and treatment of acute and chronic heart failure (2017 revised version) [377] stated that “routine use of IABP is not recommended, but its use is considered in severe cases of general heart failure that is not responsive to medical treatment”. Very few reports have investigated the use of IABP in patients with septic shock presenting with SIMD. Hiromi et al. [378] reported that the introduction of IABP saved the lives of two patients with sepsis; however, a study of ten patients conducted by Takahashi et al. [379] reported that the 28-day survival rate for the introduction of IABP was 30%, although hemodynamics did improve. There are some case reports and observational studies of the use of V-A ECMO for patients with septic shock presenting with SIMD; however, the survival rate widely varied from 15 to 70%. Huang et al. [380] investigated 52 patients in whom V-A ECMO was introduced and reported that the survival rate was 15% (8 patients); 40% (21 patients) experienced cardiac arrest prior to the introduction of V-A ECMO, and there is the possibility that introduction timing has a large influence on prognosis. A study conducted by Cheng et al. [381] on 151 adult patients with sepsis in whom V-A ECMO was introduced had reported a survival and discharge rate of 29.8%; however, an analysis that excluded those over the age of 75 years, patients with advanced malignant tumors, patients with end-stage heart/renal failure, and immunosuppressed patients (67 patients in total) reported a survival and discharge rate of 42%, suggesting that age and pathological conditions such as immunodeficiency may largely influence the prognosis. Meanwhile, Bréchot et al. [382] introduced V-A ECMO in 14 patients with septic shock (average ejection rate of 16%, average cardiac index of 1.3 L/min/m^2^) and reported a survival and discharge rate of 71.4%, with follow-up observations conducted over a year later reporting favorable quality of life. It should be noted that their study included a relatively large number of young patients (average age of 45 years), but this result shows the effectiveness of V-A ECMO. Out of 37 patients in whom V-A ECMO was introduced (average age of 54.7 years), Falk et al. [383] investigated 20 patients with decreased left ventricular function (average ejection rate of 25%) and reported an in-hospital survival rate of 90% and long-term survival rate of 75%. Vogel et al. [384] introduced veno-arteriovenous (VAV) ECMO in patients with septic cardiomyopathy (12 patients) and reported a six-month survival rate of 75%. The report by Vogel et al. in particular included five patients who experienced cardiac arrest prior to the introduction of ECMO (41.7%), and these results are thought to sufficiently show the effectiveness of ECMO usage. Takauji et al. [385] examined the prognoses of 30 patients in whom V-A ECMO was introduced from a sub-analysis of the Japan Septic Disseminated Intravascular Coagulation (JSEPTIC DIC) study conducted in Japan from 2011 to 2013 to and showed that the survival and discharge rate was 20%. These results were somewhat lower than those of global reports. However, the survival rate of patients who received V-V ECMO for ARDS in Japan has improved by over a factor of two from 36% (2009) to 79% (2016) [386], and future improvements in performance are expected even with the use of V-A ECMO in adult patients with sepsis presenting with severe cardiac dysfunction. Previous studies to date have found age [380], severe cardiomyopathy [387], cardiac arrest prior to ECMO introduction [387], and time from shock to introduction of ECMO [388] to be prognostic factors among adult patients with septic shock in which V-A ECMO was introduced. However, other factors such as improvements in ECMO devices and proficiency level of medical staff with regard to ECMO devices are also important, and it is thought that treatment strategies that consider these aspects are also needed. The number of reports of V-A ECMO for adult patients with sepsis remains insufficient, and many of these are single-center retrospective observational studies. There have not been any RCTs investigating the treatment effectiveness, and the efficacy of V-A ECMO and IABP in adult patients with sepsis presenting with severe cardiac dysfunction is currently under investigation.

**CQ7: Corticosteroid therapy**

**Introduction**

Corticosteroids exert anti-stress effects at physiological concentrations and display potent anti-inflammatory effects at pharmacological concentrations [389, 390]. In critically ill patients, such as those with sepsis, dysfunction of the hypothalamic-pituitary-adrenal axis is frequently observed, and was termed “critical illness-related corticosteroid insufficiency (CIRCI).” The guidelines for CIRCI were initially developed in 2008 and updated in 2017 [391].

Steroid therapies for sepsis are categorized as high-dose therapy and low-dose therapy based on the daily doses administered. Regarding high-dose therapy, 2 RCTs failed to show the effectiveness of high-dose methylprednisolone in the 1980s [392, 393]. Meanwhile, since a small RCT conducted by Annane et al. reported on the effectiveness of low-dose hydrocortisone in patients with relative adrenal insufficiency in 2002, low-dose therapy has received increased attention [394]. The first edition of the SSCG published in 2004 recommended 7 days of therapy for patients who were unresponsive to initial fluid resuscitation and required vasopressors. However, a large-scale RCT (the CORTICUS study) conducted in 2008 merely showed earlier shock reversal without a difference in the mortality rate [395], and the later editions of the SSCG recommended short-term hydrocortisone use in patients with septic shock who were unresponsive to fluid resuscitation and vasopressors. After a 10-year period, the results of two large-scale RCTs were published in 2018. Among these 2 RCTs, one failed to show improvements in mortality (the ADRENAL trial), whereas the other that targeted patients with more severe conditions showed significant improvements in the mortality rate (the APROCCHSS trial) [396, 397]. Thus, steroid therapies have subsequently once again been in the limelight. In the current version of the guideline, we performed a systematic review for each CQ based on the GRADE criteria, and made recommendations.

**Fig. 6:**
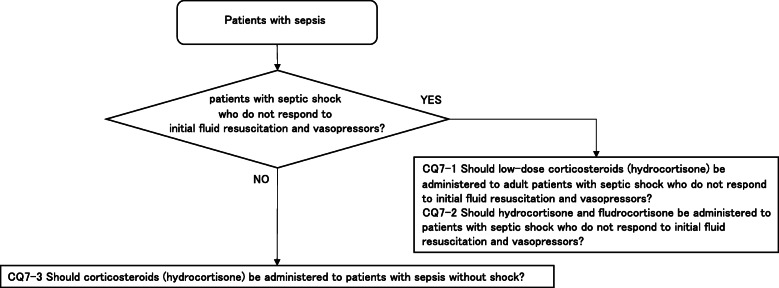
CQ7: Corticosteroid therapy (clinical flow)

Clinical flow of these CQs is shown in Fig. 6.

**CQ7-1: Should low-dose corticosteroids (hydrocortisone) be administered to adult patients with septic shock who do not respond to initial fluid resuscitation and vasopressors?**

***Answer:*** We suggest administering low-dose corticosteroids (hydrocortisone) to adult patients with septic shock who do not respond to initial fluid resuscitation and vasopressors for the purpose of withdrawing from shock (GRADE 2D: certainty of evidence = “very low”).

***Rationale***

The estimate of effect for middle term mortality(9 RCTs, *n* = 6424) was 21 fewer per 1000 (95%CI: 40 fewer to 3 more) [367, 394–401], and that for long term mortality (5 RCTs, *n* = 5716) was 23 fewer per 1000 (95%CI: 45 fewer to 4 more), indicating that the effects were limited [394–397, 402]. Meanwhile, that for the shock withdrawal period (5 RCTs, *n* = 4661) yielded a MD of 31.53 h shorter (95%CI 36.6 shorter to 26.46 shorter) [367, 395, 396, 403]. Based on these results, desirable effects were judged as small. Meanwhile, the estimate of effect of all serious adverse effects (3 RCTs, *n* = 5313) was 10 fewer per 1000 (95%CI: 23 fewer to 4 more) [367, 396, 397], that of superinfection (7 RCTs, *n* = 5825) was 8 more per 1000 (95%CI: 12 fewer to 31 more) [394–399, 402], and that of gastrointestinal bleeding (6 RCTs, *n* = 2161) was 6 more per 1000 (95%CI: 13 fewer to 32 more) [394, 395, 397–399, 402]. Thus, the undesirable effects were judged as trivial. In summary, the desirable effects for outcomes other than the “shock withdrawal period” were limited, whereas no differences in outcomes for serious adverse effects were seen regarding undesirable effects. From the perspective of an individual patient or his/her family, the balance of effects was judged as probably favoring the intervention. However, it is desirable that this intervention should not be performed as standard therapy for all patients with sepsis or septic shock. Furthermore, the RCTs selected in this analysis were all based on low-dose steroids, and this recommendation assumes that low-dose steroids are being used.

**CQ7-2: Should hydrocortisone and fludrocortisone be administered to patients with septic shock who do not respond to initial fluid resuscitation and vasopressors?**

***Answer:*** We suggest concomitant administration of hydrocortisone and fludrocortisone to adult patients with septic shock who do not respond to initial fluid resuscitation and vasopressors (GRADE 2C: certainty of evidence = “low”).

***Rationale***

The estimate of the effects for 28-day mortality (2 RCTs, *n* = 1540) was 52 fewer per 1000 (95%CI: 4 fewer to 95 fewer) [394, 397]. That for long-term mortality obtained from 3 RCTs with a low RoB (3 RCTs, *n* = 2049) was 53 fewer per 1000 (95%CI: 11 fewer to 90 fewer) [394, 397, 404] and that for shock withdrawal (1 RCT, *n* = 299) was 124 more per 1000 (95%CI: 9 more to 271 more) [394]. It was adjudged from these results that the co-administration of hydrocortisone and fludrocortisone yielded large desirable effects. Meanwhile, the effects for all serious adverse effects were as follows: superinfections (3 RCTs, *n* = 2048) yielded an effect of 33 more per 1000 (95%CI: 35 fewer to 119 more) [394, 397, 404], gastrointestinal bleeding (2 RCTs, *n* = 1539) yielded an effect of 3 fewer per 1000 (95%CI: 23 fewer to 27 more) [394, 397], and mental illness (3 RCTs, *n* = 299) yielded an effect of 4 fewer per 1000 (95%CI: 6 fewer to 47 more) [394, 397, 404]. This shows that the undesirable effects of co-administration of hydrocortisone and fludrocortisone were trivial. In summary, the desirable effects of co-administration of hydrocortisone and fludrocortisone were large, whereas the undesirable effects were trivial. Therefore, the balance of effects was judged as probably favoring the intervention [405]. The same decision would be made even when assuming worst-case scenarios (lower limit of CI for desirable effects, upper limit of CI for undesirable effects). It is desirable that this intervention be administered only among patients with septic shock that is refractory to initial fluid resuscitation and vasopressors. It should be also noted that the national health insurance coverage of fludrocortisone is limited to salt-wasting congenital adrenal hyperplasia and Addison’s disease.

**CQ7-3: Should corticosteroids (hydrocortisone) be administered to patients with sepsis without shock?**

***Answer:*** We suggest against administering hydrocortisone to patients with sepsis without shock (GRADE 2D: certainty of evidence = “very low”).

***Rationale***

The estimate of effects for 28-day mortality (3 RCTs, *n* = 437) was 2 fewer per 1000 (95%CI: 48 fewer to 74 more) [406–408]. That for progression to shock (1 RCT, *n* = 349) was 27 fewer per 1000 (95%CI: 94 fewer to 71 more) [406]. It was adjudged from these results that the desirable effects of hydrocortisone administration were trivial. Meanwhile, that for long-term mortality (2 RCTs, *n* = 382) was 26 more per 1000 (95%CI: 42 fewer to 131 more) [406, 408]. The estimate of effects for all serious adverse effects were as follows: superinfection (1 RCT, *n* = 375) yielded 46 more per 1000 (95%CI: 27 fewer to 157 more) [406] and gastrointestinal bleeding (1 RCT, *n* = 375) yielded 6 more per 1000 (95%CI: 8 fewer to 85 more) [406]. From these results, the undesirable effects of hydrocortisone administration were judged as trivial. In summary, the desirable and undesirable effects were both trivial. Therefore, the balance of effects did not support either the intervention or comparison regardless of the relative value circumstances of the patient or his/her family. This recommendation also does not apply to continuation of corticosteroid administration for patients who have been treated with corticosteroids for chronic diseases.

**CQ8: Blood transfusion therapy**

**Introduction**

Sepsis is often associated with a pathology that requires blood transfusion therapies, such as anemia or coagulopathy. However, there is limited evidence regarding blood transfusion therapy among sepsis patients, and there is still much debate regarding its indications.

Insured medical care in Japan is required to comply with the “Guidelines for the use of blood transfusion therapy, 2019 revised edition” published by the Ministry of Health, Labour and Welfare [409]. Among these, the J-SSCG 2016 is cited for blood transfusion, which states that a trigger value of Hb level 7 g/dL is recommended for anemia in sepsis patients. However, there are no items for sepsis patients regarding fresh frozen plasma and platelet concentrate [3, 4].

It is thought that there is some degree of consensus in starting blood transfusion below a hemoglobin level of 7 g/dL in relatively young intensive care patients who have no underlying cardiovascular diseases. However, there are many seniors or patients with underlying cardiovascular diseases in actual clinical practice, and it is thought that blood transfusions should be administered considering these patient backgrounds and the presence of shock. Therefore, we devised CQs on blood transfusion in cases of initial resuscitation of septic shock (CQ8–1) and cases where hemodynamics are stable (CQ8–2), where we investigated the starting criteria for appropriate blood transfusion according to sepsis pathology.

It is thought that there is some degree of consensus in not administering fresh frozen plasma or platelet concentrate transfusion to patients with sepsis without hemorrhaging tendencies and surgical procedures are not required. However, neither the J-SSCG 2016 nor the SSCG 2016 have provided recommendations based on sufficient evidence regarding fresh frozen plasma and platelet concentrate transfusion in patients with sepsis [1–4]. Coagulopathy due to systemic inflammatory response is more likely to occur in sepsis patients, and the prognosis when this is accompanied by DIC is poor. Thus, it is thought that appropriate coagulation factors and platelets should be supplemented according to coagulopathy pathology. Therefore, we devised CQs on fresh frozen plasma (CQ8–3) and platelet transfusion (CQ8–4) investigating the administration criteria of fresh frozen plasma, platelet transfusion, and administration concepts.

**Fig. 7:**
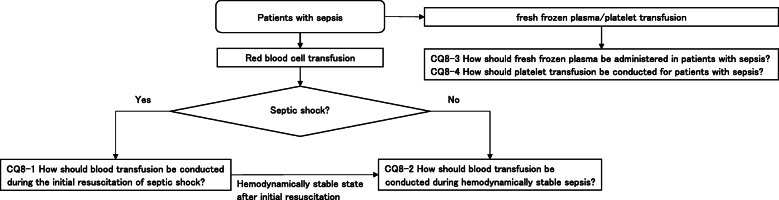
CQ8: Blood transfusion therapy (clinical flow)

Clinical flow of these CQs is shown in Fig. 7.

**CQ8-1: How should blood transfusion be conducted during the initial resuscitation of septic shock?**

***Answer:*** We suggest starting blood transfusion at a hemoglobin level of less than 7 g/dL during initial resuscitation for patients with septic shock (GRADE 2C: certainty of evidence = “low”).

***Rationale***

The J-SSCG 2016 recommends blood transfusion at a hemoglobin level below 7 g/dL for the initial resuscitation of septic shock [3, 4]. Furthermore, neither the 2019 edition of the “Guidelines for the use of blood transfusion therapy” of the Ministry of Health, Labour and Welfare nor the SSCG 2016 discussed pathological conditions such as the shock period or after shock withdrawal, but a reference recommended starting blood transfusion at a hemoglobin level of < 7 g/dL under conditions presumed to be related to shock [409]. Meanwhile, the risks of ischemic organ injury due to tissue hypoxemia, which is thought to occur when hemoglobin levels are insufficient, also need to be considered.

The results of a systematic review yielded only one relevant RCT [410]. The RCT reported that starting blood transfusion at a hemoglobin level less than 7 g/dL resulted in a 90-day mortality rate of 18 fewer per 1000 (95%CI: 76 fewer to 45 more) when compared to initiating transfusion at less than 10 g/dL. The number of ischemic events was 8 fewer per 1000 (95%CI: 33 fewer to 31 more).

Thought processes regarding blood transfusion vary on an individual basis, and there are patients or families who refuse blood transfusions due to reasons such as religion, but administering as little blood transfusion as possible and avoiding transfusion complications is generally thought to be prioritized by patients and family. After considering medical costs and burdens on medical sites, it is suggested that blood transfusions begin at hemoglobin levels less than 7 g/dL for the initial resuscitation of patients with septic shock.

It is desirable to evaluate the presence of ischemic complications during implementation. This recommendation does not apply to patients who are compensatory for hyperhemoglobinemia due to the presence of chronic hypoxemia (e.g., due to the presence of right-to-left shunts), and individual responses are required in such cases.

**CQ8-2: How should blood transfusion be conducted during hemodynamically stable sepsis?**

***Answer:*** We suggest starting blood transfusion at a hemoglobin level of less than 7 g/dL in patients with hemodynamically stable sepsis (expert consensus: insufficient evidence).

***Rationale***

Tissue hypoxia accompanying anemia is a clinically important issue. Blood transfusion is a response to this and is conducted for preventative purposes, but transfusion over the amount needed increase the risk of allergies and infection associated with the administration of blood transfusion therapy. There are also risks such as circulatory loads associated with the administration of blood transfusion therapy as well as the onset of transfusion-related acute lung injury (TRALI; frequency of lethal TRALI due to blood transfusion: 1: 2–3,000,000 products) [411]. Therefore, it is thought that administering the minimum amount of transfusions to avoid disorders associated with anemia is important.

It was adjudged in the J-SSCG 2016 that a certain degree of consensus has been reached regarding the starting criteria of blood transfusions for sepsis patients with stable hemodynamics, and this was not taken up as a CQ [3, 4]. However, clarifying the starting criteria for blood transfusion in patients with stable hemodynamics was also thought to be an important clinical issue, and this was taken up in this sepsis clinical practice guideline. The results of a systematic review yielded no relevant RCTs. Administering a minimal amount of transfusions that prevent disorders associated with anemia was thought to result in the effects of transfusion while minimizing complications as well as having a high potential benefit for patients. Meanwhile, limiting the start of blood transfusions to hemoglobin levels of 7.0 g/dL may further increase the burden on the heart and be harmful to some patients with ischemic heart disease or heart failure. From the above, although the balance of effects is thought to vary according to patient conditions, we suggest that blood transfusion should be administered at hemoglobin levels less than 7 g/dL even in sepsis patients with stable hemodynamics if severe heart failure or ischemic heart disease is not present.

It is desirable to evaluate the presence of ischemic complications during implementation. This recommendation is not applicable to patients who are compensatory for hyperhemoglobinemia due to the presence of chronic hypoxemia (e.g., due to the presence of right-to-left shunts), and individual responses are required in such cases.

**CQ8-3: How should fresh frozen plasma be administered in patients with sepsis?**

***Answer:*** We suggest administering fresh frozen plasma in patients with sepsis when hemorrhaging tendencies are observed. If surgical/invasive interventions are required, we suggest administering when PT/APTT is extended (PT is over INR 2.0 or activity level of less than 30%; APTT is over two times the upper limit of standards at each medical institution or activity level less than 25%) or when fibrinogen levels are less than 150 mg/dL (expert consensus: insufficient evidence).

***Rationale***

It has been reported that coagulopathy is associated with sepsis patients at a high rate, and the prognosis of sepsis patients with complications of coagulopathy is poor [412]. Fresh frozen plasma is sometimes administered to patients with sepsis when hemorrhaging tendencies are present or surgical procedures are required to improve coagulopathy. However, the usefulness of fresh frozen plasma, including during surgical treatment, is unclear [413, 414]. There is no set evaluation of the effectiveness and harmfulness of fresh frozen plasma in sepsis patients with the objective of improving coagulopathy, and there are various administration criteria for it even in clinical settings. From the above, it was thought that this was an important clinical issue to be addressed in sepsis treatment guidelines, and this was taken up as a CQ.

The results of a systematic review yielded no relevant RCTs. It is thought that there is a potentially high benefit to patients when administering fresh frozen plasma in order to address and prevent hemorrhaging states accompanying coagulopathy, or hemorrhaging associated with invasive interventions when coagulopathy is present. No harmful effects have been proven due to the administration of fresh frozen plasma when no hemorrhaging tendencies are seen, and no surgical procedures are required. However, there is an increased risk of allergies and infections associated with the administration of blood transfusion therapy. There is also the risk of circulatory loads associated with the administration of blood transfusion preparations as well as the onset of TRALI (frequency of lethal TRALI due to fresh frozen plasma; 1:2–300,000 products) [411]. At the very least, it is thought that the benefits of fresh frozen plasma administration outweigh the harms in cases of associated hemorrhaging symptoms due to severe coagulopathy or when hemorrhaging due to invasive interventions is predicted.

**CQ8-4: How should platelet transfusion be conducted for patients with sepsis?**

***Answer:*** We suggest conducting platelet transfusion in patients with sepsis and platelet counts of less than 10,000/μL, or less than 50,000/μL when accompanied by hemorrhaging symptoms (expert consensus: insufficient evidence). We suggest conducting platelet transfusion so as to maintain a platelet count of over 50,000/μL when active hemorrhaging is observed or when surgical/invasive procedures are needed (expert consensus: insufficient evidence).

***Rationale***

Complications of thrombocytopenia occur at a high rate among sepsis patients, and it is one of the organ disorders included in the sequential organ failure assessment score. It has been reported that sepsis patients with thrombocytopenia have a high rate of shock, acute renal injury, and hemorrhagic adverse event complications, and show poor prognoses [415, 416]. A prospective cohort study on sepsis patients in Japan also showed thrombocytopenia (< 100,000/μL) in 345/1184 patients (29.1%) [417]. Meanwhile, there is a risk of harm such as TRALI when administering platelets (frequency of lethal TRALI due to platelet administration; 1:3–400,000 products) [411]. In Japan, platelets are often administered to patients with sepsis who have hemorrhaging tendencies or who have associated thrombocytopenia and require surgical treatment. However, its usefulness is not clear. Based on the above, platelet transfusions for sepsis patients were thought to be an important clinical issue to be addressed in the sepsis clinical practice guidelines, and this was taken up as a CQ.

The results of a systematic review yielded no relevant RCTs. It is thought that the potential benefits to patients is high when administering platelet transfusions in addressing and preventing hemorrhagic symptoms associated with thrombocytopenia or the hemorrhaging which accompanies invasive interventions during thrombocytopenia. The harmful effects have not been proven for platelet transfusion when there are no hemorrhaging tendencies and surgical procedures are not required; however, there are increased risks of allergies and infection associated with blood transfusion therapy. Unlike other blood transfusion therapy, platelet preparations are stored at room temperature (20–24 °C), and care must be taken to treat infectious diseases caused by bacterial contamination. There is also the risk of circulatory loads associated with the administration of blood transfusion preparations as well as the onset of TRALI [411]. At the very least, it is thought that the benefits of platelet transfusion outweigh its harm in cases of hemorrhagic symptoms due to severe thrombocytopenia or when hemorrhaging due to invasive interventions is expected.

**CQ9: Respiratory management**

**Introduction**

Respiratory management in the treatment of sepsis involves many therapies, from oxygen therapy to mechanical ventilation and/or extracorporeal membranous oxygenation. Sufficient oxygen supply to the entire body is essential in cases in which balances between oxygen supply and demand are likely to be lost, including worsening hemodynamics. On the other hand, harmful effects of excessive oxygen administration have been indicated according to pathological condition [418]. Therefore, it was adjudged that indicating the target S_P_O_2_ range as a guide (CQ9–1) could be important from a clinical perspective. Non-invasive ventilation (NIV) [419] and nasal high-flow therapy (NHFT) [420] have been determined to be treatment options for pre-intubation respiratory management if normal oxygen therapy was insufficient (CQ9–2). Increased levels of attention have been paid to the indication of protective ventilation strategies (CQ9–3) and the selection of positive end-expiratory pressure (PEEP) settings (CQ9–4) when respiratory conditions have worsened and the patient is shifted to mechanical ventilation with tracheal intubation. Meanwhile, it is theoretically desirable to administer lung protective respiratory management as it minimizes ventilator-induced lung injury (VILI) caused by positive pressure ventilation and patient self-inflicted lung injury (P-SILI) caused by strong spontaneous respiration in the patient [421]. Consideration of hemodynamics according to disease stage and pathology, such as septic shock [422], circulatory stable period, acute respiratory distress syndrome (ARDS) [423], and the convalescent period, is needed during mechanical ventilation for patients with sepsis. Successful treatment of sepsis normally results in simultaneous improvements in respiratory condition; thus, weaning from mechanical ventilation can be considered. In addition to the evaluation of airway patency [424] and airway clearance ability [425], the spontaneous breathing trial (SBT) [426] is a typical method used to judge whether mechanical support with ventilator can be withdrawn. Whether to set a protocol for the weaning process, including SBT (CQ9–5) and whether to administer preventative NIV [427] or NHFT [428] as modes of respiratory management after tracheal extubation (CQ9–6) are thought to be important clinical issues for reducing post-extubation respiratory failure or re-intubation and succeeding in weaning patients from mechanical ventilation.

**Fig. 8:**
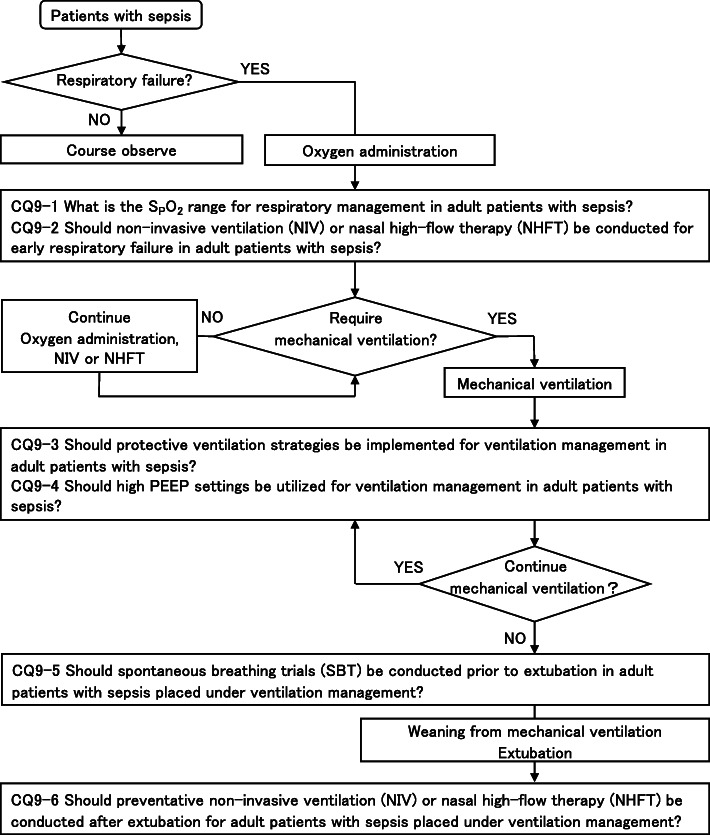
CQ9: Respiratory management (clinical flow)

Clinical flow of these CQs is shown in Fig. 8.

**CQ9-1: What is the S**_**P**_ **O**_**2**_ **range for respiratory management in adult patients with sepsis?**

***Answer:*** We suggest against setting a high target S_P_O_2_ (98–100%) during respiratory management in adult patients with sepsis (GRADE 2B: certainty of evidence = “moderate”).

***Remarks:*** This does not apply in cases where there is the possibility of a disruption in the oxygen supply/demand balance due to severe anemia or increased metabolism due to infection in cases where hemodynamics are unstable.

***Rationale***

A systematic review was performed on RCTs which compared high target S_P_O_2_ groups with low target S_P_O_2_ groups among critically ill patients requiring oxygen administration. The results of meta-analyses showed that the estimate of effects for short-term mortality (3 RCTs, *n* = 673) by setting a high target S_P_O_2_ yielded an RD of 42 more per 1000 (95%CI: 38 fewer to 156 more) [429–431] organ damage (1 RCT, *n* = 434) yielded an RD of 66 more per 1000 (95%CI: 11 fewer to 175 more) [429], and new infection (1 RCT, *n* = 434) yielded an RD of 49 more per 1000 (95%CI: 22 fewer to 153 more) [429]. Therefore, the possibility of an increased short-term mortality rate or an increased frequency of associations with additional infection or systemic organ failure may be more strongly suggested if further investigations reveal similar results as these trials. All outcomes investigated did not support respiratory management with a high target S_P_O_2_, and no outcomes were investigated for desirable effects; thus, it was adjudged that respiratory management with a low target S_P_O_2_ was likely superior. Due to the small sample size and number of trials, we conditionally suggest this after comprehensively evaluating these findings.

A specific S_P_O_2_ value of 98–100% was recorded in the recommendation. However, we found no reports that investigated to what extent S_P_O_2_ negatively impacts outcomes, and further investigations on the optimal target S_P_O_2_ range are thought to be needed in the future. Pathological conditions such as increased oxygen demand and decreased oxygen supply should also be sufficiently considered in the treatment of sepsis, and emergency measures such as increasing oxygen administration or oxygen concentration until hemodynamics recover should be used. This recommendation does not deny these actions.

The results of a meta-analysis of a total of five reports including 2 RCTs published after the period of systematic review (both published in the NEJM in 2020) [432, 433] were added as a supplement. The estimate of effects for short-term mortality (5 RCTs, *n* = 1833) yielded an RD of 12 fewer per 1000 (95%CI: 81 fewer to 81 more) [429–433], organ damage (3 RCTs, *n* = 1600) yielded an RD of 12 more per 1000 (95%CI: 51 fewer to 102 more) [429, 432, 433] and new infection (2 RCTs, *n* = 635) yielded an RD of 48 more per 1000 (95%CI: 12 fewer to 129 more) [429, 432]. Based on the above, it was adjudged that the recommendations for this CQ would not change significantly even if the benefits and harms were investigated after incorporating the latest research findings.

**CQ9-2: Should non-invasive ventilation (NIV) or nasal high-flow therapy (NHFT) be conducted for early respiratory failure in adult patients with sepsis?**

***Answer:*** We suggest conducting non-invasive ventilation (NIV) or nasal high-flow therapy (NHFT) for early respiratory failure in adult patients with sepsis (GRADE 2A: certainty of evidence = “high”).

***Rationale***

A systematic review was performed on RCTs which compared groups which underwent either NIV, NHFT, or conventional oxygen therapy (COT) during respiratory management for acute hypoxic respiratory failure. Network meta-analysis methods were used to conduct comparative investigations between the three groups. The estimate of network effects for short-term mortality were as follows: when compared to COT, NHFT yielded an RD of 65 fewer per 1000 (95%CI: 95 fewer to 28 more) (5 RCTs, *n* = 1453) [434–438]; NIV yielded an RD of 30 fewer per 1000 (95%CI: 60 fewer to 3 more) (14 RCTs, *n* = 2359) [434, 435, 439–450]. When compared to NHFT, NIV yielded an RD of 8 fewer per 1000 (95%CI: 35 fewer to 25 more) (3 RCTs, *n* = 338) [434, 435, 451]. The estimate of network effects for tracheal intubation were as follows: when compared to COT, NHFT yielded an RD of 65 fewer per 1000 (95%CI: 95 fewer to 28 fewer) (6 RCTs, *n* = 1563) [434–438, 442] and NIV yielded an RD of 60 fewer per 1000 (95%CI: 92 fewer to 29 fewer) (17 RCTs, *n* = 2506 [434, 435, 439–441, 443–449, 452–456]. When compared to NHFT, NIV yielded an RD of 5 more per 1000 (95%CI: 32 fewer to 46 more) (5 RCTs, *n* = 1584) [434, 435, 451, 457, 458]. The estimate of network effects for time until tracheal intubation were as follows: compared to COT, NHFT yielded an MD of 1.15 h longer (95%CI: 0.21 shorter to 2.09 longer) (1 RCT, *n* = 200) [434], NIV yielded an MD of 0.53 h longer (95%CI: 0.27 shorter to 0.80 longer) (2 RCTs, *n* = 284) [434, 449] When compared to NHFT, NIV yielded an RD of 0.62 h shorter (95%CI: 1.52 shorter to 0.28 longer) (2 RCTs, *n* = 432) [434, 458]. Furthermore, the surface under the cumulative ranking curves (SUCRAs) for short-term mortality were 77.3, 64.4, and 8.3 for NIV, NHFT, and COT, respectively. Those for tracheal intubation were 74.5, 74.7, and 0.8 for NIV, NHFT, and COT, respectively. Those for time until tracheal intubation were 40.3, 85.2, and 24.5 for NIV, NHFT, and COT, respectively. No differences in effects were observed for short-term mortality and time until tracheal intubation; however, the possibility of avoiding tracheal intubation with either NIV or NHFT was suggested. All outcomes raised as undesirable effects had low importance and were thus not included in investigations, and the evidence level of desirable effects was “large”; thus, it was adjudged that the balance of effects was such that intervention was likely superior. Therefore, we decided to recommend both NIV and NHFT weakly, and we conditionally suggest these after comprehensive judgment.

**CQ9-3: Should protective ventilation strategies be implemented for ventilation management in adult patients with sepsis?**

***Answer:*** We suggest implementing protective ventilation strategies for ventilation management in adult patients with sepsis (GRADE 2B: certainty of evidence = “moderate”).

***Rationale***

A systematic review was performed on RCTs which compared groups that received protective ventilation which limited plateau pressure by either low tidal volume or low plateau pressure and groups that did not (conventional) among critically ill patients who required mechanical ventilation management. We decided not to investigate PEEP values for either group. The results of a meta-analysis showed that the estimate of effects for short-term mortality (9 RCTs, *n* = 2422) were as follows: when compared to the conventional group, protective ventilation yielded an RD of 36 fewer per 1000 (95%CI: 88 fewer to 24 more) [459–467], ventilator-free days (VFDs) (3 RCTs, *n* = 1911) yielded an MD of 1.79 days longer (95%CI: 0.62 shorter to 4.20 longer) [463, 464, 467] and barotrauma (7 RCTs, *n* = 2182) yielded an RD of 8 fewer per 1000 (95%CI: 31 fewer to 28 more) [459–464, 467]. Mechanical ventilation has the dual tendency to decrease the mortality rate and increase the number of VFDs. There were no major differences in the incidence of barotrauma as an adverse event. The investigated outcomes were generally in favor of intervention; thus, it was adjudged that protective ventilation was likely superior. We conditionally suggest this after comprehensive judgment including the balance of effects and evidence level.

**CQ9-4: Should high PEEP settings be utilized for ventilation management in adult patients with sepsis?**

***Answer:*** We suggest against utilizing high PEEP settings (PEEP over 12 cm H_2_O) for the initial stage of ventilation management in adult patients with sepsis (GRADE 2B: certainty of evidence = “very low”).

***Rationale***

We performed a systematic review of RCTs which compared high PEEP setting groups and low PEEP setting groups among critically ill patients who required mechanical ventilation management. The results of meta-analyses showed the following estimate of effects of high PEEP settings when compared to low PEEP settings: short-term mortality (7 RCTs, *n* = 3657) yielded an RD of 8 fewer per 1000 (95%CI: 54 fewer to 47 more) [461, 464, 468–472] and VFD (3 RCTs, *n* = 1654) yielded an MD of 0.45 days longer (95%CI: 2.02 shorter to 2.92 longer) [464, 468, 469]. The estimate of effects for undesirable effects was as follows: incidence of barotrauma (6 RCTs, *n* = 3457) yielded an RD of 5 more per 1000 (95%CI: 23 fewer to 53 more) [461, 464, 468, 469, 471, 472] and incidence of circulatory insufficiency (1 RCT, *n* = 1010) yielded an RD of 65 more per 1000 (95%CI: 6 more to 133 more) [469]. The effects of high PEEP on decreased short-term mortality and increased number of VFD were trivial and increases in barotrauma incidence as an undesirable effect were also trivial. Meanwhile, circulatory insufficiency tended to be promoted; however, it is unclear whether low PEEP settings can be adjudged as superior based on the results of this outcome, which was obtained from only one RCT. However, all subjects in this trial were diagnosed with moderate ARDS, and may have had risks associated with PEEP-induced circulatory insufficiency as backgrounds. Circulatory suppression is emphasized by high PEEP settings with septic shock; thus, further caution is required. After comprehensively evaluating these findings, we adjudged that low PEEP settings were likely superior, and conditionally suggest this.

A specific high PEEP value of over 12 cm H_2_O was set in the recommendation. However, there have been no reports which investigated to what extent high PEEP has a negative impact on outcomes, and further investigations are needed in the future. The effects of PEEP have also been reported to vary according to the severity of ARDS; thus, a higher PEEP setting may become necessary depending on the severity of the patient’s condition when he/she is diagnosed with ARDS.

**CQ9-5: Should spontaneous breathing trials (SBT) be conducted prior to extubation in adult patients with sepsis placed under ventilation management?**

***Answer:*** We suggest utilizing weaning protocols from ventilators, including spontaneous breathing trials (SBTs) prior to extubation in adult patients with sepsis placed under ventilation management (GRADE 2D: certainty of evidence = “very low”).

***Rationale***

We performed a systematic review of RCTs which compared groups that underwent protocol-based weaning including SBT prior to extubation with groups that did not undergo such a weaning process based on a protocol among critically ill patients who required mechanical ventilation. The results of meta-analyses showed that the estimate of effects for short-term mortality (8 RCTs, *n* = 1282) in the protocol-based group yielded an RD of 10 fewer per 1000 (95%CI: 52 fewer to 45 more) when compared to the no-protocol group [473–480]. There were no relevant references on VFD. The estimate of effects for re-intubation (7 RCTs, *n* = 1081) in the protocol-based group yielded an RD of 24 fewer per 1000 (95%CI: 61 fewer to 41 more) when compared to the no-protocol group [473, 475–477, 479, 481, 482]. Weaning protocols for mechanical ventilators, including SBT, tended to decrease short-term mortality and re-intubation rates; however, the evidence level for each outcome was extremely low. Meanwhile, there were no outcomes for undesirable effects. After comprehensively evaluating these findings, we adjudged that weaning protocols for mechanical ventilators, including SBT, were likely superior, and conditionally suggest this.

**CQ9–6: Should preventative non-invasive ventilation (NIV) or nasal high-flow therapy (NHFT) be conducted after extubation for adult patients with sepsis placed under ventilation management?**

***Answer:*** We suggest conducting preventative non-invasive ventilation (NIV) or nasal high-flow therapy (NHFT) over standard oxygen therapy following extubation for adult patients with sepsis placed under ventilation management (GRADE 2B: certainty of evidence = “moderate”).

***Rationale***

We performed a systematic review of RCTs which compared groups preventatively underwent NIV, NHFT, or COT immediately after extubation among patients who underwent mechanically ventilation for more than 12 h due to acute respiratory failure and who subsequently cleared the SBT. Network meta-analysis methods were used to comparatively investigate the three groups. The estimate of network effects for short-term mortality was as follows: compared to COT, NHFT yielded an RD of 12 fewer per 1000 (95%CI: 32 fewer to 16 more) (4 RCTs, *n* = 802) [483–486], NIV yielded an RD of 31 fewer per 1000 (95%CI: 53 fewer to 1 more) (5 RCTs, *n* = 784) [487–491]. When compared to NHFT, NIV yielded an RD of 43 fewer per 1000 (95%CI: 102 fewer to 32 more) (1 RCT, *n* = 604) [492]. The estimate of network effects for the rate of re-intubation were as follows: compared to COT, NHFT yielded an RD of 69 fewer per 1000 (95%CI: 99 fewer to 12 fewer) (5 RCTs, *n* = 864) [483–486, 493] and NIV yielded an RD of 66 fewer per 1000 (95%CI: 99 fewer to 1 fewer) (4 RCTs, *n* = 664) [487–489, 491]. When compared to NHFT, NIV yielded an RD of 16 more per 1000 (95%CI: 109 fewer to 271 more) (1 RCT, *n* = 604) [492]. The SUCRA values for short-term mortality were 91.8, 46.3, and 11.8 for NIV, NHFT, and COT, respectively. The SUCRA values for re-intubation were 69.8, 77.8, and 2.8 for NIV, NHFT, and COT, respectively. There was no difference in effects for the desirable effect of decreased short-term mortality; however, NIV and NHFT yielded decreased re-intubation rates compared to COT, and it was shown that both NIV and NHFT could potentially prevent re-intubation. All outcomes raised as undesirable effects were of low importance and thus not included in investigations, and the evidence level of desirable effects was “moderate”; therefore, based on the balance of effects, it was adjudged that intervention was likely superior. Consequently, we decided to recommend both NIV and NHFT weakly, and after comprehensive judgment, we conditionally suggested this.

**CQ10: Management of pain, agitation, and delirium**

**Introduction**

The 2013 Pain, Agitation, and Delirium (PAD) guidelines [494], its revised 2018 Pain, Agitation/sedation, Delirium, Immobility, and Sleep disruption (PADIS) guidelines [495], and the J-PAD guidelines put forth by the Japanese Society of Intensive Care Medicine [496] address the management of pain, agitation, and delirium in critically ill adult patients. However, most of the clinical research that serves as the basis for the decisions in these guidelines includes critically ill patients with various pathological conditions (including postoperative patients) as subjects, and very few studies have been conducted only on sepsis. However, there is no evidence that PAD management in sepsis differs from the management of other critically ill patients. Therefore, the “analgesia/sedation/delirium management” in the J-SSCG 2016 [3, 4] was created as an excerpt from the J-PAD guidelines [496]. This guideline set six CQs for the management of pain, agitation, and delirium in sepsis patients, and a systematic review and meta-analysis was performed according to the new GRADE system. A literature review assessed patients with severe illnesses other than sepsis in these CQs.

It has been suggested that pain management based on an analgesia-first sedation protocol using evaluation tools may improve ICU and clinical outcomes. CQs were established to balance the benefits and risks of pain management methods. The prevention of agitation is extremely important for shortening the duration of ventilator management and length of stay in the ICU. As these outcomes are directly linked to patient prognosis, CQs related to the differences in agitation management, such as the selection of sedatives and light sedation practices, were established. Delirium is a phenotype of central nervous system organ damage in septic patients. It is known that there is a correlation between the duration of delirium in the ICU and cognitive impairments occurring within 3 and 12 months of discharge from the ICU. CQs related to delirium prevention methods and treatment methods have been established.

The basic principle underlying the management of critically ill patients, including those with sepsis, has been summarized as “management with the minimum amount of sedatives needed based on sufficient pain control, frequent evaluations of delirium, and rehabilitation as rapidly as possible” [3, 4]. Please refer to the PADIS guidelines [495] and J-PAD guidelines [496], which are the clinical guidelines in this field if the content here is insufficient.

**Fig. 9:**
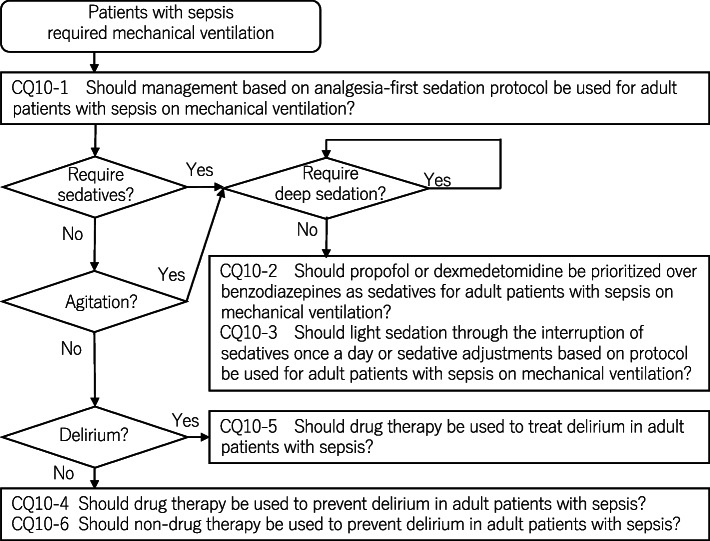
CQ10: Management of pain, agitation, and delirium (clinical flow)

Clinical flow of these CQs is shown in Fig. 9.

**CQ10-1: Should management based on analgesia-first sedation protocol be used for adult patients with sepsis on mechanical ventilation?**

***Answer:*** We suggest using management based on analgesia-first sedation protocol in adult patients with sepsis on mechanical ventilation (GRADE 2C: certainty of evidence = “low”).

***Rationale***

We performed a meta-analysis of 7 RCTs [497–503] that investigated the need to manage adult patients on mechanical ventilation with an analgesia-first sedation protocol.

The all-cause mortality due to management with an analgesia-first sedation protocol (5 RCTs, *n* = 1012) was 18 fewer 1000 (63 fewer to 35 more), the mechanical ventilation period (6 RCTs, *n* = 1090) yielded a MD that was 8.99 h shorter (20.66 shorter to 2.68 longer), the number of days in a 28-day period in which mechanical ventilation was not used (1 RCT, *n* = 113) yielded an MD that was 4.2 days longer (0.32 longer to 8.08 longer), and the length of stay in the ICU (6 RCTs, *n* = 1090) yielded an MD that was 15.15 h shorter (26.08 shorter to 4.22 shorter). Serious complications due to management with an analgesia-first sedation protocol (7 RCTs, *n* = 1296) occurred at a rate of 13 fewer per 1000 (36 fewer to 19 more), and the onset of delirium (1 RCT, *n* = 79) occurred at a rate of 55 fewer per 1000 (159 fewer to 194 more). Therefore, it was adjudged that the balance of effects was such that intervention was likely superior.

**CQ10-2: Should propofol or dexmedetomidine be prioritized over benzodiazepines as sedatives for adult patients with sepsis on mechanical ventilation?**

***Answer:*** We suggest using propofol or dexmedetomidine over benzodiazepines as sedatives for patients with sepsis on mechanical ventilation (GRADE 2D: certainty of evidence = “very low”).

***Rationale***

The selection of sedatives has been reported to influence the incidence of agitation. Preventing agitation can be directly linked to prognosis; thus, the choice of sedative during mechanical ventilation management is extremely important. Therefore, a systematic review of sedative interventions based on either propofol or dexmedetomidine with benzodiazepine sedatives as a control was performed. A meta-analysis was conducted after confirming 14 RCTs [504–518]. Compared to sedation with benzodiazepines, sedation with propofol or dexmedetomidine yielded a mortality rate (10 RCTs, *n* = 1573) of 4 more per 1000 (32 fewer to 50 more), and an incidence rate of agitation of 66 fewer per 1000 (119 fewer to 3 more). The MD for the duration of mechanical ventilation and length of stay in the ICU were each 1.56 days shorter (2.46 shorter to 0.67 shorter) and 2.06 days shorter (2.72 shorter to 1.39 shorter), respectively. Unplanned extubation yielded a corresponding rate of 31 more per 1000 (22 fewer to 128 more). Considering the intervention-based benefits of reduced duration of mechanical ventilation and length of stay in the ICU, it was adjudged that the intervention was likely superior.

**CQ10–3: Should light sedation through the interruption of sedatives once a day or sedative adjustments based on protocol be used for adult patients with sepsis on mechanical ventilation?**

***Answer:*** We suggest using light sedation through the interruption of sedatives once a day or sedative adjustments based on protocol for patients with sepsis on mechanical ventilation (GRADE 2C: certainty of evidence = “low”).

***Rationale***

The practice of light sedation is important not only for confirming the level of consciousness and detecting agitation at an early stage, but also for shortening the duration of mechanical ventilation and length of stay in the ICU. Therefore, we conducted a systematic review with the objective of comparing the practice of light sedation, which is performed by suspending sedatives once a day or a protocol-based adjustment of sedative use, to that of deep sedation. A meta-analysis was conducted on 2 RCTs [519, 520]. The practice of light sedation resulted in a mortality rate (2 RCTs, *n* = 257) that was 57 fewer per 1000 (135 fewer to 60 more). The duration of mechanical ventilation (2 RCTs, *n* = 257) yielded a MD of 2.49 days shorter (4.43 shorter to 0.54 shorter), and the length of stay in the ICU (2 RCTs, *n* = 257) had an MD of 3.34 days shorter (6.09 shorter to 0.60 shorter). Unplanned extubation (1 RCT, *n* = 128) yielded a corresponding rate of 37 fewer per 1000 (61 fewer to 88 more). From these results, it was adjudged that the intervention was likely superior.

**CQ10-4: Should drug therapy be used to prevent delirium in adult patients with sepsis?**

***Answer:*** We suggest administering dexmedetomidine for delirium prevention in adult patients with sepsis (GRADE 2C: certainty of evidence = “low”). We suggest against the administration of haloperidol (GRADE 2B: certainty of evidence = “moderate”). We suggest against the administration of atypical antipsychotics (GRADE 2C: certainty of evidence = “low”). We suggest against the administration of statins (GRADE 2D: certainty of evidence = “very low”).

***Remarks:*** We recommend against the routine administration of dexmedetomidine to patients who do not require sedation. Furthermore, dexmedetomidine administration can cause hemodynamic fluctuations, so this should ideally be administered under the supervision of a physician who is experienced with systematic management in an ICU (expert consensus).

***Rationale***

The results of a systematic review yielded the following RCTs that conformed to the PICO criteria: these included studies with dexmedetomidine, 8 [510, 521–527]; haloperidol, 7 [514, 521, 528–532]; atypical antipsychotics, 3 [514, 533, 534]; and statins, 2 [535, 536]. A meta-analysis was performed using these RCTs. Prophylactic administration of dexmedetomidine reduced the incidence of delirium (7 RCTs, *n* = 1658) by 155 per 1000 (95%CI: 203 fewer to 83 fewer), and it was adjudged that the desired effects were moderate. The effect of prophylactic administration of haloperidol on the incidence of delirium (5 RCTs, *n* = 2159) was 34 fewer per 1000 (95%CI: 92 fewer to 40 more). The expected effect of atypical antipsychotics in 2 RCTs (*n* = 227) with only post-operative patients as subjects yielded a decrease in 203 per 1000 people (95%CI: 225 fewer to 111 fewer). The expected value of the effects of statins in 1 RCT (*n* = 142) yielded 9 fewer per 1000 (95%CI: 94 fewer to 66 more).

Meanwhile, the incidence rate of serious adverse events due to the prophylactic administration of dexmedetomidine decreased by 53 per 1000 (95%CI: 69 fewer to 8 more) and that due to haloperidol decreased by 2 per 1000 (95%CI: 6 fewer to 13 more). There were no studies that investigated serious adverse events regarding the prophylactic administration of atypical antipsychotics or statins, or alternatively, showed no adverse events in either the intervention group or control group, and the estimated value of undesirable effects was unknown.

The onset of undesirable effects regarding dexmedetomidine was trivial, and moderate desirable effects were observed as regards the incidence of post-ICU-discharge cognitive disorders and delirium; thus, it was adjudged that interventions were likely superior. The desirable effects of haloperidol were limited, and undesirable effects were trivial; therefore, it was adjudged that neither intervention nor comparative controls was superior to the other. Desirable effects for delirium onset were observed for atypical antipsychotics; however, the research subjects were only post-operative patients, and it was adjudged that the desirable effects were trivial. Furthermore, the undesirable effects were unknown. Therefore, there was insufficient evidence for the utility of prophylactic administration of atypical antipsychotics among sepsis patients, and it was adjudged that neither intervention nor the comparative controls were superior to the other. Desirable effects were limited for statins, and undesirable effects were also trivial; thus, it was adjudged that neither intervention nor comparative controls were superior.

**CQ10-5: Should drug therapy be used to treat delirium in adult patients with sepsis?**

***Answer:*** We suggest against administering dexmedetomidine for delirium treatment in adult patients with sepsis (GRADE 2D: certainty of evidence = “very low”). We suggest against administering haloperidol (GRADE 2C: certainty of evidence = “low”). We suggest against administering atypical antipsychotics (GRADE 2B: certainty of evidence = “moderate”).

***Remarks:*** The use of dexmedetomidine, haloperidol, or atypical antipsychotics should not be prevented when the patient’s life or body is at risk due to hyperactive delirium.

***Rationale***

The results of a systematic review yielded the following RCTs that conformed to the PICO criteria: one on dexmedetomidine [537], one on haloperidol [538], and three on atypical antipsychotics [538–540]. A meta-analysis was performed on these RCTs. The results of a systematic review yielded 1 RCT (*n* = 71) including post-operative patients. In this RCT, dexmedetomidine administration resulted in a higher mortality (RR 4.13, 95%CI: 0.21–82.95) and 1.37 days shorter ICU stay (95%CI: 3.82 shorter to 1.08 longer). For haloperidol, the mortality rate (1 RCT, *n* = 376) was 38 more per 1000 (95%CI: 51 fewer to 154 more), number of days with delirium (1 RCT, *n* = 376) was 0.34 days shorter (95%CI: 1.18 shorter to 0.5 longer), and the length of stay in the ICU (1 RCT, *n* = 376) was 0.33 days shorter (95%CI: 1.92 shorter to 1.26 longer). For atypical antipsychotics, the mortality rate (2 RCTs, *n* = 410) was 3 fewer per 1000 (95%CI: 82 fewer to 98 more), the number of days with delirium (2 RCTs, *n* = 410) was 1.75 days shorter (95%CI: 4.31 shorter to 0.81 longer), and the length of stay in the ICU (2 RCTs, *n* = 410) was 1.1 shorter (95%CI: 2.48 shorter to 0.28 longer). Therefore, it was adjudged that the desired effects for each drug were trivial. Meanwhile, there were no reports on serious adverse events as outcomes of the three drugs. Therefore, the desirable effects of the three drugs were trivial, and the undesirable effects were unknown. The balance of effects was thought to be such that neither the intervention nor the comparative controls were superior.

**CQ10-6: Should non-drug therapy be used to prevent delirium in adult patients with sepsis?**

***Answer:*** We suggest using non-drug therapy to prevent delirium in adult patients with sepsis (GRADE 2C: certainty of evidence = “low”).

***Rationale***

Non-pharmacologic therapies evaluated as interventions included sleep improvement (e.g., eye masks, earplugs, and circadian rhythm improvement), arousal promotion (e.g., glasses, hearing aids, and orientation improvement), and relaxation (excluding rehabilitation medicine). The results of a systematic review yielded 10 RCTs that conformed to the PICO criteria [541–550] and we performed a meta-analysis using these studies.

The results of another systematic review including post-operative patients showed that the estimated value of the effects of mortality (4 RCTs, *n* = 884) was 15 fewer per 1000 (95%CI: 57 fewer to 42 more). That of cognitive dysfunction following discharge from the ICU (based on mini mental state examination) (1 RCT, *n* = 32) was 0.2 points higher (95%CI: 1.27 lower to 1.67 higher), and that for the number of delirium-free days (2 RCTs, *n* = 799) was 0.01 days longer (95%CI: 1.22 shorter to 1.24 longer). The incidence rate of delirium (6 RCTs, *n* = 1028) decreased by 44 per 1000 (95%CI: 149 fewer to 131 more) and that for the length of stay in the ICU (5 RCTs, *n* = 904) was 0.14 days shorter (95%CI: 1.06 shorter to 0.79 longer). Based on the above, the desired effects due to the intervention were judged to be small. Meanwhile, no studies have reported serious adverse events.

Therefore, the desirable effects were small, and the undesirable effects were unknown. However, it is thought that almost no undesirable effects were estimated from the content of the intervention. Based on the above, it was adjudged that the intervention was likely superior.

**CQ11: Acute kidney injury/blood purification**

**Introduction**

AKI is a pathological condition in which the homeostasis of the human body is disrupted due to a rapid decline in renal function. The clinical practice guidelines for AKI were published by the Kidney Disease Improving Global Outcomes (KDIGO) organization in 2012 and presented new AKI diagnostic criteria and severity classifications. AKI can be diagnosed using this standardized definition, and the significant impact of AKI on the outcomes has become clear in various clinical settings.

AKI is a syndrome characterized by a wide spectrum of diseases. Sepsis has frequently been observed as an etiology of AKI, and a poor prognosis has been reported for septic AKI [551]. The mortality rate of patients with severe AKI requiring renal replacement therapy (RRT) as a complication of sepsis is particularly high, and an analysis of the Japanese Diagnosis Procedure Combination database showed that the in-hospital mortality rate of these patients with severe AKI was approximately 50% [552]. The pathophysiology of septic AKI is complex, and disorders, such as those of the inflammatory response and mitochondrial dysfunction, are assumed to contribute to the pathogenesis of AKI in addition to dysregulated hemodynamics [553]. Meanwhile, no drugs have been clinically proven to reduce the incidence of AKI. Diuretics are commonly administered for septic AKI, with the aim of fluid management. Therefore, this guideline adopted CQs related to the administration of furosemide (11–1) and atrial natriuretic peptides (11–2). A CQ related to dopamine has also been adopted to confirm the role of dopamine (11–3) in septic AKI.

Blood purification therapy is a treatment modality that removes the causative agent in the blood via an extracorporeal blood circulation and replaces deficient substances. Among these, RRT is the most commonly used blood purification therapy. There is no firmly established evidence regarding the optimal RRT conditions for AKI. Therefore, this guideline adopted CQs regarding the selection of continuous or intermittent RRT (11–4), the timing of RRT initiation (11–5), and treatment doses in RRT (11–6). With regard to the time of initiation of RRT in particular, the STARRT AKI study was published just after the evidence was evaluated in this guideline [554]. This RCT does not support early initiation; therefore, we adjudged that this study was not in conflict with the recommendations made in the present guideline.

Endotoxin adsorption therapy is another blood purification therapy for sepsis other than RRT. This therapy has been developed in Japan, and many RCTs have been conducted recently on this therapy outside Japan. The present guideline adopted this as a CQ to evaluate the evidence (11–7).

**Fig. 10:**
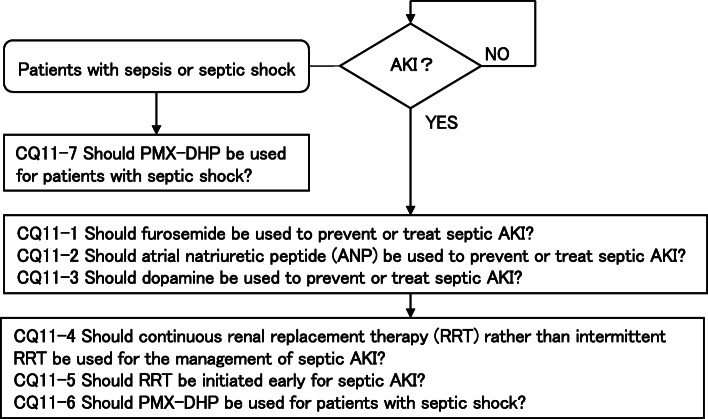
CQ11: Acute kidney injury/blood purification (clinical flow)

Clinical flow of these CQs is shown in Fig. 10.

**CQ11-1: Should furosemide be used to prevent or treat septic AKI?**

***Answer:*** We suggest against using furosemide for preventing or treating septic AKI (GRADE 2C, certainty of evidence = “low”).

***Rationale***

Furosemide could be theoretically beneficial for maintaining urine flow to prevent the obstruction of the renal tubules and reducing the oxygen consumption capacity of the renal tubules [555–557]. To examine these renal protective effects of furosemide, various clinical studies have been conducted since the 1980s. Unfortunately, these trials have failed to show the efficiency of renal protection by furosemide [558]. However, furosemide is widely used for fluid management in sepsis treatment; thus, it was thought that we should continue to include this issue in this guideline.

Our systematic review aimed to extract RCTs that comparatively examined furosemide administration and placebo, standard treatment, or no treatment among adult patients who were critically ill or with sepsis or septic shock. Unfortunately, our literature search found no relevant RCTs in which furosemide was administered for the prevention of AKI. Meanwhile, six eligible RCTs in which furosemide was used for treating AKI were identified. Then, the results of the extracted RCTs were integrated [559–564].

The estimated value of effects for in-hospital mortality (6 RCTs, *n* = 649) yielded an increase of 39 per 1000 (95%CI: 26 fewer to 122 more). Also, the requirement for renal replacement therapy (3 RCTs, *n* = 206) increased by 40 more per 1000 (95%CI: 103 fewer to 299 more). Thus, there were no clear benefits of furosemide administration for the treatment of AKI. Regarding the evidence certainty, the directionality of the estimated value of the effects was consistent among the above critical outcomes. Hence, the overall certainty of the evidence was set as “low”, the same as the highest certainty among the applied outcomes.

This CQ about furosemide is related to preventing and treating septic AKI and not to correcting fluid overload. In case of excessive body fluids, appropriate fluid management with diuretics including furosemide should be prioritized.

**CQ11-2: Should atrial natriuretic peptide (ANP) be used to prevent or treat septic AKI?**

***Answer:*** We suggest against using ANP for preventing or treating septic AKI (GRADE 2D, certainty of evidence = “very low”).

***Rationale***

Atrial natriuretic peptide (ANP) has been approved in some countries as a therapeutic drug for acute heart failure. Therefore, its possible effects on AKI have been investigated mainly for cardiovascular surgery patients [565–567]. Additionally, recent basic research of ANP also indicated the cardiovascular effect and the renal protective one [568–570]. However, its effects on septic AKI have been controversial as recent meta-analysis mentioned [565–567, 571]. Thus, this topic was picked up as an important CQ in this guideline.

Our systematic review aimed to extract RCTs that compared ANP administration to a placebo, standard treatment, or no treatment among adult patients who were critically ill or with sepsis or septic shock. Unfortunately, our literature search found no relevant RCTs in which ANP was administered to prevent AKI. Meanwhile, three eligible RCTs in which ANP was used for treating AKI were identified. Then, the results of the extracted RCTs were integrated [572–574].

The estimated value of effects for the requirement for RRT (3 RCTs, *n* = 779) decreased by 58 per 1000 (95%CI: 157 fewer to 73 more). Meanwhile, the mortality outcomes (3 RCTs, *n* = 779) showed an increase of 18 per 1000 (95%CI: 57 fewer to 110 more). Hence, the desired effects of ANP for the AKI treatment were thought to be trivial. On the other hand, hypotension has been reported as a side effect of this drug. The side effect could be harmful to the hemodynamics of sepsis or septic shock patients. Therefore, we suggest against using this drug to treat septic AKI.

The directionality of the desired and undesired effects of the integrated results was inconsistent among the examined outcomes. Thus, the evidence certainty was assessed as “very low.”

**CQ11-3: Should dopamine be used to prevent or treat septic AKI?**

***Answer:*** We suggest against using dopamine for preventing or treating septic AKI (GRADE 2C, certainty of evidence = “low”).

***Rationale***

Dopamine was used as a renal protective pressor agent because it was assumed to provide renal vasodilation, increase the glomerular filtration rate, and yield a natriuretic effect when administered at a low dose of 1–3 μg/kg/min. However, its effectiveness has been rejected mainly by RCTs conducted in the 2000s [575–577]. Nevertheless, given its use under the term of “renal dose” in clinical settings, we have decided to choose this as an important clinical issue.

A systematic review extracted RCTs that comparatively investigated dopamine administration with a placebo, standard treatment, or no treatment among adult patients who were critically ill, or who had infection, sepsis, or septic shock. The results showed that there were no relevant RCTs in which dopamine was administered to prevent AKI. Meanwhile, one RCT in which dopamine was administered for the purposes of treating AKI was found [578].

The estimated value of effects for mortality at the time of discharge from the ICU decreased by 25 per 1000 (95%CI: 114 fewer to 89 more). That of a requirement for renal replacement therapy yielded a decrease of 27 per 1000 (95%CI: 98 fewer to 79 more), suggesting that the desired effects of dopamine were trivial. The estimated value of effects for mortality at the time of hospital discharge yielded an increase of 24 per 1000 (95%CI: 73 fewer to 150 more), suggesting that the undesired effects were trivial. Therefore, we suggest against using dopamine as a standard treatment.

The directionalities of the two important outcomes, mortality at the time of discharge from the ICU and mortality at the time of hospital discharge, were inconsistent; thus, the overall certainty of the evidence was set as “low.”

**CQ11-4: Should continuous renal replacement therapy (RRT) rather than intermittent RRT be used for the management of septic AKI?**

***Answer:*** Either continuous or intermittent RRT can be selected for septic AKI (GRADE 2C, certainty of evidence = “low”). Continuous RRT should be used for hemodynamically unstable patients (Good Practice Statement).

***Rationale***

RRT is an essential treatment for life support among patients with highly advanced septic AKI. Modalities that are currently in use for RRT include continuous or intermittent RRT; however, the use of either one for septic AKI depends on not only pathological conditions, but also the experience and care system of the medical facility. Meanwhile, observational studies have reported that there is a tendency to select continuous renal replacement therapy (CRRT) under conditions of circulatory instability. Therefore, it was determined that this selection was important in terms of deciding the treatment strategy, and it was chosen as a CQ.

We extracted RCTs that comparatively investigated either CRRT or intermittent renal replacement therapy (IRRT) in adult septic AKI patients or those who had AKI due to severe illness. Among the 5 extracted RCTs, one RCT showed significant differences in severity after random allocation [579–583]. As the certainty of the evidence in these 5 RCTs becomes very low, we integrated the results of the 4 RCTs after excluding this RCT [580–583].

The estimated value of the effects for mortality outcomes yielded a decrease of 6 fewer per 1000 (95%CI: 69 fewer to 63 more), that of dialysis dependence yielded a decrease of 28 per 1000 (95%CI: 61 fewer to 68 more), and that of combined outcomes between dialysis dependence and mortality decreased by 42 per 1000 (95%CI: 185 fewer to 158 more). Furthermore, hemorrhaging complications decreased by 3 per 1000 (95%CI: 29 fewer to 46 more). Therefore, it was adjudicated that the desired effects due to CRRT were trivial. Meanwhile, no clear undesired effects were observed; thus, the balance of effects was adjudicated such that CRRT was slightly superior. However, the certainty of evidence was low, and it was clear that the workload on medical staff in the case of CRRT was higher than that in IRRT. Based on the above, a conclusion could not be reached as to whether CRRT was superior to IRRT.

Meanwhile, there were no RCTs that compared CRRT and IRRT in patient groups with unstable hemodynamics. However, the current state in actual clinical practice is such that CRRT is selected for patients with unstable hemodynamics, and we decided to recommend this as a good practice statement.

**CQ11-5-1: Should RRT be initiated early for septic AKI (Stage 2 vs. Stage 3 or absolute indications)?**

***Answer:*** We make no recommendation on whether RRT should be initiated early at Stage 2 for patients with septic AKI.

**CQ11-5-2: Should RRT be initiated early for septic AKI (Stage 3 vs. absolute indications)?**

***Answer:*** We suggest against initiating RRT at Stage 3 for patients with septic AKI rather than absolute indication (GRADE 2D, certainty of evidence = “very low”).

***Rationale***

It is uncertain when to initiate RRT for patients with AKI accompanied by sepsis. Early intervention with RRT before patients meets the criteria for absolute indications may sound promising; however, unnecessary RRT increases risks of complications and can be harmful. The uncertainty of the timing has been addressed in RCTs that adopted different AKI stages as early intervention. Accordingly, the CQ has two answers according to the definitions of early and late initiation of RRT.

RCTs comparing the timing of RRT at any stage of AKI or absolute indications in patients with AKI were retrieved. The systematic review yielded 1 RCT that compared the initiation of RRT at stage 2 with stage 3 AKI or absolute indications and 2 RCTs that compared the initiation of RRT at stage 3 with absolute indications [584–586].

The RCT by Zarbock et al. reported early initiation of RRT at stage 2 AKI had beneficial effects on mortality and a composite outcome of mortality and dialysis dependence. However, adverse events, i.e., hemorrhagic complications, were not reported in the article, which limited the balanced interpretation of the effects [584]. Furthermore, the trial was conducted at a single center; as such, the results were adjudicated insufficient to be applied into clinical practice. Therefore, the guideline committee decided not to provide a recommendation on whether to start RRT at stage 2 AKI in patients with sepsis [584].

From the 2 RCTs that compared RRT initiation at stage 3 AKI with absolute indications, mortality toward increased and no difference observed in the composite outcome of mortality and dialysis dependence [585, 586]. On the contrary, hemorrhagic complications decreased slightly with the early RRT at stage 3 [585, 586]. The available evidence showed no beneficial effects of initiating RRT at stage 3, albeit no apparent harms. Given that early initiation of RRT inherits issues of increased costs and workload, we suggest against initiating RRT at stage 3 AKI.

**CQ11-6: Should a large RRT dose be delivered for septic AKI?**

***Answer:*** We suggest against increasing a RRT dose beyond the standard dose for patients with septic AKI (GRADE 2C, certainty of evidence = “low”).

***Rationale***

An improved prognosis might be expected by increasing the clearance of inflammatory cytokines and various mediators when performing RRT among septic AKI patients, and clinical investigations were conducted on increasing the doses in dialysis/filtration. Therefore, appropriately setting the prescribed dose of RRT is important in the treatment of septic AKI, and this was chosen as a CQ to be investigated. Although the standard prescribed dose in Japanese insurance practice is approximately 15 mL/kg/h, the international standard dose is 25 mL/kg/h; thus, attention is needed when interpreting the results of research conducted outside Japan.

RCTs that compared RRT at high doses against septic AKI with RRT at low doses were extracted. A total of 6 RCTs were extracted [587–592]. RCTs that were conducted using extremely high doses (≥50 mL/kg/h) were very different from the real world clinical practice in Japan and were excluded from this analysis [588, 591, 592].

The results of integrating the three extracted RCTs showed that the estimated values of effects of mortality outcomes (3 RCTs, *n* = 2789) yielded an increase of 22 per 1000 (95%CI: 13 fewer to 58 more), and those of dialysis dependence (3 RCTs, *n* = 2096) and combined outcomes of dialysis dependence and mortality (3 RCTs, *n* = 2786) yielded increases of 22 per 1000 (95%CI: 9 fewer to 57 more) and 12 per 1000 (95%CI: 12 fewer to 43 more), respectively [587, 589, 590]. The desired and undesired effects were adjudicated as “unknown” and “trivial,” respectively. Therefore, the balance of effects was such that the comparative control was likely superior.

Furthermore, RRT at high doses slightly increases medical costs, induces frequent dialysis fluid/replacement fluid exchange and filter/circuit clotting, and increased the workload on the medical staff. Based on the above, we suggest against increasing the amount of RRT doses to that above standard levels.

Regarding the certainty of evidence, all serious outcomes were evaluated as “low” and had the same directionality; thus, the overall certainty of evidence was also set as “low.”

**CQ11-7: Should PMX-DHP be used for patients with septic shock?**

***Answer:*** We suggest against using PMX-DHP for patients with septic shock (GRADE 2B, certainty of evidence = “moderate”).

***Rationale***

Direct hemoperfusion with polymyxin B-immobilized fiber column (PMX-DHP) was developed in Japan and is expected to improve the pathophysiological derangements of sepsis through endotoxin removal [593]. As the treatment involves extracorporeal circulation, risks of adverse events should also be considered. Systematic reviews of RCTs assessing its effectiveness and adverse events had been published previously [594–599].

An update of the systematic review was conducted for the guideline to assess the effects of PMX-DHP in patients with septic shock. RCTs that compared PMX-DHP with sham perfusion or usual care were retrieved, and three relevant trials were identified through the databases search [600–602]. Meta-analysis reported that the overall mortality at the longest follow up increased by 12 per 1000 (95%CI: 123 fewer to 223 more) and any adverse events as defined in each trial yielded an increase of 17 per 1000 (95%CI; 19 fewer to 58 more). The beneficial effects were not observed, and harms increased slightly. As such, the PMX-DHP was adjudicated to be inferior to the control or usual care. The guideline committee suggest against the use of PMX-DHP for patients with septic shock.

Two prespecified critically important outcomes, i.e., mortality and adverse events, indicated toward harm. GRADE assessment for mortality was very low and that for adverse events were moderate. Accordingly, the certainty of evidence for the recommendation was adjudicated to be moderate.

**CQ12: Nutrition support therapy**

**Introduction**

This guideline covers a total of 9 CQs, with 8 basic CQs on administration of nutrition to septic patients and one CQ relating to vitamins C and D, which have attracted attention in recent years. Systematic reviews were performed for 7 of these CQs; however, there was little evidence that was limited to only patients with sepsis. Thus, our recommendations are based on RCTs that assessed critically ill patients commensurate with septic patients.

CQ12–1 relates to whether enteral or parenteral nutrition should be prioritized. Enteral nutrition is thought to suppress bacterial translocation by maintaining the structure of the intestinal flora and intestinal mucosa as well as the function of gut-associated lymphoid tissue. Therefore, an investigation was conducted to determine whether enteral nutrition was actually beneficial compared to parenteral nutrition. For CQ12–2, a systematic review was performed on the benefits and harms of initiation of enteral nutrition in hemodynamically unstable patients. Serious gastrointestinal complications such as intestinal ischemia and ischemic enteritis, which are problems in enteral nutrition among hemodynamically unstable patients, were set as serious outcomes. In CQ12–3, the balance of benefits and harms of initiation of enteral nutrition within 24–48 h of initiation of treatment for severe illnesses compared to initiation after this period was investigated.

The amount of nutrition administered during enteral nutrition was investigated in CQ12–4. A systematic review compared groups that received an amount of energy either less than that consumed or roughly equivalent. The former includes trophic feeding, which is about one-fourth of the amount of consumed energy or 500 kcal/day (20 kcal/hr) and permissive underfeeding/hypofeeding, which involves mild energy restrictions with about 60–70% of the amount of consumed energy administered. The latter includes cases that begin with small amounts and ultimately aims to administer an amount commensurate with the energy consumed, or methods that aim from the outset to administer an amount of energy commensurate with that consumed and decrease the amount when the residual gastric amount increases or when symptoms of intolerance such as diarrhea occur. In CQ12–5, we examined whether supplemental parenteral nutrition should be added when the target amount of energy cannot be administered via enteral nutrition alone. In CQ12–6, we investigated the optimal protein dose in the acute phase. The systematic review compared doses less than 1 g/kg/day and more than 1 g/kg/day because the currently recommended dose of protein administration in Japan is less than 1 g/kg/day [603] and the lower recommended limit in several guidelines was 1.2–1.3 g/kg/day [604–606].

In CQ12–7, we investigated the administration of vitamins C and D. This has attracted increased attention following the report that in-hospital mortality significantly improved with the administration of vitamin C in patients with sepsis [607]. However, an RCT published in 2020 reported no improvements in 28-day or 90-day mortality [608]. For vitamin D as well, ICU patients with vitamin D deficiency were reported to have a worse prognosis [609], and an RCT reported that supplementation tended to improve the mortality rate [610]. An RCT published in 2019 reported that vitamin D yielded no benefits [611]. Therefore, a systematic review was performed to verify the effects of administration of vitamins C and D.

CQ12–8 is a BQ related to the initiation and tolerance of enteral nutrition, and CQ12–9 is also a BQ that provides information on nutritional therapy following the acute phase.

**Fig. 11:**
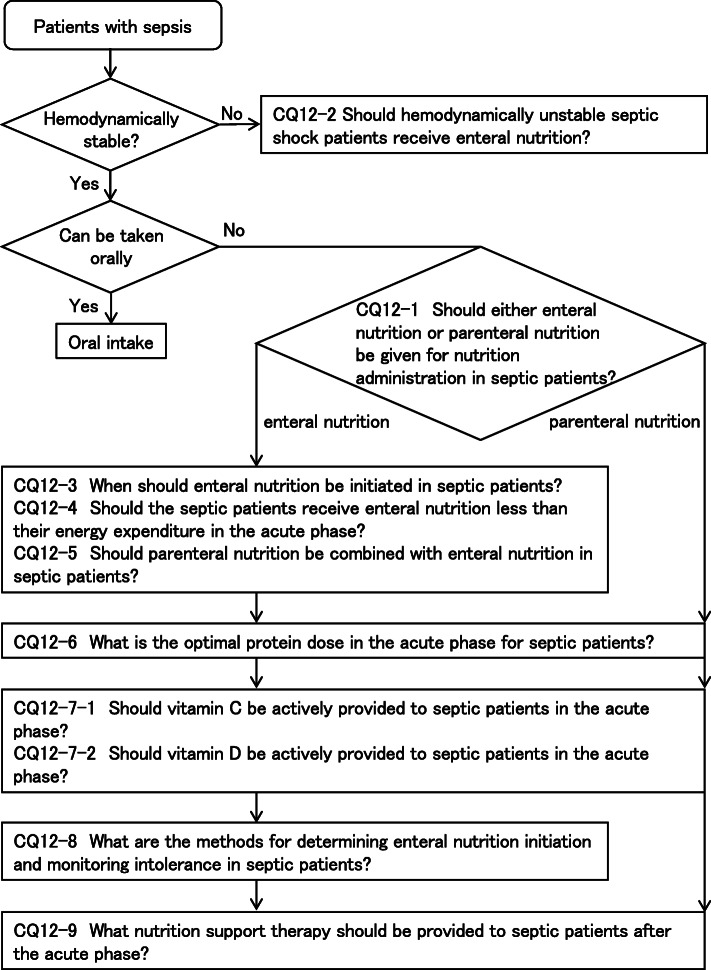
CQ12: Nutrition support therapy (clinical flow)

Clinical flow of these CQs is shown in Fig. 11.

**CQ12-1: Should either enteral nutrition or parenteral nutrition be given for nutrition administration in septic patients?**

***Answer:*** We suggest enteral nutrition be administered for septic patients. (GRADE 2D: certainty of evidence = “very low”).

***Rationale***

A meta-analysis was performed on 24 RCTs [612–635]. The estimated values of the desirable anticipated effects were as follows: bloodstream infection yielded a RD of 19 fewer per 1000 (95%CI: 32 fewer to 4 more) (9 RCTs, *n* = 2976), pneumonia yielded an RD of 18 fewer per 1000 (95%CI: 41 fewer to 12 more) (8 RCTs, *n* = 3066), abdominal infections yielded an RD of 39 fewer per 1000 (95%CI: 46 fewer to 30 fewer) (7 RCTs, *n* = 3159), the duration of mechanical ventilation yielded a MD of 0.36 days shorter (95%CI: 0.93 shorter to 0.2 longer) (4 RCTs, *n* = 563), and the length of stay in hospital yielded an MD of 2.51 days shorter (95%CI: 4.78 shorter to 0.24 shorter) (10 RCTs, *n* = 5515). The desirable anticipated effect was determined to be moderate based on these results. Meanwhile, the estimated value of 90-day mortality yielded an RD of 20 more per 1000 (95%CI: 20 fewer to 68 more) (4 RCTs, *n* = 4844) as an undesirable anticipated effect. Thus, the undesirable anticipated effect was determined to be trivial. Therefore, we concluded that enteral nutrition was likely superior to parenteral nutrition.

**CQ12-2: Should hemodynamically unstable septic shock patients receive enteral nutrition?**

***Answer:*** We suggest against administering enteral nutrition in hemodynamically unstable septic shock patients (GRADE 2D: certainty of evidence = “very low”).

***Rationale***

A meta-analysis was performed using 1 RCT [627]. The estimated values of desirable anticipated effects were as follows: infections acquired in the ICU yielded a RD of 16 fewer per 1000 (95%CI: 42 fewer to 13 more); the length of stay in hospital yielded an RD of 1.00 days shorter (95%CI: 2.42 shorter to 0.42 longer) (1 RCT, *n* = 2410). It was adjudged from these results that the desirable anticipated effect was trivial. Meanwhile, the estimated values of the undesirable anticipated effects were as follows: 90-day mortality yielded an RD of 21 more per 1000 (95%CI: 17 fewer to 63 more), gastrointestinal pseudo-obstructions yielded an RD of 7 more per 1000 (95%CI: 0 to 30 more), and intestinal ischemia yielded an RD of 12 more per 1000 (95%CI: 2 more to 38 more) (1 RCT, *n* = 2410). From the above, it was adjudged that the undesirable anticipated effect was small. Thus, we thought that enteral nutrition was not superior to parenteral nutrition in this population.

**CQ12-3: When should enteral nutrition be initiated in septic patients?**

***Answer:*** We suggest initiating enteral nutrition at an early period of acute phase (within 24–48 h following the start of treatment to critical illness) for septic patients (GRADE 2D: the certainty of evidence = “very low”).

***Rationale***

A meta-analysis was performed using 13 RCTs [636–648]. The estimated values of the desirable anticipated effects were as follows: mortality yielded a RD of 27 fewer per 1000 (95%CI: 63 fewer to 25 more) (13 RCTs, *n* = 709); pneumonia yielded an RD of 85 fewer per 1000 (95%CI: 173 fewer to 41 more) (6 RCTs, *n* = 441). It was judged from these results that the desirable anticipated effects were moderate. Meanwhile, the estimated values of the undesirable anticipated effects were as follows: bacteremia yielded an RD of 48 more per 1000 (95%CI: 69 fewer to 240 more) (6 RCTs, *n* = 354) and length of stay in hospital yielded a MD of 0.41 days longer (95%CI: 2.71 shorter to 3.53 longer) (5 RCTs, *n* = 217). Based on the above, it was judged that the undesirable anticipated effects were small. Therefore, we concluded that early enteral nutrition was superior to late enteral nutrition.

**CQ12-4: Should the septic patients receive enteral nutrition less than their energy expenditure in the acute phase?**

***Answer:*** We suggest the septic patients receive enteral nutrition less than their energy expenditure in the acute phase. (GRADE 2B: certainty of evidence = “moderate”).

***Rationale***

We performed a meta-analysis of 18 RCTs [647, 649–665]. The estimated values of desirable anticipated effects were as follows: mortality yielded a RD of 2 fewer per 1000 (95%CI: 23 fewer to 21 more) (18 RCTs, *n* = 12,679), the length of hospital stay yielded a MD of 0.35 days shorter (95%CI: 2.68 shorter to 1.99 longer) (10 RCTs, *n* = 6728), all-cause infections yielded an RD of 3 fewer per 1000 (95%CI:44 fewer to 47 more) (11 RCTs, *n* = 6245), pneumonia yielded an RD of 25 fewer per 1000 (95%CI: 50 fewer to 4 more) (10 RCTs, *n* = 7778), bacteremia yielded an RD of 6 fewer per 1000 (95%CI: 18 fewer to 11 more) (9 RCTs, *n* = 10,768), and catheter-related infections and bloodstream infections yielded an RD of 19 fewer per 1000 (95%CI: 34 fewer to 15 more) (5 RCTs, *n* = 1608). It was judged from these results that the desirable anticipated effects were small. Meanwhile, no judgement could be made on undesirable anticipated effects because there were no reports of serious adverse effects. Based on the above, we thought that hypocaloric enteral nutrition is superior to eucaloric enteral nutrition.

**CQ12-5: Should parenteral nutrition be combined with enteral nutrition in septic patients?**

***Answer:*** We suggest supplemental parenteral nutrition be combined in septic patients receiving insufficient amount of enteral nutrition (GRADE 2D: certainty of evidence = “very low”).

***Rationale***

A meta-analysis was performed using 5 RCTs [656, 665–668]. The estimated values of the desirable anticipated effects were as follows: 90-day mortality yielded a RD of 18 fewer per 1000 (95%CI: 138 fewer to 195 more) (1 RCT, *n* = 120); respiratory infections yielded an RD of 64 fewer per 1000 (95%CI: 143 fewer to 49 more) (4 RCTs, *n* = 624). It was adjudged from these results that the desirable anticipated effects were moderate. Meanwhile, the estimated values of the undesirable anticipated effects were as follows: bloodstream infection yielded an RD of 6 more per 1000 (95%CI: 62 fewer to 293 more) (3 RCTs, *n* = 504), urinary tract infections yielded an RD of 25 more per 1000 (95%CI: 40 fewer to 199 more) (3 RCTs, *n* = 550), and abdominal infections yielded an RD of 52 more per 1000 (95%CI: 28 fewer to 1000 more) (2 RCTs, *n* = 430). From the above, it was adjudged that the undesirable anticipated effects were moderate. Thus, we thought that enteral nutrition with supplemental parenteral nutrition was superior to enteral nutrition alone.

**CQ12-6: What is the optimal protein dose in the acute phase for septic patients?**

***Answer:*** We suggest providing less than 1 g/kg/day of protein (peptides, amino acids) to septic patients in the acute phase (GRADE 2D: certainty of evidence = “very low”).

***Rationale***

A systematic review was performed on trials that separated critically ill patients undergoing treatment in the ICU between intervention groups with an acute dose of peptides (proteins and amino acids) administered at levels of over 1 g/kg/day and control groups with doses lower than 1 g/kg/day. We then performed a meta-analysis using 6 RCTs [669–674]. The estimated values of the desirable anticipated effects were as follows: mortality yielded a RD of 4 fewer per 1000 (95%CI: 51 fewer to 62 more) (5 RCTs, *n* = 730), physical function evaluation yielded a MD of 0.45 higher (95%CI: 4.57 lower to 5.46 higher) (3 RCTs, *n* = 489), and muscle mass yielded an MD of 0.2 higher (95%CI: 0.56 lower to 0.96 higher) (2 RCTs, *n* = 157). It was adjudged that the desirable anticipated effects were trivial. Meanwhile, the estimated values of the undesirable anticipated effects were as follows: length of stay in hospital yielded an MD of 2.36 days longer (95%CI: 1.42 shorter to 6.15 longer) (5 RCTs, *n* = 733); length of mechanical ventilation yielded an MD of 0.07 days longer (95%CI: 0.02 shorter to 0.16 longer) (5 RCTs, *n* = 777), and duration of antibiotic treatment yielded an MD of 0.15 days longer (95%CI: 0.07 longer to 0.23 longer) (1 RCT, *n* = 474). It was adjudged that the undesirable anticipated effects were small. From the above, we thought that protein administration at a dose lower than 1 g/kg/day was superior to that at a dose of more than 1 g/kg/day.

**CQ12-7-1: Should vitamin C be actively provided to septic patients in the acute phase?**

***Answer:*** We suggest providing vitamin C to septic patients (GRADE 2D: certainty of evidence = “very low”).

***Rationale***

A meta-analysis was performed using 11 RCTs [608, 675–684]. The estimated values of the desirable anticipated effects were as follows: 28-day mortality yielded a RD of 55 fewer per 1000 (95%CI: 131 fewer to 52 more) (5 RCTs, *n* = 1646), in-hospital mortality yielded an RD of 25 fewer per 1000 (95%CI: 105 fewer to 83 more) (7 RCTs, *n* = 1798), the length of stay in the ICU yielded a MD of 0.58 days shorter (95%CI: 1.45 shorter to 0.28 longer) (6 RCTs, *n* = 1394), and AKI yielded an RD of 18 fewer per 1000 (95%CI: 111 fewer to 92 more) (2 RCTs, *n* = 248). Of these two RCTs which used AKI as an outcome (Fujii et al., 2020 [608]; Tanaka et al., 2000 [677]), that conducted by Tanaka et al. (2000) [677] was a small-scale study (37 patients) and reported an AKI incidence rate of 0% for both the intervention and control groups. The estimated value of effects for AKI was largely due to the report published by Fujii et al. (2020) [608]. The study showed slightly decreasing tendencies for both 28-day and 90-day mortality, and this was thought to imply an improvement in extremely serious outcomes for patients, such that the desirable anticipated effect was judged to be “small”. Meanwhile, the estimated value of length of hospital stay yielded an MD of 0.64 days longer (95%CI: 1.24 shorter to 2.52 longer) (5 RCTs, *n* = 1556) as the undesirable anticipated effect. The length of stay in the hospital tended to be prolonged due to vitamin C administration; however, this duration was thought to be extremely short. Based on the above, it was thought that the undesirable anticipated effects were “trivial”. Thus, we thought that vitamin C was superior to placebo or control.

**CQ12-7-2: Should vitamin D be actively provided to septic patients in the acute phase?**

***Answer:*** We suggest against providing vitamin D in septic patients (GRADE 2D: certainty of evidence = “very low”).

***Rationale***

We performed a meta-analysis of 11 RCTs [610, 611, 685–693]. The estimated values of the desirable anticipated effects were as follows: 28-day or 30-day mortality yielded a RD of 8 fewer per 1000 (95%CI: 50 fewer to 46 more) (6 RCTs [610, 611, 685, 688, 689, 691]: *n* = 1966), the 90-day mortality yielded an RD of 28 more per 1000 (95%CI: 18 fewer to 85 more) (3 RCTs [611, 690, 691], *n* = 1157), in-hospital mortality yielded an RD of 95 fewer per 1000 (95%CI: 180 fewer to 41 more) (4 RCTs [610, 686, 687, 693], *n* = 632), and the length of stay in hospital yielded a MD of 0.32 days shorter (95%CI: 2.15 shorter to 1.50 longer) (9 RCTs [610, 611, 685, 686, 688–692], *n* = 1886). The results showed that low vitamin D levels increased the 90-day mortality rate, had no effect on the 28-day or 30-day mortality rate, and decreased the in-hospital mortality rate. It was adjudged that the desirable anticipated effects of vitamin D administration were “absent” or “trivial”. Meanwhile, the estimated value of hypercalcemia yielded an RD of 7 fewer per 1000 (95%CI: 20 fewer to 65 more) (5 RCTs [610, 611, 686, 688, 691], *n* = 1276), and it was adjudged that the undesirable anticipated effect was trivial. Thus, we thought that neither vitamin D nor placebo/control was superior.

**CQ12-8: What are the methods for determining enteral nutrition initiation and monitoring intolerance in septic patients?**

***Answer:*** Findings such as bowel sounds, which indicate contractility of the gastrointestinal tract, at the start of enteral nutrition should not be required. Meanwhile, various findings show intolerance following the initiation of enteral nutrition, including the lack of intestinal sounds, abnormal intestinal sounds, vomiting, intestinal dilation, diarrhea, gastrointestinal bleeding, and excessive gastric residue. Excessive gastric residue suggests intolerance, but the gastric residue volume criteria for determining the presence of intolerance are unknown (Provision of information for background question).

***Rationale***

Little research has been conducted on sepsis patients. Therefore, we outline the decisions on enteral nutrition start and tolerance based on findings obtained in studies of critically ill patients. Enteral nutrition should be initiated when the gastrointestinal tract is usable in hemodynamically stable patients. Details on the criteria for hemodynamic stability are presented in CQ12–2, and those on the start times of enteral nutrition are presented in CQ12–3.

The presence of bowel sounds and flatulence are routinely monitored when investigating the initiation of enteral nutrition. However, although the presence of bowel sounds indicates that the intestine is motile, this should not be implied as equivalent to the health of the gastrointestinal tract (e.g., intestinal permeability, barrier function, and absorption capacity). Furthermore, studies that compared groups of professionals that did or did not wait to listen to bowel sounds, flatulence, or watch for defecation when initiating enteral nutrition showed that there were no differences in patient prognosis [694]. The absorption capacity has been shown to decrease when enteral nutrition is delayed [638], and it is thought that, at the very least, there is no need to have bowel sounds as a prerequisite for starting enteral nutrition.

Gastrointestinal intolerance refers to a state in which gastrointestinal symptoms occur with enteral nutrition administration, and in which nutritional supplements cannot be sufficiently administered [695]. Gastrointestinal intolerance presents with many symptoms, including vomiting, abdominal pain, excessive gastric residual volume, bloating, flatulence, gastrointestinal bleeding due to gastric stasis, intestinal obstruction, intestinal ischemia, diarrhea due to increased peristalsis, and decreased absorption capacity. However, there are no clear criteria for the assessment of gastrointestinal tolerance, and decisions such as treating the underlying disease, using intestinal prokinetic drugs, and reducing/suspending enteral nutrition need to be individually made by identifying the diseases that cause these symptoms.

Gastric residual volume is also considered as a feature of gastrointestinal intolerance. However, gastric residual volume has not been shown to be correlated with the incidence of pneumonia [696], gastric emptying capacity [697], and the incidence of reflux or aspiration [698]. Furthermore, vomiting has been found to decrease by measuring the gastric residual volume [699]. However, a report has also indicated that increase in feeding tube obstruction (which could be partly due to curd formation [solidification] of the proteins in the gastric contents refluxed at the time of measurement) and unnecessary enteral nutrition suspension (e.g., suspension even when the gastric residue is in a clinically non-problematic state), in turn reduce the enteral nutrition dose as a result and had no influence on prognosis [700]. Some researchers recommend suspending enteral nutrition and searching for the cause when more than 500 mL of fluid is withdrawn with a single round of suction [605]. However, the criteria for assessment of the gastric residual volume at which enteral nutrition should be reduced or suspended are unclear, and it can be said that there are insufficient data supporting the routine measurement of gastric residual volume.

**CQ12-9: What nutrition support therapy should be provided to septic patients after the acute phase?**

***Answer:*** Provision of energy that meets the goals (around 25–30 kcal/kg/day, including protein) is thought to be needed when the patients overcome the clinical conditions of acute phase, or where about one week has passed following the onset of critical illness. Some experts are of the opinion that protein dose of over 1 g/kg/day is ideal in this phase. However, there are other expert opinions that the energy dose should be increased at an earlier phase for patients with malnutrition prior to exacerbation of the disease (Provision of information for background question).

***Rationale***

As suggested in CQ12–4, there are cases where nutrition is intentionally administered at a level lower than the energy consumption in the acute phase, or in which the nutritional dose is reduced due to factors beyond one’s control. However, the energy debt created in these cases must be given due consideration. Energy debt is the cumulative difference between the amount of energy consumed and administered, and a larger energy debt has been reported to result in a worsened prognosis [701, 702]. Limited observational studies have shown the relationship between energy debt and prognosis, and these results may be affected by confounding factors. However, it is self-evident that large energy debts have negative influences on patients’ immunity and body composition, and it is thought that sufficient energy must be administered when transitioning from the acute phase to the recovery phase.

The transition from the acute phase to the recovery phase varies widely depending on the patient’s condition, and recovery-phase nutrition therapy should be substituted into the treatment when the patient is clinically deemed to have moved out of the acute phase. Many clinical trials of acute-phase nutritional therapy have an intervention limit of approximately 7 days [649, 653, 656, 660], and the general strategy has been to administer nutrition that satisfies the required energy (about 25–30 kcal/kg/day, including proteins) after that, including previous energy debts [604, 605]. As a reference, a RCT of acute lung injuries [703] showed that patient groups with high energy doses had a high mortality rate when nutrition was administered prior to the seventh day, and a conversely low mortality rate tendency when nutrition was administered on the eighth day onwards. These results suggest the need for a review of nutrition therapy when transitioning from the acute phase to the recovery phase.

Proteins may also need to be secured as well once patients recover from the acute phase. As discussed in CQ12–6, there is insufficient evidence as to how much protein (g/kg/day) should specifically be taken after the acute phase. However, a minimum protein provision of 1 g/kg/day is widely accepted and is the recommended dietary intake in healthy individuals.

There is an opinion that sufficient energy administration should be considered from the acute phase among patients with malnutrition (e.g., low body weight and decreased muscle mass). However, sudden energy administration to extremely malnourished patients can potentially induce refeeding syndrome, and it is necessary to strictly monitor the levels of phosphate, potassium, magnesium, and other electrolytes when feeding.

**CQ13: Blood glucose management**

**Introduction**

Glycemic control is important in patients with sepsis because hyperglycemia can worsen patients’ prognoses by affecting the immune system and exacerbating infectious diseases. In contrast, hypoglycemia is an important hazard of glycemic control using insulin, and its onset is associated with a worsened prognosis among critically ill patients [704]. Therefore, it is necessary to consider the balance between benefits and harms when setting the target blood glucose level. Furthermore, erroneous blood glucose level measurements can result in inappropriate insulin use. Based on the above, “target blood glucose level” and “blood glucose measurement method” were selected as CQs.

**Fig. 12:**
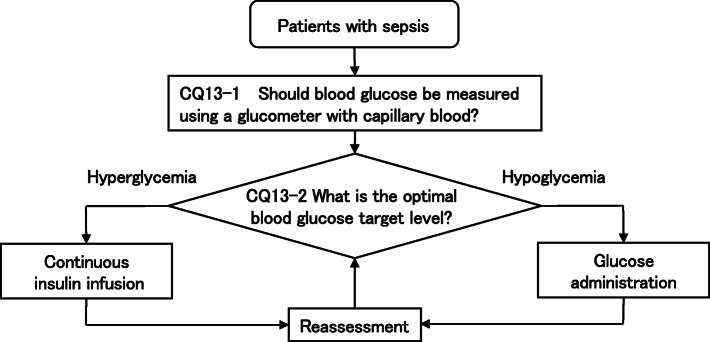
CQ13: Blood glucose management (clinical flow)

Clinical flow of these CQs is shown in Fig. 12.

**CQ13-1: Should blood glucose be measured using a glucometer with capillary blood in septic patients?**

***Answer:*** We suggest against the use of a glucometer with capillary blood in patients with sepsis (GRADE 2A: certainty of evidence = “high”).

***Rationale***

A meta-analysis was conducted using 43 observational studies. Measurement errors outside the acceptable range were evaluated by defining a value of ±20% of the blood glucose level in the laboratory as the acceptable range of error upon agreement. The estimated value of effects (per 1000 measurements) for the onset of measurement errors outside of the acceptable range yielded a RD of 45 more per 1000 (95%CI: 11 more to 164 more) (3 studies, *n* = 2800) [705–707] when the glucometer (capillary blood) was compared to the blood gas analyzer (arterial blood/venous blood). The RD was 58 more per 1000 (95%CI: 12 more to 134 more) (8 studies, *n* = 5924) [705–712] when the glucometer using capillary blood was compared to that using arterial blood/venous blood. The RD was 39 more per 1000 (95%CI: 14 more to 90 more) (3 studies, *n* = 5075) [705–707] when the glucometer (capillary blood) was compared to the blood gas analyzer/glucometer (arterial blood/venous blood). The RD was 10 fewer per 1000 (95%CI: 12 fewer to 0) (5 studies, *n* = 4321) [705–707, 713, 714] when the blood gas analyzer (arterial blood/venous blood) was compared to the glucometer (arterial blood/venous blood). Therefore, it was determined that the desirable anticipated effects of the glucometer using capillary blood were trivial. Hyperglycemia increases the incidences of mortality and infection, whereas hypoglycemia contributes to the incidences of neuropathy and mortality. Among patients in whom measurements have large errors, opportunities for rapid treatment may be lost. Measurement methods with glucometer using capillary blood had approximately 39 to 58 more measurement errors outside the acceptable range per 1000 measurements when compared to measurement methods with blood gas analyzers or glucometer using arterial blood/venous blood. Thus, the undesirable effects were moderate. Based on the above, we thought that measurement methods with either blood gas analyzers or glucometer using arterial blood/venous blood were likely superior to measurement methods with glucometer using capillary blood.

**CQ13-2: What is the optimal blood glucose target level in septic patients?**

***Answer:*** We suggest an optimal target blood glucose range of 144–180 mg/dL in septic patients (GRADE 2D: certainty of evidence = “very low”).

***Rationale***

A network meta-analysis was performed using 35 RCTs [404, 658, 715–747]. We divided target blood glucose levels into less than 110 mg/dL, 110–144 mg/dL, 144–180 mg/dL, and > 180 mg/dL. The results showed that the estimated values of mortality were as follows: when compared to < 110 mg/dL, a range of 110–144 mg/dL yielded a RD of 40 fewer per 1000 (95%CI: 100 fewer to 30 more) (1 RCT, *n* = 90), a range of 144–180 mg/dL yielded an RD of 27 fewer per 1000 (95%CI: 45 fewer to 8 fewer) (5 RCTs, *n* = 7323), and a range > 180 mg/dL yielded an RD of 4 more per 1000 (95%CI: 22 fewer to 35 more) (12 RCTs, *n* = 8027). When compared to a range of 110–144 mg/dL, 144–180 mg/dL yielded an RD of 6 more per 1000 (95%CI: 104 fewer to 147 more) (1 RCT, *n* = 20) and a range > 180 mg/dL yielded an RD of 28 more per 1000 (95%CI: 14 fewer to 81 more) (8 RCTs, *n* = 884). When compared to a range of 144–180 mg/dL, > 180 mg/dL yielded an RD of 1 more per 1000 (95%CI: 0 to 3 more) (1 RCT, *n* = 212). The estimated values of infection were as follows: when compared to a range < 110 mg/dL, 144–180 mg/dL yielded an RD of 5 fewer per 1000 (95%CI: 19 fewer to 10 more) (3 RCTs, *n* = 6185), and a range > 180 mg/dL yielded an RD of 25 more per 1000 (95%CI: 8 more to 43 more) (8 RCTs, *n* = 6104). When compared to a range of 110–144 mg/dL, > 180 mg/dL yielded an RD of 62 more per 1000 (95%CI: 3 more to 135 more) (5 RCTs, *n* = 485). There were no direct comparisons between ranges < 110 mg/dL and > 180 mg/dL, ranges of 110–144 mg/dL and 144–180 mg/dL, and ranges of 144–180 mg/dL and > 180 mg/dL.

The estimated values of hypoglycemia were as follows: when compared to a range < 110 mg/dL, 110–144 mg/dL yielded an RD of 13 more per 1000 (95%CI: 42 fewer to 103 more) (1 RCT, *n* = 90), 144–180 mg/dL yielded an RD of 63 fewer per 1000 (95%CI: 67 fewer to 58 fewer) (5 RCTs, *n* = 7331), and > 180 mg/dL yielded an RD of 85 fewer per 1000 (95%CI: 94 fewer to 75 fewer) (12 RCTs, *n* = 8342). When compared to a range of 110–144 mg/dL, 144–180 mg/dL yielded an RD of 66 fewer per 1000 (95%CI: 72 fewer to 58 fewer) (1 RCT, *n* = 302), and > 180 mg/dL yielded an RD of 88 fewer per 1000 (95%CI: 121 fewer to 37 fewer) (7 RCTs, *n* = 730). When compared to a range of 144–180 mg/dL, > 180 mg/dL yielded an RD of 0 per 1000 (95%CI: 0 to 0), due to an incidence rate of 0 in the control group) (1 RCT, *n* = 212). Therefore, we thought that a range of 144–180 mg/dL was superior to other target ranges.

**CQ14: Body temperature control**

**Introduction**

Body temperature is a vital sign that is measured on a daily basis, and fever or hypothermia triggers an evaluation of patient condition and change in treatment [748, 749]. As the body temperature varies by measurement site, it is necessary to obtain measurements in the most reliable sites as much as possible [748]. Abnormal body temperatures are often observed in patients with sepsis. Registry studies of sepsis patients in Japan reported that body temperatures at the time of ICU admission were as follows: less than 36 °C, 11.1%; 36–38 °C, 49.4%; and > 38 °C, 39.4% [750]. A multi-center prospective observational study conducted across 25 facilities in Japan and South Korea (the FACE study) reported that 40.5 and 11.5% of ICU patients experienced fever with temperatures over 38.5 °C and over 39.5 °C, respectively [751].

Body temperature is generally controlled in a narrow range of about 37 ± 0.5 °C by the hypothalamus, and fever is one of the adaptive reactions to infection and biological invasion [752]. Fever is a biological defensive response that triggers increased antibody production, T cell activation, cytokine synthesis, and neutrophil/macrophage activation. It has been repeatedly reported that fever was associated with a decreased mortality rate among patients with severe infection [753, 754]. Meanwhile, fever has negative aspects, such as patient discomfort, increased respiratory and myocardial oxygen demand, and central nervous system disorders [749].

Antipyretic therapy for patients with fever can be expected to decrease the pulse rate, respiratory rate, and oxygen consumption. It is also expected to relieve patient discomfort. Therefore, antipyretic therapy is generally provided to critically ill patients with fever. On the other hand, antipyretic therapy may suppress defensive responses that are beneficial to the body, and antipyretics have adverse effects such as gastrointestinal damage, liver and renal dysfunction, and hypotension [755, 756].

Antipyretic therapy can be classified as “drug-based antipyretic therapy” and “cooling-based antipyretic therapy” such as cooling on the surface of the body. Drug-based antipyretic therapy includes the use of non-steroidal anti-inflammatory drugs or acetaminophen. Cooling-based antipyretic therapy is subclassified into body-surface cooling and core-cooling techniques. Antipyretic therapy is considered to be an important issue among septic patients with fever.

Hypothermia among septic patients is thought to be caused by the loss of body temperature maintenance functions, and this is more likely to occur in patients with higher disease severity than those with fever. Hypothermia is defined as a temperature below 36 °C according to the definition of the Acute Physiology and Chronic Health Evaluation II score, sepsis, or infection-related ventilator-associated complications [20, 757, 758]. Analyses based on sepsis registries in Japan also showed that hypothermia with temperatures below 36 °C occurred among more than 10% of patients within 24 h of admission to the ICU, and the mortality rate of patients with hypothermia was high among those with sepsis [750, 759].

Hypothermia is associated with impaired protective ability against infection and also results in adverse effects such as bradycardia, decreased cardiac contractility, arrhythmia, and decreased ventilatory response. Furthermore, hypothermia with a core body temperature of less than 35 °C can induce decreased cardiac contractility, cardiac diastolic dysfunction, and coagulation abnormalities, and temperatures below 33 °C can decrease platelet function [760–764].

In this way, the prognosis of septic patients presenting with hypothermia is poor. Re-warming for septic patients with hypothermia may be considered as novel treatment. Therefore, whether to manage septic patients with hypothermia by re-warming are important issues.

**Fig. 13:**
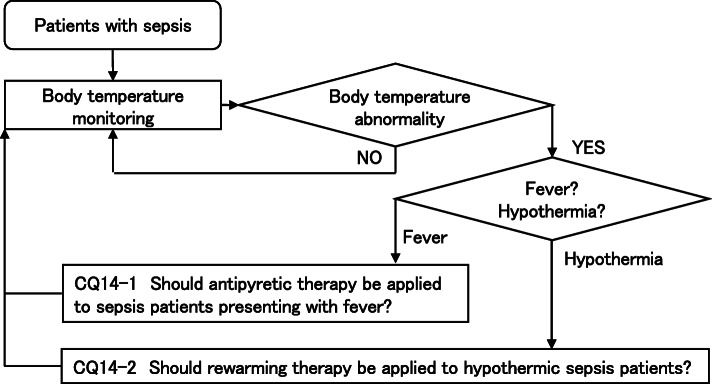
CQ14: Body temperature control (clinical flow)

Clinical flow of these CQs is shown in Fig. 13.

**CQ14-1: Should antipyretic therapy be applied to sepsis patients presenting with fever?**

***Answer:*** We suggest against conducting antipyretic therapy to sepsis patients presenting with fever (GRADE 2A: certainty of evidence = “high”).

***Rationale***

A meta-analysis evaluated 7 RCTs of patients who met the diagnostic criteria for sepsis [765–771]. We performed two types of analyses regarding mortality outcomes: one using all RCTs, and another which analyzed RCTs with a low risk of bias. We planned to use the analysis which only used RCTs with a low risk of bias for high certainty of evidence [765–769, 771].

The estimated value of effects for in-hospital mortality yielded a decrease of 14 fewer per 1000 (95%CI: 52 fewer to 30 more) (6 RCTs, *n* = 1439). That for the duration of treatment in the ICU yielded a MD of 0.26 days shorter (95%CI: 0.99 shorter to 0.46 longer) (2 RCTs, *n* = 889). Therefore, it was adjudged that the desired effect was trivial. The estimated value of effects for serious adverse effects yielded a RD of 13 fewer per 1000 (95%CI: 22 fewer to 7 more) (2 RCTs, *n* = 1144). Therefore, it was adjudged that the undesired effect was trivial. It was further adjudged that in the balance of effects, neither the intervention nor comparative control were superior to the other, regardless of the relative value setting for in-hospital mortality.

**CQ14-2: Should rewarming therapy be applied to hypothermic sepsis patients?**

***Answer:*** We suggest attempting to correct the body temperature of hypothermic (core body temperature < 35 °C) sepsis patients while considering hemodynamic stabilization when hemodynamic disorders and coagulation abnormalities related to hypothermia are observed (expert consensus: insufficient evidence).

***Rationale***

A literature review of 203 articles was performed using the search terms “re-warming”, “sepsis”, and “septic shock”. We confirmed that there were no RCTs on re-warming for adult patients with sepsis or septic shock with hypothermia.

Decreased cardiac contractility, cardiac diastolic dysfunction, and coagulation abnormalities can occur during hypothermia. It is highly likely that a slow re-warming attempt would be beneficial to patients when these abnormalities were thought to be due to hypothermia. The desired effects are thought to be small. However, it should be sufficiently noted that hemodynamic destabilization and relative decreases in circulating blood volume can occur during re-warming from a hypothermic state, and it was adjudged that the undesired effects were small.

The balance between the benefits and harms of re-warming therapy for septic patients with hypothermia is thought to vary according to the patient’s condition. The benefits of re-warming are thought to outweigh the harms when hypothermia is associated with circulatory insufficiency.

**CQ15: Diagnosis and treatment of disseminated intravascular coagulation in patients with sepsis**

**Introduction**

Changes in coagulation/fibrinolysis are observed even in the early phase of sepsis and worsen along with the condition. It is known that the mortality rate of patients with sepsis significantly increases when the disease is complicated by abnormalities of systemic coagulation such as DIC [772]. Since DIC is a state characterized by systemic hypercoagulation that induces microcirculatory disorders, it contributes to the development of organ dysfunction [773]. The fibrinolytic function is also activated in response to activation of coagulation in DIC; however, its extent varies according to the underlying disease. DIC is subclassified into the fibrinolysis-suppressing and fibrinolytic types. The fibrinolytic function is usually insufficient for activated coagulation in DIC caused by sepsis. The fibrinolysis-suppressing type of DIC due to sepsis often plays a role in the occurrence of organ dysfunction but presents a lower risk of bleeding that leads to poor prognoses [774].

The diagnosis of DIC in sepsis is essential to the assessment of the severity of sepsis and determining the timing of intervention. The “acute DIC diagnostic criteria” proposed by the Japanese Association for Acute Medicine are widely used in Japan1). In contrast, the “overt-DIC diagnostic criteria” proposed by the International Society on Thrombosis and Haemostasis are the international standard [775]. The acute DIC diagnostic criteria were specially designed for the diagnosis of acute DIC and have the advantages of simplicity and early diagnosis. The overt-DIC diagnostic criteria are designed to define DIC more strictly and therefore are more complicated. As a result, it has been indicated that the timing of diagnosis can be delayed [776, 777]. Inappropriate anticoagulation therapy is likely not only to be ineffective but also to increase the risk of adverse events. Thus, it is important to differentiate between patients with and without DIC [778].

It is necessary to monitor the states of coagulation/fibrinolysis in real-time and initiate anticoagulant therapy at the appropriate time according to the diagnosis of DIC. Since it is not possible to determine which diagnostic criteria are superior, it is important to choose proper diagnostic criteria for specific purposes, and we provide guidance on this in CQ15–1. When the diagnosis is made, we also recommend that other diseases that mimic DIC be differentiated based on CQ15–2. It is worth noting that in cases in which DIC diagnostic criteria are not satisfied, re-examination should be performed with the awareness that coagulation abnormalities are associated with outcome, and intensive care should be initiated so as not to delay treatment. Needless to say, in the management of DIC, it is essential to deal with the underlying causes. However, some patients may benefit from anticoagulation therapy. Evaluations based on evidence of representative therapeutic agents are presented in CQ15–3 through 6.

**Fig. 14:**
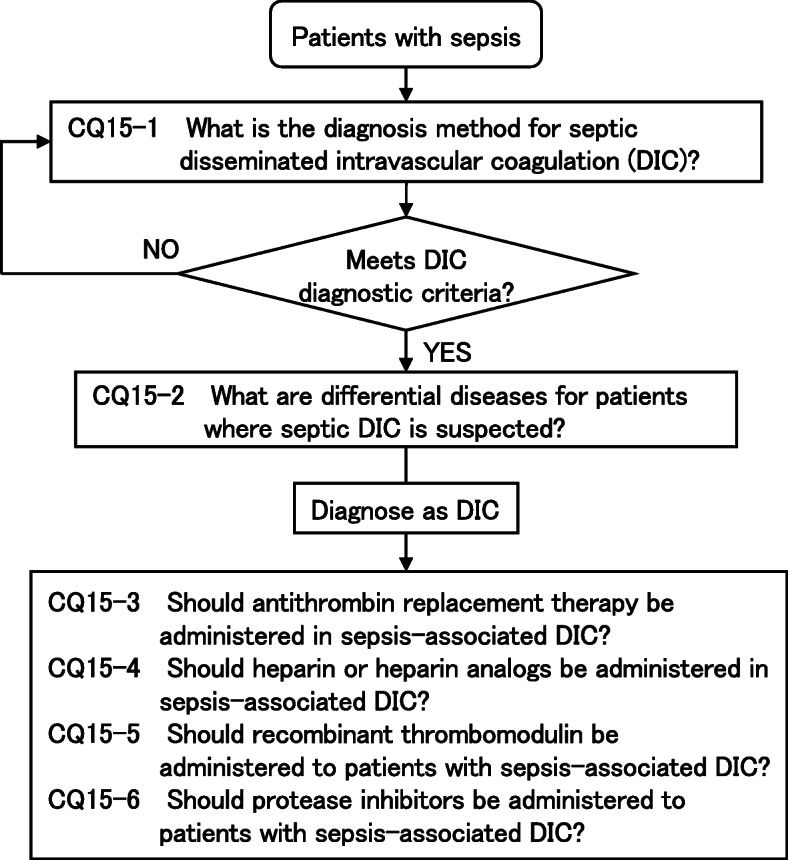
CQ15: Diagnosis and treatment of disseminated intravascular coagulation in patients with sepsis (clinical flow)

Clinical flow of these CQs is shown in Fig. 14.

**CQ15-1: What is the diagnosis method for septic disseminated intravascular coagulation (DIC)?**

***Answer:*** There are multiple diagnostic criteria for conducting DIC diagnosis. The acute DIC diagnostic criteria are widely used in Japan, while the ISTH overt-DIC is used as the international standard. It is difficult to determine the superiority between diagnostic criteria, and these should be used according to the purpose (Provision of information for background question).

***Rationale***

Coagulation/fibrinolysis disorders are present even in the early phase of sepsis due to perturbed interactions among the innate immune system, platelets, and the vascular endothelium. DIC refers to the systemic activation of coagulation, and if it is severe enough, it causes tissue malcirculation and organ dysfunction. Septic DIC has been recognized as one of the most critical conditions in sepsis due to its high frequency and severity. Two large-scale observational studies conducted in Japan reported that the mortality rate of patients with septic DIC was significantly higher than that of patients with sepsis [779, 780] Against this background, the diagnosis of DIC has been prioritized in the management of sepsis.

In recent years, multiple studies have reported that anticoagulant therapies could improve outcomes only among patients with DIC, but not among patients without DIC [778, 781]. Furthermore, large-scale observational studies conducted in Japan have shown that even the active screening and diagnosis of DIC in sepsis was associated with improved patient outcomes [782]. Based on these findings, the correct diagnosis of DIC in sepsis is suggested as a process that could improve outcomes by determining the appropriate timing for initiating interventions.

However, there is no consensus on which DIC diagnostic criteria should be used. The first DIC diagnostic criteria were established by the Ministry of Health, Labour and Welfare of Japan and published in 1979, followed by various criteria, including the overt-DIC diagnostic criteria released by the International Society on Thrombosis and Haemostasis (ISTH), the acute DIC diagnostic criteria put forth by the JAAM, and the Japanese Society on Thrombosis and Haemostasis DIC diagnostic criteria.

Among these, the JAAM acute DIC diagnostic criteria [772] and the ISTH overt-DIC diagnostic criteria [775] are the most widely used. The JAAM acute DIC diagnostic criteria include a systemic inflammatory response syndrome score and the reduction rate of platelet count over time as diagnostic factors in order to detect coagulation disorders with a high sensitivity. The acute DIC criteria are most frequently used in Japan, whereas overt-DIC criteria, which are more strictly designed to avoid overdiagnosis, are used as the international standard.

It is impossible to determine which criteria are superior because there is no gold standard for the diagnosis of DIC. To determine the superiority, studies that compare patients’ outcomes after the treatment following various DIC diagnostic criteria are necessary. However, this type of evidence cannot be achieved at present. The different characteristics are owing to the different objectives of each diagnostic criterion. As many clinicians consider septic DIC as a target for the anticoagulant therapies, and early initiation is more effective in Japan, they require an indicator that makes early stage treatment possible. In contrast, since clinicians in other countries do not consider septic DIC as a specific target for treatment, strict diagnostic criteria to accurately assess the pathophysiological conditions are more suitable. As such, it does not make sense to compare superiority or inferiority, and choosing the appropriate criteria with a sufficient understanding of their characteristics. For example, to avoid overdiagnosis, the overt-DIC criteria are the better choice. Conversely, the acute DIC diagnostic criteria are more suitable to avoid overlooking DIC.

The above-mentioned viewpoints regarding DIC diagnosis have been discussed by the working group for DIC in the guideline committee, and the details have been described in a review paper [776].

**CQ15-2: What are differential diseases for patients where septic DIC is suspected?**

***Answer:*** Thrombotic thrombocytopenic purpura (TTP), hemolytic uremic syndrome (HUS) and heparin-induced thrombocytopenia (HIT) are common DIC-like pathological conditions. These types of diseases require managements that are different from that of DIC (Provision of information for background question).

***Rationale***

DIC refers to a systematic activation of coagulation that arises from various underlying diseases. A survey conducted in Japan by the Japanese Association for Acute Medicine reported that the incidence rate of DIC is high and exceeded 50% among patients with sepsis [780]. Thrombotic microangiopathy (TMA) mimics DIC but should be differentiated since it can quickly lead to a life-threatening condition without adequate treatment. TMA is characterized by microangiopathic hemolytic anemia (MAHA), consumptive thrombocytopenia, and organ dysfunction due to microthrombosis. TMA includes HUS caused by Shiga toxin-producing *Escherichia coli* (STEC); TTP, which is caused by either congenital conditions (Upshaw–Schulman syndrome) or acquired autoantibody-induced ADAMTS13 (a disintegrin-like and metalloproteinase with thrombospondin type 1 motif 13), a depletion in the cleavage enzyme of the von Willebrand factor (vWF); atypical HUS (aHUS), due to the dysregulated activation of complements; and secondary TMA, due to other causes (e.g., autoimmune diseases, transplantation-related states, infection, drugs, etc.) [783]. The frequency of TMA occurrence has been reported to be approximately 1/150th that of DIC [784]. However, there is still the possibility of TMA or co-existence of TMA when patients show laboratory findings similar to those of DIC.

Various flow-charts have been proposed in recent years for the diagnosis of TMA [785–787]; however, many of these focus on the differentiation of DIC. The focus should rather be put on detecting unusual features of DIC at the initial stage in these differential diagnoses [785–787]. The diagnosis and treatment of septic DIC should be rapidly performed; however, it is important to look back at the diagnosis when the treatment response is poor or the clinical signs are atypical. In such a situation, the possibility of TMA should be kept in mind and the treatment must be promptly switched to the specific treatment for each disease (e.g., plasma exchange, molecular-targeted therapy, etc.) [788]. In addition, there is need for an early discrimination of HIT which often complicates thrombosis with thrombocytopenia. Clinically, screening for HIT can be made with 4Ts scoring [789], and more accurately with the detection of antibodies. Meanwhile, hemolysis, elevated liver enzymes, and low platelets (HELLP) syndrome [790] is a severe form of pregnancy-induced hypertensive syndrome that rapidly improves through delivery; thus, it can be relatively easily differentiated during clinical diagnosis. However, congenital TTP and aHUS can secondarily occur or coexist through the increase in the level of vWF during pregnancy and caution must be taken in such cases [791]. Therefore, the review paper published by the working group on DIC treatment from this guideline committee has also proposed a flowchart for the differential diagnoses of DIC in the early stage [788].

**CQ15-3: Should antithrombin replacement therapy be administered in sepsis-associated DIC?**

***Answer:*** We suggest antithrombin replacement therapy for patients with sepsis-associated DIC (GRADE 2C, certainty of evidence = “low”).

***Rationale***

Antithrombin has anticoagulant properties predominately manifested by inhibition of thrombin and activated factor X. Apart from its anticoagulant activities, antithrombin also possesses direct anti-inflammatory effects manifested by promotion of prostacyclin production in vascular endothelial cells [792]. Antithrombin is expected to potentially regulate the progression of DIC is widely used in Japan. However, previous studies have shown conflicting results regarding the beneficial effects of antithrombin on mortality among patients with sepsis, and no definitive evidence has been established.

We performed a systematic review and meta-analysis on 5 RCTs [793–797] that evaluated the efficacy of antithrombin administration in adult patients with DIC in sepsis and found that the effect of antithrombin administration on mortality showed a decrease of 134 deaths per 1000, whereas the adverse effect on hemorrhagic complications showed an increase of 9 events per 1000. The relative value of favorable effects (a reduced mortality rate) was generally higher than that of adverse effects (increased hemorrhagic complication). Therefore, we suggest that the benefits of antithrombin administration likely outweigh the harms.

**CQ15-4: Should heparin or heparin analogs be administered in sepsis-associated DIC?**

***Answer:*** We suggest against administering heparin or heparin analogs as a standard treatment for patients with sepsis-associated DIC (GRADE 2D, certainty of evidence = “very low”).

***Rationale***

Heparin is one of the oldest agents used in the treatment of DIC in sepsis in Japan. However, there is no established evidence confirming the survival benefit of heparin in sepsis. We performed a systematic review and meta-analysis of 2 RCTs that investigated the effects of heparin/heparinoid administration in adult patients with DIC in sepsis [798, 799].

The effect of heparin/heparinoid administration on mortality was a decrease of 58 deaths per 1000. Its effect on hemorrhagic complications was a decrease of 52 events per 1000. However, given that the number of studies included in the current meta-analysis and the sample sizes for all outcomes were small, it was judged that the certainty of the evidence was very low. Furthermore, the upper and lower limits of the confidence intervals were large, and the directionality of the effects was different. Thus, the superiority of either intervention or comparative controls could not be judged. Therefore, we recommend against the use of heparin/heparinoids as a standard treatment for DIC in sepsis.

**CQ15-5: Should recombinant thrombomodulin be administered to patients with sepsis-associated DIC?**

***Answer:*** We suggest administering recombinant thrombomodulin for patients with sepsis-associated DIC (GRADE 2C, certainty of evidence = “low”).

***Rationale***

Recombinant thrombomodulin binds to thrombin, promotes the activation of protein C, and exhibits anticoagulant effects by inhibiting further thrombin generation. In addition, it has been shown that its lectin-like domain has unique anti-inflammatory activity [800]. Recombinant thrombomodulin is therefore expected to be beneficial in the treatment of DIC in sepsis and is widely used in Japan.

We performed a systematic review and meta-analysis of 3 RCTs that investigated the effects of recombinant thrombomodulin administration in adult patients with DIC in sepsis [801–803]. In one of the eligible studies [801], we used the results of sub-group analysis that met the entry criteria at the time of drug administration. The effect of recombinant thrombomodulin therapy on mortality was 41 fewer deaths per 1000. Its effect on hemorrhagic complications was 12 more per 1000. The relative value of favorable effects (a reduced mortality rate) was generally higher than that of adverse effects (increased hemorrhagic complications). Therefore, we suggest that the benefits of recombinant thrombomodulin administration outweigh its harms.

**CQ15-6: Should protease inhibitors be administered to patients with sepsis-associated DIC?**

***Answer:*** We suggest against administering protease inhibitors as standard treatment for patients with sepsis-associated DIC (GRADE 2D, certainty of evidence = “very low”).

***Rationale***

Protease inhibitors suppress excessive coagulation activity in DIC. As they also inhibit fibrinolytic activity, protease inhibitors are considered to have a lower risk of hemorrhagic complications than other anticoagulant drugs. Protease inhibitors have been frequently used in Japan as a clinical therapeutic option for DIC due to various underlying diseases, such as sepsis. Although they play an important role in anticoagulant therapy for DIC, no studies have shown the beneficial effects of protease inhibitors on improvement of clinical outcomes.

We performed a systematic review and meta-analysis on 2 RCTs [804, 805] that investigated the effects of protease inhibitors in adult patients with DIC in sepsis. The effect of protease inhibitor administration on mortality outcomes was 39 fewer deaths per 1000. Its effect on hemorrhagic complication outcomes was 161 fewer per 1000. However, since the number of studies included in the current meta-analysis and the sample sizes for all outcomes were small, it was suggested that the certainty of the evidence was very low. Furthermore, the upper and lower limits of the confidence intervals were large, and the directionality of the effects was different. Thus, the superiority of either intervention or comparative controls could not be judged. Therefore, we recommend against the use of protease inhibitors as a standard treatment for DIC in sepsis.

**CQ16: Venous thromboembolism countermeasures**

**Introduction**

Venous thromboembolism (VTE) includes both deep vein thrombosis (DVT) and pulmonary embolism (PE). VTE is a pathological condition that requires care as it is a life-threatening complication that may occur during hospitalization. The “Guidelines for Diagnosis, Treatment, and Prevention of Pulmonary Thromboembolism and Deep Vein Thrombosis (2017 revised edition)” published in Japan presented the necessary prophylaxis according to the risk of VTE onset [806]. In this guideline, severe infections were listed as additional risk factors for VTE onset alongside the moderate risk factors of old age, long-term bed rest, cardiopulmonary disease and cancer-bearing status.

There are few studies on VTE among patients with severe infections or sepsis, and there has not been any highly reliable report apart from that published by Kaplan et al. adopted in the Japanese version of the Surviving Sepsis Campaign Guidelines 2016 [807]. A prospective trial of 113 patients hospitalized in the ICU due to sepsis or septic shock conducted by Kaplan et al. showed that the incidence rates of VTE and PE were high at 37.2 and 3.5%, respectively, although VTE prophylaxis was administered to all patients. The proportions of patients who required indwelling central venous catheters (OR 4.37) and mechanical ventilation (OR 2.35) were particularly high. A study of more than 3 million cancer patients conducted in the United States showed that the incidence of VTE increased as complications increased; however, the most influential complication was infection, including sepsis (sepsis 14%, invasive candidiasis 16%, pneumonia 11%, and indwelling venous catheter infection 14%) [808].

The risk of VTE increases among patients with infectious diseases in a hypercoagulable state due to inflammation. Therefore, a common consensus is to administer anticoagulation therapy and physical therapy to prevent VTE. However, there is still little research on the incidence rate of VTE among patients with sepsis associated with severe coagulopathy and DIC. There is also ongoing discussion about effective prophylaxis. Therefore, in this section, we formulated CQs on VTE measures among patients with sepsis.

**Fig. 15:**
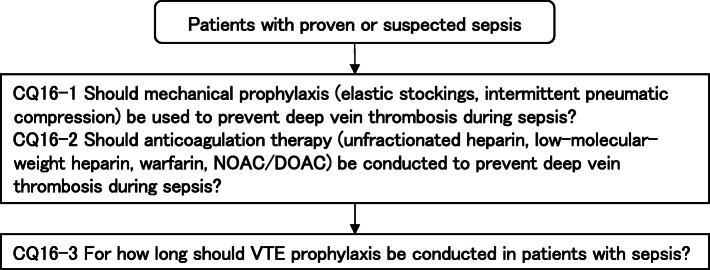
CQ16: Venous thromboembolism countermeasures (clinical flow)

Clinical flow of these CQs is shown in Fig. 15.

**CQ16-1: Should mechanical prophylaxis (elastic stockings, intermittent pneumatic compression) be used to prevent deep vein thrombosis during sepsis?**

***Answer:*** We suggest using mechanical prophylaxis (elastic stockings, intermittent pneumatic compression) to prevent deep vein thrombosis in patients with sepsis (expert consensus: insufficient evidence).

***Rationale***

Anticoagulation therapy and mechanical prophylaxis are recommended in the SSCG 2016. Furthermore, the J-SSCG 2016 suggest anticoagulation therapy and mechanical prophylaxis according to the risk level as “expert consensus: no evidence” [1–4]. However, these guidelines were derived from references which included various post-operative and critically ill patients who were hospitalized in the ICU. There is no evidence-based opinion on the effectiveness and harmfulness of each prophylaxis on sepsis patients. It is thought to be important to administer mechanical prophylaxis (elastic stocking and intermittent air compression) to prevent VTE, and the analyses were limited to only septic patients.

A systematic review found no RCTs on this subject. Systematic reviews on critically ill patients in the ICU or RCTs of injured patients reported that mechanical prophylaxis was non-inferior to low-molecular-weight heparin [809, 810]. RCTs on critically ill patients with a risk of hemorrhage and RCTs of concomitant anticoagulation therapy among critically ill patients reported that intermittent air compression was ineffective [811, 812].

It has been reported that the risk of VTE onset was high in septic patients. We suggest using mechanical compression to prevent deep vein thrombosis as mechanical prophylaxis may prevent lethal complications such as pulmonary embolism. Care should be taken during implementation since blood flow disorders may occur in patients with skin injuries due to mechanical compression, diabetes, or obstructive arteriosclerosis.

**CQ16-2: Should anticoagulation therapy (unfractionated heparin, low-molecular-weight heparin, warfarin, NOAC/DOAC) be conducted to prevent deep vein thrombosis during sepsis?**

***Answer:*** We suggest conducting anticoagulation therapy to prevent deep vein thrombosis in patients with sepsis (expert consensus: insufficient evidence).

***Rationale***

RCTs and meta-analyses of critically ill patients in the ICU reported that the incidence rate of VTE among patients receiving VTE prophylaxis due to either low-molecular weight heparin (LMWH), unfractionated heparin (UFH), or fondaparinux decreased by approximately 40–60% [813, 814]. However, the incidence rate of VTE could vary widely from approximately 22–80% according to the patient’s illness and pathological state, and careful interpretations must be made to evaluate whether the results could be generalized to sepsis [815]. The “Guidelines for Diagnosis, Treatment, and Prevention of Pulmonary Thromboembolism and Deep Vein Thrombosis (2017 revised edition)” published in Japan described the risk classifications for DVT and its corresponding prophylaxis [806]. However, none of them contained evidence for sepsis patients, and caution is required in interpreting it. Administering anticoagulation therapy as VTE prophylaxis was thought to be an important clinical issue, and the analyses were limited to sepsis patients.

A systematic review was performed, but yielded no RCTs. The risk of VTE onset was high among sepsis patients, and anticoagulation therapy may be able to prevent lethal complications like PE. The risks of hemorrhage due to anticoagulation therapy are present, as is the risk of HIT when heparin is administered. However, many reports showed no significant increases in the incidence of hemorrhage, and very few cases were serious when this was present. Based on the above, we suggest that anticoagulation therapy should be administered as VTE prophylaxis after adjudging that the benefits of VTE prophylaxis due to anticoagulation therapy outweigh its harms.

Caution is required in its use due to the risk of hemorrhage from anticoagulation therapy and the risk of HIT onset during heparin use.

**CQ16-3: For how long should VTE prophylaxis be conducted in patients with sepsis?**

***Answer:*** We suggest conducting venous thromboembolism (VTE) prophylaxis in patients with sepsis until they are able to walk or discharged from the hospital (expert consensus: insufficient evidence).

***Rationale***

VTE prophylaxis via mechanical compression and anticoagulation therapy are recommended in the SSCG 2016 and the J-SSCG 2016. However, there is no evidence-based interpretation of the period during which each mode of prophylaxis should be administered to sepsis patients [1–4]. Mechanical prophylaxis as a mode of VTE prophylaxis leads to an increased risk of inducing blood flow disorders in the compressed area. Furthermore, anticoagulation therapy has the risk of inducing hemorrhaging complications. Based on these facts, it is thought that VTE prophylaxis should not be administered indiscriminately. However, the optimal period of VTE prophylaxis administration to sepsis patients has not been established, and decisions of the suspension period varies by facility or attending physician even in clinical practice. Based on the above, the CQ regarding how long to administer VTE prophylaxis to sepsis patients was thought to be highly important.

We performed a systematic review but found no relevant RCTs. If we used mechanical prophylaxis or anticoagulation therapy for preventing VTE during periods that patients were not able to be mobilized and deceased it when patients started to be mobilized, the risks of blood flow disorders due to mechanical prophylaxis or hemorrhaging complications due to anticoagulation therapy might be minimized. Meanwhile, VTE could occur after the patient leaves the bed or is discharged from the hospital, and could lead to lethal complications such as PE. We suggest that mechanical compression or anticoagulation therapy should be administered until the patient is capable of walking or is discharged from the hospital in terms of the balance of the preventative effects against VTE and the risks of complications.

The risk of VTE is high in practice even after the patient gets out of bed or is discharged from the hospital (e.g., patients who are not able to walk independently, or transfer of mechanically ventilated patients for their rehabilitation) and extended prophylaxis may be necessary.

**CQ17: ICU-acquired weakness and early rehabilitation**

**Introduction**

In 2010, the Society of Critical Care Medicine proposed the concepts of PICS and ICU-AW, while the physical and psychological problems that present in the subacute and chronic phases following discharge from the ICU have been gaining increasing attention [816]. PICS refers to the physical, cognitive, and mental impairments that occur during or after admission to the ICU and after discharge from the hospital. ICU-AW, which is the physical component of PICS, is a syndrome that presents with acute symmetric limb weakness that develops after admission to the ICU. Both PICS and ICU-AW are widely being recognized as affecting not only the long-term prognosis of ICU patients but also the mental states of their families. There have been various recent reports on PICS and ICU-AW [817, 818], and this chapter set the three interventions of early rehabilitation, passive joint exercise therapy, and neuromuscular electrical stimulation therapy as CQs and investigated their effectiveness through a meta-analysis. Understanding PICS and ICU-AW and their interventions should have the objective of rehabilitation, which goes beyond saving the lives of patients receiving intensive care, and collaboration with healthcare professionals not involved in intensive care is also necessary. Both are attracting attention as new issues in the field of intensive care, and it is important to share the latest knowledge on the prevention and treatment at the onset.

**Fig. 16:**
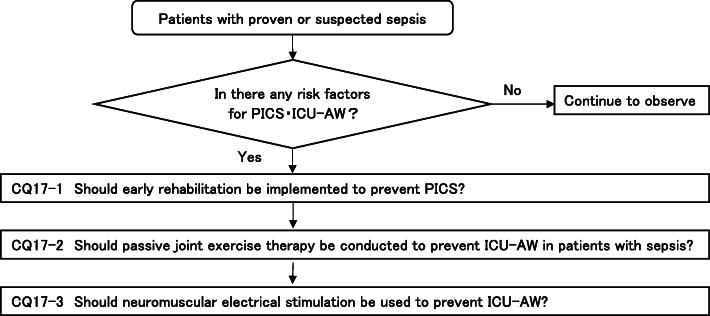
CQ17: ICU-acquired weakness and early rehabilitation (clinical flow)

Clinical flow of these CQs is shown in Fig. 16.

**CQ17-1: Should early rehabilitation be implemented to prevent PICS?**

***Answer:*** We suggest conducting early rehabilitation to prevent PICS in patients with sepsis (GRADE 2D, certainty of evidence = “very low”).

***Rationale***

Early rehabilitation of ICU patients is thought to prevent PICS by increasing muscle mass, improving physical function, encouraging patients to get out of bed early, and improving activities of daily living (ADL). However, the evaluation of the effectiveness and safety of early rehabilitation in sepsis patients has not been determined, and there are various definitions, types, start times, and implementation periods for early rehabilitation, even in clinical practice. In this CQ, we defined early rehabilitation as the following items (1)–(4) and investigated the preventive effects on PICS.

1. Physical therapy and/or occupational therapy (excluding cognitive therapy)
2. Includes rehabilitation outside the bed
3. Starts earlier than in the control group
4. Starts within 1 week of admission to the ICU

The results of a meta-analysis showed that the estimated value of the effects of in-hospital stay (10 RCTs, *n* = 1224) was 2.86 days shorter (95%CI: 5.51 shorter to 0.21 shorter), that of 36-item short-form health survey physical functioning scale score at 6 months (3 RCTs, *n* = 241) was 4.65 higher (95%CI: 16.13 lower to 25.43 higher), that of in-hospital medical research council (MRC) score (3 RCTs, *n* = 196) was 4.84 higher (95%CI: 0.36 higher to 9.31 higher), that of hospital anxiety and depression scale score at 6 months (1 RCT, *n* = 37) was 0.3 higher (95%CI: 4.92 lower to 5.52 higher), that of the mini mental state examination score at 6 months (1 RCT, *n* = 165) was 0.6 higher (95%CI: 0.25 lower to 1.45 higher), and that of in-hospital mortality (7 RCTs, *n* = 924) was 15 more per 1000 (95%CI: 24 fewer to 71 more). It was judged from these results that the desired effects were small. The estimated value of effects for the onset of adverse events (5 RCTs, *n* = 706) was 14 fewer per 1000 (95%CI: 38 fewer to 55 more). Therefore, it was judged that undesired effects were trivial. Based on the above, it was judged that the intervention was likely superior.

**CQ17-2: Should passive joint exercise therapy be conducted to prevent ICU-AW in patients with sepsis?**

***Answer:*** We suggest conducting passive mobilization as standard treatment for patients with sepsis (GRADE 2D: certainty of evidence = “very low”).

***Rationale***

The onset of ICU-AW is correlated with poor prognosis in patients. Rehabilitation intervention is started at an early stage to prevent the onset of ICU-AW. However, it is difficult to introduce active exercise therapy at an early stage in critically ill patients with sepsis, and passive joint exercise therapy is often the main treatment. Therefore, clarifying the effectiveness of passive joint exercise therapy in the prevention of the onset of ICU-AW in patients with sepsis is important in terms of considering rehabilitation intervention plans; thus, a meta-analysis was performed.

The estimated value of effects for MRC score yielded a MD of 0.96 lower (95%CI: 4.13 lower to 2.21 higher) (3 RCTs, *n* = 366), that for 6-min walk distance (6MWD) yielded an MD of 10.5 m higher (95%CI: 63.45 lower to 84.46 higher) (2 RCTs, *n* = 173), that for functional independence measure (FIM) yielded an MD of 3.00 higher (95%CI: 5.42 lower to 11.42 higher) (1 RCT, *n* = 115), that for the length of stay in the ICU yielded an MD of 0.36 days longer (95%CI: 1.79 shorter to 2.51 longer) (4 RCTs, *n* = 277), that for the length of stay in hospital yielded an MD of 0.74 days longer (95%CI: 3.68 shorter to 5.15 longer) (4 RCTs, *n* = 277), and that for the duration of mechanical ventilation yielded an MD of 0.14 days longer (95%CI: 1.03 days shorter to 1.31 longer) (4 RCTs, *n* = 531). Therefore, it was judged that the desired effects were small.

The estimated value of effects for various adverse events yielded a RD of 18 fewer per 1000 (95%CI: 42 fewer to 38 more) (3 RCTs, *n* = 416). The undesired effects were judged to be trivial.

Based on the above, it was judged that the intervention was likely superior.

**CQ17-3: Should neuromuscular electrical stimulation be used to prevent ICU-AW?**

***Answer:*** We suggest against using neuromuscular electrical stimulation as a standard treatment to prevent ICU-AW in patients with sepsis (GRADE 2D: certainty of evidence = “very low”).

***Rationale***

Neuromuscular electrical stimulation is expected to be effective in preventing muscle weakness in critically ill patients. It has been reported that effective muscle contraction is difficult to achieve in patients with sepsis, those who use pressor agents, and those with edema [819], and the effectiveness of neuromuscular electrical stimulation in sepsis patients is unclear. The J-SSCG 2016 recommended against neuromuscular electrical stimulation as ICU-AW prophylaxis for patients with sepsis or those in intensive care [3, 4]. Based on subsequent findings, this CQ investigated the preventive effects of ICU-AW onset with neuromuscular electrical stimulation.

The results of a meta-analysis showed that the estimated value of effects for the onset of ICU-AW at the time of discharge from the ICU (1 RCT, *n* = 28) was 0 per 1000 (95%CI: 183 fewer to 665 more). The MRC at the time of discharge from the ICU (1 RCT, *n* = 28) yielded a MD of 1.00 higher (95%CI: 4.19 lower to 6.19 higher), the number of days of mechanical ventilation (7 RCTs, *n* = 262) yielded a MD of 1.56 days shorter (95%CI: 3.12 shorter to 0.01 longer), in-hospital mortality (5 RCTs, *n* = 251) yielded a MD of 39 fewer per 1000 (95%CI: 174 fewer to 219 more), and length of stay in the ICU (5 RCTs, *n* = 212) yielded a MD of 3.23 days longer (95%CI: 3.35 shorter to 9.81 longer). Therefore, it was judged that the desired effects were trivial.

Various adverse events (pain, discomfort, and pad allergies) were set as an outcome; however, no descriptions were provided in the article results. Thus, an evaluation was not possible, and the undesired effects were unclear. A “neuromuscular electrical stimulator” was needed for intervention, and therefore, administering this at a facility that does not have this device requires its purchase. Therefore, its feasibility was judged to be “likely not”. Based on the above, it was judged that it was desirable not to administer neuromuscular electrical stimulation as a standard therapy of ICU-AW prophylaxis in all critically ill patients.

**CQ18: Pediatric considerations**

**Introduction**

Pediatric sepsis is a serious pathological condition that kills 10–20% of patients, with an even higher mortality rate among patients with septic shock [820, 821]. The J-SSCG 2016 [3, 4] proposed 15 CQs on pediatric sepsis; however, post-publication surveys of usage reported that the compliance rate with the recommendations/suggestions relating to children was only less than 5% [822]. Therefore, we started out to work in this amendment with the clear objective of creating a “guideline that people would use.”

First, in anticipation that the definitions of pediatric sepsis would change according to Sepsis-3 [19] in the near future, we decided not to propose CQs here relating to its definitions that were actively taken up in the J-SSCG 2016. Next, we did not comprehensively address all questions relating to pediatric sepsis management, but instead focused on items regarding decisions that would be difficult to make in clinical settings. Furthermore, as was the case in the previous guideline, issues in pre-term babies or in the transition period immediately following birth, which are areas of neonatology, were not included in the scope of this guideline.

A total of 14 CQs were initially proposed. Among these, the CQ relating to the management policy of sepsis refractory to fluid resuscitation was recognized by the committee as common to both adults and children, and a recommendation was made as a Good Practice Statement (see CQ21–3). As a result, discussions proceeded with the remaining 13 CQs in the pediatric working group, and we provided information on five of these as background questions (empiric antibacterial drugs, anti-herpes virus drugs, blood pressure management targets, methods of evaluating response to fluid resuscitation, and the appropriate rate and amount of fluid resuscitation). A recommendation was also made for one CQ as an expert consensus since no appropriate RCTs could be obtained through systematic review (intravenous immunoglobulin).

Recommendations were made according to the results of a systematic review based on the GRADE methodology for the remaining seven CQs (application of practice algorithms, first-line inotropic/vasoactive agents, vasopressin, systemic steroids, erythrocyte transfusion, acute blood purification therapy, and tight glycemic control). Although there was still very little evidence specific to children during this process, we also found new RCTs being conducted for some of these questions [823–827]. However, there were also many questions for which no new research had been conducted so far, and recommendations were carefully examined for those questions while considering the trends in evidence seen in the adult domain.

Finally, we discuss the future prospects of pediatric sepsis research. Many recent large-scale RCTs on pediatric sepsis have been published in developing and emerging countries [828]. Community-acquired infectious diseases and sepsis are still recognized as central issues of healthcare in these regions, and the ease of patient recruitment is also considered one of these factors. However, careful scrutiny is required when extrapolating these research results to medical environments in developed countries due to the indirect nature of the work. Furthermore, it is desirable to accumulate knowledge on long-term survival and functional prognosis in addition to short-term survival as an outcome indicator precisely because our patients are children with a long life ahead.

**Fig. 17:**
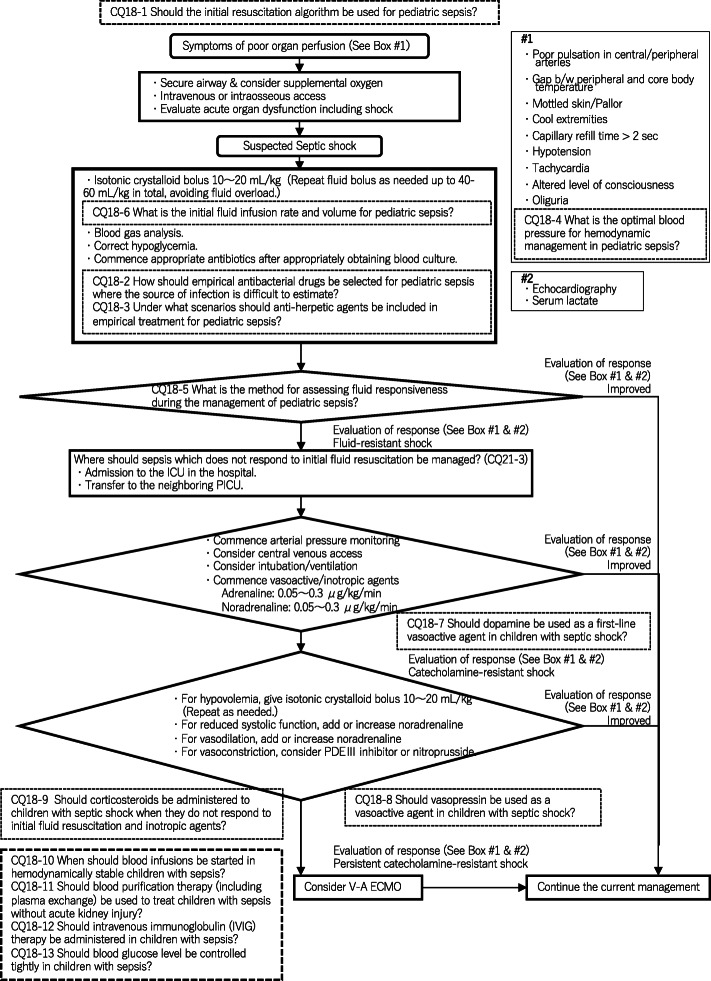
CQ18: Management algorithm for pediatric septic shock (clinical flow)

Clinical flow of these CQs is shown in Fig. 17.

**CQ18-1: Should the initial resuscitation algorithm be used for pediatric sepsis?**

***Answer:*** We suggest using the initial resuscitation algorithm for pediatric sepsis (GRADE 2D: certainty of evidence = “very low”).

***Rationale***

Clinical algorithms such as the American College of Critical Care Medicine–Pediatric Advanced Life Support (ACCM–PALS) [829] have been used to perform evaluations and interventions of children with septic shock via a systematic approach and for recovery from shock as quickly as possible. However, its validity and reliability need to be verified.

As there were no RCTs on this CQ, one observational trial was used [830], and the biases with effects were evaluated according to the ROBINS-I tool. The observational trial used in this CQ considered the ACCM-PALS algorithm [829] as an intervention in a cohort comparison. The estimated effect for mortality (1 observational trial, *n* = 91) yielded a RD of 303 fewer per 1000 (95%CI: 357 fewer to 107 fewer); thus, the desirable effects were deemed large. No piece of literature has investigated the time to withdrawal from shock. We did not plan in advance the evaluation of the harmful outcomes of using clinical algorithms. There was a concern of fluid overload as a result of initial resuscitation using the algorithm. However, we believe that these effects would be reflected in increased mortality rates; therefore, we did not consider other harmful outcomes as critical. Considering the large desirable effects, it is likely to be valid to estimate that the intervention is superior.

Points of consideration related to implementation include the early recognition and handling of fluid overload. Initial resuscitation of children with sepsis requires diligent evaluation of peripheral circulatory insufficiency and improvement of organ perfusion as well as findings of fluid overload such as coarse crackles, increased work of breathing, and hepatomegaly [831]. Prompt suspension of fluid resuscitation or slowing of fluid administration should be considered as soon as fluid overload is suspected.

**CQ18-2: How should empirical antibacterial drugs be selected for pediatric sepsis where the source of infection is difficult to estimate?**

***Answer:*** Antibacterial drugs which cover the possible microorganisms should be selected with consideration of the site of occurrence (e.g., community, hospital, ICU) and patient background (e.g., immune status, treatment history) (see Table 14 for reference) (Provision of information for background question).

**Table 14:** CQ18–2: How should empirical antibacterial drugs be selected for pediatric sepsis when the source of infection is difficult to identify?

	Inferred microorganisms	Notes
**Community-acquired** Cefotaxime (ceftriaxone) [Less than 1 month old with high possibility of meningitis] Add ampicillin in consideration of *Listeria monocytogenes* [More than 1 month old with high possibility of meningitis] Add vancomycin [High risk of ESBL-producing bacteria] Switch to meropenem	*Streptococcus pneumoniae*, *Haemophilus influenzae*, *Staphylococcus aureus*, *E. coli*, etc.	Consider underlying diseases, immune function, history of local endemics, etc.
**Hospital-acquired** Cefotaxime (ceftriaxone) or cefepime or piperacillin tazobactam or meropenem (+vancomycin) (+antifungal drugs)	*Enterobacteriaceae*, non-glucose fermenting bacteria such as *Pseudomonas aeruginosa*, *Staphylococcus aureus* including *MRSA*, fungi, etc.	Consider underlying diseases, treatment history, immune function, previous detection of resistant bacteria, in-hospital antibiograms, etc. Add vancomycin or antifungal drugs according to risk
Dosage Cefotaxime	200 mg/kg/day, every 6 h (meningitis; 300 mg/kg/day, every 6 h) maximum of 12 g/day	
Ampicillin	200 mg/kg/day, every 6 h (meningitis; 400 mg/kg/day, every 6 h) maximum of 12 g/day	
Cefepime	150 mg/kg/day, every 8 h	maximum of 6 g/day)
Piperacillin tazobactam	337.5 mg/kg/day, every 8 h	maximum of 18 g/day
Meropenem	120 mg/kg/day, every 8 h	maximum of 6 g/day
Vancomycin	60 mg/kg/day, every 6 h	

***Rationale***

The selection of antibiotics is determined by considering the patient’s age, site of infection, background, and estimated organ transferability [832]. The site of infection is an important element when considering the causative microorganism. Pediatric community-acquired bacterial infections are frequently caused by *Streptococcus pneumoniae, Haemophilus influenzae*, *Staphylococcus aureus*, and Enterobacteriaceae represented by *Escherichia coli*. These bacteria are usually sensitive to cefotaxime, which is a third-generation cephalosporin. However, *Listeria* has a relatively high frequency of involvement among children younger than 1 month with sepsis [833], the addition of ampicillin should be considered. Cephalosporin- and carbapenem-resistant strains of *Streptococcus pneumoniae* should be considered when the possibility of meningitis is high in children 1 month after birth [834, 835], and the possibility of adding vancomycin should be assessed [836]. Finally, patient background such as underlying illness, immune states such as primary immunodeficiency and asplenia, and the surrounding epidemic history should be considered when selecting antibiotics.

In recent years, the prevalence of extended spectrum β-lactamases (ESBL) producing bacteria among the Enterobacteriaceae has been increasing [837]. The choice of carbapenems in the treatment of infections caused by ESBL-producing bacteria needs to be considered when initiating treatment for sepsis in which Enterobacteriaceae are thought to be causative microorganisms, such as pyelonephritis, intra-abdominal infections, or meningitis in neonates, and when there is a high risk of drug-resistant bacteria, such as patients with a history of prior antimicrobial administration or medical exposure [838, 839].

Antibiotics for the treatment of pediatric sepsis in general wards or the ICU should be selected in a similar process. In addition to Enterobacteriaceae, non-fermenting bacteria such as *Pseudomonas aeruginosa* and *Acinetobacter* can also be causative microorganisms [840], antimicrobial agents should be selected based on risk and severity. The same is true for the choice of antibiotics for the treatment of MRSA and fungal infections (see CQ4–3). A past history of drug-resistant bacterial detection in the patient and exposure to antibacterial drugs would increase the possibility of drug-resistant bacteria or fungi being identified as causative microorganisms [841]. The sensitivity of microorganisms to each drug varies by facility; therefore, antibiograms in the hospital should be referenced when selecting antibacterial drugs.

**CQ18-3: Under what scenarios should anti-herpetic agents be included in empirical treatment for pediatric sepsis?**

***Answer:*** There are cases where a central nervous system infection is suspected or a bacterial source of infection cannot be specified in neonates, because the prevalence of the herpes simplex virus is higher and they can easily become severe once infected (Provision of information for background question).

***Rationale***

Children are more likely to have sepsis due to a virus infection than adults; among these and treatable viruses include the HSV. Delayed treatment has been reported to result in an increased mortality rate and severe sequelae [842, 843]. Sepsis due to HSV has non-specific clinical symptoms, and it is difficult to determine whether the pathogen is HSV based on clinical images or rapid testing. Therefore, the initiation of administration of anti-herpetic drugs should be considered before a definitive diagnosis is established.

Meanwhile, the excessive use of anti-herpetic drugs has been reported to be increasing among children older than 30 days [844], and there are concerns about these drugs due to their adverse effects or costs, and the fact that HSV-induced sepsis is not a particularly high-frequency event. Large-scale observational studies conducted in North America showed that, among 26,533 patients younger than 60 days who visited the ER (no record of the number of sepsis patients), those with HSV infection remained at 112 (0.42%), of which 36 patients (0.14%; 95%CI: 0.10 to 0.19%) had the central nervous system type and 32 patients (0.12%; 95%CI: 0.08 to 0.17%) had the systemic type [845]. In other words, the incidence is extremely low, and it can be said that the proportion of patients for whom favorable effects would be achieved with anti-herpetic drug administration as an empiric treatment is extremely limited. In reality, in the studies mentioned above, anti-herpetic drugs should have been administered as empiric treatment among 588 patients (95%CI: 435 to 769) in order to treat one patient each younger than 60 days with the central nervous system- and systemic-type of HSV infection. The median age of patients with HSV infection was 14 days (interquartile range [IQR] 9 to 24), and the contraction frequency was higher among patients aged 0–28 days than among those aged 29–60 days (odds ratio 3.9; 95%CI: 2.4 to 6.2). This would mean that 152 (95%CI: 123 to 185) and 583 (95%CI: 384 to 909) patients, respectively, would have started receiving anti-herpetic drugs as empiric treatment to treat a single patient each with HSV infection aged 0–28 days and 29–60 days [845]. Therefore, the favorable effects of empirically administering anti-herpetic drugs would be expected more in children aged 0–28 days.

Serious adverse effects such as renal dysfunction [846], cytopenia, and neuropsychiatric symptoms can occur when using anti-herpetic drugs. The risk of tissue damage due to extravasation should also not be ignored in infants with thin blood vessels. Furthermore, there may be an increase in fluid load due to the large volume of water required to dilute the anti-herpetic drug. The confirmation of HSV infection using methods such as polymerase chain reaction assays takes several days at most facilities; therefore, there is a risk of extending the length of hospitalization until empiric treatment with an anti-herpetic drug has been completed [847].

At present, no RCTs have investigated whether anti-herpetic drugs should be included as empiric treatment among pediatric patients with sepsis. However, as mentioned above, it is thought that an increased proportion of patients older than 29 days is negatively affected. Furthermore, it is inappropriate to initiate anti-herpetic drugs as empiric treatment in children with sepsis, among whom the source of infection can be clearly estimated (e.g., those with pneumonia and urinary tract infection). As such, it is advisable that anti-herpetic drugs should be included as empiric treatment in patients younger than 1 month who are likely to have central nervous system infections or who have sepsis with no presumed site of infection.

Needless to say, patients with confirmed HSV infection, regardless of age group, should be treated promptly with anti-herpetic drugs [842, 843].

**CQ18-4: What is the optimal blood pressure for hemodynamic management in pediatric sepsis?**

***Answer:*** Suitable values for the optimal blood pressure are unknown, and this should be set with consideration to age and organ perfusion. The median value for the mean blood pressure “55 + age x 1.5 mmHg” and the 5th percentile value “40 + age x 1.5 mmHg” in healthy children are used as a reference (Provision of information for background question).

***Rationale***

Blood pressure is commonly used in the management of sepsis as an assessment indicator when making decisions in evaluating treatment effects or changing the course of treatment. Hypotension has been identified as a sign of decreased tissue perfusion in the management of children with sepsis [3, 4, 848]. However, the optimal blood pressure largely depends on the age and body weight. Furthermore, we need to take into account the general conditions and organ damage among patients, and the tissue perfusion pressure in response to these, which makes it difficult to discuss them uniformly. We believe that it should be meaningful to understand the background of the reference values and to keep the evidence organized.

It is desirable to tailor the targets of mean blood pressure considering the necessary organ perfusion in each case; however, the relative merit of the management based on the systolic blood pressure remains unclear. There are no existing references on numerical targets, and a consensus could not be reached among the experts involved in preparing this guideline. A study of a large sample was conducted in the United States on the normal range of blood pressure in healthy children [849]. Indices based on the age range for systolic and diastolic blood pressure as well as mean blood pressure are presented, which can be used as a reference when setting targets and acceptable lower limits of blood pressure. However, it should be noted that target blood pressures need to be set considering the individual pathology and the corresponding required organ perfusion.

**CQ18-5: What is the method for assessing fluid responsiveness during the management of pediatric sepsis?**

***Answer:*** Assessments for fluid responsiveness include clinical findings (changes in pulse rate, blood pressure, temperature difference between peripheral and central skins, strength of pulsation, and capillary refill time (CRT)) and test values (e.g., lactate clearance, echocardiography findings) (Provision of information for background question).

***Rationale***

Similar to those in adults, proper systemic management and infectious disease treatment are two essential elements of sepsis treatment in children, and adequate preloading during initial management is the basis for the process of increasing cardiac output and stabilizing the hemodynamics [3, 4, 848]. However, it is not easy to assess whether preloading is appropriate, and excess fluid has been indicated to potentially prevent the recovery of organ function [850].

Methods of assessment of responsiveness to fluid resuscitation include 1) indicators for predicting in advance whether the cardiac output increases when fluid resuscitation is implemented and 2) indicators for assessing after the fact that cardiac output increased after administering fluid resuscitation.

This guideline uses the term “fluid responsiveness prediction” for 1); however, at present, a sufficiently reliable predictive indicator of fluid responsiveness does not exist in the field of pediatrics [851]. A systematic review performed by Gan et al. among critically ill children with various backgrounds showed that there was no reliable static indicator, and the respiratory variation in aortic blood flow peak velocity (ΔV_peak_) measured via Doppler echocardiography was the only reliable dynamic indicator [851]. However, although a recent systematic review and meta-analysis performed by Desgranges et al. among children in the ICU and operating room confirmed these findings, the authors indicated that the cut-off value introduced by different studies ranged from 7 to 20%, and that it was premature to apply these results in clinical decision making [852]. It should be noted that the reliability of SVV, PPV and ultrasonographic assessments of the inferior vena cava diameter, whose effectiveness as predictive indicators for fluid responsiveness among adults has been established, has not been verified in multiple studies of children [851]. Although PLR has been suggested to be effective, there has only been one report on this so far [853]. Furthermore, none of the studies incorporated into these systematic reviews were specific to sepsis.

Meanwhile, it is desirable to use 2) during the initial fluid resuscitation process to re-assess effects by combining multiple indicators each time a bolus of 10–20 mL/kg of isotonic crystalloid fluid is administered. Unexpected fluid overload can occur if increases in cardiac output due to fluid are not periodically re-assessed and fluid administration is continued as before. Clinical findings such as the correction of tachycardia or hypotension, improvements in the pulsation, and reductions in peripheral/central system temperature differences suggest an increase in the stroke volume and cardiac output. It is also important to assess for improvements in findings such as altered states of consciousness or oliguria caused by organ hypoperfusion [848].

The capillary refill time (CRT) is a clinical sign in which the peripheral circulation is assessed by measuring how many seconds it takes for improvements in skin color to occur immediately after relieving pressure following pressure ischemia of the skin on the fingertips/toes or trunk. Values exceeding 2 s typically suggest decreased skin perfusion, and are suggestive of impaired peripheral circulation [854, 855]. CRT assessment is non-invasive and is widely used as an indicator of circulatory management that can be repeatedly measured [848]. Reports have indicated that a CRT ≤ 2 s in children admitted to the pediatric ICU was correlated with ScvO_2_ ≥ 70% [856], and that there was a correlation between a CRT > 3 s and mortality [857]. Meanwhile, the CRT is known to be influenced by a variety of factors including patient age, assessment location, pressure time, ambient temperature, and skin temperature [854], and care must be taken to ensure that assessment methods are consistent, such as using a stopwatch [855]. For some indicators, consistency between evaluators was determined to be low [854], and its correlation with invasive hemodynamic indicators such as cardiac index was low [858, 859]. Thus, assessing hemodynamics only with the CRT should be avoided.

Increased lactate levels primarily reflect tissue hypoxia, and have been used to define adult septic shock in Sepsis-3 [19]. Multiple observational studies in the field of pediatrics have also indicated that hyperlactemia at the time of diagnosis was correlated with an increased mortality rate [860–862], that the lack of decreases in lactate level with fluid- or cardiovascular agent-based interventions was correlated with mortality [863, 864], and that normalized lactate levels were correlated with recovery of organ function [865]. Meanwhile, it was indicated that cases of pediatric septic shock diagnosed based on clinical findings did not always present with hyperlactemia, regardless of whether the shock pattern was compensatory or non-compensatory (i.e., hypotensive) [848]. As such, decreases in lactate levels due to fluid resuscitation can be used as an assessment indicator for determining effectiveness only in patients whose lactate levels elevated on presentation. However, the cut-off value for lactate clearance that can be deemed effective is not clear, and this needs to be determined with other hemodynamic indicators, similar to that of the CRT. It should be noted that a recent RCT that evaluated hemodynamic management with CRT normalization compared with that of lactate clearance in septic shock among adults showed that the former was not superior to the latter in terms of 28-day mortality [319].

Echocardiography can be used to perform repeated non-invasive assessments at the bedside, and does not only provide objective information for determining the preload and contractility, but can also confirm congenital heart diseases, pulmonary hypertension, and right heart failure [848]. This can be used to assess whether the left ventricular end-diastolic volume was properly corrected by fluid resuscitation, and also acts as a basis for determining whether fluid resuscitation to the extent of inducing atrioventricular valve regurgitation was an overload. Ranjit et al. instituted standard management of pediatric septic shock as well as echocardiography assessments within 6 h after diagnosis, and reported that fluid resuscitation and cardiovascular agent adjustments were possible in many patients [866]. However, it should be noted that it is still unclear, including the evidence from this study, whether adding hemodynamic assessment via echocardiography into the standard management would improve prognosis.

Finally, many reports have indicated the harmful effects of fluid overload in both adults and children. A systematic review of children in the ICU performed by Alobaidi et al. indicated that fluid overload was correlated with an increased mortality rate, lengthening of ventilation duration, and worsened acute kidney injury [850], and that efforts to avoid fluid overload are essential. When increased work of breathing, moist rales, hepatomegaly, or a galloping sound on auscultation are found during initial fluid resuscitation, fluid administration should immediately be suspended [848], fluid overload should be suspected, and the preload conditions should be re-assessed including echocardiography.

**CQ18-6: What is the initial fluid infusion rate and volume for pediatric sepsis?**

***Answer:*** In children with sepsis not complicated by heart failure, there is a method for repeating a bolus administration 10–20 mL/kg at a time while assessing response to an initial fluid resuscitation. Meanwhile, the occurrence of clinical findings which suggest fluid overload or a blunted fluid response should serve as a reference for suspending fluid resuscitation. There is no high-quality evidence regarding the upper limits of fluid infusion rate or volume (Provision of information for background question).

***Rationale***

Proper initial fluid resuscitation is important in the treatment of sepsis. The pediatric septic shock initial treatment algorithm [3, 4] and American College of Critical Care Medicine–Pediatric Advanced Life Support (ACCM–PALS) algorithm [848] indicate that when septic shock is suspected, bolus administrations of 20 mL/kg of isotonic crystalloid solution can be administered over 5–10 min, with repeated administrations up to 40–60 mL/kg in the first hour if needed when symptoms of shock persist. Furthermore, there have been reports of improved survival or reduced length of hospital stay due to treatment, which followed the ACCM-PALS algorithm [830, 867, 868] or initial treatment algorithm [869–874], including rapid fluid resuscitation.

However, a multicenter, open-label RCT (Fluid Expansion As Supportive Therapy [FEAST] trial) that investigated the effects of initial fluid resuscitation in children with high fever accompanied by circulatory insufficiency (including children with septic shock) showed that the mortality rate was higher in the group with rapid fluid resuscitation than in the group that did not undergo this procedure [828]. This study was conducted in a clinical environment in which intensive care management, including mechanical ventilation, was unavailable, which was different from the situation in Japan, but suggests the need to recognize the risks of fluid overload in the treatment of sepsis. An RCT that compared 20 mL/kg fluid bolus administrations every 15–20 min and every 5–10 min among children with septic shock reported a higher risk of requiring mechanical ventilation in the latter group [875]. Furthermore, the possibility that 20 mL/kg as a single dose of fluid bolus induces fluid overload has been investigated [876].

Taking these findings into consideration, initial resuscitation using rapid fluid infusion in a medical environment in which intensive care management is available in Japan has been the basis of treatment of pediatric sepsis; however, a somewhat conservative fluid bolus administration of 10–20 mL/kg of isotonic crystalloid solution is more valid than a conventional amount of 20 mL/kg. It is also important to assess fluid overload and blunted responsiveness to fluid during and after bolus fluid administration.

The presence of moist rales, respiratory distress, and an enlarged liver, which suggest the possibility of fluid overload, serves as a reference for suspending fluid resuscitation. Furthermore, fluid responsiveness can be assessed by improvements in peripheral circulation (e.g., reduced peripheral/central system temperature difference), increased blood pressure, reduced heart rate, increased urine output, and improvements in the level of consciousness (see CQ18–5). However, if the response becomes blunt as bolus infusions are intermittently repeated, the suspension of fluid resuscitation or slowing of fluid administration should be considered [3, 4, 848]. It should be noted that there is no high-quality evidence on the upper limit of the fluid infusion rate or volume.

**CQ18-7: Should dopamine be used as a first-line vasoactive agent in children with septic shock?**

***Answer:*** We suggest against using dopamine ad a first-line vasoactive agent in children with septic shock, and instead suggest selecting either adrenaline or noradrenaline according to hemodynamics (for adrenaline - GRADE 2D: certainty of evidence = “very low”; for noradrenaline - expert consensus: insufficient evidence).

***Rationale***

The J-SSCG2016 [3, 4] positioned adrenaline as a first-line inotropic/vasoactive agent for use among children with septic shock. However, as it did not make a clear recommendation for or against the use of dopamine, dopamine may still be used frequently in clinical practice in Japan [877]. A systematic review yielded 2 RCTs that conformed to the PICO criteria [823, 878], and we conducted a meta-analysis of these trials. Both RCTs set adrenaline as a comparative control.

With regard to the desirable effects of dopamine relative to adrenaline, the estimated effects of the length of stay in the pediatric ICU yielded a MD of 1.00 days shorter (95%CI: 3.95 shorter to 1.95 longer) (1 RCT, *n* = 60) [878]. With regard to the undesirable effects of dopamine relative to adrenaline, the estimated effects for 28-day mortality yielded a RD of 136 more per 1000 (95%CI: 61 fewer to 590 more) (2 RCTs, *n* = 180) [823, 878], that for resolution of shock within 1 h yielded an RD of 286 fewer per 1000 (95%CI: 368 fewer to 58 fewer) (1 RCT, *n* = 60) [823], that for vasoactive drug-free days yielded a MD of 4.80 days shorter (95%CI: 8.44 shorter to 1.16 shorter) (1 RCT, *n* = 120) [878], and that for serious adverse effects (healthcare-associated infections and ischemia) yielded an RD of 126 more per 1000 (95%CI: 50 fewer to 764 more) (2 RCTs, *n* = 180) [823, 878]. Accordingly, the desirable effects of dopamine were deemed trivial, whereas the undesirable effects were deemed moderate. Therefore, we adjudged that the balance of effects between desirable and undesirable effects was such that the comparative control of adrenaline was superior.

We found no RCTs on the desirable and undesirable effects of dopamine relative to noradrenaline; therefore, these effects remain unclear. However, using noradrenaline, which mainly stimulates α-receptors, seems a pharmacologically rational choice in patients presenting with hemodynamic features of vasodilatory shock. Meanwhile, the risk of healthcare-associated infections due to immunosuppression through suppression of prolactin secretion may exist only among patients who receive dopamine since the actions on dopamine receptors are limited to dopamine. Accordingly, in patients with hemodynamic features of vasodilatory shock, the desirable effects of dopamine are likely to be trivial, whereas the undesirable effects are likely small; therefore, we adjudged that the comparative control of noradrenaline was likely superior.

It should be noted that the 2 RCTs used in this CQ [823, 878] do not have the same dose adjustment protocols for dopamine and adrenaline. Furthermore, this recommendation does not preclude the use of dopamine under circumstances in which adrenaline or noradrenaline is unavailable.

**CQ18-8: Should vasopressin be used as a vasoactive agent in children with septic shock?**

***Answer:*** We suggest against using vasopressin as a vasoactive agent in children with septic shock (GRADE 2D: certainty of evidence = “very low”).

***Rationale***

Vasopressin may improve the hemodynamic conditions of children with septic shock via a vasopressor effect based on a mechanism that is different from that of other catecholamines and may allow us to avoid extracorporeal membrane oxygenation therapy. However, harms that may ensue, such as ischemia or worsening prognosis and the balance between its benefits and harms are unclear. A systematic review yielded 2 RCTs that conformed to the PICO criteria [879, 880], and we conducted a meta-analysis of the results of these trials.

The interventions include the administration of vasopressin [879] and its derivative terlipressin [880], with comparative controls being placebo and conventional treatments, respectively. The estimated effects for the length of stay in the pediatric ICU (2 RCTs, *n* = 123) yielded a MD of 3.64 days shorter (95%CI: 9.82 shorter to 2.53 longer). The desirable effects were deemed to be small. The estimated effects for mortality (2 RCTs, *n* = 123) yielded a RD of 60 more per 1000 (95%CI: 130 fewer to 250 more) [879, 880], and that for time to vasoactive drug-free hemodynamic stability (1 RCT, *n* = 65) yielded a MD of 2.60 h longer (95%CI: 49.95 shorter to 55.15 longer) [879]. Furthermore, the estimated effects of serious adverse events (digital ischemia, thrombosis, cardiac arrest, and gastrointestinal bleeding) (2 RCTs, *n* = 123) yielded a RD of 40 more per 1000 (95%CI: 60 fewer to 140 more) [879, 880]. Therefore, the undesirable effects due to vasopressin were moderate. Based on the above, we adjudged that the balance of its effects was likely in favor of the comparative control.

When considering the administration of vasopressin, serious adverse effects such as digital ischemia should be carefully monitored while evaluating in each patient whether desirable effects can be expected and indiscriminate drug administration is discouraged.

**CQ18-9: Should corticosteroids be administered to children with septic shock when they do not respond to initial fluid resuscitation and inotropic agents?**

***Answer:*** We suggest against the routine administration of corticosteroids in children with septic shock when they do not respond to initial fluid resuscitation and inotropic agents (GRADE 2D: certainty of evidence = “very low”).

***Rationale***

Three RCTs were included in the analysis for mortality (*n* = 155) [824, 825, 881], and the estimated effects yielded a risk difference of 40 fewer per 1000 (95%CI: 167 fewer to 130 more). Furthermore, 2 RCTs were analyzed for time to recovery from shock [825, 881]. One RCT (*n* = 68) [825] showed averages of 60.0 h (routine steroid administration group) and 139.2 h (comparative control group). The other RCT (*n* = 38) [881] showed median values of 49.5 h (routine steroid administration group) and 70 h (comparative control group), with effects estimated to be present to some extent. Therefore, the desirable effects were deemed small. In terms of the risk of secondary infection (2 RCTs, *n* = 87) [824, 881], the estimated effects yielded a risk difference of 41 more per 1000 (95%CI: 73 fewer to 284 more). Two RCTs were analyzed in terms of the length of stay in hospital [824, 825]. One RCT (*n* = 68) [825] showed averages of 11.4 days (routine steroid administration group) and 8.2 days (comparative control group), and the other RCT (*n* = 49) [824] showed median values of 10.7 days (routine steroid administration group) and 9.6 days (comparative control group), with slight extensions estimated in the intervention group. Therefore, the desirable effects were deemed small. Based on the above, both the desirable and undesirable effects of the intervention were deemed small, and we adjudged that neither the intervention nor the comparative control could be supported regardless of the relative value of outcomes placed by patients and families.

Note that steroid cover is essential regardless of the presence of shock when patients with congenital adrenal hyperplasia or those who have been receiving systemic steroids for a long period of time are afflicted with sepsis.

**CQ18-10: When should blood infusions be started in hemodynamically stable children with sepsis?**

***Answer:*** We suggest starting blood transfusions with a hemoglobin level of 7.0 g/dL as a threshold for critical, hemodynamically stable children with sepsis (GRADE 2C: certainty of evidence = “low”).

***Rationale***

The thresholds of red blood cell transfusion should be carefully considered in pediatric intensive care in terms of the diversity of disease backgrounds, the handling of patients with a wide range of ages and body weights, and avoiding unnecessary transfusion. We conducted a systematic review of the transfusion threshold among critically ill children with stable hemodynamics, and 2 RCTs were included in the analysis [826, 882].

In both RCTs, the threshold of the hemoglobin concentration for initiating blood transfusion was lower in the intervention group (7 g/dL in both trials) and higher in the control group (Lacroix et al., 2007: 9.5 g/dL [882] and Akyildiz et al., 2018: 10 g/dL [826]). With regard to all-cause mortality (2 RCTs, *n* = 797), the estimated effects of the intervention yielded a RD of 6 fewer per 1000 (95%CI: 28 fewer to 38 more). With regard to blood transfusion complications (1 RCT, *n* = 637), the estimated effects of the intervention yielded a risk difference of 28 more per 1000 (95%CI: 62 fewer to 153 more) [882]. Furthermore, with regard to the length of stay in the ICU (2 RCTs, *n* = 797) and the duration of mechanical ventilation (2 RCTs, *n* = 797), the estimated effects of the intervention yielded a MD of 0.62 days shorter (95%CI: 1.76 shorter to 0.51 longer) and a MD of 0.00 days (95%CI: 0.84 shorter to 0.84 longer), respectively [826, 882]. Therefore, it was adjudged that neither the intervention nor the comparative control was superior to the other. The direction of the estimated effects for all the outcomes were consistent; thus, the overall certainty of the evidence was “low”. Based on the 2 RCTs included in this CQ, it was thought that starting blood transfusion was valid when hemoglobin levels were below 7 g/dL in critically ill septic children with stable hemodynamics.

Note that starting blood transfusion at a higher threshold may need to be considered in children with some underlying conditions such as cyanotic heart diseases.

**CQ18-11: Should blood purification therapy (including plasma exchange) be used to treat children with sepsis without acute kidney injury?**

***Answer:*** We suggest against using blood purification therapy to treat children with sepsis without acute kidney injury (GRADE 2D: certainty of evidence = “very low”).

***Rationale***

We conducted a systematic review because the decisions varied as to whether to initiate blood purification therapy in the treatment of children with sepsis in clinical settings.

Only one trial was included in the analysis [883]. There were no data related to the length of stay in the ICU, the duration of mechanical ventilation, or the time to withdrawal from shock. With regard to all-cause mortality (1 RCT, *n* = 48), the estimated effect yielded a risk difference of 377 more per 1000 (95%CI: 30 fewer to 1000 more); thus, the desirable effects were deemed trivial. There were no data related to serious adverse events, so this could not be analyzed. Even considering that the estimated effect for mortality was derived from one small-sized RCT, the undesirable effects of the intervention were deemed moderate [883]. Therefore, the balance of desirable and undesirable effects was likely in favor of the comparative control. However, we do not deny decisions to implement the intervention due to case-dependent indications.

Note that the recommendation of this CQ does not negate the use of plasma exchange for indicated underlying diseases or renal replacement therapy for severe acute kidney injury and fluid overload refractory to diuretics.

**CQ18-12: Should intravenous immunoglobulin (IVIG) therapy be administered in children with sepsis?**

***Answer:*** We suggest against administering IVIG for children with sepsis (expert consensus: insufficient evidence).

***Rationale***

IVIG therapy for severe infections is listed in the National Health Insurance registry of Japan, and is widely used, although its efficacy in improving clinical prognosis remains uncertain. Larger doses have been attempted overseas for immunomodulation; however, their effects have not been consistent across studies. Furthermore, high-quality RCTs in the field of pediatrics (apart from neonatology) are lacking [884–887]. It has been suggested that IVIG should not be administered to adult patients with sepsis. We conducted a systematic review since the evaluation of the effectiveness/harmfulness of IVIG administration to children with sepsis has not been established.

Although one RCT was extracted [888], this was an extremely small-scale and biased article; thus, the committee unanimously agreed to avoid making recommendations based on this evidence alone. Considering that the favorable effects of IVIG could not be expected in adult patients (see CQ5–1) and that the therapeutic effects of IVIG in severe infection were clearly negated in the high-quality, large-scale, multi-center RCT conducted mainly among neonates (the INIS trial) [887] and meta-analyses that include it [884, 889] it is reasonable to assume that the desirable effects of IVIG are also trivial in children. Serious adverse effects of IVIG include anaphylaxis, acute kidney injury, liver dysfunction, aseptic meningitis, and extravasation, which are not serious and rare. Thus, the undesirable effects are deemed trivial. Both the desirable and undesirable effects are trivial, and neither the intervention nor the comparative controls are superior to the other. For this reason, we do not recommend the administration of IVIG as standard therapy for all children with sepsis.

**CQ18-13: Should blood glucose level be controlled tightly in children with sepsis?**

***Answer:*** We suggest against controlling blood glucose level tightly in children with sepsis (GRADE 2C: certainty of evidence = “low”).

***Rationale***

Hyperglycemia may affect immunity, exacerbate infection, and worsen patients’ prognoses, resulting in a higher mortality rate and a longer length of stay in hospital in both children and adults [890–893]. Therefore, glycemic control is an important aspect in the management of sepsis among children. In contrast, hypoglycemia induced by insulin is an important hazard of glycemic control and has been associated with poor prognoses among critically ill children [891, 894]. Therefore, we conducted a systematic review to determine whether to exercise tight glycemic control in children with sepsis. The significance of tight glycemic control was unlikely to differ between children with sepsis and other critically ill children; therefore, the subject of this study was not limited to sepsis.

Five RCTs [827, 895–898], were included in the analysis. The estimated effects for all-cause mortality (5 RCTs, *n* = 3923) yielded a RD of 1 fewer per 1000 (95%CI: 14 fewer to 17 more) [827, 895–898] and the length of stay in the ICU (3 RCTs, *n* = 3049) yielded a MD of 0.50 days shorter (95%CI: 0.52 shorter to 0.48 shorter) [895, 897, 898], and the duration of mechanical ventilation (3 RCTs, *n* = 3049) yielded an MD of 0.30 days shorter (95%CI: 0.32 shorter to 0.27 shorter). The desirable effects of the intervention were deemed trivial. The estimated effects of the frequency of hypoglycemic events (5 RCTs, *n* = 3933) yielded an RD of 105 more per 1000 (95%CI: 66 more to 166 more) [827, 895–898], and the undesirable effects of the intervention were deemed significant. Therefore, the balance of effects was such that the comparative control was likely superior, and we suggested against the intervention. Note that the recommendation of this CQ does not negate the use of insulin among children with persistent hyperglycemia (with a serum glucose level above 180 mg/dL), which is thought to cause osmotic diuresis.

**CQ19: Neuro intensive care**

**Introduction**

Sepsis causes various types of organ failure, with the brain being one of the affected organs; several symptoms have been identified with this condition [899]. Furthermore, the mortality rate among sepsis patients with acute brain dysfunction is significantly higher than in sepsis patients without such dysfunction. There are various causes leading to acute brain dysfunction during sepsis, and the underlying pathophysiological mechanisms are complex [900]. Therefore, it is important to differentiate between sepsis-related acute brain dysfunction and neurological disease complications. It is possible to institute early stage interventions for treatable causes and improve the neurological prognosis [899]; thus, it is important to differentiate and diagnose acute brain dysfunction in patients with sepsis.

It is not rare for sepsis patients to have neurological abnormalities. It is important not to overlook acute brain dysfunction, which requires additional treatment and changes during treatment, such as cerebral infarction, non-convulsive status epilepticus, drug-induced encephalopathy, and secondary meningitis, in addition to sepsis-related acute brain dysfunction in in which sepsis treatment is the primary element. It was thought that this should be raised as CQs in this guideline for this reason.

**Fig. 18:**
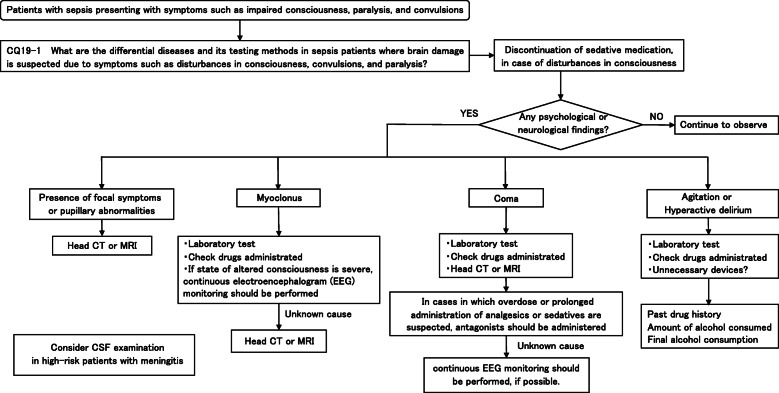
CQ19: Neuro intensive care (clinical flow)

Clinical flow of these CQs is shown in Fig. 18.

**CQ19–1: What are the differential diseases and its testing methods in sepsis patients where brain damage is suspected due to symptoms such as disturbances in consciousness, convulsions, and paralysis?**

***Answer:*** Intracranial lesions (e.g., stroke) and potential causes (e.g., metabolic disorders) are first differentiated with the assumption that there may be compound causes for brain damage. Tests include neuroimaging, continuous electroencephalography (EEG) monitoring, biochemical tests, confirmation of the causative agent, and cerebrospinal fluid examination if necessary. Neuroimaging are performed urgently if focal neurologic signs were observed (Provision of information for background question).

***Rationale***

The causes of acute brain dysfunction due to sepsis can be divided into (i) narrowly defined sepsis-associated brain damage, (ii) broadly defined sepsis-associated brain damage, and (iii) neurological disease complications of sepsis [899, 901]; however, in reality, many of these pathophysiologies overlap [902]. Categories (ii) and (iii), in particular, require specific treatment, therefore, differentiation is important. Acute brain dysfunction due to sepsis includes a wide range of symptoms including delirium, mild altered states of consciousness, and coma [901].

*Classifications of brain dysfunction due to sepsis*

A) Narrowly defined sepsis-associated brain damage

This directly influences the brain through inflammatory mediators, and is a pathological condition referred to as sepsis-associated encephalopathy [901]. The increased levels of inflammatory mediators that accompany sepsis can cause vascular endothelial cell activation, disruption of the blood-brain barrier, disruption of vascular autoregulatory functions, neutrophil migration into the brain tissue, microglia activation, regulatory neurotransmitter adjustment disorders, and mitochondrial failure as well as induce diffuse acute brain damage. MRI may reveal leukoencephalopathy in severe cases [903, 904].

B) Broadly defined sepsis-associated brain damage.

This refers to brain dysfunction caused by organ failure (outside the brain) due to sepsis, including hypotension, hypoxia, uremia or electrolyte abnormalities due to renal dysfunction, hyperammonemia due to hepatic dysfunction, or indirectly caused by drugs [899, 901, 903].

III) neurological disease as a complication of sepsis

This refers to new pathological conditions in the central nervous system caused by meningitis that occur concomitantly with infectious endocarditis, subarachnoid hemorrhage due to the rupture of an infectious cerebral aneurysm, cerebral abscesses, cerebral infarction due to decreased cerebral perfusion, and status epilepticus.

*Differential diagnosis of acute brain dysfunction due to sepsis and their testing methods*

Of the acute forms of brain dysfunction that occur due to sepsis, all classifications other than narrowly defined sepsis-associated brain damage involve cases in which some form of intervention is needed in addition to sepsis treatment. Therefore, it is important to diagnose and classify acute brain dysfunctions among patients with sepsis. Sedatives should be discontinued, or their doses reduced if possible, and the differential diagnosis should begin by performing physical examination after minimizing drug effects. The following is an example of the process of differentiation based on physical findings [901, 905]: (1) Are there focal symptoms or pupillary abnormalities? (2) Is there myoclonus? (3) Is the patient comatose? (4) Is the patient in a state of agitation or hyperkinetic delirium?

1. If focal symptoms or pupillary abnormalities are present, organ-based abnormalities such as cerebral infarction due to hypotension or hypoperfusion and cerebral hemorrhage due to coagulation disorders will be ranked higher in the differentiation, and neuroimaging tests using computed tomography or MRI should be prioritized.
2. If myoclonus is present and the state of altered consciousness is mild, the possibility of electrolyte abnormalities, uremia, metabolic abnormalities such as hepatic encephalopathy, or drug-induced encephalopathy due to antibacterial drugs should be considered, and both biochemical tests and confirmation of the drug used should be prioritized. The European Society of Intensive Care Medicine recommends that the possibility of complications of non-convulsive status epilepticus should be considered in patients with metabolic abnormalities due to renal or liver injury or when drug-induced encephalopathy due to antibacterial drugs is the cause, and that continuous electroencephalogram (EEG) monitoring should be performed [906].
3. If the patient is comatose, non-convulsive status epilepticus, metabolic abnormalities, and drugs are ranked higher in the differentiation. However, it is important to first rule out organ-based disease complications such as intracranial hemorrhage, which require emergency intervention. Blood investigations and drugs are confirmed after conducting neuroimaging tests. If the causes are still unclear, continuous EEG monitoring should be performed if possible. In cases in which overdose or prolonged administration of analgesics or sedatives are suspected, antagonists such as flumazenil and naloxone should be administered, and improvements in the level of consciousness should be confirmed. If there is no evidence of seizure waves on the EEG, and the EEG predominantly shows slow waves, theta waves, or suppression patterns, narrowly defined sepsis-associated brain damage, or broadly defined sepsis-associated brain damage, such as diffuse cerebral ischemia due to hypoperfusion, analgesic overdose, or prolongation of its effects can be differentiated as the causes of altered states of consciousness [900].
4. If the patient is in a state of agitation or hyperkinetic delirium, electrolyte abnormalities and metabolic abnormalities should be confirmed as well as any drugs or unnecessary devices that prolong delirium. Alcohol use and benzodiazepine withdrawal are often overlooked as causes of delirium, and it is important to confirm the past drug history, amount of alcohol consumed, and final alcohol consumption [901].

It is important to consider the neurological disease as a complication of sepsis in addition to the differentiations listed in items (1)–(4) above. Among these, the complications of meningitis require changes in the type of antibacterial drug and dose; thus, the diagnosis is particularly important in these cases. Among the various types of sepsis of non-central nervous system origin, meningitis complications commonly include bacterial pneumonia, otitis media, sinusitis, and infectious endocarditis [907]. It is often impossible to differentiate which among combinations of infectious endocarditis and meningitis is secondary, and the frequency of meningitis in infectious endocarditis varies according to each study, ranging from 0 to 20% [907, 908]. *Staphylococcus aureus* and *Streptococcus pneumoniae* are the most common causative bacteria of secondary meningitis from distant sources [907, 908]. Retrospective studies that investigated 1025 meningitis patients showed that *Staphylococcus aureus* (33%) and *Streptococcus pneumoniae* (54%) accounted for a majority of the causative bacteria among patients who had both meningitis and infectious endocarditis [909, 910]. Alcohol addiction and immunodeficiencies were reported as risk factors among patients.

**CQ20: Patient- and Family-Centered Care**

**Introduction**

The relatively short-term vital prognosis of sepsis patients has dramatically improved in recent years due to developments in intensive care medicine, accumulation of evidence, and the spread of clinical practice guidelines [911]. Meanwhile, a multilateral RCT that targeted sepsis patients [912] showed that of 2130 patients with independent lifestyles prior to hospitalization, approximately one-third died within 6 months, and of the 580 patients for whom quality of life measurements could be performed after 6 months, 41.6% were unable to have independent lifestyles. In light of these circumstances, the Society of Critical Care Medicine proposed the important concept of PICS in 2012 [816]. PICS is physical, cognitive, or mental impairment that occurs during or after discharge from the ICU, or even after discharge from the hospital. It is a pathological condition that affects not only the long-term prognoses of critically ill patients who require intensive care for conditions such as sepsis, but also the mental health of their families. Japan in particular is an aging country unlike any in the world, and more than 25% of its total population is over the age of 65 years [913]. A structure in which there is an increasing number of people in need of care as the lifesaving rate increases cannot be said to be a healthy state from a social perspective, and it is self-evident that the ways in which PICS can be prevented and improved will become an increasingly serious problem in sepsis treatment in the future.

The J-SSCG 2016 [3, 4] was the first guideline in the world to take up PICS as an independent chapter, and recommendations relating to early rehabilitation in order to prevent PICS are described. According to a survey of members of the Japanese Society of Intensive Care Medicine (453 respondents), early rehabilitation was initiated in 92.1% of respondents, due in part to recommendations made by guidelines and support with regard to medical fees [914]. Meanwhile, approximately 40% of respondents’ facilities were either unaware of or did not use the terms PICS or ABCDEF bundles [914]. Intensive care should be individualized. Furthermore, the ICU is a site for intensive care; however, it is also a place in which patients live. There are many CQs that should be considered: what considerations are necessary during an ICU stay in order to administer clinical treatments that respect the humanity of patients with various value systems and ways of thinking?, what should the relationship be between patients and their families?, and what should health professionals do in order to provide mental support to these people? In this context, the J-SSCG 2020 contains a new independent chapter, in which “Patient- and Family-Centered Care” was taken up as a topic. Content primarily relating to physical function was addressed in the chapter regarding “ICU-AW/PICS/early rehabilitation”, whereas the chapter on “Patient- and Family-Centered Care” was positioned to handle content relating to the mental state of patients and their families, and the care environment and decision-making support in the ICU. A total of six CQs, including two background questions, were taken up by a multidisciplinary working group for this chapter. There are some with poor levels of evidence; however, these are extremely important areas that can improve the quality of future sepsis treatment and intensive care. We hope that “Patient- and Family-Centered Care”, which respects the humanity of the individual patient and family, will serve as a basis for exploring what the main concepts in this subject should be.

**Fig. 19:**
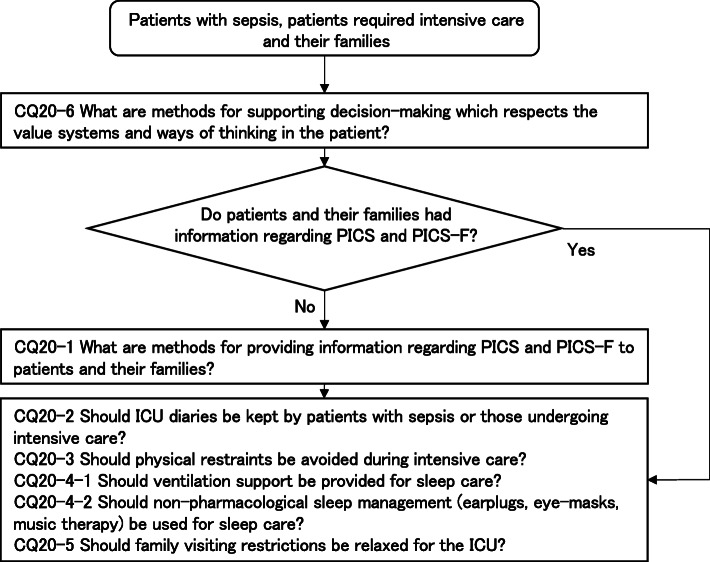
CQ20: Patient-and Family-Centered Care (clinical flow)

Clinical flow of these CQs is shown in Fig. 19.

**CQ20-1: What are methods for providing information regarding PICS and PICS-F to patients and their families?**

***Answer:*** Providing accurate yet continuous information regarding PICS and PICS-F to patients and their families is thought to be important. There are increasing tendencies among medical staff working with the patient to provide handouts at the time of ICU admission/discharge and providing appropriate information. There are initiatives which continuously provide information, such as rounds after discharge from the ICU and the establishment of follow-up outpatients (Provision of information for background question).

***Rationale***

In a survey conducted among the members of the Japanese Society of Intensive Care Medicine, 61% of those who worked in the ICU were familiar with or used the terms and disease concepts of PICS [914]. It is difficult for patients and their families to obtain information relating to PICS and PICS–Family (PICS–F) when many health professionals working in the ICU are unfamiliar with PICS. Meanwhile, PICS and PICS–F occur at a high rate among sepsis patients and their families, respectively [915]. For this reason, many patients and their families confront PICS and PICS–F with insufficient information and live with various forms of pain, anxiety, fear, and conflicts toward treatment. Accurately, yet continuously providing information relating to PICS and PICS–F to patients and families can lead to understanding and reassurance that PICS and PICS–F are not special abnormalities that only occur among patients or their loved ones [916]. Furthermore, this may lead to advanced prediction, early detection, and rapid response to PICS and PICS–F [916].

Handing out leaflets at the time of ICU admission/discharge is an extremely simple method of providing information. Appropriate information can be provided by creating a leaflet that includes an overview of PICS and PICS–F, their symptoms, and contact information, which can then be handed out to patients and their families upon ICU admission/discharge. It is important in such cases that there is dual communication among patients, their families, and health professionals so that this does not end with one-sided provision of information. A multi-center RCT showed that providing leaflets which included an overview of the ICU and information relating to medical equipment, improved family understanding and satisfaction [917]. However, little research has verified the usefulness of providing leaflets that includes information on PICS and PICS–F, and further research is needed.

Rounds and visits after discharge from the ICU are methods through which ICU physicians and nurses provide information to patients after discharge from the ICU by visiting their beds. A report has indicated that 46% of patients had false delusional memories such as nightmares or hallucinations after discharge from the ICU [918]. Rounds and visits after discharge from the ICU do not only compensate for discrepancies and unclear aspects relating to ICU experience and treatment understanding, but also assess disease conditions and dysfunction. It is expected that this may have the effect of adjusting at an early stage the need for ICU readmission and appropriate specialist outpatient consultations, in coordination with the attending physician. A qualitative study [919] reported that visits after ICU discharge helped with the understanding of the ICU experience, and a report has indicated that the use of support programs for correcting memory distortions during visits after discharge from the ICU resulted in significant improvements in anxiety, depression, and stress disorders after discharge from the hospital [920].

Outpatient follow-ups after discharge from the ICU have primarily increased in Europe over the last 20 years. Outpatient follow-ups were set up primarily for patients who stayed for at least 3–4 days in the ICU in 30% of ICUs in the United Kingdom (UK) in 2006 [921]. The primary medical care provided during outpatient follow-ups included physical, mental, and cognitive function, and quality of life (QOL) assessments using screening tools, rehabilitation, mental/cognitive function support, introduction to the appropriate specialist outpatient, and medication management. An RCT was conducted in three facilities in the UK to assess the effectiveness of outpatient follow-ups; however, no significant improvements in QOL, anxiety, depression, or PTSD were observed at 12 months after hospital discharge [922]. Outpatient follow-ups forms and methods as well as subject patients, have not been sufficiently studied, and further detailed studies are needed in the future. Furthermore, the establishment of a medical system, including medical fees, is essential for this to become widespread in Japan.

The effectiveness of providing information relating to PICS and PICS–F to patients and their families has not been sufficiently validated. The implementation rate in ICUs in Japan was also low at less than 10% [914]; however, it is thought that its implementation would expand depending on future research.

**CQ20-2: Should ICU diaries be kept by patients with sepsis or those undergoing intensive care?**

***Answer:*** We suggest keeping an ICU diary for adult patients with sepsis or those undergoing intensive care (GRADE 2D: certainty of evidence = “very low”).

***Rationale***

A systematic review identified 3 RCTs that conformed to the PICO criteria of this CQ which investigated the effects of keeping ICU dairies on adult sepsis patients or intensive care patients [923–925]. We performed a meta-analysis of these trials. It should be noted that no RCTs that were limited to sepsis patients were found; thus, the subjects were patients with sepsis or those undergoing intensive care.

The estimated value of the effects of incidence of post-traumatic stress disorder due to intervention was 51 fewer per 1000 (95%CI: 123 fewer to 41 more). Furthermore, the Hospital Anxiety and Depression Scale (HADS) anxiety score decreased by an average of 0.82 (95%CI: 2.45 lower to 0.82 higher) and HADS depression score decreased by an average of 1.01 (95%CI: 3.55 lower to 1.53 higher) due to the intervention. Therefore, the desired effects of intervention were judged to be small.

One RCT evaluated how troublesome ICU diaries were as an adverse event. The extent of troublesomeness was evaluated on a 10-point scale, with “not at all troublesome” being scored 0 and “the most troublesome” scored 10. Families (*n* = 78) scored a mean of 0.69 ± 1.46, friends (*n* = 4) scored 2.0 ± 2.45, nurses (*n* = 98) scored 1.6 ± 0.19, doctors (*n* = 12) scored 1.75 ± 1.48, and medical staff other than nurses (*n* = 6) scored 1.0 ± 0.63, with results showing that the intervention was not particularly troublesome. Therefore, the undesired effects of intervention were thought to be trivial.

The estimated value of effects in this CQ varied widely and had low certainty; however, it was judged that the intervention was likely superior.

**CQ20-3: Should physical restraints be avoided during intensive care?**

***Answer:*** We suggest avoiding physical restraints during intensive care for adult patients with sepsis or those undergoing intensive care (GRADE 2C: certainty of evidence = “low”).

***Rationale***

We integrated qualitative evidence from 16 qualitative studies [926–941] based on the Confidence in the Evidence from Reviews of Qualitative research (CERQual). Patients who underwent physical restraint at the ICU stated that they did not remember the physical restraint or that it was not a problem because it was to ensure safety; however, they also thought that it should not be implemented since it violates human rights and dignity (certainty of evidence: “low”). Family members thought that physical restraints were inevitable but felt sorry for the patient, and they felt grateful for the thoughtful explanations provided by health professionals and their efforts to minimize physical restraints (certainty of evidence: “very low”). Health professionals were concerned about the adverse events of physical restraints but still performed them to ensure safety while feeling helpless in a dilemma (certainty of evidence: “high”). As an alternative to physical restraint, health professionals thought that it was important to provide care that respected the individual as a human being, along with generous staffing and other structural arrangements (certainty of evidence: “high”).

The results of a meta-analysis of 15 observational studies [942–956] showed that the OR of delirium incidence (10 observational studies, *n* = 2184) was 0.09 (95%CI: 0.04 to 0.19), mechanical ventilation duration (2 observational studies, *n* = 1132) yielded a difference of 0.80 days shorter (95%CI: 6.71 shorter to 5.12 longer), the length of stay in the ICU (4 observational studies, *n* = 1105) yielded a difference of 3.99 days shorter (95%CI: 7.91 shorter to 0.07 shorter), and the OR of the occurrence of unplanned device removal (5 observational studies, *n* = 4878) was 0.36 (95%CI: 0.13 to 0.98). Significant correlations were thus seen in the intervention group, but the risk of bias of most of the primary studies was extremely severe, so it was difficult to show a causal relationship between the intervention and outcome.

The results of CERQual showed that implementing physical restraints during intensive care may violate the human rights and dignity of the patient and impose psychological burdens on health professionals (e.g., their feelings of powerlessness and inner struggle); therefore, it is thought that avoiding physical restraints provides a small benefit. The desired effects are small, and the undesired effects are not clear. On this basis, it was judged that the balance of effects was such that the intervention was likely superior.

**CQ20-4-1: Should ventilation support be provided for sleep care?**

***Answer:*** We suggest adding ventilation support as part of sleep care for adult patients with sepsis or those undergoing intensive care (GRADE 2D: certainty of evidence = “very low”).

***Rationale***

A previous systematic review [495] reported that additional ventilation support improved sleep care. Based on this, we performed another systematic review in which we added ventilation support as part of sleep care to the amount of objective sleep (total sleep time/total recording time, etc.) as outcomes. A meta-analysis of 5 RCTs conforming to the PICO criteria [957–961] showed that the estimated value of effects for the amount of objective sleep yielded a MD of 12.2 higher (95%CI: 4.12 higher to 20.28 higher), and the desired effects were thought to be small. There have also been no reports on the harms of adding ventilation support during mechanical ventilation, and it was difficult to evaluate the undesired effects. There were no reports on the harms associated with the intervention; however, considering that the onset of harm due to intervention is trivial in clinical settings, it was judged that adding ventilation support as part of sleep care was likely superior.

**CQ20-4-2: Should non-pharmacological sleep management (earplugs, eye-masks, music therapy) be used for sleep care?**

***Answer:*** We suggest non-pharmacological sleep management for adult patients with sepsis or those undergoing intensive care (GRADE 2D: certainty of evidence = “very low”).

***Rationale***

A previous systematic review [495] did not provide a clear answer as to whether non-pharmacological sleep management should be used as sleep care. Therefore, we performed a systematic review with subjective evaluations of sleep (e.g., patient questionnaires that used the Verran and Snyder–Halpern Sleep Scale and others) and the amount of objective sleep (total sleep time/total recording time, etc.). A meta-analysis of four RCTs that conformed to the PICO criteria [542, 962–964] showed that the estimated value of effects for subjective evaluations yielded a standardized mean difference (SMD) of 1.5 higher (95%CI: 1.11 higher to 1.9 higher). The estimated value of effects for the amount of objective sleep yielded a MD of 2.46 lower (95%CI: 9.94 lower to 5.01 higher) and it was thought that the desired effects were small. There were also no articles that discussed the harms of using eye masks, earplugs, and music therapy as sleep care, and this was difficult to evaluate. There were no articles that discussed the harms of intervention, but it was thought that the clinical harm due to intervention was small. Therefore, it was judged that non-pharmacological sleep management (eye masks, earplugs, and music therapy) was likely superior as sleep care.

**CQ20-5: Should family visiting restrictions be relaxed for the ICU?**

***Answer:*** We suggest relaxing family visiting restrictions for adult patients with sepsis or those undergoing intensive care (GRADE 2D: certainty of evidence = “very low”).

***Rationale***

We retrieved and merged data from three RCTs that met the PICO criteria of this CQ [965–967]. The results showed that relaxation of visiting restrictions reduced the incidence of delirium by 68 per 1000 (95%CI: 148 fewer to 132 more). There was no difference in the median duration of stay in the ICU between the intervention and control groups, which was 5.0 days (IQR 3.0 to 8.0) in both groups. Likewise, the effect of interventions on the occurrence of depression among patients yielded a mean HADS score of 0 (95%CI: 0 to 0). For family members, the median HADS depression score (intervention group/control group) was 4.0 (IQR 2.0 to 8.0)/5.0 (IQR 2.0 to 9.0) and the median HADS anxiety score was 6.0 (IQR 3.0 to 8.2)/7.0 (IQR, 4.0 to 11.0). Given the step-wise scoring systems of HADS with 0–7: normal, 8–10: borderline abnormal, and 11–21: abnormal, the difference in median score was not considered to be clinically significant. Based on these results, it was thought that the desirable effects due to the intervention were small.

Meanwhile, the incidence of any infections during ICU stay was evaluated as an undesirable effect. Based on the data derived from two of the RCTs (*n* = 1908), the relaxation of visiting restrictions resulted in a reduced incidence of infection during ICU stay by 4 per 1000 (95%CI: 20 fewer to 20 more), which suggested that the undesirable effects were trivial.

In conclusion, the relaxation of visiting restrictions was expected to have desirable effects on the incidence of delirium, although the effects were small, whereas it is suggested that the undesirable effects due to the intervention were trivial. Although the certainty of the evidence is extremely low, the relaxation of visiting restrictions is likely to be superior.

**CQ20-6: What are methods for supporting decision-making which respects the value systems and ways of thinking in the patient?**

***Answer:*** There are methods which support decision making which respects the value systems and ways of thinking of the patient through repeated multi-disciplinary conferences including patients and their families. Methods which carefully identify surrogate intention-estimating individuals (e.g., families) who estimate the intentions of the patient themselves have been proposed when the intentions of the patient are unclear. It is important to respect the intentions of the patients as well as to provide medically accurate information to patients and their families (Provision of information for background question).

***Rationale***

The importance of decision-making support is increasing as medical care becomes increasingly complex, and its value systems, thought processes, and lifestyles become increasingly diverse. Surveys conducted in Japan indicated that many Japanese citizens wished to decide their own treatment policy upon consultation with or explanation from their physicians [968]. Meanwhile, a report indicated examples in which the treatment policy was changed upon the decision of other family members, regardless of the decision on the treatment policy made by the patients themselves or surrogate decision makers [969]. In such a context, decision-making support based on informed consent or advance directives (ADs) has been promoted; however, a large-scale cluster RCT that validated the effectiveness of AD showed no significant improvements in the quality of care and patient outcomes [970]. This was because it was difficult for the patients themselves to make predictions due to the complexity of the actual circumstances, and that it was not clear whether the decision made at the time would remain the same on the present day. There are rapid changes in medical conditions and the environment, particularly in the fields of emergency/intensive care; thus, these tendencies are thought to be pronounced here. For these reasons, discussions over time, rather than informed consent and AD at a single moment in time, have become more important.

Shared decision-making (SDM) and advance care planning (ACP) have recently been proposed. These methods are a continuous and two-way process that supports decision-making by patients and their families (including not only families, but acquaintances and friends trusted by patients and whom they would like to make treatment/care decisions on their behalf). Health professionals provide accurate information that serves as evidence of patients’ conditions and treatment options/methods, and patients and their families can provide information such as the value systems and ways of thinking of the patients themselves. Patient-based decision-making is the basis of this process, and it has been proposed that treatment policy decisions are made through discussions in repeated multi-disciplinary conferences [971]. SDM is the process of dialogue and thinking about what is best for patients, which in turn serves as the basis for ACP. When the patient cannot confirm their ways of thinking, surrogate decision makers such as family and others should be carefully identified, the estimated ways of thinking of the patient are respected, and the best policy for the patient is proposed. Furthermore, if the family or others are unable to presume the patient’s ways of thinking, there is a method by which sufficient discussion with the family and others is held through multi-disciplinary conferences, based on the policy of what is best for the patient [971]. These methods are not completed once a decision has been made, and it is considered important to repeat this process according to the passage of time, changes in the mental and physical conditions of the patient, and changes in medical assessments. Furthermore, it is recommended that the contents of the discussion during this process should be recorded in writing each time [971].

The development of emergency and intensive care medicine has enabled the lives of sepsis patients (who could not be saved with conventional methods) to be saved [972]. The terminal stages in the fields of emergency/intensive care have changed alongside this, and sufficient discussion with medical teams comprising multiple physicians (ideally from multiple departments), including the attending physician, nurses, and other health professionals are needed to clarify the terminal stage [973]. It is difficult to conclusively define the terminal stage; however, it is important to provide medically accurate information to patients, their families, and others so as not to lead lives that can clearly be saved into the terminal stage or mistakenly recognize life-prolonging treatments as life-saving actions.

Reports have indicated that these types of SDM and ACP discussions have reduced stress, depression, and anxiety among families after bereavement [974, 975]. The efficacies of SDM and ACP have not yet been sufficiently validated; however, its implementation is thought to expand with future research and medical systems.

**CQ21: Sepsis Treatment System**

**Introduction**

The diagnostic criteria for sepsis have been redefined, and medical professionals are also required to change to a system for treating more serious infectious diseases. In the J-SSCG 2020, a new section on the sepsis treatment system (STS) was included to respond to such changes in the treatment system, and CQs on the system of treating sepsis were included. The basic thought process underlying the STS is that the early recognition and awareness of sepsis and its treatment using an appropriate system leads to improvements in treatment performance. Guidelines also play an important role in activities of awareness such as increasing awareness and recognition of the severity of sepsis or the significance of creating appropriate treatment systems even for the general public and medical professionals who are not involved in sepsis treatment. Furthermore, the ways in which sepsis treatment should be evaluated to ensure diagnostic and treatment quality are included in this section. All medical staff must be able to use the sepsis early detection system to use it effectively. The rapid response system (RRS) is one that can reliably report changes in a patient’s medical condition and respond immediately to such a report. Therefore, the following two CQs relate to a system of early recognition of sepsis, “What are the methods for detecting sepsis at an early stage in the general ward and ER?” and “What is the role of a rapid response system (RRS) which acts against changes in the condition of patients in the general ward where sepsis is suspected?”, have been presented.

The CQ “Where will sepsis refractory to initial fluid resuscitation be managed?” was presented regarding treatment systems that can be used to suspect sepsis at an early stage and provide intensive care.

Clarifying the quality indicator in the initial treatment of sepsis and appropriately evaluating the treatment process will lead to an improvement in the overall quality of sepsis treatment. Therefore, the CQ, “What are quality indicators for initial treatment of sepsis?” was presented.

It was thought that healthcare professionals as well as the general public are widely aware of the importance of the above-mentioned concepts underlying sepsis and early detection/treatment was important in the prevention and improved prognosis of sepsis. Thus, the CQ, “What kinds of activities raise awareness of sepsis?” was presented. Collaboration between the Global Sepsis Alliance and the World Health Organization as well as initiatives by academic societies in Japan are discussed.

**Fig. 20:**
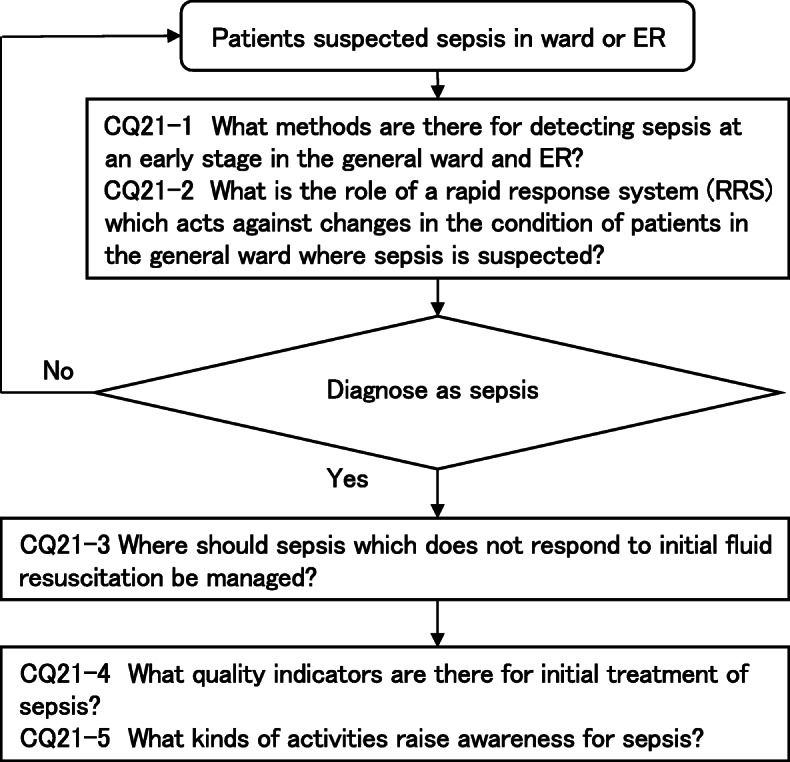
CQ21: Sepsis Treatment System (clinical flow)

Clinical flow of these CQs is shown in Fig. 20.

**CQ21-1: What methods are there for detecting sepsis at an early stage in the general ward and ER?**

***Answer:*** Screening tools such as qSOFA and the early warning score are available as methods which can detect sepsis at an early stage in general wards and in the ER (Provision of information for background question).

Early stage detection and intervention in sepsis are essential for improving the associated mortality rate. Early stage detection of sepsis enables the institution of early stage intervention such as fluid resuscitation and antibacterial drug administration, which can improve patient outcomes [976]. A definition of sepsis based on SIRS was proposed in 1991. However, there are some problems with SIRS, such as its low specificity as a tool for early stage detection of sepsis [977], and in the general ward, only approximately half of patients with sepsis are able to fulfill the two criteria for SIRS [198].

In 2016, along with Sepsis-3, the qSOFA score was proposed as a screening tool for suspected sepsis on the general wards and ER with fewer categories than SIRS [978].

It has been reported that the qSOFA is a more accurate predictor of early detection of sepsis and in-hospital mortality compared to SIRS, the Logistic Organ Dysfunction System (LODS), and the SOFA score [979].

A positive qSOFA score had high specificity outside the ICU in the early detection of in-hospital mortality, acute organ dysfunction, and ICU admission [978].

Furthermore, a meta-analysis of six studies comparing the qSOFA score and SIRS favored the qSOFA score (RR 0.03; 95%CI 0.01 to 0.05; *P* = 0.002) as a predictor of in-hospital mortality [980]. In contrast, the qSOFA may have low sensitivity for recognizing sepsis [981]. Furthermore, the rapid response system (RRS) is a system that detects and responds to suddenly changing cases in the hospital, including those of sepsis, at an early stage. The National Early Warning Score (NEWS), which was published in the UK by combining a number of indicators among these activation tools and outputting a score, was also assessed as an early stage detection tool for sepsis [982]. The NEWS was significantly better at predicting in-hospital mortality among patients with primary infections according to Redfern et al. (receiver operating characteristic area under the curve, NEWS: 0.805 vs. qSOFA: 0.677) [983]. Presently, screening for early stage detection should use scoring systems that can be implemented at each respective facility.

**CQ21-2: What is the role of a rapid response system (RRS) which acts against changes in the condition of patients in the general ward where sepsis is suspected?**

***Answer:*** The rapid response system (RRS) is a system which detects and responds to changes in the condition of patients in the hospital, and there is an opinion where its introduction is expected to improve prognosis of patients even for sepsis (Provision of information for background question).

***Rationale***

The mortality rate attributable to sepsis has been decreasing steadily due to the spread of treatment standardization through the SSCG [972]. Further decreases in the mortality rate in the future would require other approaches in addition to following standard treatment.

Early recognition and treatment interventions for sepsis are important alongside the spread and compliance with standard sepsis treatment, and these are essential for improved prognosis [984]. Changes in the sepsis diagnostic criteria in 2016 led to sepsis being defined as a pathological condition that progressed to organ failure due to infection (Sepsis-3). Sepsis-3 uses the SOFA score in organ failure assessment. This in turn requires blood tests and blood gas analysis, which are time-consuming procedures, making this a complicated option for screening. As such, a simple screening method that can be used at the bedside is needed for general hospital wards and ERs. These types of patients who progress to organ failure often exhibit some form of vital sign abnormalities from the early stage of sepsis. Thus, the qSOFA, which is based on simple vital signs (systolic blood pressure, respiratory rate, and level of consciousness), is recommended as a bedside screening tool for patients with suspected sepsis.

Meanwhile, the RRS is a system that recognizes pathological changes such as vital sign abnormalities in critically ill patients, including those with sepsis at an early stage, and prevents exacerbation of conditions, particularly cardiopulmonary arrest. Generally, the RRS is a system that involves the early detection/intervention in pathological changes by the physician as well as multi-disciplinary medical staff (e.g., nurses, physiotherapists, pharmacists, and clinical engineering technicians), medical students, and patients’ families. It was introduced overseas in the 1980s but was not introduced in Japan until the 2000s, where the construction of an RRS during sudden in-hospital changes was recommended with the action goals of the Japanese Coalition for Patient Safety (“PARTNERS”), with it only recently becoming widespread.

The RRS activation criteria are expected to vary according to each medical facility; however, it is generally activated by recognizing one of multiple vital sign abnormalities in respiration, circulation, consciousness, etc. Often, qSOFA categories such as systolic blood pressure, respiratory rate, and consciousness level are included among these. As such, sepsis screening is also possible through RRS activation when infection is suspected. Furthermore, the Early Warning Score (EWS), which assigns individual weights to multiple vital signs and scores them, is often used in the RRS activation criteria. Sepsis is suspected in the NEWS, used in the RRS proposed by the UK National Health Service (NHS), when a total score of five or higher, or three or more points in any single category, is obtained [985]. No RCT has investigated the efficacy of the RRS and sepsis screening; however, there are reports showing that an RRS enabled early treatment of sepsis/septic shock, leading to an improved prognosis [986]. Furthermore, a report has indicated that the Modified Early Warning Score (MEWS), which is used in RRS activation as a score that predicts vital prognosis and ICU emergency admission in patients with suspected infection in the general ward or the ER, and NEWS were superior to the qSOFA score and SIRS categories [987].

Early sepsis detection in the general ward or ER with the RRS allows for the achievement of the recommended treatment with the 1-h bundle, and an improved vital prognosis of sepsis may be possible as a result.

**CQ21-3: Where should sepsis which does not respond to initial fluid resuscitation be managed?**

***Answer:*** Sepsis which does not respond to initial fluid resuscitation should be managed in a facility where intensive care can be conducted (Good Practice Statement).

***Rationale***

Due to its high morbidity rate, sepsis also requires treatment from medical personnel who are not specialized in intensive care. There are mild cases that can be treated even in general wards. Patients with severe sepsis need to be transferred to hospital beds with a high care level, and a suitable hospital bed needs to be selected by assessing the severity of the disease. There is a concern that environments that cannot sufficiently provide the medical resources needed for treatment (e.g., staffing with appropriate medical skills, monitoring, and equipment including ventilators) may have negative effects on patient prognosis. Appropriate bed selection varies relative to the function, scale, and bed usage situation of each facility; thus, it is not possible to relate severity to appropriate bed classification in a generalized manner [988], but this committee recommends this CQ as a good practice statement in order to provide a necessary intensive treatment. It should be noted that transfer risks, distance, and methods need to be considered when transporting patients out of the hospital.

This recommendation targets patients who did not respond to initial fluid resuscitation, but no high-quality evidence was found in selecting this subject. The Society of Critical Care Medicine ICU Admission, Discharge and Triage Guidelines cites life-threatening sepsis as an example of a level 2C recommendation (suggestion, low evidence level) for admission to the ICU [989]. Considering that this guideline is intended for general medical practitioners who institute treatments in environments without an ICU, and that the criteria would not be effective unless it was as simple as possible due to the diverse phenotype of sepsis, we set a criterion of “when the patient is unresponsive to initial fluid resuscitation” for transfer to a site where intensive treatment is possible. We made the assumption of septic shock, but also considered that lactate levels (which are a requirement by definition) cannot be determined at many facilities. Furthermore, although “unresponsive” is a vague term, we made this recommendation with the decision that some flexibility is needed according to the medical resources available at each facility. The categories of non-responsiveness include persistent hypotension, prolonged disturbances of consciousness, poor respiratory status, and poor lactate clearance. However, it is important to comprehensively determine not only the severity but also the necessary medical resources and recovery prospects [989].

Furthermore, various treatment algorithms in pediatric sepsis management suggest that tracheal intubation, mechanical ventilation, or cardiovascular agents should be initiated after securing a central venous line when the patient is deemed unresponsive to initial fluid resuscitation [848, 984]. Sepsis is a highly fatal condition that induces multiple organ dysfunction, and in the same way that a similar treatment algorithm was shown in the pediatric chapter of this guideline, transitioning to intensive care management when the patient is “unresponsive to initial fluid resuscitation” is likely valid. In other words, if it is possible to transfer a patient to a bed at a hospital with intensive care and there is an ICU nearby that is capable of managing severely ill children, transfer out of the hospital to that unit should be considered. This is known to be correlated with an increased number of patients and a favorable treatment performance for severely ill pediatric patients, in addition to those with sepsis [990–993]. Furthermore, reports have indicated that vital prognosis does not worsen if a team with the skills and equipment to transfer severely ill children do this [994–996], and this should be considered when examining the adequacy and methods of inter-hospital transport.

Evidence of the merits of ICU treatment is limited to observational studies. Reports among patients not limited to those with sepsis include the following [997–1003]. A delay of an hour due to maximum ICU capacity was shown to increase the adjusted risk ratio of ICU mortality to 1.015 (95%CI: − 1.006 to 1.023). Groups in which worsening of symptoms on the general ward to consultation by an ICU team was delayed (> 7.7 h) had an increased 30-day mortality rate (adjusted OR 1.8; 95%CI: 1.1 to 2.9) when compared to groups which experienced no delay (< 1 h). A duration of more than 6 h from when the patient was deemed severely ill with the EWS to ICU transfer increased the in-hospital mortality rate (33.2% vs. 24.5%, *P* < 0.001), with the odds ratio of in-hospital mortality increasing by 3% for every hour of delay. RCTs are virtually impossible in this field, and a consensus was reached with the current evidence that critically ill patients should be managed at the ICU even in the Admission, Discharge, and Triage Guidelines [989]. This is more limited with regard to sepsis, but a report has indicated that every hour of delay from hospital visit to ICU admission in severe septic/septic shock increased the adjusted odds ratio of the mortality rate by 1.11 (95%CI: 1.01 to 1.02) [1004].

The committee has discussed the conditions of “sites where intensive treatment can be conducted”, particularly the ways in which intensive care physicians are involved; however, it is difficult to clarify patient and environmental factors due to their relative nature. Japan’s specific intensive care management fee, pediatric specific ICU management fee, and requirements for emergency care hospitalization charges can be set to a single standard. With regard to the involvement of intensive care physicians, a systematic review reported a decrease in the in-hospital mortality rate (RR 0.83; 95%CI: 0.70 to 0.99) and a reduced hospital stay (weighted mean difference of − 0.17 days; 95%CI: − 0.31 to 0.03) was reported in the high-intensity model (closed ICU in which the intensive care physician has decision rights, or a consultation with the intensive care physician is required for all patients) relative to the low intensity model (open ICU in which each department is managed independently, or where there is no intensive care physician) [1005, 1006]. However, there have also been reports that indicated the correlation between intensive care physician interventions and increased in-hospital mortality rate, and the risk of decreased quality of treatment by intensive care physicians due to their excessive tests/procedures, or the insufficient transfer of patient information [1007].

Furthermore, the effects of the high-intensity model varied according to the specialization of the ICU, region, and the year in which the study was conducted [1006]. Although there is an extremely limited amount of research on sepsis, a multi-center study conducted in Japan (FORECAST) reported that the closed ICU had a higher compliance rate for the 3-h bundle (adjusted OR 2.84, 95%CI: 1.28 to 6.28) [417].

**CQ21-4: What quality indicators are there for initial treatment of sepsis?**

***Answer:*** Quality indicators for initial treatment of sepsis include implementation rates for each indicator, such as blood culture collection, lactate level measurement, early administration of antimicrobial drug, initial fluid resuscitation, and repeated intravascular volume/cardiac function assessment (Provision of information for background question).

***Rationale***

It is important to make assessments using a treatment quality indicator (QI) created by considering appropriate treatment strategies and desirable outcomes to improve the quality of treatment. Detecting sepsis at an earlier stage and progressing with treatment that follows the EGDT protocol was thought to be effective in conventional initial-stage treatment of sepsis, and has been recommended in previous guidelines [984]. However, as shown in the results of the ProCESS trial published in 2014, it was confirmed that prognosis did not improve with the EGDT protocol [314–316]. With a focus on these results, in 2015, the Center for Medicare and Medicaid Services (CMS) at the Department of Health and Human Services (HHS) of the United States government established the Severe Sepsis and Septic Shock Early Management Bundle (SEP-1) in the Hospital Inpatient Quality Reporting Program as a QI for sepsis treatment [1008]. Since then, the strategy has changed from a conventional protocol-based treatment to advance the achievement of the treatment bundle. As such, each of the items taken up as a bundle is important from the perspective of monitoring treatment quality in sepsis. The SEP-1 QI has six items: (1) blood culture implementation, (2) lactate level measurement and (3) appropriate antibacterial drug administration within 3 h of sepsis onset; (4) 30 mL/kg of fluid resuscitation in cases of septic shock, (5) repeated lactate level measurements within 6 h if initial lactate levels exceed 2.0 mmol/L, and (6) use of vasoactive agents when hypotension is prolonged [1009]. Furthermore, although not stated in SEP-1, the initial response to septic shock does not only include the possible need for fluid resuscitation, but also intravascular volume and cardiac function assessments using ultrasound tests [1010].

Recent reports indicated a decrease in mortality rate when antibacterial drugs were administered within 1 h [1011], and early stage lactate level measurements enabled early stage treatment intervention and improved patient prognosis [1012]. However, reports that investigated the achievement levels of each item in SEP-1 and sepsis prognosis indicated that the QI other than that for broad-spectrum antibacterial drug administration within 3 h [1013] had a poor basis for improving treatment effects [1014]. Furthermore, it is necessary to assess the suitability of early stage antibacterial drug administration [1015] As seen above, the current state is such that appropriate QIs have not been clarified even overseas.

**CQ21-5: What kinds of activities raise awareness for sepsis?**

***Answer:*** There have been events like “World Sepsis Day” for the general public and seminars for healthcare professionals held, taking the lead by the Global Sepsis Alliance and World Health Organization (WHO) (Provision of information for background question).

***Rationale***

The Surviving Sepsis Campaign, which began in 2002, has spread its concepts of sepsis and standard treatment globally through its SSCGs since 2004; however, guidelines alone do not bring about sepsis prevention and early stage detection, and the fact that many lives were lost without sepsis being recognized was an issue. In 2010, the Global Sepsis Alliance (GSA) was formed in Europe with the objective of widely communicating sepsis concepts and prevention/early stage detection not only among medical practitioners but also among the general public [1016].

Under its slogan, “Stop sepsis, save lives!”, the GSA conducted public awareness activities, setting five goals to be achieved by 2020: (1) Sepsis incidence would have reduced (by 20%) globally thanks to effective prevention strategies. (2) Sepsis survival is on the rise (by 10%) around the world for adults, children, and newborns. (3) People everywhere will have improved access to appropriate rehabilitation services. (4) Public and professional understanding and awareness of sepsis will have risen, and (5) measurement of the global burden of sepsis and the positive impact of sepsis control and management interventions will have improved. While the objective of the SSCG was to disseminate standard treatment, the objective of the GSA was to communicate everything from sepsis prevention and early stage detection to treatment in a manner that is easy to understand to the general public and medical practitioners not associated with the ICU. For this reason, the GSA set September 13th as “World Sepsis Day” and has hosted events relating to sepsis across the world on this day. The GSA has also called on the World Health Organization (WHO) for cooperation, and in 2017, sepsis was recognized as a “globally urgent problem to be solved” at the WHO General Meeting.

In 2020, the GSA set six new goals to be achieved by 2030 [976]: (1) The global incidence of sepsis will decrease through strategies to prevent infection. (2) Governments will ensure that the three pillars of infection management (infection prevention, antimicrobial stewardship, and the urgent recognition and management of sepsis) will be considered jointly at the policy level. (3) Sepsis survival will increase among children (including neonates) and adults in all countries through the promotion and adoption of early recognition system and standardized emergency treatment. (4) Access to appropriate rehabilitation services will have improved for all patients worldwide. (5) Public and professional understanding and awareness of sepsis will improve; and (6) measurement of the global burden of sepsis and the impact of sepsis control and management interventions will have improved significantly. Moving forward, the GSA will work towards these objectives with the WHO and call for infection prevention and sepsis measures in each nation.

Primarily with the GSA committee, the Japanese Society for Intensive Care Medicine has hosted public events for “World Sepsis Day” and “Sepsis Seminars” for medical practitioners since 2013. The Japanese Association for Acute Medicine became involved in the activities of the GSA from 2018, followed by the Japanese Association for Infectious Diseases in 2019, and these activities have evolved into a “Japanese Sepsis Alliance” (JaSA), conducted jointly by these three associations/societies [1017]. JaSA engages in activities which communicate sepsis treatment guidelines and sepsis knowledge to medical practitioners and citizens through sepsis seminars, public lectures, and their website [1017].

**CQ22: Stress Ulcer Prophylaxis**

**Introduction**

Upper gastrointestinal bleeding associated with stress ulcers is a problem among critically ill patients such as those with sepsis. Recent improvements in the quality of management for these patients have decreased the incidence of upper gastrointestinal bleeding to 2–5% [1018]. However, preventing stress ulcers is important because the onset of upper gastrointestinal bleeding was correlated with an increased mortality rate [1019]. Preventative methods include the administration of anti-ulcer drugs such as acid-suppressive medications, antacids, and mucosa protective anti-gastric ulcer drugs. However, changes in bacterial gut flora can occur due to increases in the pH of gastric acid following the administration of anti-ulcer drugs, and this can promote the colonization of pathogens that cause ventilator-associated infections in the stomach, trachea, and bronchi, and in turn increase the risk of ventilator-associated pneumonia [1020]. Among acid-suppressive medications, proton pump inhibitors (PPIs) may also increase the risk of *Clostridioides difficile* infection [1020]. In this way, there are both benefits and harms in prevention using anti-ulcer drugs; thus, this was verified in CQ22–1. This systematic review included histamine 2 (H_2_) receptor blockers, PPIs, and sucralfate as anti-ulcer drugs; however, there have not been any investigations on the superiority of any of these drugs. Regarding the relative superiority of these drugs, PPIs were shown to have the highest preventative effects against upper gastrointestinal bleeding through network meta-analysis; however, they have been reported to potentially increase the risk of pneumonia, and this should be used as a reference [1021].

Finally, another important CQ is how long prevention should be continued with anti-ulcer drugs once started. Thus, we provided information about the risks of peptic ulcers, the necessity of anti-ulcer drugs, the adverse effects of anti-ulcer drugs, and the relationship between enteral nutrition and anti-ulcer drugs in CQ22–2 as a BQ.

**Fig. 21:**
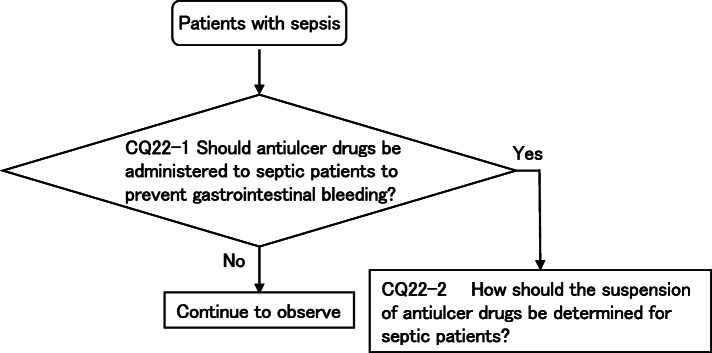
CQ22: Stress Ulcer Prophylaxis (clinical flow)

Clinical flow of these CQs is shown in Fig. 21.

**CQ22-1: Should antiulcer drugs be administered to septic patients to prevent gastrointestinal bleeding?**

***Answer:*** We suggest administering antiulcer drugs to septic patients to prevent gastrointestinal bleeding (GRADE 2B: certainty of evidence = “moderate”).

We performed a meta-analysis of 30 RCTs [1022–1051]. The estimated values of desirable anticipated effects were as follows: gastrointestinal bleeding yielded a RD of 44 fewer per 1000 (95%CI: 54 fewer to 28 fewer) (14 RCTs, *n* = 4884); mortality yielded an RD of 3 more per 1000 (95%CI: 22 fewer to 33 more) (8 RCTs, *n* = 4314). Meanwhile, the estimated values of the undesirable anticipated effects were as follows: pneumonia yielded an RD of 4 more per 1000 (95%CI: 16 fewer to 28 more) (8 RCTs, *n* = 4286); *Clostridioides* infection yielded an RD of 4 fewer per 1000 (95%CI: 9 fewer to 5 more) (3 RCTs, *n* = 3607); various serious adverse effects yielded an RD of 5 more per 1000 (95%CI: 6 fewer to 20 more) (7 RCTs, *n* = 4143). Considering the balance of these benefits and harms, we thought that administering antiulcer drugs was likely superior compared with placebo.

**CQ22-2: How should the suspension of antiulcer drugs be determined for septic patients?**

***Answer:*** The specific decision criteria for suspending antiulcer drugs are unclear. Clinical decision criteria include when bleeding risk factors have decreased, side effects such as pancytopenia or liver dysfunction have occurred, and when sufficient enteral nutrition was able to be administered (Provision of information for background question).

***Rationale***

*Risks of peptic ulcer and the need for anti-ulcer agents*

Peptic ulcers occur when the body is subjected to stress. Ulceration increases the risk of bleeding because insults such as sepsis are often accompanied by dysfunctional hemostasis/coagulation, such as thrombocytopenia and DIC. The clinical factors that determine whether anti-ulcer agents may be discontinued include whether the general condition improves and the patient enters a recovery phase, if the risk of ulceration is reduced, or if hemostatic coagulation dysfunction is improved and the risk of bleeding is reduced. Meanwhile, anti-ulcer drugs should be carefully discontinued in the following cases: 1) administration of drugs with adverse effects of ulcer formation, such as steroids or non-steroidal anti-inflammatory drugs (NSAIDs), 2) administration of anticoagulants or antiplatelet agents, 3) patients with a history of ulcers, or 4) when there are concerns about disorders of gastric or duodenal blood flow [1052].

*Adverse effects of anti-ulcer agents*

Pancytopenia and hepatic dysfunction may be clinical problems as adverse effects of anti-ulcer agents such as PPIs and H_2_ receptor blockers. Differentiation is needed in critically ill patients as other factors may present with similar symptoms. Patients among whom PPIs or H_2_ receptor blockers are thought to be the cause recover relatively quickly with the discontinuation of drug administration, and some reports have stated that patients recovered in an average of 7 days after the discontinuation of the drug [1053]. Thus, the adverse effects of drugs may determine the discontinuation of anti-ulcer agents. Drugs of another class should be used (e.g., PPI to an H_2_ receptor blocker), if the risk of peptic ulcers is high even with adverse effects due to anti-ulcer agents. Drugs should be changed to those with relatively few adverse effects (e.g., gastric protective agents) when the risk of peptic ulcers is deemed to be low.

*Relationship between enteral nutrition and anti-ulcer agents*

The gastric pH decreases in an empty stomach and increases following food intake. In critically ill patients, such as those with sepsis, the lack of increase in gastric pH due to fasting is thought to be the cause of peptic ulcer formation, in addition to various stressors. Therefore, the administration of anti-ulcer agents is a logical decision because the stomach pH does not easily increase during fasting or when enteral nutrition doses via gastric administration are minimal. It has been reported that enteral nutrition via gastric administration has a gastric acid buffering effect similar to that of a regular diet, and the continuous administration of enteral nutrition has the potential to increase the pH more than H_2_ receptor blockers and PPIs in critically ill patients [1054]. Based on these results, it is expected that the gastric pH would increase with sufficient amounts of enteral nutrition via gastric administration, which may be a factor in deciding to discontinue anti-ulcer agents. A recent meta-analysis reported that there were no significant differences in the incidence of gastrointestinal bleeding between patients who received enteral nutrition alone and those who received enteral nutrition with anti-ulcer agents; instead, groups who received concomitant anti-ulcer agents had a significantly higher risk of pneumonia [1055]. Meanwhile, increases in pH due to nutrition are thought to be unlikely when enteral nutrition is administered via the jejunum; thus, the administration of anti-ulcer agents may be necessary, but there is insufficient evidence.

## Data Availability

Additional file 1: Financial and academic COIs and roles of committee members (https://www.jsicm.org/pdf/guidelineEN/Additionalfile1.pdf) Other supplementary materials and files associated with the clinical practice guideline can be found at https://www.jsicm.org/pdf/J-SSCG2020_supplementary_appendix01.pdf.
